# Severe Acute Respiratory Syndrome Coronavirus 2 Diagnostic Tests for Border Screening During the Very Early Phase of Coronavirus Disease 2019 Pandemic: A Systematic Review and Meta-Analysis

**DOI:** 10.3389/fmed.2022.748522

**Published:** 2022-02-14

**Authors:** Pearleen Ee Yong Chua, Sylvia Xiao Wei Gwee, Min Xian Wang, Hao Gui, Junxiong Pang

**Affiliations:** ^1^Saw Swee Hock School of Public Health, National University Health System, National University of Singapore, Singapore, Singapore; ^2^Centre for Infectious Disease Epidemiology and Research, National University of Singapore, Singapore, Singapore

**Keywords:** diagnostic accuracy test, systematic review, acute respiratory infection, COVID-19, molecular test, serologic test, sensitivity and specificity

## Abstract

Diagnosis of severe acute respiratory syndrome coronavirus 2 (SARS-CoV-2) during border screening among returning residents and prioritized travelers during the early phase of a pandemic can reduce the risk of importation and transmission in the community. This study aimed to compare the accuracy of various SARS-CoV-2 diagnostics and assess their potential utility as border screening for infection and immunity. Systematic literature searches were conducted in six electronic databases for studies reporting SARS-CoV-2 diagnostics (up to April 30, 2020). Meta-analysis and methodological assessment were conducted for all included studies. The performance of the diagnostic tests was evaluated with pooled sensitivity, specificity, and their respective 95% confidence intervals. A total of 5,416 unique studies were identified and 95 studies (at least 29,785 patients/samples) were included. Nucleic acid amplification tests (NAAT) consistently outperformed all other diagnostic methods regardless of the selected viral genes with a pooled sensitivity of 98% and a pooled specificity of 99%. Point-of-care (POC) serology tests had moderately high pooled sensitivity (69%), albeit lower than laboratory-based serology tests (89%), but both had high pooled specificity (96–98%). Serology tests were more sensitive for sampling collected at ≥ 7 days than ≤ 7 days from the disease symptoms onset. POC NAAT and POC serology tests are suitable for detecting infection and immunity against the virus, respectively as border screening. Independent validation in each country is highly encouraged with the preferred choice of diagnostic tool/s.

## Introduction

Rapid and accurate diagnosis of Severe Acute Respiratory Syndrome Coronavirus 2 (SARS-CoV-2) infection is crucial to contain the spread and inform clinical decisions to manage the patients' coronavirus disease 2019 (COVID-19) disease severity (1, 2). Diagnostics play an essential role during the border screening to prevent importation and transmission in the community. Many countries have implemented travel restrictions, such as the closure of borders or limiting entry into the country, to control the spread (3). However, these are unsustainable measures as the pandemic evolves over time (4, 5).

Active border screening of SARS-CoV-2 contributes to minimizing potential community transmission from the importation of cases and assist in priority travel. These screening tests may come in different forms. First, it could be *via* nucleic acid amplification tests (NAAT) which involve detection of viral genome in nasopharyngeal and oropharyngeal swabs through reverse-transcription PCR (RT-PCR). This is currently the reference standard for the diagnosis of SARS-CoV-2 infection. Tests typically target the envelope (E), nucleocapsid (N), spike (S), RNA-dependent RNA polymerase (RdRp), and open reading frame 1 (ORF1) genes (6). Second, it could be *via* serology tests, which involve the detection of IgM and IgG antibodies against SARS-CoV-2 in the blood typically 6–7 days after disease onset (7). These antibodies are likely to remain detectable in the blood even after at least 8 months (8, 9). Third, it could also be *via* chest imaging, screening suspected patients for features of COVID-19 infection as RT-PCR was limited during the early pandemic (10). As PCR performance and capacity improves, imaging can be reserved to diagnose and monitor patients with severe condition or poorer prognosis (10). Incorporating artificial intelligence into imaging gives rise to another potential diagnostic test as it increases diagnosis efficiency and accuracy, especially during the very early pandemic phase (10).

Currently, there are limited systematic and comprehensive assessments on the progress and status of diagnostic tests development at the very early phase of the COVID-19 pandemic for border screening (11, 12). Therefore, this review aimed to compare the accuracy of different diagnostic tests (molecular, serology, clinical features, point of care testing, and imaging) and assess their potential utility as border screening for infection and immunity against SARS-CoV-2.

## Materials and Methods

### Search Identification and Selection

This study was conducted with reference to Cochrane's Preferred Reporting Items for Systematic Reviews and Meta-Analyses (PRISMA) guidelines. Systematic searches were conducted in PubMed, Cochrane Library, Scopus, and Embase databases for published literature, and BioRvix and medRvix databases for grey literature on April 30, 2020. Since no restriction was set on the time period, the search timeline was from the point of database inception to April 30, 2020. The search keywords such as “COVID-19,” “2019-ncov,” “SAR-CoV-2,” “diagnos^*^,” “polymerase chain reaction,” “serology,” “point of care,” “computed tomography,” “sensitivity,” and “specificity” were used to identify and extract articles that assessed diagnostic accuracy of existing COVID-19 diagnostic tools as presented in Supplementary Tables S1, S2. Reference lists of relevant reviews were hand-searched to identify any additional studies.

The screening was done in duplicate by three authors (PEYC, MXW, SXWG). Identified publications were hierarchically screened according to the following criteria, and included in the review if they fulfilled all criteria:

1. Population: Cases are laboratory-confirmed COVID-19 patients with no restriction on the countries, race, age group, and severity. No restriction on control definitions, which may or may not be tested for COVID-19. Controls in this review include (i) laboratory-confirmed negative COVID-19 negative patients, (ii) pre-pandemic controls without clinical suspicion of COVID-19, (iii) controls with other confirmed infections, or (iv) healthy controls.
2. Intervention/exposure: Diagnostic tests (including Point-of-Care tests) to identify and/or confirm SARS-CoV-2 infection; no restriction on the time points of the infection and sampling sites. Diagnosis tests for prognosis will be excluded.
3. Outcome: Clinical sensitivity and specificity.
4. Comparator: SARS-CoV-2 samples or patients confirmed by nucleic acid tests or next-generation sequencing.

**Figure 1:**
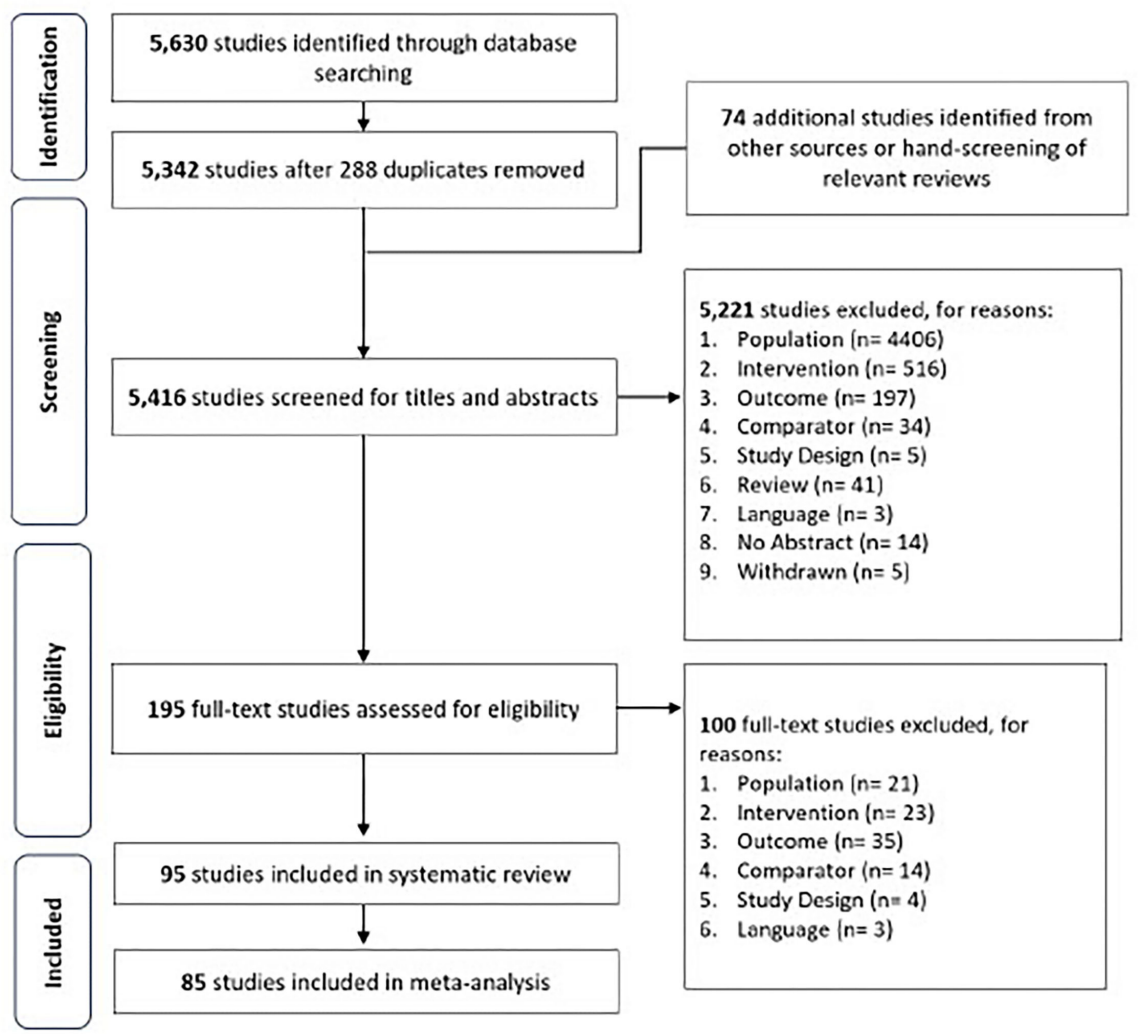
Flow diagram of study selection.

Disagreements were discussed with the fourth author (JP) to reach a final consensus. This review defines the reference standard test as either PCR or sequencing. Point-of-care (POC) diagnostics are defined primarily as tests that can be conducted at the point of patient care, outside laboratory settings. Titles and abstracts of extracted studies were first assessed for relevance before full texts of relevant studies were retrieved further screening with the above criteria. If available, published versions of included preprint studies were used for data extraction. A PRISMA flow diagram of the study selection process is shown in Figure 1.

### Data Extraction

A developed data extraction form was pilot tested with a subset of eligible studies and the finalized form consolidated the following information from each study: author, country of conduct, study and population characteristics, the type of index diagnostic test, and their criteria/cut off, and outcome measures. Outcome measures include true positive (TP), true negative (TN), false positive (FP), false negative (FN), sensitivity, specificity, and any other key findings.

Each index test assessed in included studies was extracted as an individual study, i.e., if multiple index tests were examined in a single study using the same/overlapped population/samples, each index test was considered an independent study with its own dataset. In studies assessing outcomes with control groups, controls tested by the reference test will be preferred, otherwise, a generalizable control such as a healthy population will be selected. In addition, the per-patient and high-performance outcomes will be preferred if alternative data were reported. Lastly, only validation and test set results were extracted from studies assessing diagnostic models or artificial intelligence (AI) trained programs. The summary of findings is presented in Supplementary Tables S3A,B, S12.

### Quality Assessment

The methodological quality of included studies was evaluated with Quality Assessment of Diagnostic Accuracy Studies 2 (QUADAS-2). The original tool includes 14 items assessing the risk of bias, sources of variation, and reporting quality; possible responses to each item were “yes,” “no,” or unclear”(13). Signaling questions and their scoring were adapted from Deeks et al. (14) and McInnes et al. (15) and modified to suit this review. The questions are presented in Supplementary Table S4. Studies were assessed for risk of bias in four domains: patient selection, index test, reference test, and flow and timing. The risk of bias in each domain will be rated for each study; possible domain ratings are low, unclear, or high risk of bias.

Quality assessment was conducted in duplicate by PEYC, MXW, and SXWG. Discrepancies were resolved by consensus with J.P. before moving on to the next stage of analysis.

### Statistical Analysis

The main outcome compared the accuracy of diagnostic tests with different working principles: (i) nucleic acid amplification test excluding sequencing (NAAT), (ii) NAAT POC tests, (iii) sequencing, (iv) serology, (v) serology (POC), (vi) imaging, (vii) imaging with artificial intelligence (AI), (viii) clinical and/ or laboratory features model, and (viii) combination of diagnostic tests. Serology tests (including POC tests) were analyzed based on the detection of immunoglobulin G (IgG), immunoglobulin M (IgM), IgG and/or IgM, and antibodies (Ab). Subgroup analysis was further conducted according to the following: (i) duration of samples collected from the onset of symptoms, early ( ≤ 7 days) and late (≥ 7 days) phase for serology and serology (POC); (ii) gene targets of NAAT and NAAT (POC); (iii) sample collection from different specimen sites for NAAT and NAAT (POC); (iv) differential performance of diagnostic tests on symptomatic and asymptomatic individuals; (v) geographical regions where studies were performed, based on Asia, China, America, and Europe.

For studies with incomplete data, TN, TP, FN, and FP were calculated based on the 2 × 2 table or as much as possible, the inbuilt calculator in Review Manager 5.3. Bivariate analysis was conducted with the provided TN, TP, FN, and FP to generate sensitivity, specificity, diagnostic odds ratio (DOR), and the summary receiver operating characteristic curves (SROC) with their corresponding 95%CI.

In the comparison of diagnostic accuracy across different working principles, forest plots included studies reporting both sensitivity and specificity. Subgroup analyses included studies reporting either one or both sensitivity and specificity. In the subgroup analysis on symptomatic and asymptomatic patients, specificity was not pooled as controls did not display COVID-19 symptoms and cannot be stratified. Continuity correction was performed for DOR and SROC, and sensitivity and specificity when necessary.

Sensitivity, defined as TP/(TP+FN), indicates the proportion of positive cases that the test correctly identifies in COVID-19 subjects (12). Specificity, defined as TN/(TN+FP), indicates the proportion of negative results a test correctly identifies in non-COVID-19 samples (12). The calculation of DOR and SROC utilized both sensitivity and specificity (16). A high DOR indicates good diagnostic accuracy. The area under the curve (AUC) of SROC—which reflects the overall performance of the test—was also calculated. An AUC of one indicates a perfect test.

The I^2^ statistic and Cochrane test were used to evaluate statistical heterogeneity. Heterogeneity was characterized as minimal (<25%), low (25–50%), moderate (50–75%), or high (>75%) and significant if *p* < 0.05. The publication bias for the included studies was assessed through Deek's funnel plot asymmetry test. The slope coefficient with *p* < 0.10 indicated a significant asymmetry. Meta-analysis was conducted. However, this study prioritized and only discussed pooled estimates derived from three or more studies. Full meta-analysis results can be found in the Supplementary Material. Publication bias was conducted when there were more than two studies available.

The R software (mada package: R Foundation for Statistical Computing, Vienna, Austria) and Review Manager 5.3 (RevMan) [Computer program]. The Cochrane Collaboration, 2020.

## Results

### Screening Results and Characteristics of Included Studies

A total of 5,416 unique studies were screened for relevance with their titles and abstracts. Subsequently, 5,221 studies and 100 studies were respectively excluded in the primary and the full-text screening. Studies excluded during full-text screening can be found in Supplementary Table S5. Of the 95 studies eventually included in this review, 85 studies were included in the meta-analysis.

The 95 included studies involved a total of at least 29,785 patients/samples. At least 11 studies recruited asymptomatic patients. Studies included in this review were mostly conducted in China (*n* = 56), Italy (*n* = 10), and the United States (*n* = 6). There were three studies each from Hong Kong and the Netherlands, and two studies each from Japan, the United Kingdom, and Germany. One study was conducted each in South Korea, Slovenia, Spain, Taiwan, France, Canada, Belgium, Sweden, and Denmark. There was one study conducted across two countries, in China and US.

### Comparison Between Diagnostic Tests

**Figure 2:**
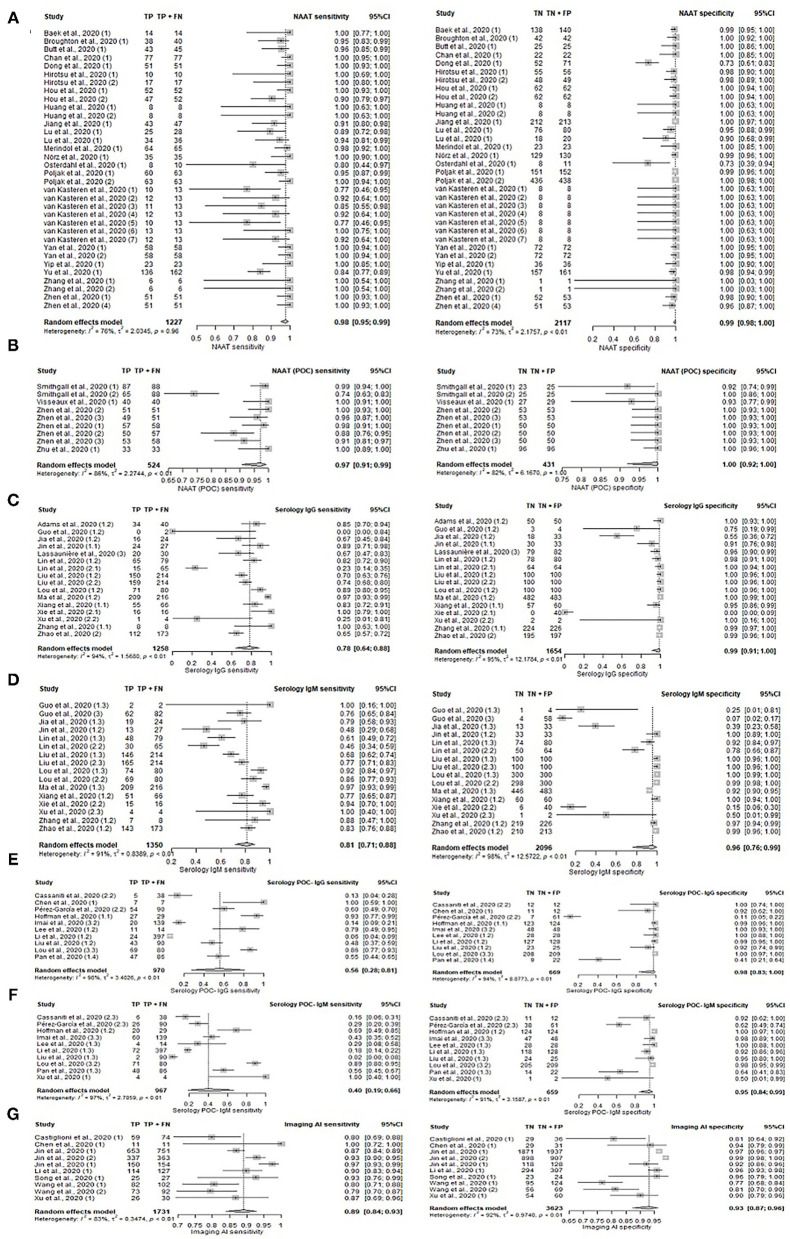
Forest plot on diagnostic accuracy test. Sensitivity and specificity for **(A)** NAAT, **(B)** NAAT (POC), **(C)** serology IgG, **(D)** serology IgM, **(E)** serology IgG (POC), **(F)** serology IgM, and **(G)** Imaging (AI).

A total of 77 unique studies provided all required data and were included in the main comparison of different diagnostic methods tests in the following Table 1, Figure 2, and Supplementary Figure S1. Of all diagnostic methods compared, NAAT (*n* = 34) and NAAT (POC) (*n* = 9) have the highest accuracy in identifying the true positive and negative individuals in their samples: sensitivity [NAAT: 98%, 95%CI: 95–99%; NAAT (POC): 97%, 95%CI: 91–99%] and specificity [NAAT: 99%, 95%CI: 98–100%; NAAT (POC): 100%, 95%CI: 92–100%].

**Table 1:** Pooled estimates for the different diagnostic tests.

**Tests**	**No. of studies**	**Cases (samples/patients)^**∧**^**	**Controls (samples/patients)^**∧**^**	**Sensitivity**	**Specificity**	**DOR**	**SROC**
NAAT	34	1,227	2,117	98 (95, 99)	99 (98,100)	571.94 (293.6, 1,114.1)	0.976
NAAT (POC)	9	524	431	97 (91, 99)	100 (92, 100)	1,265.5 (457.6, 3,500.0)	0.987
Sequencing	2	19	7	100^*^ (82, 100)	43^*^ (10, 82)	NA	NA
		9	4	100^*^ (66, 100)	0^*^ (0, 60)		
Serology IgG and/or IgM	11	1,238	1,252	89 (82, 93)	98 (71, 100)	110.6 (14.9, 818.4)	0.824
Serology IgG and IgM	4	301	569	55 (43, 67)	95 (86, 98)	21.1 (1.7, 264.0)	0.895
Serology IgG	16	1,258	1,654	78 (64, 88)	99 (91, 100)	90.1 (24.6, 329.7)	0.911
Serology IgM	16	1,350	2,096	81 (71, 88)	96 (76, 99)	48.4 (11.1, 211.9)	0.871
Serology Ab	12	1,859	3,857	96 (91, 98)	100 (98, 100)	1,936.7 (605.5, 6,194.6)	0.987
Serology IgA	2	30	82	93^*^ (78, 99)	93^*^ (85, 97)	NA	NA
		216	483	99^*^ (96, 100)	98^*^ (96, 99)		
Serology (POC)-IgG and/or IgM	28	1,532	1,808	69 (61, 77)	96 (92, 98)	37.6 (16.0, 88.0)	0.873
Serology (POC)-IgG and IgM	6	654	1,283	64 (42, 82)	99 (92, 100)	81.2 (9.0, 729.3)	0.928
Serology (POC)-IgG	10	970	669	56 (28, 81)	98 (83, 100)	20.4 (2.7, 154.2)	0.832
Serology (POC)-IgM	10	967	659	40 (19, 66)	95 (84, 99)	8.8 (2.0, 38.7)	0.798
Serology (POC)-AB	1	80	209	97^*^ (91, 100)	95^*^ (91, 98)	NA	NA
Antigen test (POC)-N antigen	1	56	31	98^*^ (90, 100)	100^*^ (89, 100)	NA	NA
Imaging	8	1,122	1,598	82 (65, 91)	62 (47, 75)	7.3 (3.9, 13.6)	0.767
Imaging AI	10	1,731	3,623	89 (84, 93)	93 (87, 96)	104.8 (37.0, 297.7)	0.932
Clinical and/or laboratory	4	564	352	86 (75, 92)	84 (72, 92)	29.2 (9.8, 86.9)	0.906
NAAT + Imaging	1	87	481	91^*^ (83, 96)	67^*^ (61, 72)	NA	NA
Serology + Serology + Serology (POC)-IgG	1	80	100	94^*^ (86, 98)	99^*^ (95, 100)	NA	NA
Serology + Serology + Serology (POC)-IgM	1	80	209	94^*^ (86, 98)	97^*^ (94, 99)	NA	NA
Serology + Serology + Serology (POC)-AB	1	80	209	99^*^ (93, 100)	94^*^ (90, 97)	NA	NA
Serology + Serology-IgM	1	65	64	38^*^ (27, 51)	98^*^ (92, 100)	NA	NA
Serology + Serology-IgG	1	65	64	23^*^ (14, 35)	100^*^ (94, 100)	NA	NA

* *Results were not pooled due to insufficient studies*.

∧ *May consist of overlapping samples/patients due to the recruitment of same population for different test kit within the study*.

Serology tests were generally less sensitive (range 55–98%), while specificity remained high (range 95–100%), depending on the test's targeted antibody. The number of tests with the respective targeted antibody/antibodies was summarized in Table 1. Serology tests concurrently detecting for IgG and/or IgM antibodies (*n* = 11; 89%, 95%CI: 82–93%) was the most sensitive, i.e., accurate in identifying true positives, followed by detecting for IgM only (*n* = 16; 81%, 95%CI: 71–88%), IgG only (*n* = 16; 78%, 95%CI: 64–88%), and for both IgG and IgM (*n* = 4; 55, 95%CI: 43–67%). Sensitivity for tests detecting unspecified Ab antibodies (96%, 95%CI: 91–98%) was also relatively high. Two serology studies involving IgA antibodies had high sensitivities of 93 and 99%, with a high specificity of 93 and 98% respectively. Overall, serology (POC) has low pooled sensitivity ranging from 40 to 69%, albeit high pooled specificity ranging from 95 to 99%.

Antigen test (POC) has a comparative sensitivity 98%, 95%CI: 90–100% and specificity 100%, 95%CI: 89–100% with NAAT (POC). However, this was based on a single study.

The incorporation of AI into diagnostic imaging (*n* = 10; 89%, 95%CI: 84–93%) resulted in superior sensitivity compared to conventional imaging alone (*n* = 8; 82%, 95%CI: 65–91%). Likewise, imaging (AI) had relatively high specificity of 93%, 95%CI: 87–96% as compared to conventional imaging (62%, 95%CI: 47–75%). Diagnostics based on clinical and/or laboratory features (*n* = 4) ranked between imaging (AI) and imaging with its pooled sensitivity of 86% (95%CI: 75–92%) and specificity of 84% (95%CI: 72–92%).

While outperforming conventional imaging, clinical/laboratory feature-based diagnostics still fell below AI incorporated imaging. Nonetheless, imaging methods generally had better sensitivity than most serological methods detecting IgG/IgM.

The use of laboratory-based serology methods together with POC serology for IgG, IgM, and Ab respectively increased sensitivity to 94–99%. However, only one study reported the use of this approach and results should be interpreted with caution.

Sequencing results from two studies were highly sensitive at 100%, but specificity was inconclusive with one study at 43% and the other at 0%. Inconclusive specificity resulted from the non-inclusion of controls in the study sample, while 0% specificity resulted from the novel test's inability to accurately identify true negatives, i.e., controls from the sample. This could be due to the low number of controls (n=4) present in the small study sample of 13 individuals.

### Serology and POC Serology in Early and Late Phases of the Disease

**Figure 3:**
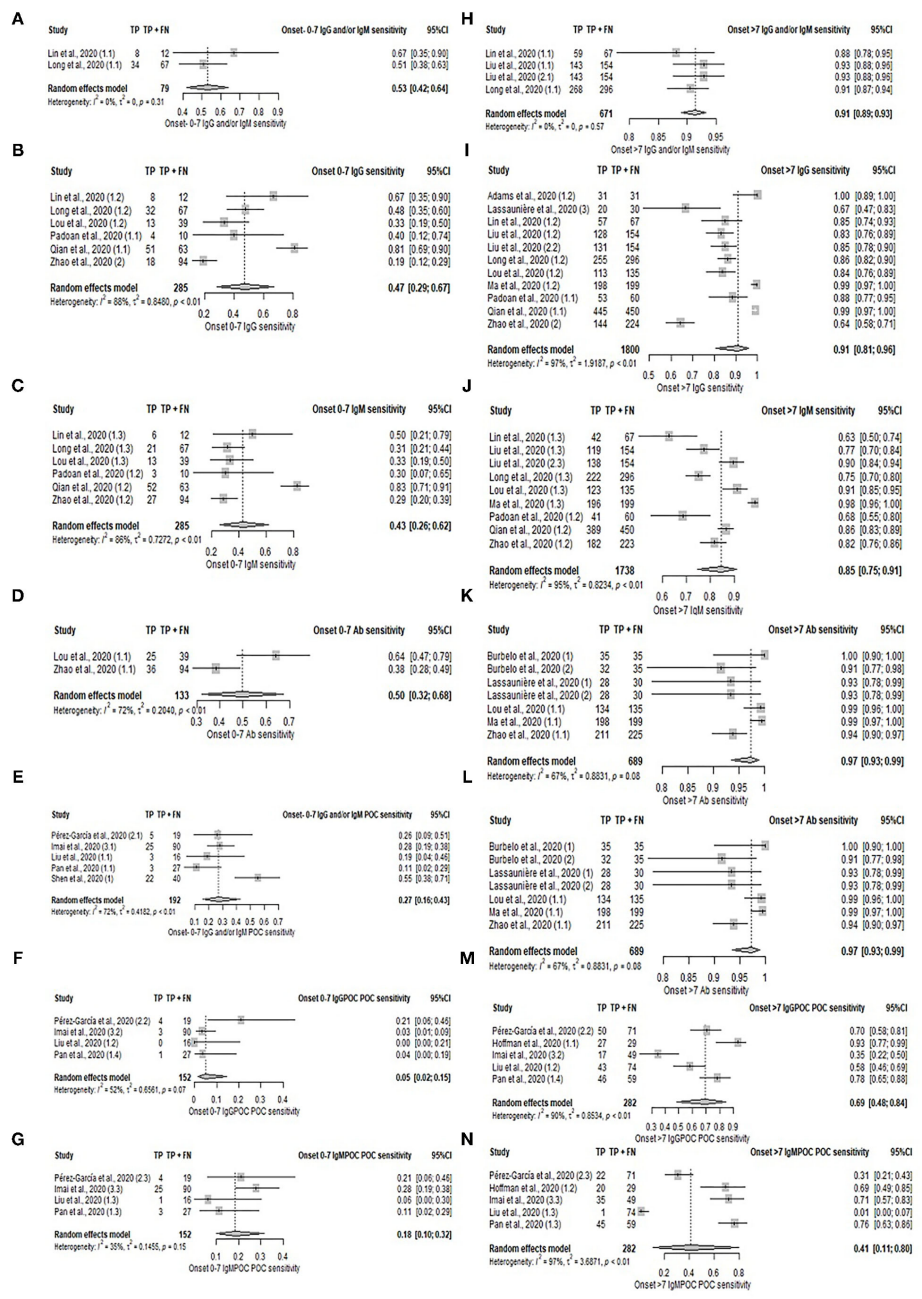
Forest plot on sensitivity of serology test in early and late phases of disease. **(A–G)** Pooled sensitivity for early phase: **(A)** IgG and/or IgM, **(B)** IgG, **(C)** IgM, **(D)** Ab, **(E)** IgG and/or IgM (POC), **(F)** IgG, and **(G)** IgM. **(H–N)** Pooled sensitivity for late phase: **(H)** IgG and/or IgM, **(I)** IgG, **(J)** IgM, **(K)** Ab, **(L)** IgG and/or IgM (POC), **(M)** IgG, and **(N)** IgM.

In the subgroup analysis according to disease onset shown in Figure 3, Supplementary Table S6, and Supplementary Figures S2, S3, laboratory-based serology and POC serology had higher sensitivity in samples taken in the late phase (≥ 7 days) compared to samples taken in the early phase ( ≤ 7 days) of the disease symptoms onset. The pooled sensitivity estimates are as follows; IgG: 91% (95%CI: 81–96%) vs. 47% (95%CI: 29–67%); IgM: 85% (95%CI: 75–91%) vs. 43% (95%CI: 26–62%); IgG and/or IgM (POC): 83% (95%CI: 76–88%) vs. 27% (95%CI: 16–43%); IgG (POC): 69% (95%CI: 48–84%) vs. 5% (95%CI: 2–15%), and IgM (POC): 41% (95%CI: 11–80%) vs. 18% (95%CI: 10–32%). Pooled sensitivity of serology on samples taken during the late phase was 91% (95%CI: 89–93%) for IgG and/or IgM, and 97% (95%CI: 93–99%) for unspecified Ab. Comparison of sensitivity between disease phases was not possible as meta-analysis was limited by the presence of only 2 studies in the early phase period. Their sensitivities are as follows: IgG and/or IgM (67 and 51%) and Ab (64 and 38%). Specificity in POC during the early and late phases could not be compared, as most early phase categories comprised only two studies. The pooled specificities of samples taken during late phase are as follows: IgG and/or IgM (POC): 98% (95%CI: 33–100%); IgG (POC): 77% (95%CI: 15–98%), and IgM (POC): 92% (95%CI: 44–99%). The specificity estimates of early phase studies excluded from the meta-analysis are: IgG and/or IgM (78 and 56%), IgG (89 and 56%), and IgM (100 and 78%). The remaining categories comprised only 1 study and were similarly excluded from the meta-analysis. The specificity of serology tests across stages was not comparable due to the limited studies reporting specificity in the early phase.

### Symptomatic and Asymptomatic Patients

**Figure 4:**
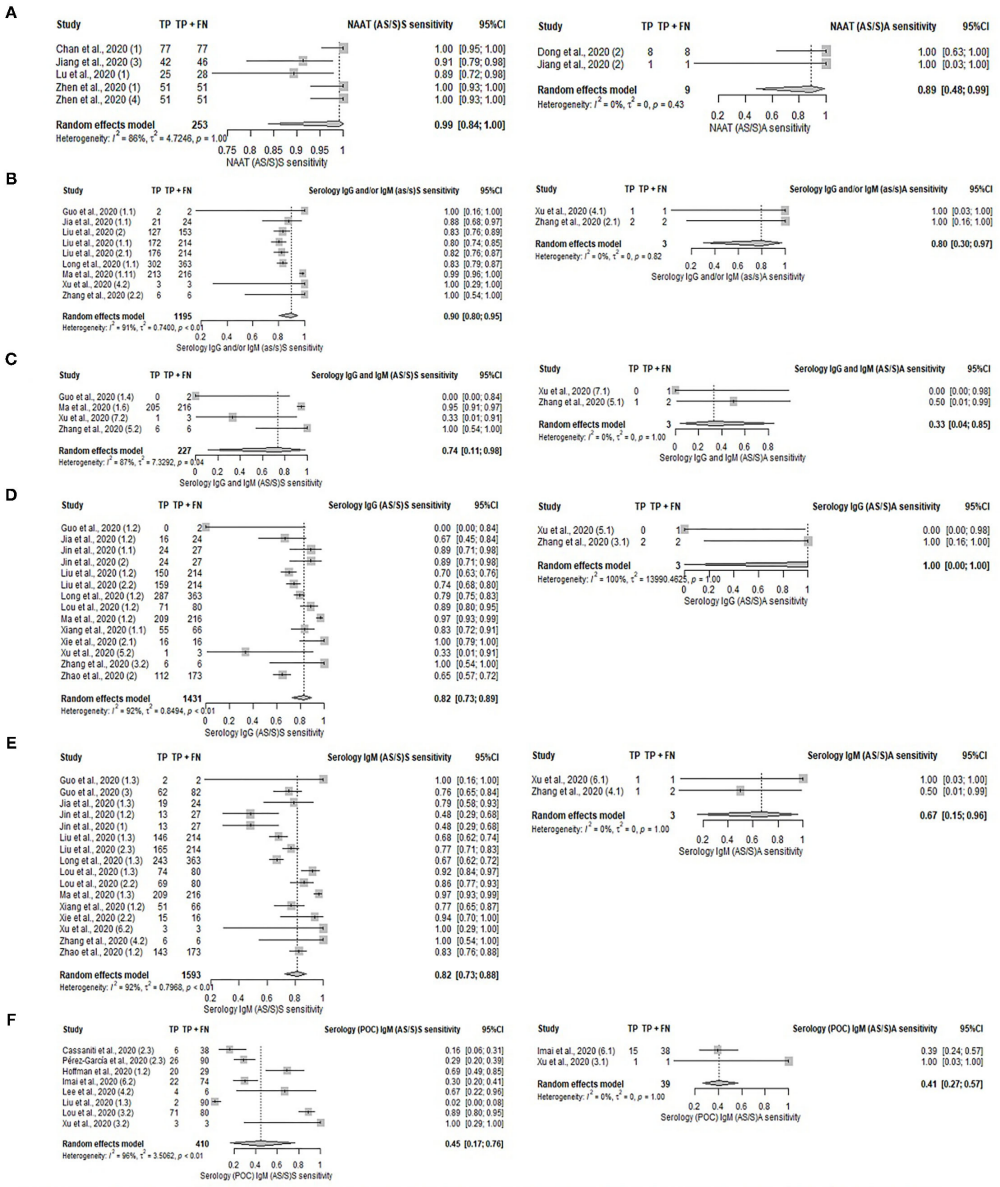
Forest plot on diagnostic test accuracy between symptomatic (left) and asymptomatic (right) patients. **(A)** NAAT, **(B)** serology IgG and/or IgM, **(C)** serology IgG and IgM, **(D)** serology IgG, **(E)** serology IgM, and **(F)** serology IgM (POC).

A comparative analysis of different tests [serology, serology (POC), NAAT, and imaging] was performed between symptomatic and asymptomatic patients. However, the subgroup analysis was limited by the small number of studies involving asymptomatic patients; serology (POC) IgG and/or IgM, serology (POC) IgG and imaging each only had one study with asymptomatic patients. Diagnostic tests were suggestive to be more sensitive in symptomatic patients than asymptomatic patients, as presented in Supplementary Table S7, Supplementary Figure S4, and Figure 4. In terms of the serological detection of IgG/IgM, sensitivities ranged from 74 to 90% in symptomatic patients—[IgG and/or IgM: 90% (95%CI: 80–95%), IgG and IgM: 74% (95%CI: 11–98%); IgG: 82% (95%CI: 73–89%); IgM: 82% (95%CI: 73–88%)]. The pooled sensitivity of the aforementioned tests on asymptomatic patients was not analyzed due to the presence of only two studies for each. Their respective sensitivities are as follows: serology IgG and/or IgM (100% for both), IgG and IgM (50 and 0%), IgG (100 and 0%), and IgM (100 and 50%). Sensitivities of IgG/IgM detection by serology (POC) in symptomatic patients ranged from 45 to 66%; IgG and/or IgM: 66% (95%CI: 41–84%); IgG: 62% (95%CI: 26–88%); IgM: 45% (95%CI: 17–76%). The pooled sensitivity of IgM (POC) on asymptomatic patients was not analyzed due to the presence of only two studies reporting sensitivities of 39 and 100%. The pooled sensitivities of NAAT and imaging for symptomatic patients are 99% (95%CI: 84–100%), and 82% (95%CI: 67–91%) respectively. NAAT studies on asymptomatic patients that were not included in the meta-analysis had sensitivities of 100%.

### Diagnostic Accuracy Across Different Geographical Locations

Studies were categorized into four groups—Asia (excluding China), America (the United States and Canada), China, and Europe (France, Germany, Denmark, Italy, Netherland, Slovenia, Spain, and the United Kingdom). Diagnostic accuracies were compared across the four groups. The performance of NAAT was comparable in terms of estimates, and consistently outperformed most of the other diagnostic methods regardless of region. Meta-analysis comprised predominately of serology IgG used in China observed pooled sensitivity of 80% and a pooled specificity of 97%; pooled sensitivity and specificity of serology (POC) IgG and/or IgM was 80 and 88%, respectively. Other model-based diagnostic methods in China had sensitivity ranging from 85 to 92%, and specificity ranging from 56 to 94%. Serology performance in Europe was only available for IgG, giving a pooled sensitivity of 79% (lower than in China's studies) and specificity of 97%. Serology (POC) IgG and/or IgM in Europe had pooled sensitivity 67% (lower than in China's studies) and a pooled specificity of 97%. Asia (excluding China) and America are limited by the small number of studies. Further details can be found in Supplementary Figure S5 and Supplementary Table S8.

### Gene Target

**Figure 5:**
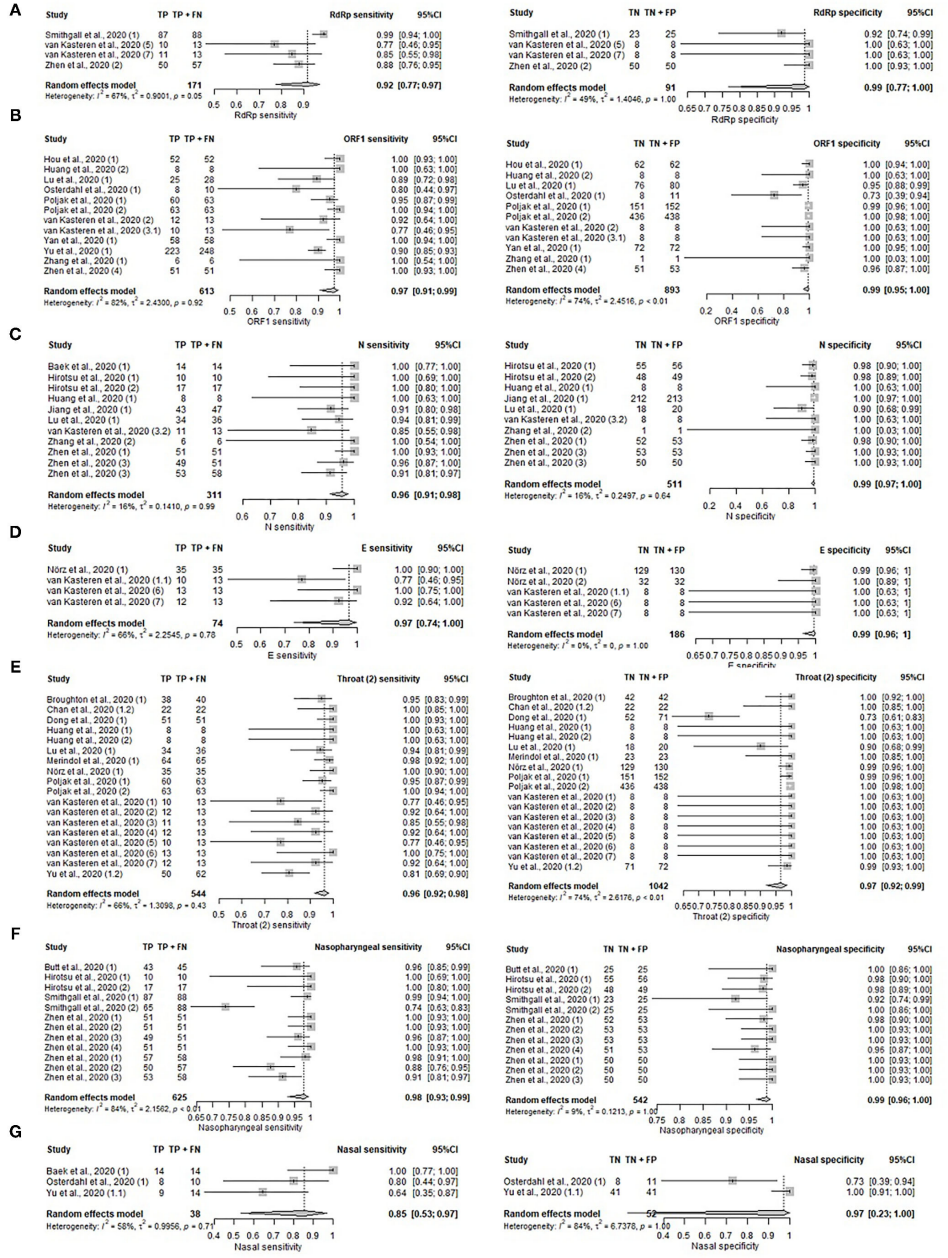
Forest plot on gene targets and specimen sites. **(A–D)** Pooled sensitivity and specificity on gene target. **(A)** RdRp gene, **(B)** ORF1 gene, **(C)** N gene, **(D)** E gene. **(E–G)** Pooled sensitivity and specificity on specimen sites. **(E)** Unspecified throat, **(F)** nasopharyngeal, and **(G)** nasal.

The diagnostic performance of individual genes, N, ORF1, S, RdRp, E, and non-structural protein 2 (Nsp2) were analyzed using data from 17 unique studies, as depicted in Figures 5A–D, Supplementary Table S9, and Supplementary Figure S6. Nsp2 was found to have the highest sensitivity (100%, 95%CI: 85–100%) and specificity (100%, 95%CI: 90–100%), but the data was contributed by only a single study. Otherwise, high sensitivity (92–97%) and specificity (99–100%) were observed across all genes that had sufficient studies for meta-analysis. The sensitivity for the remaining genes were 96% (95%CI: 91–98%) for N gene, 97% (95%CI: 91–99%) for ORF1 gene, 92% (95%CI: 77–97%) for RdRp gene, and 97% (95%CI: 74–100%) for E gene. The specificity for these genes were 99% (95%CI: 97–100%) for N gene, 99% (95%CI: 95–100%) for ORF1 gene, 99% (95%CI: 77–100%) for RdRp, and 99% (95%CI: 96–100%) for E gene. The pooled performance of the S gene was not analyzed as only two studies were available. The sensitivities of the two studies were 77 and 100% respectively, while the specificities were both 100%.

### Specimen Site

The performance of NAAT and NAAT (POC) tests on specimens collected from different sampling sites were compared in Figures 5E–G, Supplementary Table S10, and Supplementary Figure S7. Samples compared include nasopharyngeal (Sensitivity: 98%, 95%CI: 93–99%; Specificity: 99% 95%CI: 96–100%), unspecified throat region (Sensitivity: 96%, 95%CI: 92–98%; Specificity: 97%, 95%CI: 92–99%), nasal (Sensitivity: 85%, 95%CI: 53–97%). Nasal specimens were not assessed for pooled specificity due to the presence of only two studies reporting specificities of 73 and 100%. Sputum collected from the lower respiratory tract was documented in only two studies as well, which reported sensitivities of 100 and 90%. The following estimates were based on a single study: sputum (Specificity: 90%, 95%CI: 73–98%), saliva (Sensitivity: 100%, 95%CI: 91–100%), and stool (Sensitivity: 100%, 95%CI: 40–100%). High sensitivity was observed across all sampling sites with saliva and stool having the highest sensitivity of 100% and nasal with the lowest sensitivity of 85%. For upper respiratory tract specimens, pharyngeal (throat) samples were most commonly collected, followed by nasopharyngeal and nasal samples. As stool and saliva samples only comprised one study each on sensitivity, these sample types were excluded from the pooled analysis. Chan et al. (17) and Yu et al. (18) tested the urine, blood, and plasma of COVID-19 patients, but none were detected positive by the reference test.

### Quality Assessment

Most studies (71.7%) were rated of high risk of bias in the domain of patient selection, largely due to their case-control design. In the index test domain, the majority of the studies (65.5%) had unclear risk mainly due to the uncertainty of blinding to reference test results during the interpretation of the index test result. We were unable to ascertain the risk of bias in 89.4% of studies in the reference standard domain since most did not report on targeting two gene sites or testing the negative samples twice for the reference tests conducted. In the flow and timing domain, the majority of the studies (49.6 %) were at high risk of bias as control samples in most studies were not definitively tested by the reference test; control samples were from the pre-pandemic period, those positive for other diseases or healthy volunteers. The results are shown in Supplementary Figure S8 and Supplementary Table S11.

The applicability of studies to this review was assessed across the domains of patient selection, reference standard, and index test used. Studies were rated as low, high, or unclear levels of concern regarding their applicability. In terms of the reference standard, there was little concern about the applicability of all studies utilized in this review. While most studies (58%) were of low level of concern in the index test domain, 40.2 and 1.8% of studies were of high and unclear levels of concern, respectively. This is attributed to the fact that multiple diagnostic kits reported were non-commercialized kits and may be conducted and interpreted differently from commercialized products. The applicability of included studies to our review fared poorly in the domain of patient selection, with only 29.2% graded of low concern. There was a high level of concern for 62% of studies due to the case-control design utilized, which is known to falsely increase sensitivity and specificity through spectrum bias (19). The level of concern for applicability of 8.8% of studies cannot be ascertained.

### Publication Bias

Publication bias analysis were conducted for NAAT, NAAT (POC), serology tests (IgG and/or IgM, IgG and IgM, IgG, IgM and Ab), serology (POC) test (IgG and/or IgM, IgG and IgM, IgG, IgM), imaging, imaging AI, and clinical and/or laboratory modeling. Publication bias was detected only in serology test IgG and IgM (*p* = 0.05), serology test IgG (*p* = 0.01), serology test IgM (*p* = 0.08), and NAAT (*p* = 0.04). No significant bias was observed for serology (POC) IgG and/or IgM (*p* = 0.69); serology (POC) IgG and IgM (*p* = 0.52); serology (POC) IgG (*p* = 0.91); serology (POC) IgM (*p* = 0.93); imaging (*p* = 0.85); imaging (AI) (*p* = 0.21); serology IgG and/or IgM (*p* = 0.13); serology AB (*p* = 0.47); NAAT (POC) (*p* = 0.17), and clinical and/or laboratory model (*p* = 0.75). The results were presented in Supplementary Figure S9.

## Discussion

### Comparison of All Tests

#### NAAT

Our study affirms the better performance of NAAT and NAAT (POC) over other diagnostic tests. NAAT, which detects active infection, has been recommended by the National Institutes of Health (NIH) (20) and Centers for Disease Control and Prevention (CDC) (21). Interestingly, NAAT (POC) retained high sensitivity and specificity despite a shorter workflow. NAAT (POC) included in the review were mostly commercialized test kits using automating RT-PCR, reverse transcription loop-mediated isothermal amplification (RT-LAMP), and an in-house test using mRT-LAMP-LFB. An alternative to RT-PCR, RT-LAMP amplifies RNA under isothermal conditions (22), enabling a simpler, cheaper, and smaller performance device than a thermal cycler (22). There are variants of RT-LAMP utilizing different detection methods such as iLACO assay, One-pot RT-LAMP assay, Integrated RT-LAMP, and CRISPR-Cas12. There is mounting evidence on the potential of RT-LAMP as a POC test as it is simpler, fast, and as sensitive as RT-PCR (22, 23). Nonetheless, NAAT and NAAT (POC) performance should be interpreted with caution. While not many studies in this review reported cycle threshold (Ct) values, NAAT and NAAT (POC) performance may be influenced by the variation in Ct values used to determine positive cases across studies. Thus, cases/controls could be categorized as false negatives/positives depending on the Ct values used in the index tests.

#### Serology Tests

Serology tests detect antibodies against SARS-Cov-2, usually detectable after 1–3 weeks of symptom onset (24). They are not recommended for the diagnosis of acute COVID-19 infections and can delay infection control efforts in the community (20, 21). However, they can be important for epidemiological surveillance since the detection of antibodies against SARS-Cov-2 can indicate past and asymptomatic infections (21). In a typical humoral response, the body first produces IgM within 5–7 days of infection (25). However, for SARS-CoV-2 infection, all three isotypes, IgM, IgG, and IgA can be detected in a narrow timeframe of seroconversion (25). The detection of IgG and IgA before IgM, pointing to weak IgM response in SARS-CoV-2 infections, has been evidenced (25). A study detected specific IgG during the early phase of illness in some individuals, 4–6 days after symptoms (25). These collectively suggest that IgG or IgA detection could be more sensitive than the conventional IgM detection in the early stage of infection (25). As IgA is in charge of mucosal immunity, it is a crucial first-line defense against such respiratory viruses. Studies have reported its detection as early as 3 days after symptoms appear before class-switching to IgG (26). However, CDC refrained from concluding any distinction in assay performance based on immunoglobulin classes, IgG, IgG, and IgM, or total antibody. CDC further recommends against the use of IgA assay due to insufficient information on the dynamics of IgA detection in serum (24). Serological methods are also limited by disease prevalence in the community, which varies with the outbreak duration and virus strain in the country and the effectiveness of mitigation measures (27). Low prevalence in the population, with the mainly asymptomatic general population, may challenge the serology test's accuracy in determining past infection (27). Our findings for serology (POC) corroborate those by Ricco et al., who found commercially available serology (POC) tests with a moderate sensitivity of 64.8%, and high specificity of 98% in IgG/IgM (28). Likewise, WHO does not recommend the use of serology (POC) tests for patient care purposes (29). Serology (POC) tests are based on the lateral flow immunoassay (LFIA) technique—a solid-phase immunoassay combining the principles of thin-layer chromatography and immune recognition reaction (30).

Interestingly, serology (POC) and NAAT (POC) tests' sensitivities differ greatly from their laboratory tests counterparts. Our study noted a similar performance between NAAT and NAAT (POC) while serology (POC) has lower performance when compared to the laboratory method. NAAT (POC), such as Simplexa COVID-19 Direct (Diasorin Molecular LLC, Cypress, CA) (31) and GenMark ePlex SARS-CoV-2 assay (32), essentially automate steps in the workflow of a laboratory assay. The similar performance of the diagnostic tests could be attributed to the same fundamental methodology utilized in the assays. Conversely, serology (POC) relies on the immunochromatographic visualization of lines on nitrocellulose membranes. The simplicity of cassette-like serology (POC) tests is a double-edged sword, as results are visually interpreted. This increases the ease of interpretation but also the subjectivity of the interpretation. Sensitivity is reduced as results are prone to a false negative. Other factors that may interfere with serology (POC) test performance include execution by inadequately trained personnel, which is common in manpower-strained settings, loading of insufficient sample volume due to dropper usage (33), and delayed interpretation of serological assay cassettes (34), which may distort the results initially obtained. The commencement of vaccination programs globally may not entirely affect the use of serology for SARS-CoV-2 diagnosis if the vaccines used do not result in the production of target antibodies (such as anti-N, E, or M) of the serology test. The four vaccines are mainly used in mass vaccination programs, namely those by Pfizer-BioNTech (BNT162b2), AstraZeneca (AZD1222), Gamaleya (Sputnik-V), and Moderna (mRNA-1273) all result in the generation of antibodies against the spike protein (35–37) Moreover, the majority of the registered diagnostic tests with the United States Food and Drug Authority emergency use authorization are detecting other structural genes/proteins, rather than the spike protein. With the potential increasing need to assess for the presence of immunity due to either prior (but not recent infection) infection or due to vaccination at the border, the POC serological tests will become highly relevant with more countries adopting the requirement of the immunity “passport” in the near future.

#### Imaging

Severe acute respiratory syndrome coronavirus 2 (SARS-CoV-2) primarily infects the respiratory system, resulting in lung complications (12). Imaging methods such as CT and x-ray, albeit highly sensitive, are limited by their low specificity. While CT can identify indistinct signs of the disease and at earlier onset as compared to x-ray (38), there is no defined feature to differentiate COVID-19 from other respiratory conditions such as pneumonia or acute respiratory distress (39). In times of pandemic, radiologists are insufficiently exposed to clinical manifestation of the disease to accurately differentiate cases (40). Furthermore, as patients with mild disease presentation or in the early stage of COVID-19 may not present abnormality in their chest radiography, findings might be misled by potential comorbidities (39). Hence, the choice between using CT or x-ray for diagnosis ought to factor in the duration since the onset of symptoms. Nevertheless, using solely imaging to diagnose patients with COVID-19 is not recommended due to variability in chest imaging findings documented to date (41). Imaging should ideally be used for supplementary diagnosis or resource allocation in test-kit constrained settings.

This review is the first to include studies that incorporated AI in diagnostic imaging to the best knowledge of the authors. Imaging (AI) was observed to have higher specificity as compared to conventional imaging as AI algorithms were trained to differentiate COVID-19 from other pneumonia (42). The incorporation of AI in imaging tools can shorten result turnover from minutes to seconds (43), which is crucial in managing the large influx of patients during a pandemic. Given the novelty of AI as an emerging technology in diagnosis, it would be recommended to harness imaging (AI) as a supportive tool for radiologists or as a complementary test for COVID-19.

#### Clinical Features and/or Laboratory Parameters

Clinical features or laboratory parameters have been increasingly utilized in models to diagnose or predict COVID-19. This could overcome the limitations of PCR and assist in test allocation in resource-constrained settings. Clinical manifestations in patients with COVID-19 range from mild symptoms to severe respiratory failure and even death (44). A systematic review by Styurf et al. identified cough, sore throat, fever, myalgia or arthralgia, fatigue, and headache with a respective sensitivity of at least 50% (45). Fever, myalgia or arthralgia, fatigue, and headache were further identified to be at least 90% specific in diagnosing COVID-19, the combination of signs and symptoms was not explored (45).

This review identified the relatively high performance of modeling approaches incorporating clinical features or laboratory parameters. However, the result was highly heterogeneous, possibly due to the spectrum of variables included in each model. Models were either based on laboratory parameters exclusively, like the COVID-19 Assistant Discrimination 2.0 (46), or combined with demographic variables, like the COVID-19 Diagnosis Aid APP (47). The overall variables included in each model ranged from 3 to 11, across the 4 studies included in the meta-analysis. Age was the most common demographic variable, while sex was included only on one occasion (48), lactate dehydrogenase was the most common laboratory parameter. The model by Kurstjen et al. (48), Corona-score, incorporated imaging results by CT and X-ray, while that by Li et al. (49) was the only model that incorporated overt clinical signs like respiratory symptoms and fever. Diagnostic modeling approaches utilizing commonplace laboratory test results could increase the efficiency of COVID-19 testing, which is especially crucial in resource-constrained settings. However, estimates from diagnostic models often risk being overly optimistic and misleading due to suboptimal methodology. A living systematic review by Wynants et al. recommended against using predictive models for current practice and further advised the use of updated patient data from the same setting to prevent miscalibration (50).

### Serology Sample Collection Duration

While serology tests were generally high in sensitivity and specificity; their usage should be considered in relation to the duration since symptom onset. Our review found higher diagnostic sensitivity in patients tested in the late phase as compared to the early phase of the disease. This finding echoes the review by Bastos et al., which found that pooled sensitivity across the immunoglobulin classes increases with the time of sample collection from symptom onset (51). A possible reason is the rapid production of antibodies at later stages of the disease when the viral load decreases with increased seroconversion (52). Zhao et al. demonstrated that the RNA test was the most sensitive during the early phase (within 7 days) of the illness, while serology tests had low positive rates in the early phase, but outperformed RNA tests 8 days following symptom onset (53). These collectively suggest the complementary use of serology test with NAAT test in the later stage of the disease.

### Asymptomatic vs. Symptomatic

Symptomatic patients were likely reviewed in most of the included studies because they were easier to identify and more predisposed to health-seeking behavior. Furthermore, symptom-based testing may be prioritized by health authorities due to limited capacity, especially in the early phase of the pandemic. This study was not able to infer the performance of the diagnostic tests between the symptomatic and asymptomatic patients due to the small number of studies and samples in the asymptomatic group. Nonetheless, pooled results of two studies suggested higher sensitivity of serology and NAAT diagnostic tests in symptomatic patients. Serology tests might be less sensitive on asymptomatic individuals (34, 54, 55) or those with mild disease (56) as they mount weaker antibody responses. Asymptomatic individuals have been observed to produce significantly less IgG/IgM than symptomatic individuals, which potentially limits the performance of serology tests (54, 55). This is also observed by better test performance of serology tests (both POC and non-POC) in symptomatic patients as compared to some studies with asymptomatic patients in our review. While there is ample evidence of similar viral load between asymptomatic and symptomatic patients, significantly faster viral clearance was observed in the former (57, 58). Higher viremia causes lung damage that can be observed from radiography. Hence, a rapid turnover of the virus within the body may result in poorer performance of NAAT and imaging methods that rely on the detection of viral genomic material and lung manifestation. Although it is intuitive to assume a lack of viremia-induced lung inflammation in the absence of symptoms, Hu et al. identified CT abnormality in 71% of asymptomatic patients (59) while 54% of the asymptomatic patients in the Princess Diamond cruise ship had lung opacity in their chest CT (60). On the other hand, Salvatore et al. had identified that Ct values vary with the presence of symptoms. Individuals with no symptoms at the time of sample collection had higher Ct values as compared to those reporting any symptoms (median Ct values 33.3 vs. 29.3) (61). Thus, holding all other factors constant, a higher Ct cut-off value increases the potential for false positives. Likewise, a lower Ct cut-off value may be more prone to false negatives, particularly in asymptomatic patients.

### Across Different Geographical Locations

In a 2014 study, Vivaldo et al. discussed differences in sensitivities of diagnostic tests due to the epidemiological evolution of DENV serotypes (62). Similarly, we draw attention to the influence of SARS-CoV-2 distribution across geographical regions on diagnostic test accuracy. As of August 2020, there are at least 6 strains of coronavirus predominating in different geographical regions (63). A genome-wide analysis found a higher frequency of amino acid mutation in Europe, followed by Asia and North America (64). Potential mismatch between diagnostic RT-PCR assays and SARS-CoV-2 genome caused by mutations can result in false negatives by deterring primer-binding and amplification (65). Hence, alternative diagnostic tests, e.g., serology tests may complement this potential pitfall of PCR primers being insufficiently accurate for detection in the presence of evolving variants. In this review, we could not infer significant differences between the geographical origins of the samples and the sensitivity of amplification tests. This suggests the continued relevance of current diagnostic tests in detecting SARS-CoV-2 infections globally. A reason behind this could be the inherent stability of coronaviruses, emphasized by genome data based on more than 90,000 SARS-CoV-2 isolates (66). Alternatively, the advantageous use of two gene targets for diagnosis could have secured detection even if one target region has mutated sufficiently. We could not discern distinct differences in the performance of serology assays and imaging on samples from various geographical origins. As meta-analysis comprising only two studies was considered insufficiently robust, performance estimates of serology (non-POC) and imaging tests used outside China were largely excluded from the comparison. It is worthy of our attention as different strains could be characterized by distinct pathogenicity, which induce different levels of lung and clinical manifestations picked up by imaging methods. Altered immunogenicity in the various strains could translate to differential immune response, affecting serology assay performance and their effective time period. In line with our review finding, there is no literature reporting differential immunogenicity or pathogenicity of the strains to the knowledge of the authors at the point of writing. Alternatively, pre-test probabilities varying across geographical regions due to differing local prevalence may have influenced the false-positive rates, and thus the accuracy estimates (67).

### Gene Target

Evidence backing the performance of gene targets is largely conflicting. The WHO established E gene and RdRp gene assays as first-line screening and confirmatory assays and recommended the less sensitive N gene assay as an additional confirmatory assay (68). Conversely, Chu et al. suggested the use of E and N assay as screening assays, and RdRp and Orf1b as confirmation assays after identifying the N gene to have better sensitivity than Orf1b (69). Others have reported that only the RdRp gene is almost specific for SARS-CoV-2. Interestingly, a preprint study by Loying et al. reported that the N gene persisted significantly longer [mean 12.68 days (S.D. ± 3.24)] than the OFR1ab gene [mean 12.09 days (S.D. ± 2.88)] in their study of 46 patients (70). This review observed high sensitivity and specificity across N, S, ORF1ab, RdRp, E, and Nsp2 genes, contrary to inconsistent literature on gene target performance. The strength of this observation lies in the greater number of clinical samples analyzed. Nonetheless, assays should include at least two gene targets to avoid possible cross-reaction with other endemic coronavirus or the occurrence of genetic drift of SARS-CoV-2 (71). The inclusion of a conserved and a specific region can reduce the possibility of false negatives as SARS-Cov-2 may evolve in a new population (71). Difference in the persistence of gene positivity can also come to play a crucial role in COVID-19 diagnostics.

### Specimens Type

Our subgroup analysis on different specimen types used in amplification tests found nasopharyngeal specimens as the most sensitive. Most samples were from the upper respiratory tract, albeit of unspecified location. We were unable to perform a comparative analysis between upper and lower respiratory tract specimens due to the paucity in studies utilizing lower respiratory tract specimens. The specimen collection site is important for the successful diagnosis of infection, given the reliance on amplification tests by health authorities in the community spread prevention and border reopening efforts (30).

While studies have identified lower respiratory samples to be more sensitive than upper respiratory tract samples (72, 73), their use is limited by safety concerns and technical challenges in sample collection (72). A review by Bwire et al. showed a moderate positive rate of 45.5% in nasopharyngeal specimens and a low positive rate of 7.6% in oropharyngeal specimens (74). This review only included a single study by Chan et al. investigating the use of saliva specimens (17). More recent studies have established saliva as a useful alternative sample type. Yokota et. al. reported NAAT sensitivity of 92% (90%CI: 83–97%) using saliva as compared to 86% (90%CI: 77–93%) using nasopharyngeal swabs during mass screening (23). The sensitivity of saliva samples was at least on par with nasopharyngeal samples, as concluded by Wyllie et. al. in a separate study, and positive detection in asymptomatic healthcare workers was also higher using saliva samples (75). Overall, saliva potentially minimized false positives, with better detection performance and lesser false negatives as compared to nasopharyngeal, oropharyngeal, and sputum samples in populations with low viral load (76). Self-collection of saliva samples by patients can further mitigate the risk of exposure faced by healthcare workers, alleviating demands for swabs and personal protective equipment (23).

In this review, Chan et al. and Yu et al. did not detect the virus in the urine of patients with COVID-19, suggesting that urine is inappropriate for COVID-19 testing. This is corroborated by Bwire et al.'s lack of virus detection in urine, low detection in blood, and moderate detection in serum (74). However, viral presence in the urine could depend on disease severity as Nomoto et al. detected the virus in urine samples of a moderate and a severe patient (77). A study by Zhang et al., established body fluids and excretions as viral shedding routes (78). However, virus detection from various sample sites due to different viral shedding pathways may not translate to sufficient sensitivity for diagnosis. The single study by Chan et al. using stool specimens in this review showed high sensitivity (17). Separately, Chen et al. detected SARS-CoV-2 in the stool of 67% of COVID-19 patients and concluded that viral presence was not associated with disease severity as positivity in stool specimens persisted in most samples even after pharyngeal swabs became negative (79). On that note, the WHO recommends the use of stool specimens from the second week of symptom onset when there is clinical suspicion of SARS-Cov-2 despite negative respiratory specimens (80).

### Limitation

This review is constrained by the following limitations. Firstly, not all controls have received the reference test. At least 62.7% of the controls in the included studies and at least 66.4% of the controls included in the meta-analysis had received the reference test. Controls who were not tested or unclear were mostly constituted of samples obtained before the COVID-19 period, positive for other viruses or from healthy volunteers and blood donors at medical institutions. These subjects could have been misclassified as controls, resulting in lowered specificity. However, since pooled specificity in this review remains relatively high for most tests, there may be a minimal likelihood of misclassification. The second possible limitation is the interval between the novel and the reference test. Reference tests in some studies were conducted upon hospital admission, whilst novel tests may be conducted with new samples collected at a later time point. This may affect the time-sensitivity of the results, especially if the novel test in question is a genome amplification test (81). On the other hand, re-testing old samples run the risk of false-negative if specimen degradation occurred under improper transport or storage conditions (82). Thirdly, this review accepted either PCR or sequencing as reference tests, which can potentially cause misclassification. While we accepted two types of reference tests, sensitivity analysis was not performed since only three included studies utilized sequencing as the reference test and, hence, unlikely to yield a meaningful comparison. Fourthly, the predominant use of case-control study design by most included studies plausibly inflates both sensitivity and specificity estimates by the spectrum bias (83). It is worthy to note that most cases were consecutively recruited patients at medical institutions despite controls usually being historic, stored samples. Another potential gap was the lack of antigen test development, which could be another viable rapid testing tool. Since antigen tests detect viral proteins, the possibility of it picking up a case precedes serology testing, which detects antibodies developed as part of our immune response. Next, this review could not compare diagnostic test performance between different age groups due to a lack of studies involving children. There is a gap in the existing literature that is skewed toward COVID-19 in adults. Studies on children are limited possibly due to testing practices that prioritizes symptomatic patients, health care workers, and institutionalized seniors. As children tend to have milder symptoms, they are less likely to be tested and diagnosed (84). Yet, there is no substantial evidence of differences in the viral load and persistence of virus detection over time between adults and children. The inclusion of children may shed light on different diagnostic test performances in a normal population setting. This review could not do a subgroup analysis of test performance comparing immunocompromised and healthy individuals. Burbelo et al. concluded in their preprint that immunocompromised individuals generally have a delayed antibody response compared to healthy individuals (85). More concrete evidence comes from Zhao et al., who postulated that early incomplete clearance of SARS-CoV-2 virus caused repeated negative RT-PCR tests and delayed antibody response in a patient with COVID-19 with HIV-1 and Hepatitis C coinfection (86). The selection of diagnostic tests for this group of patients ought to consider these differences in immune response in addition to the time point of testing from disease onset. This review was only able to compare the different timing from onset to the sample collection for the serology test including POC as there were limited studies for the other diagnostic tests. Lastly, only studies published up to April 30, 2020 were included in this review, and thus, only reflected tests developed in the very early phase of the pandemic. Major developments in COVID-19 diagnostic testing occurred since the search for relevant studies in this review, to develop more accurate and efficient tests. Hence, tests presented in this review may not encompass all tests that have been commercialized or authorized for use, especially more current modalities such as breath test (87) and quantitative antigen test (88). Nonetheless, most of the newly developed or improved versions of existing tests are built on the same technology as those presented in this review, maintaining continued relevance of the comparison between molecular tests, serologic tests, and radiologic tests with or without incorporation of artificial intelligence made in this review. Findings presented in this review may be useful to aid policy makers in assessing the suitability of a test for border screening or rapid diagnosis during the very early stages in the case of a future pandemic—better understanding of the performance of each test type in early outbreak phases will allow quicker response to control virus spread.

## Conclusion

Nucleic acid amplification tests had the highest performance, among others. Amplifications tests should be employed as the reference standard test to detect SARS-CoV-2 infection whenever possible. Point-of-care NAAT and serology tests have high potential utility for border screening due to their ease of conduct and shorter turnaround time. However, it should be noted that the time point since symptom onset and severity of the patient at the point of testing will influence the performance. Serologic tests were more sensitive when testing is done in the later phase of infection. All diagnostic tests were more sensitive among symptomatic than asymptomatic individuals, which emphasizes the importance of quarantining at-risk individuals and mandatory post-quarantine testing during the early phase of the pandemic. As more countries are making pre-departure and post-arrival PCR testing mandatory, in addition to two or three PCR testing during the quarantine period, and potentially with the immunity “passport” requirement, it would be highly reasonable to deploy POC tests for border screening to alleviate the resource and time constraint for the increasing demand of laboratory tests as we progressively reopen the borders.

## Data Availability Statement

The original contributions presented in the study are included in the article/Supplementary Material, further inquiries can be directed to the corresponding authors.

## Author Contributions

JP conceptualized the study, supervised screening progress, validated the final screened studies and analysis, and critically reviewed the manuscript. PC, MW, and SG drafted the manuscript, screened the studies, and extracted data. PC and SG analyzed data and prepared the figures and tables. All authors have read and agreed to the published version of the manuscript.

## Funding

This research received funding from the Ministry of Defense (N-608-000-065-001).

## Conflict of Interest

The authors declare that the research was conducted in the absence of any commercial or financial relationships that could be construed as a potential conflict of interest.

## Publisher's Note

All claims expressed in this article are solely those of the authors and do not necessarily represent those of their affiliated organizations, or those of the publisher, the editors and the reviewers. Any product that may be evaluated in this article, or claim that may be made by its manufacturer, is not guaranteed or endorsed by the publisher.
