# Supplementary material for: Severe Acute Respiratory Syndrome Coronavirus 2 Diagnostic Tests for Border Screening During the Very Early Phase of Coronavirus Disease 2019 Pandemic: A Systematic Review and Meta-Analysis
Source: Front Med (Lausanne). 2022 Feb 14;9:748522. doi: 10.3389/fmed.2022.748522 (PMC8882616; doi:10.3389/fmed.2022.748522)
Supplement: Supplementary file 1 [file Data_Sheet_1.docx]

# **Supplementary**

# **Figures**

# **Figure S1: Diagnostic Tests Forest Plot on Sensitivity and Specificity, SROC and Diagnostic OR for 1. NAAT, 2. NAAT (POC), 3. Sequencing, 4. Serology (IgG and/or IgM), 5. Serology (IgG and IgM), 6. Serology (IgG), 7. Serology (IgM), 8. Serology (Ab), 9. Serology (IgA), 10. Serology (POC, IgG and/or IgM), 11. Serology (POC, IgG and IgM), 12. Serology (POC, IgG), 13. Serology (POC, IgM), 14. Imaging, 15. Imaging (AI) and 16. Clinical Features and Laboratory Parameters Model**

## NAAT


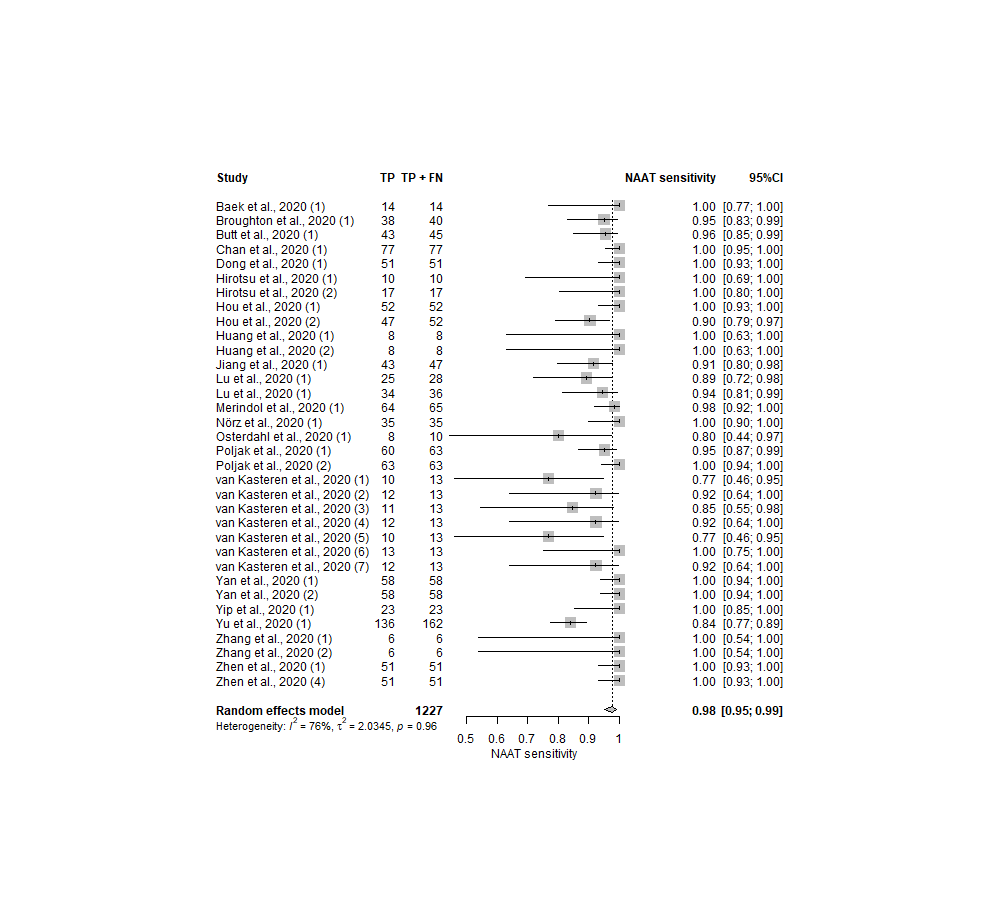

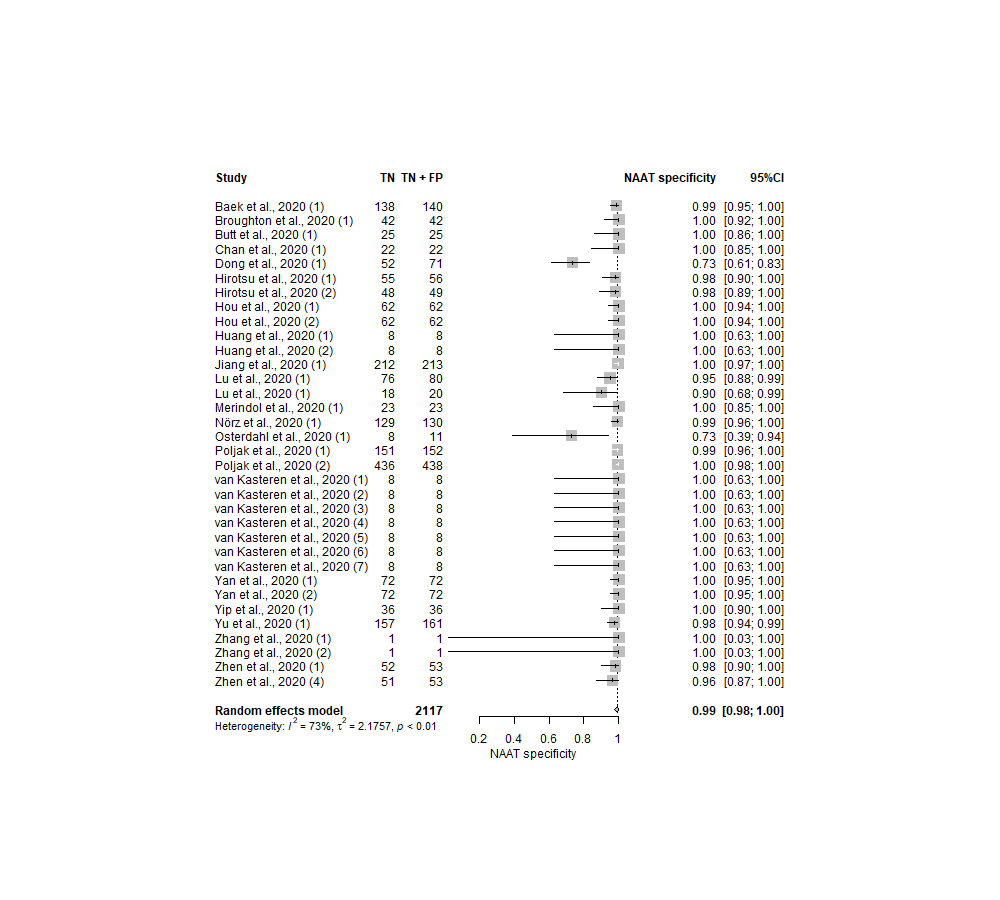


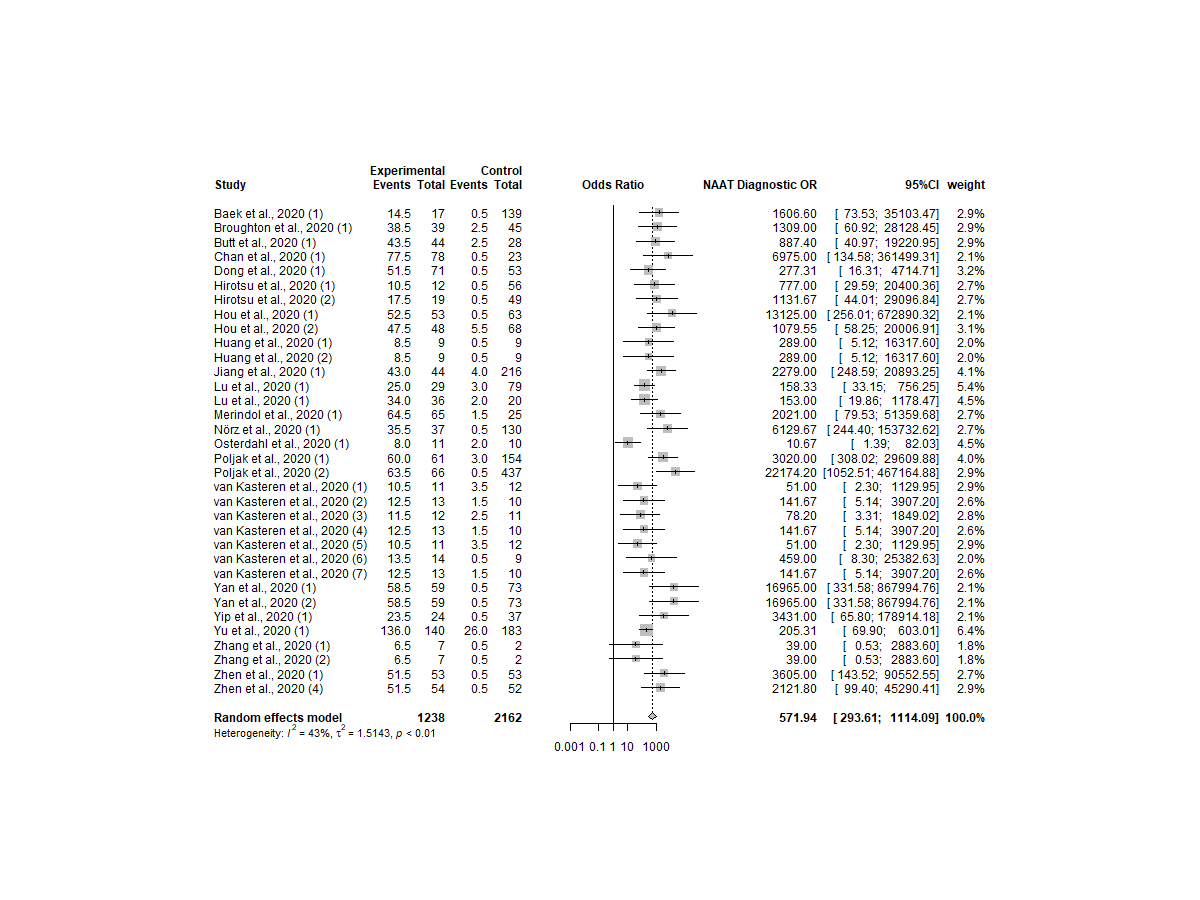

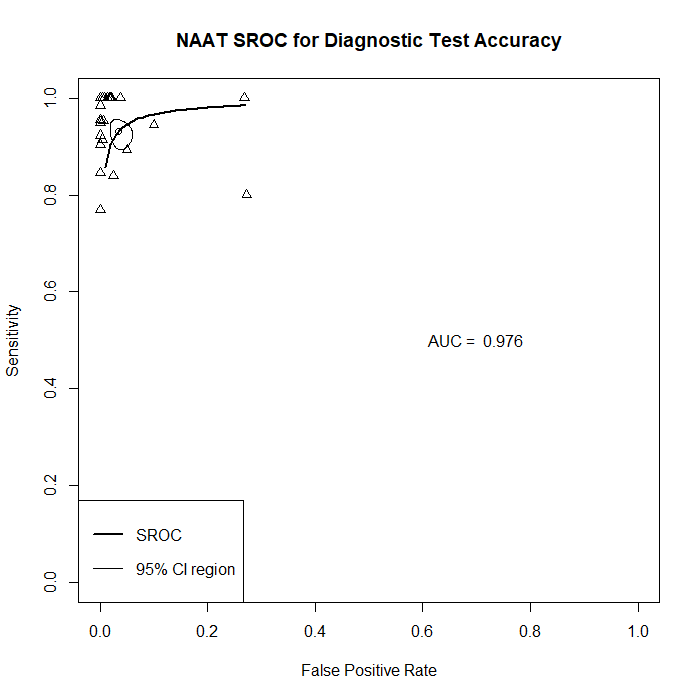


## NAAT (POC)
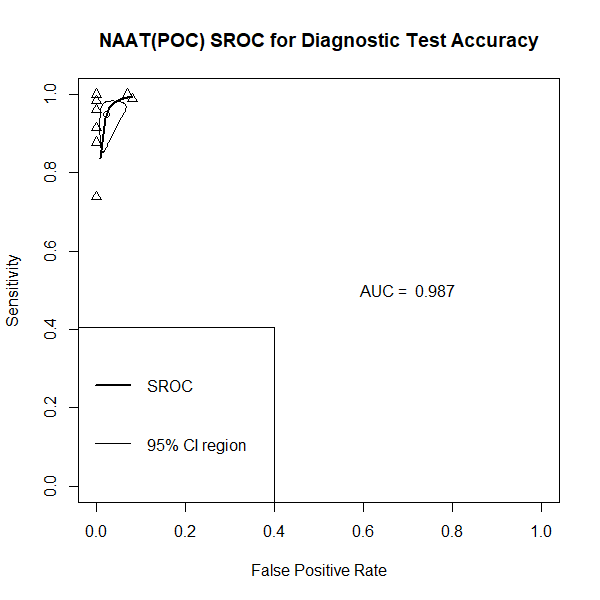


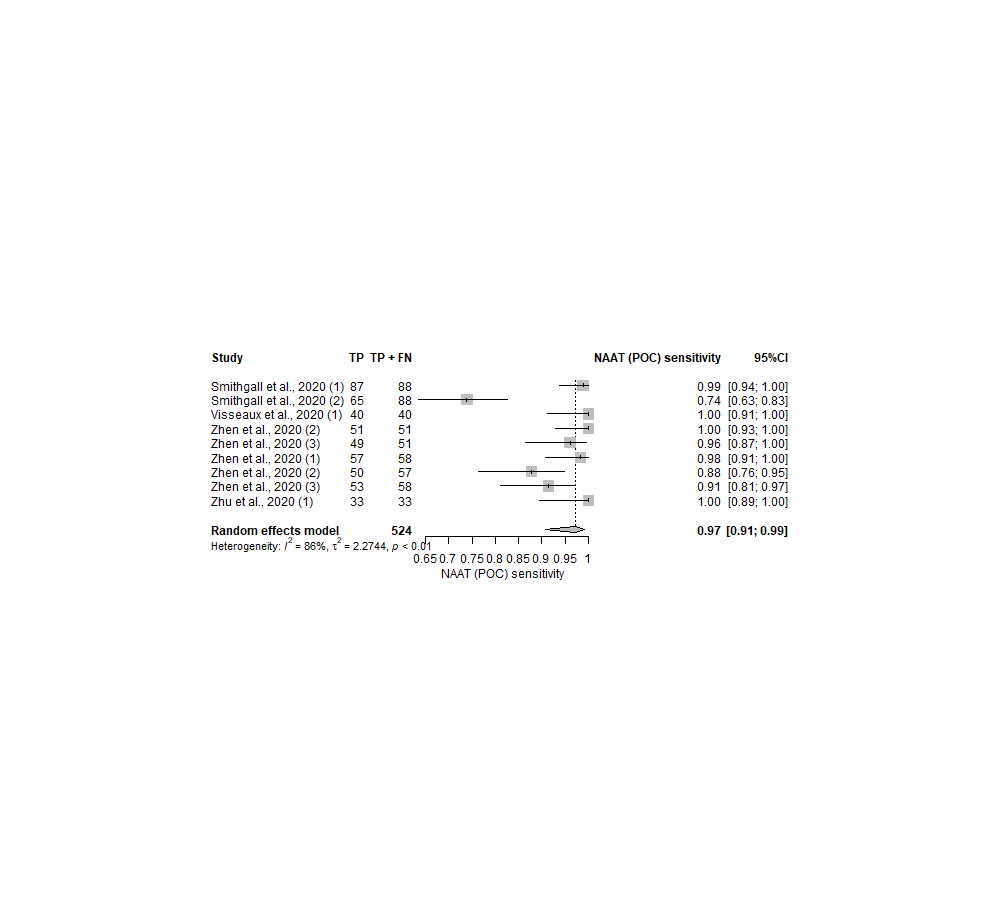


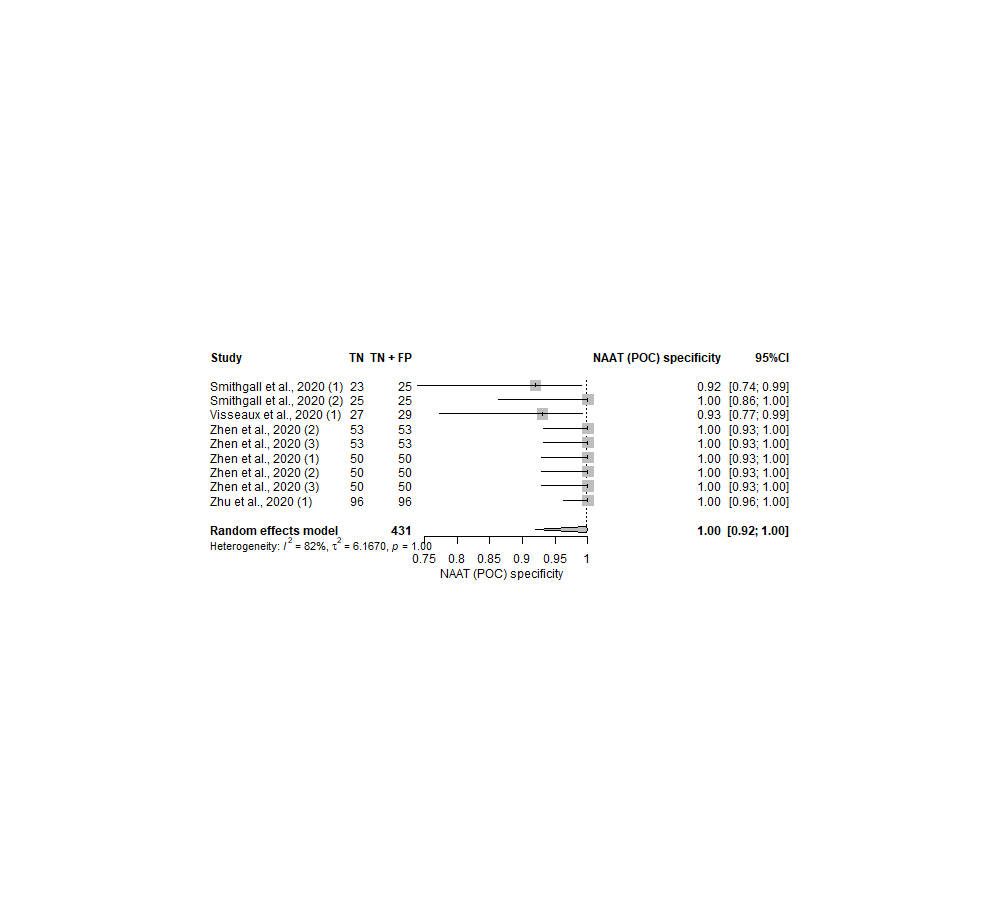


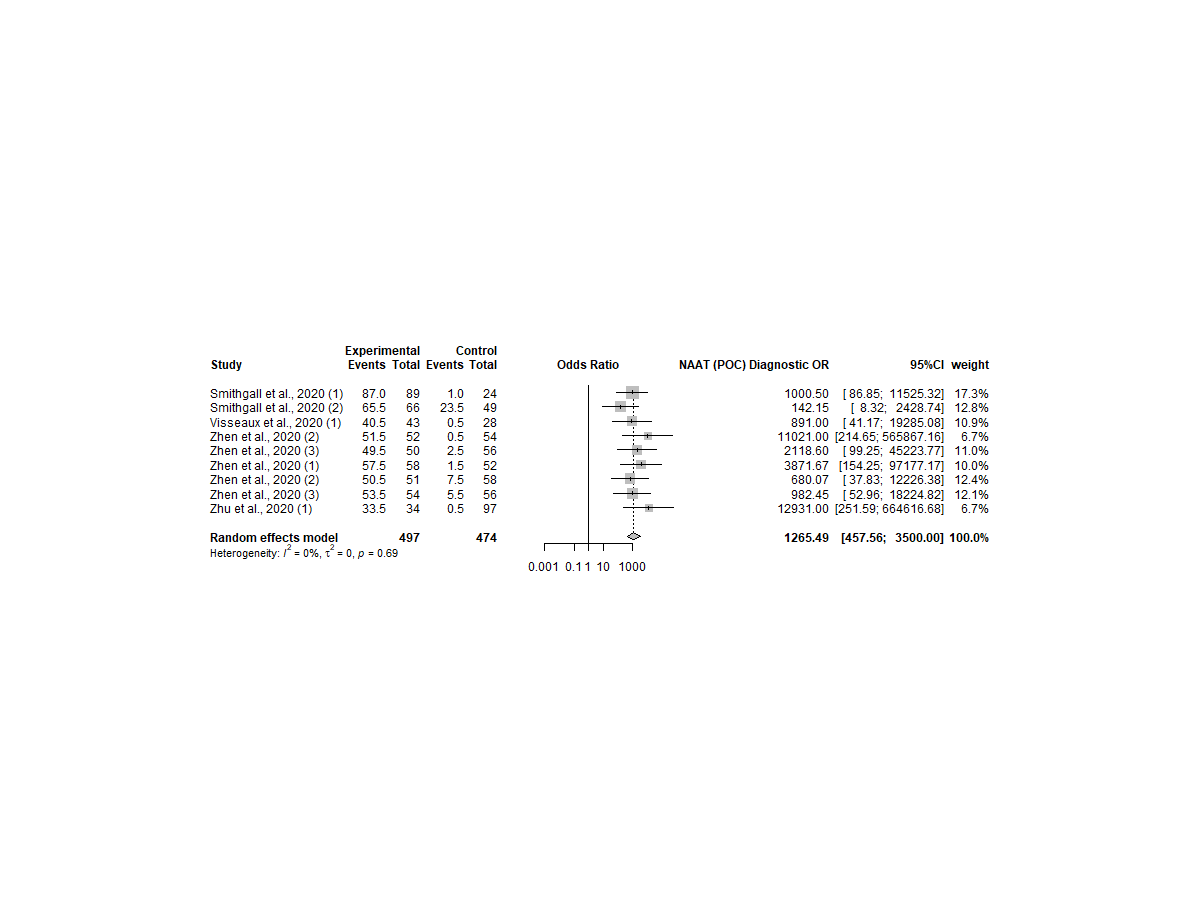


##
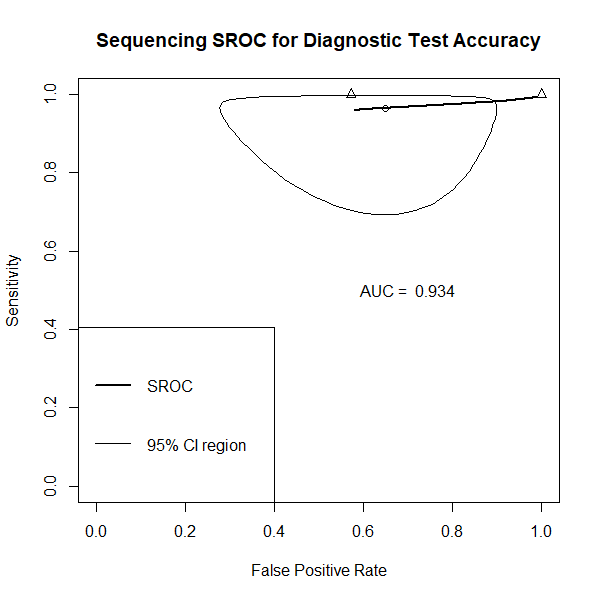
Sequencing


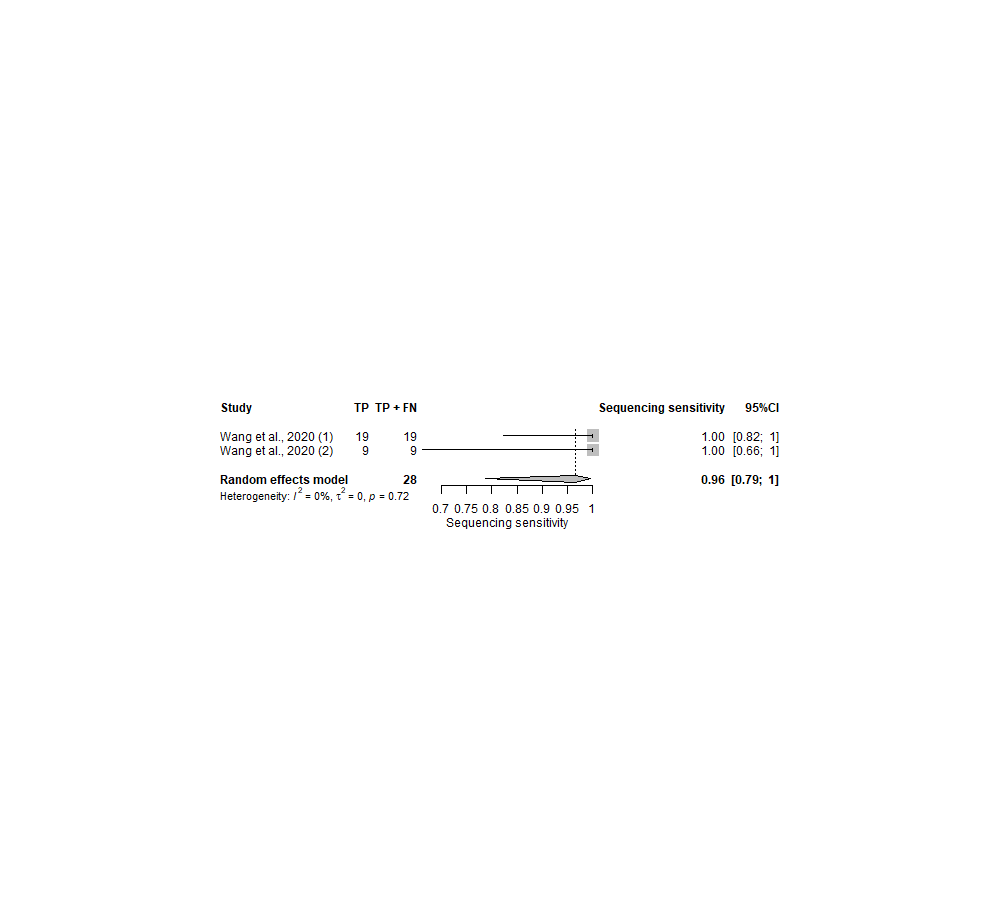


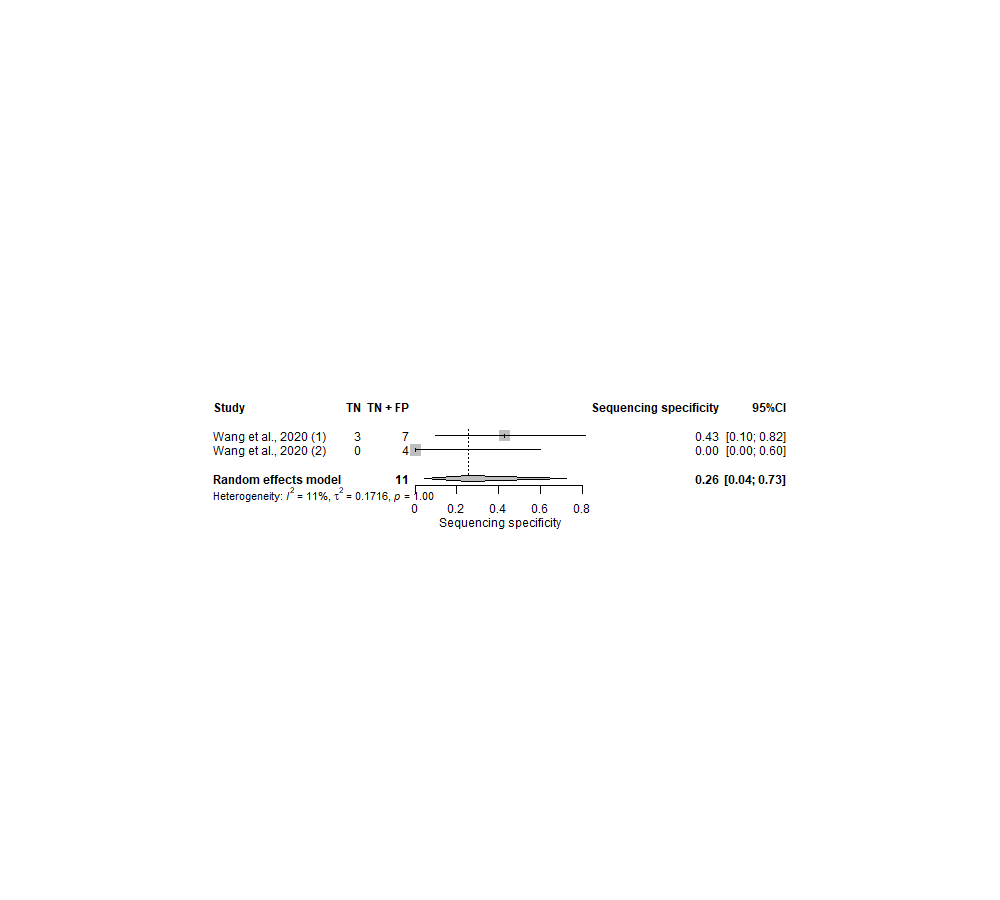


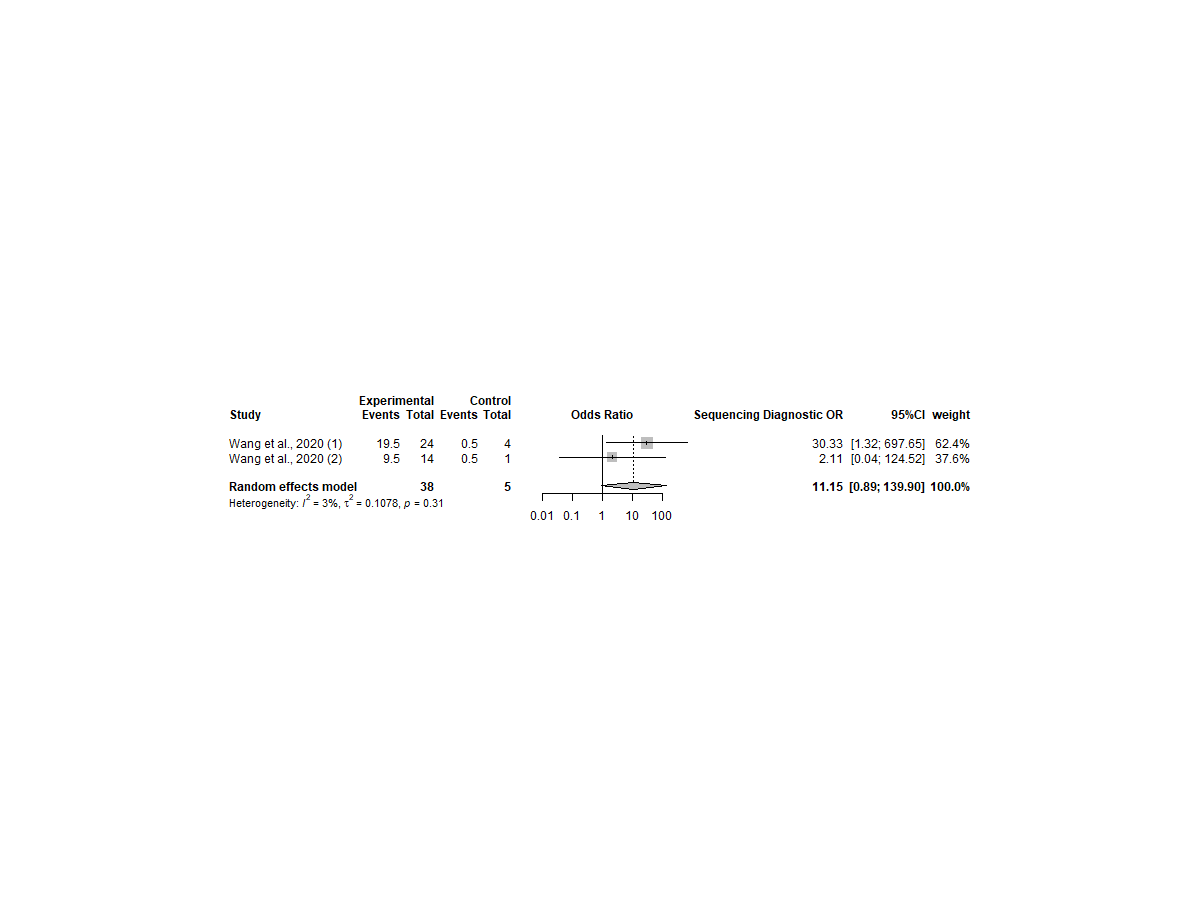


## Serology (IgG and/or IgM)


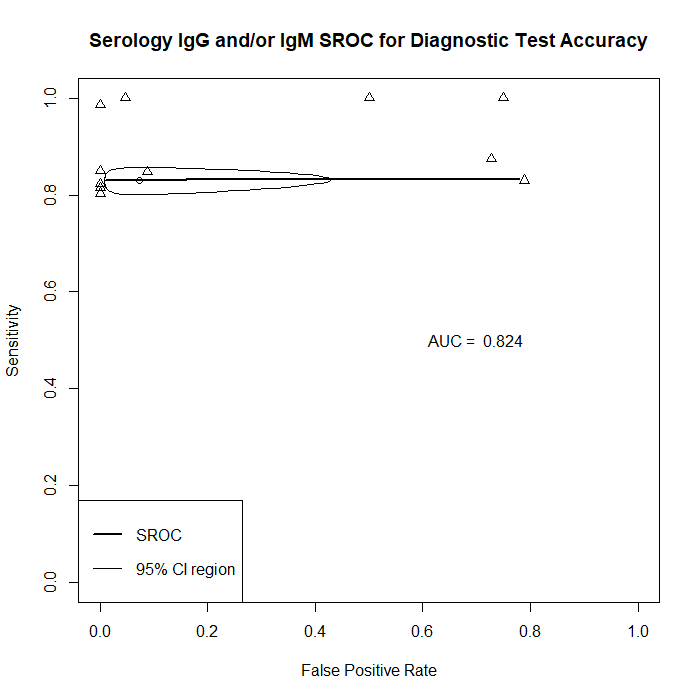

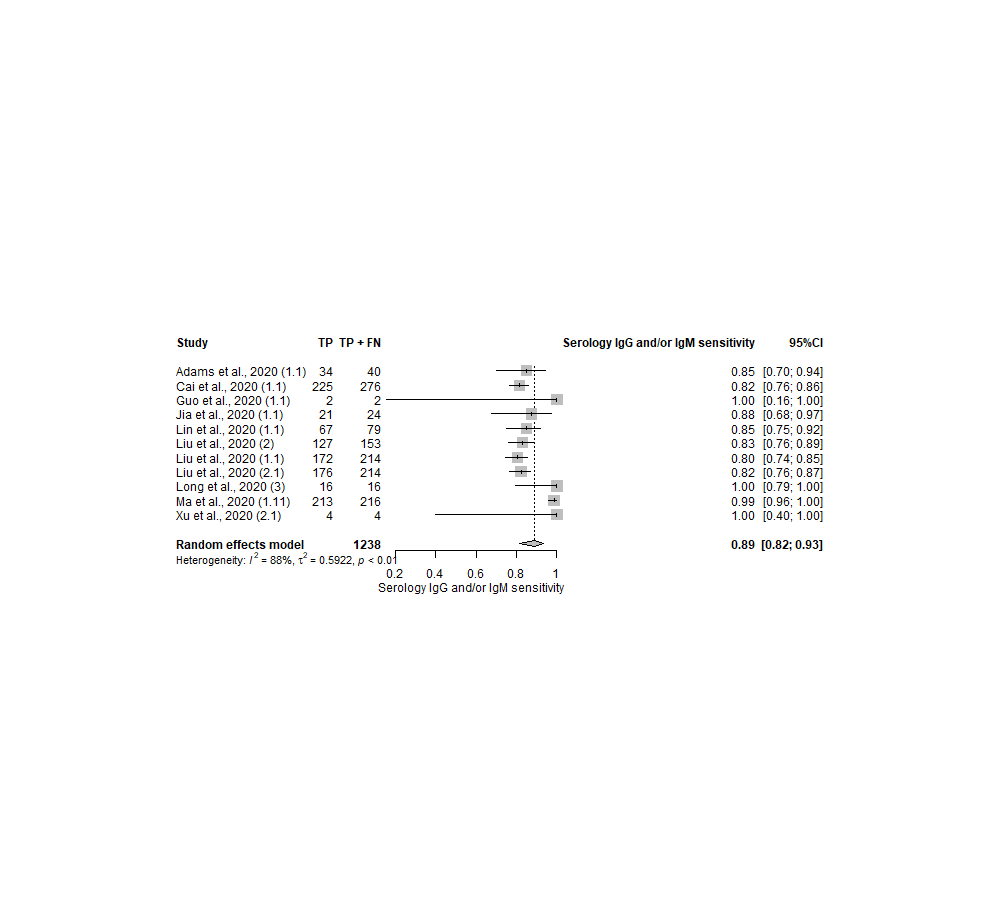


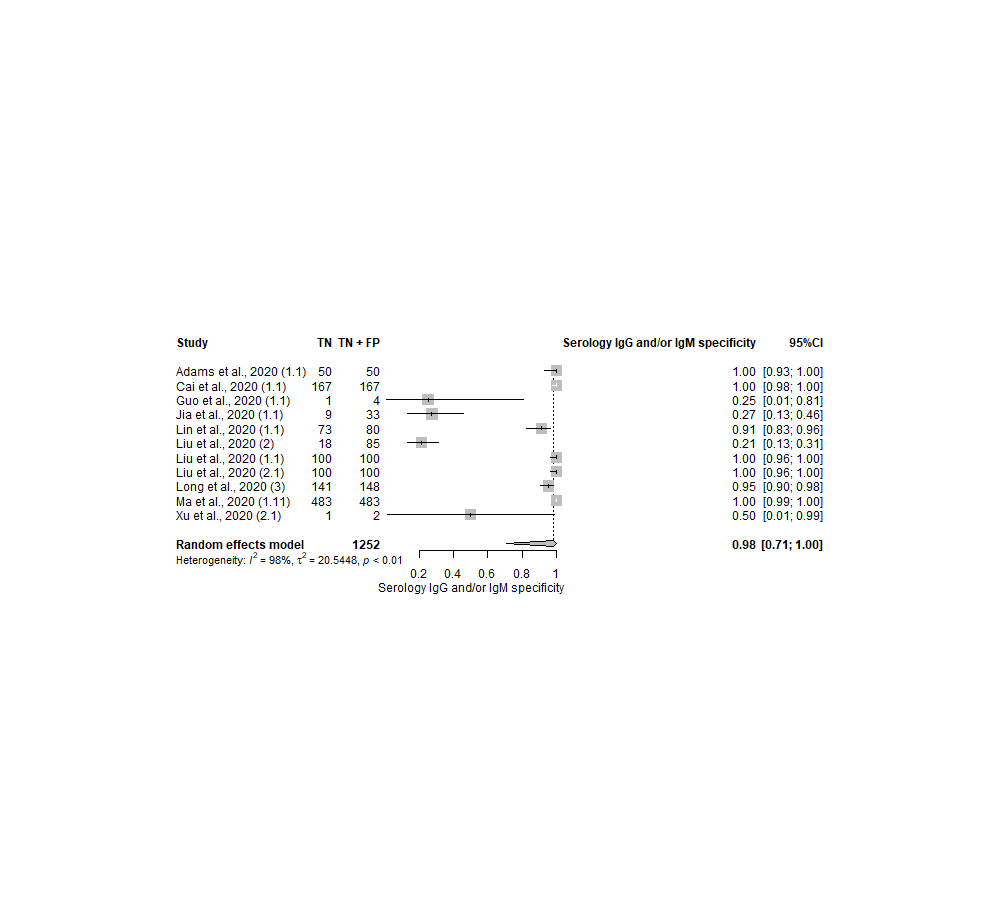


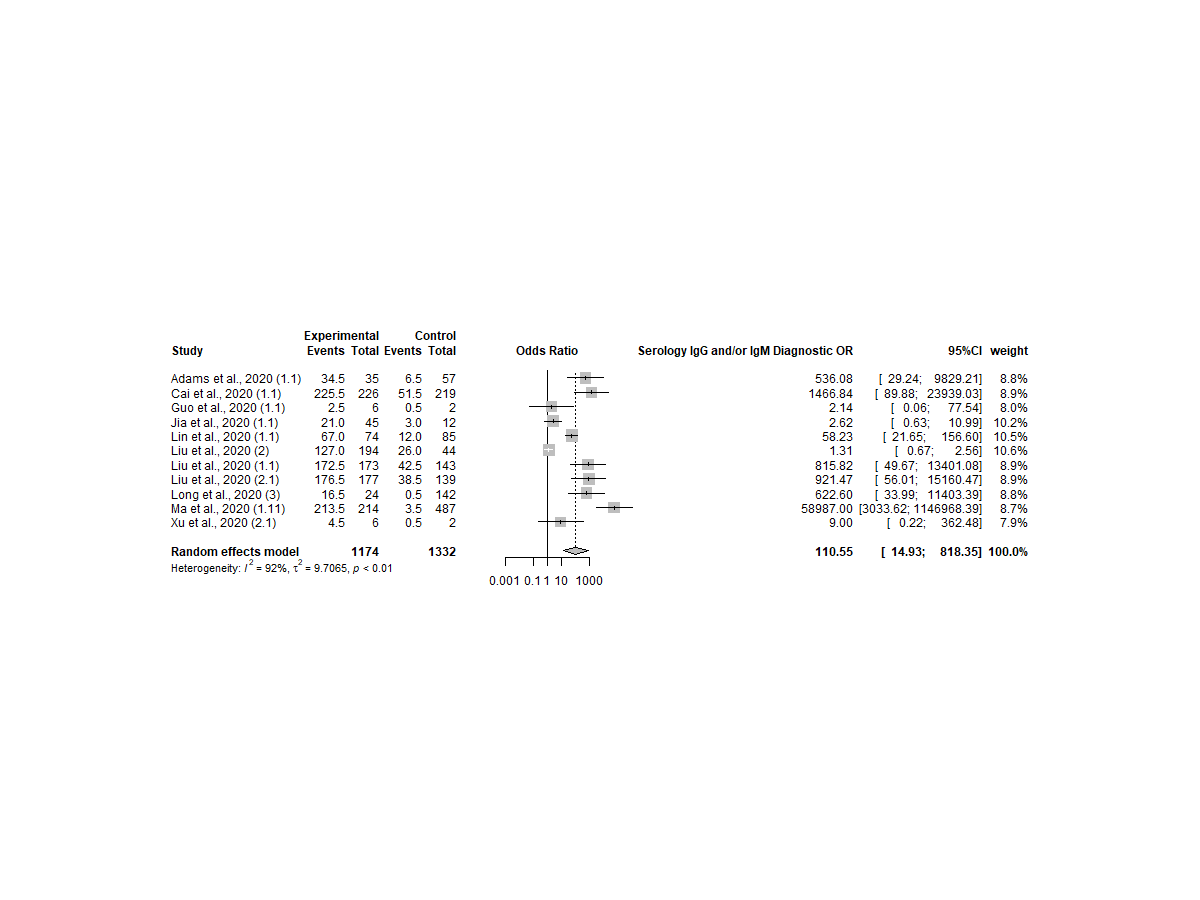


##
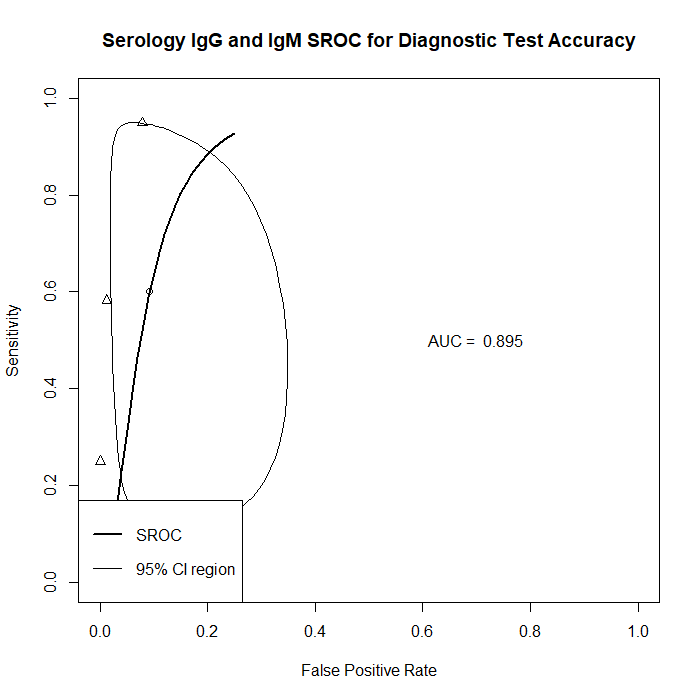
Serology (IgG and IgM)


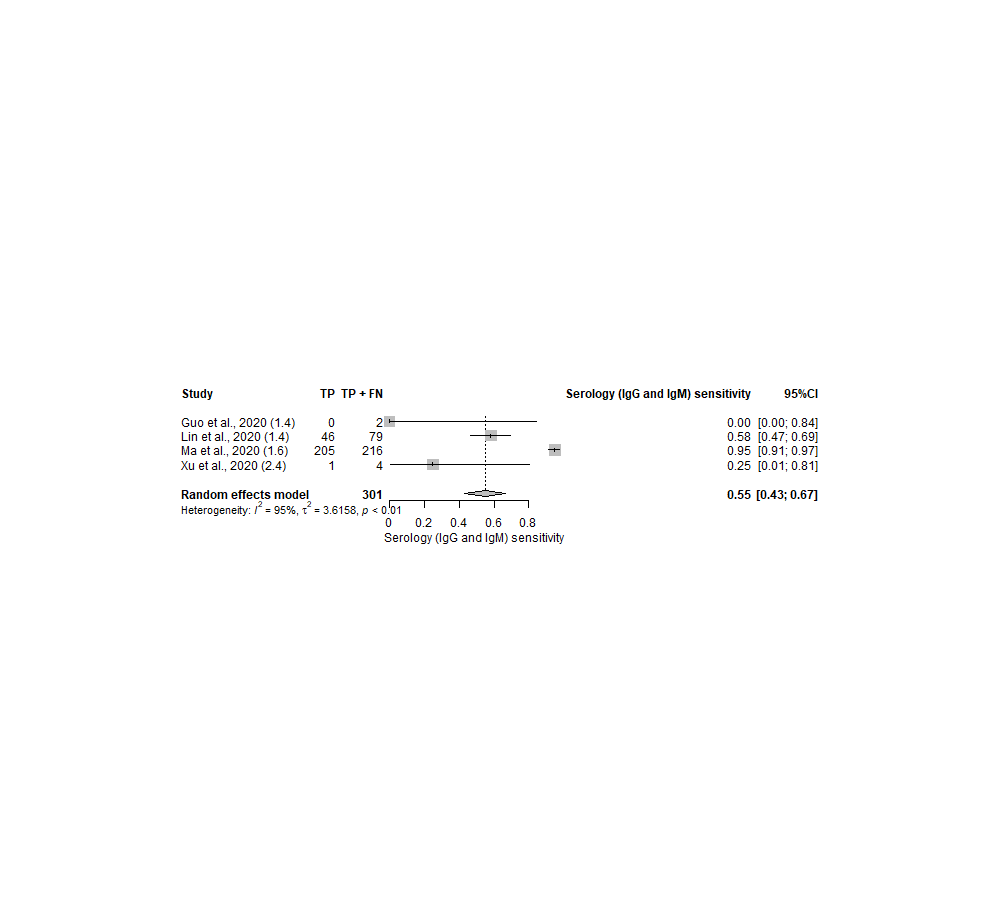


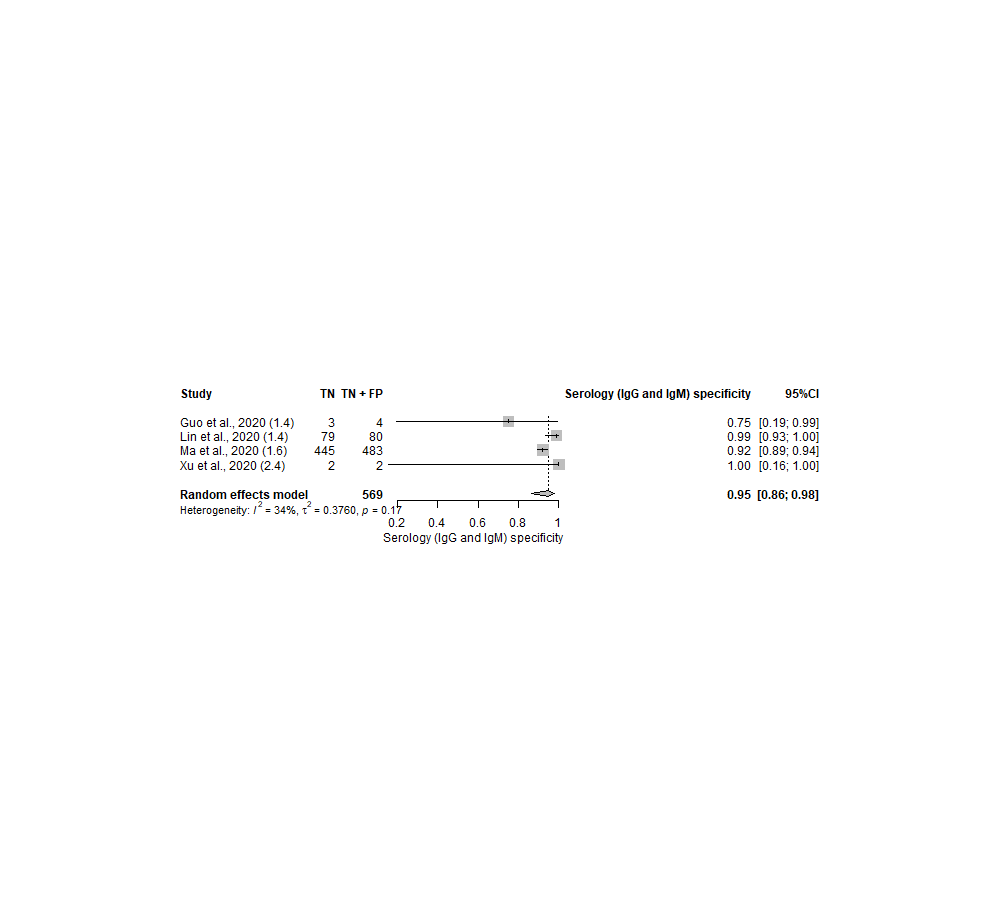


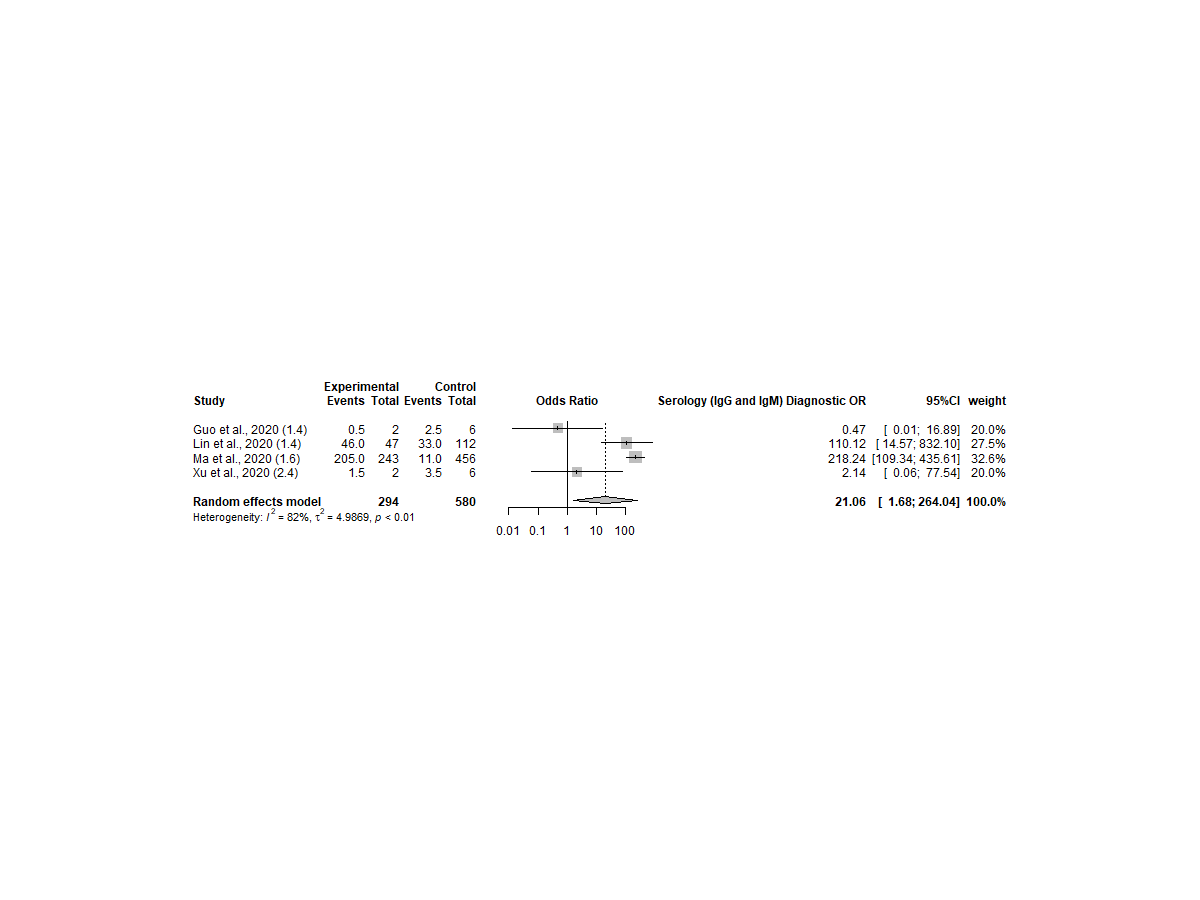


## 6. Serology (IgG)


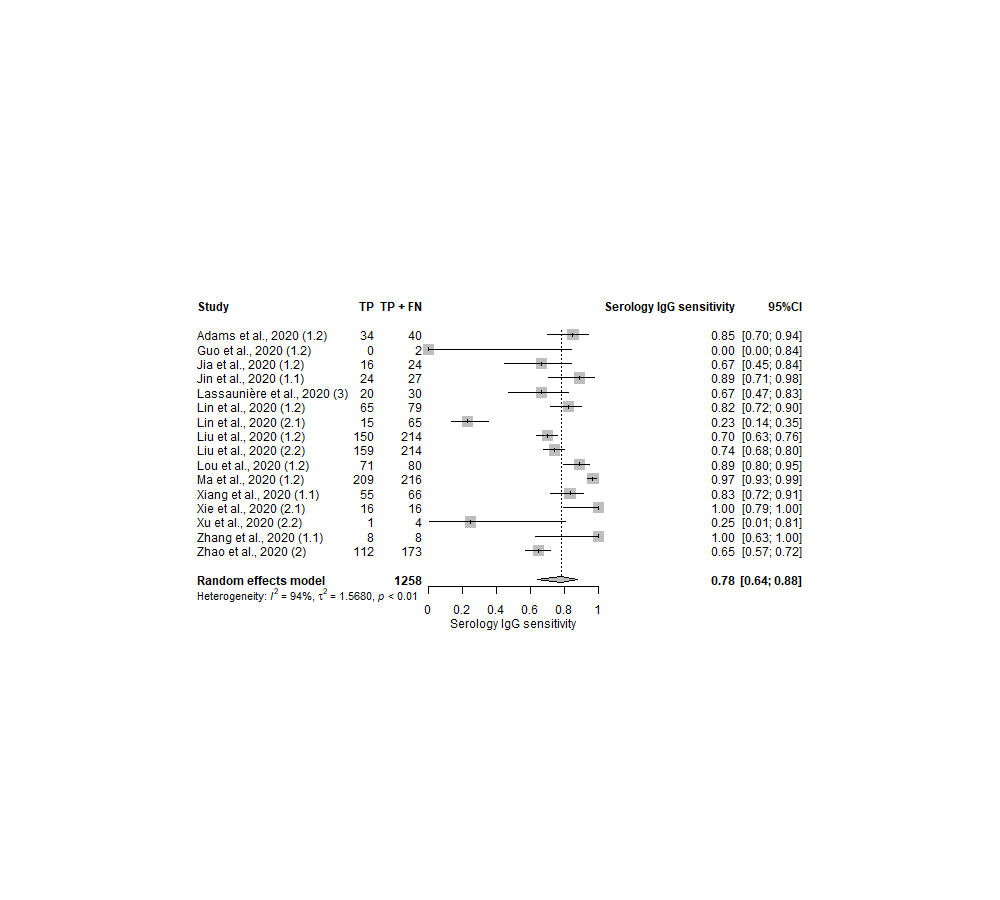

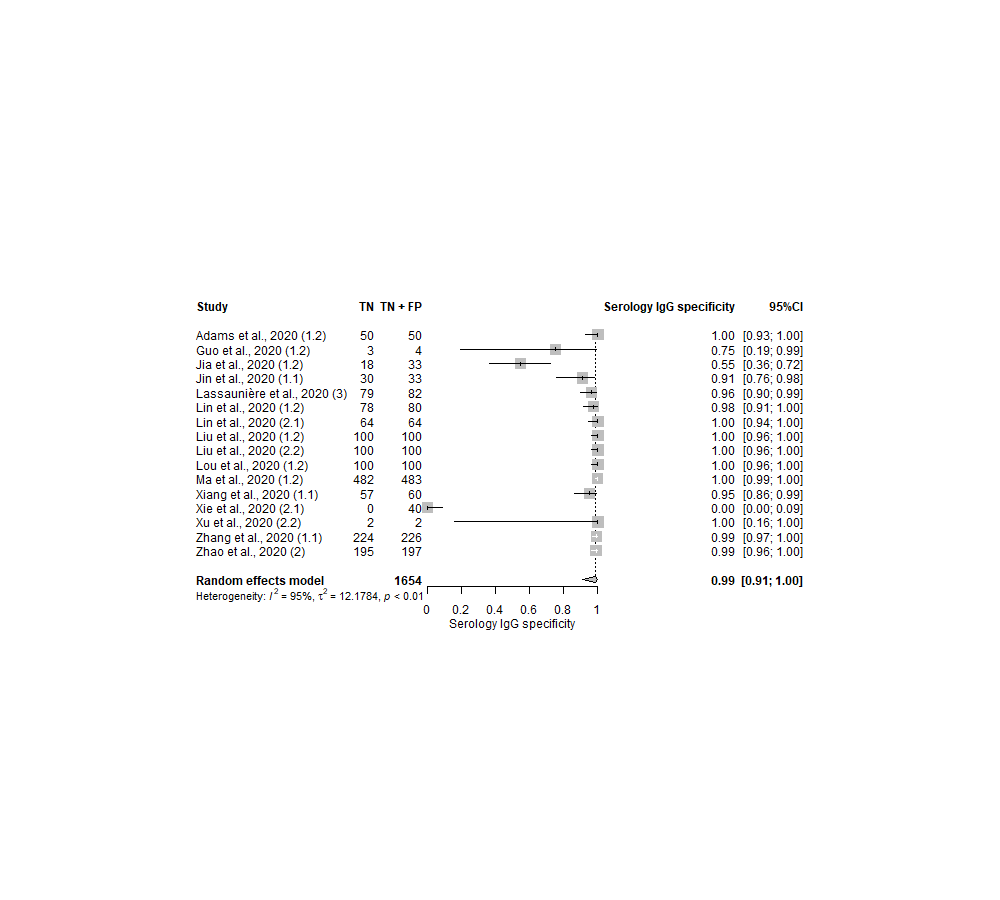


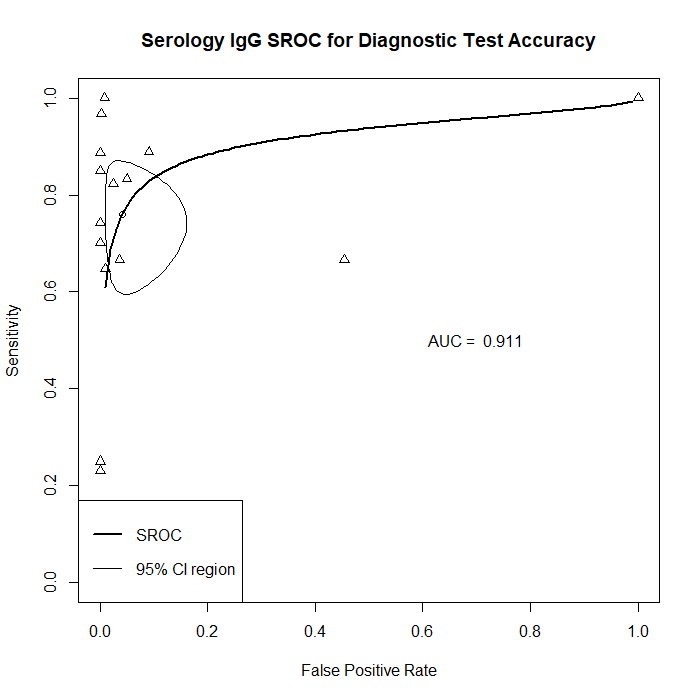

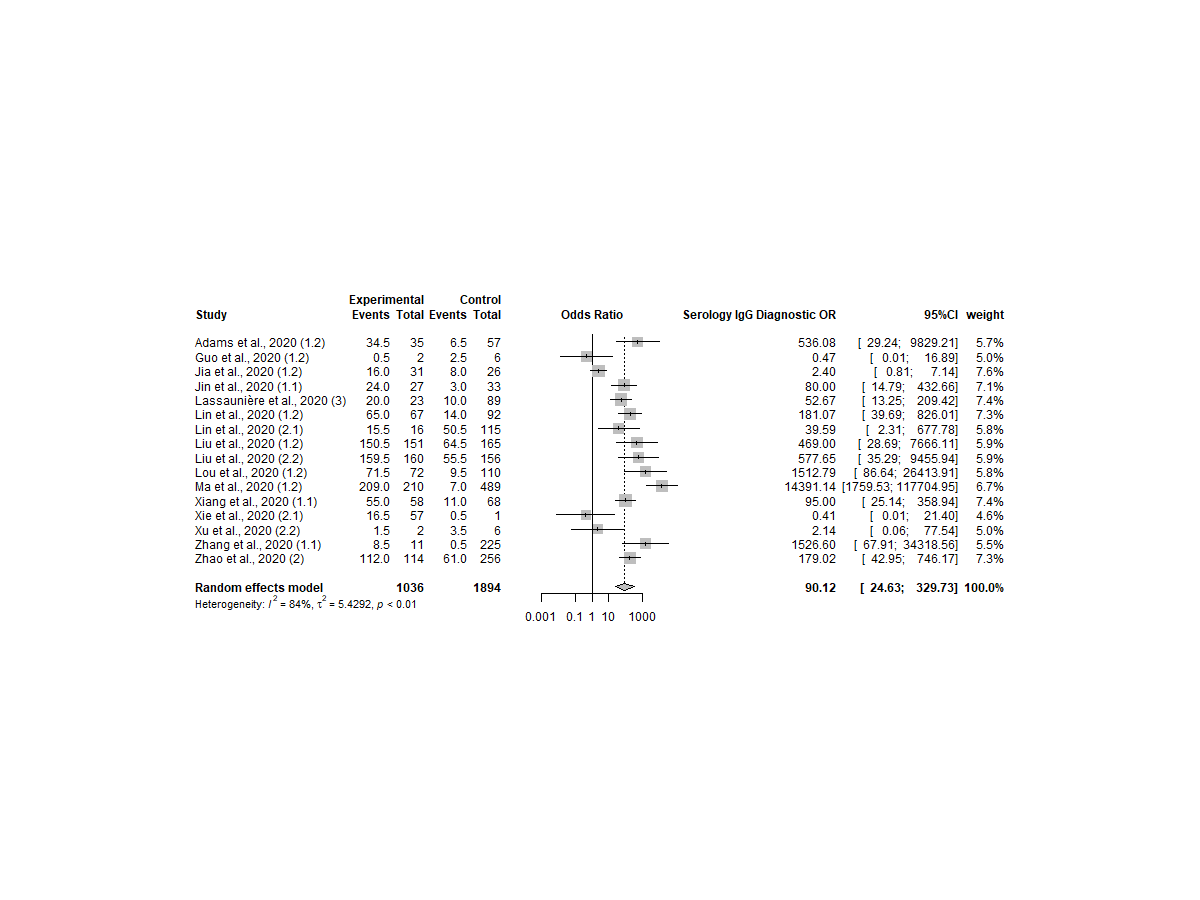


## 7. Serology (IgM)


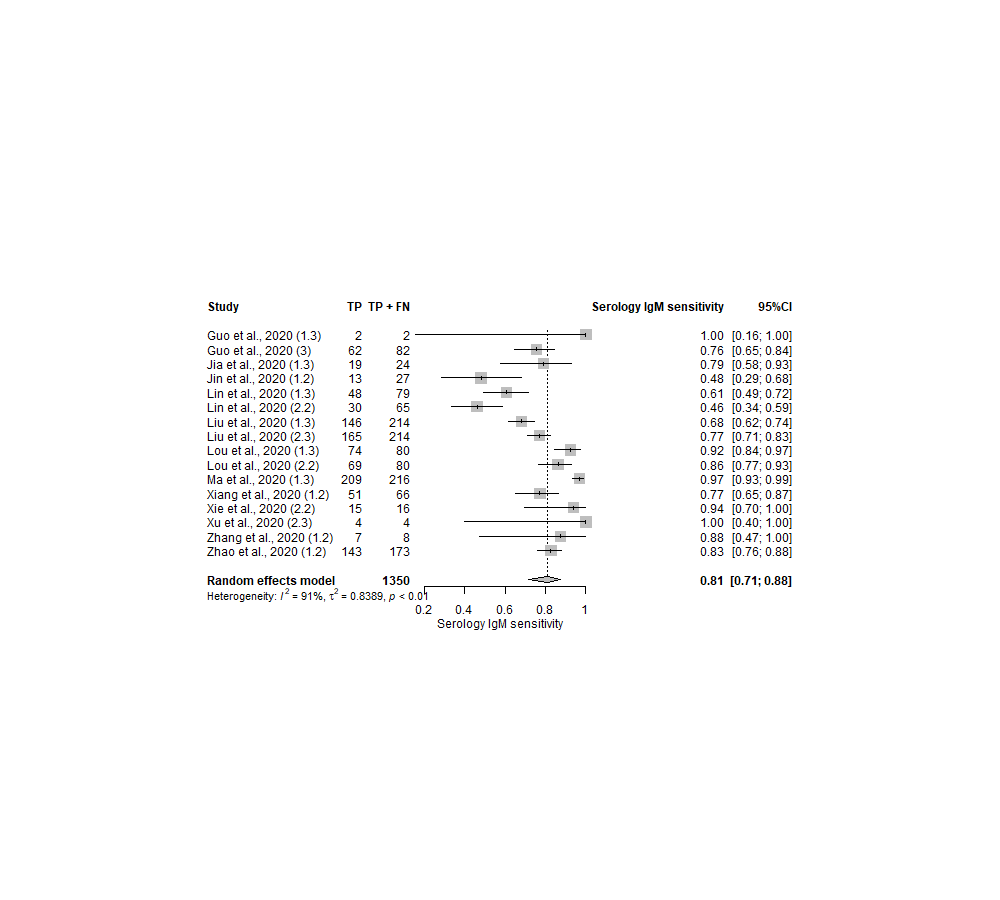

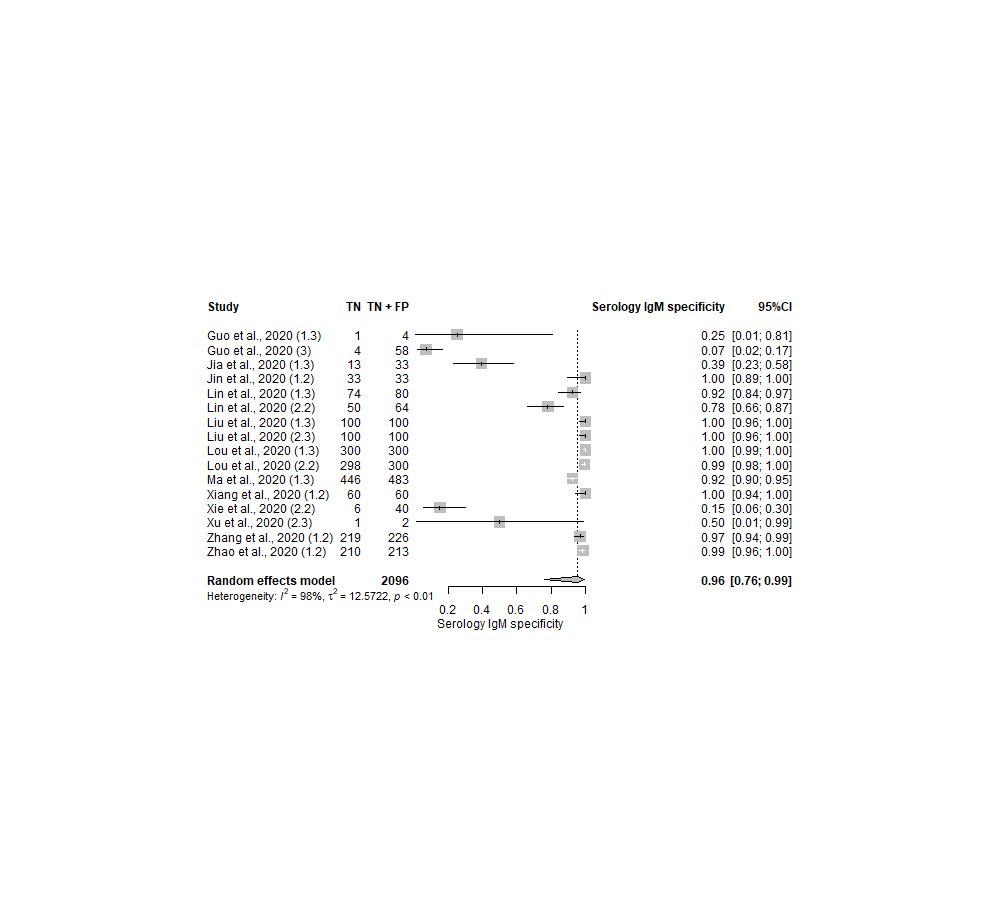


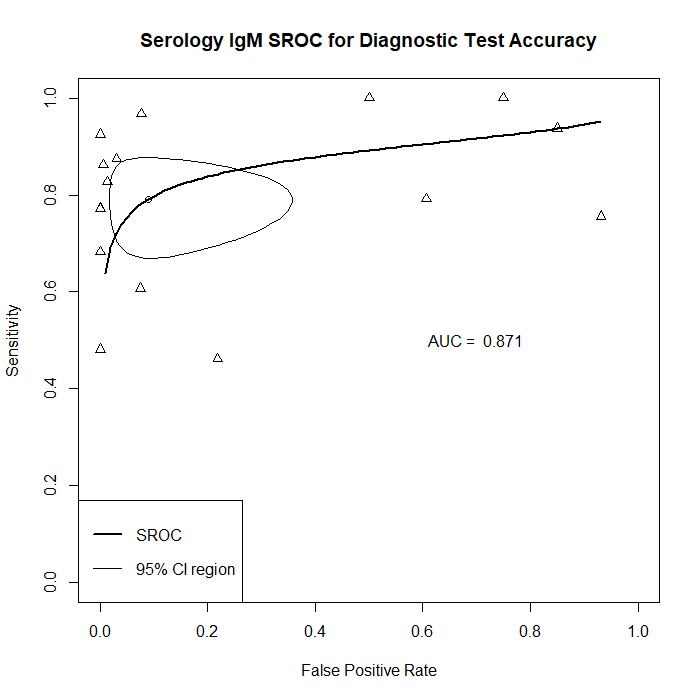

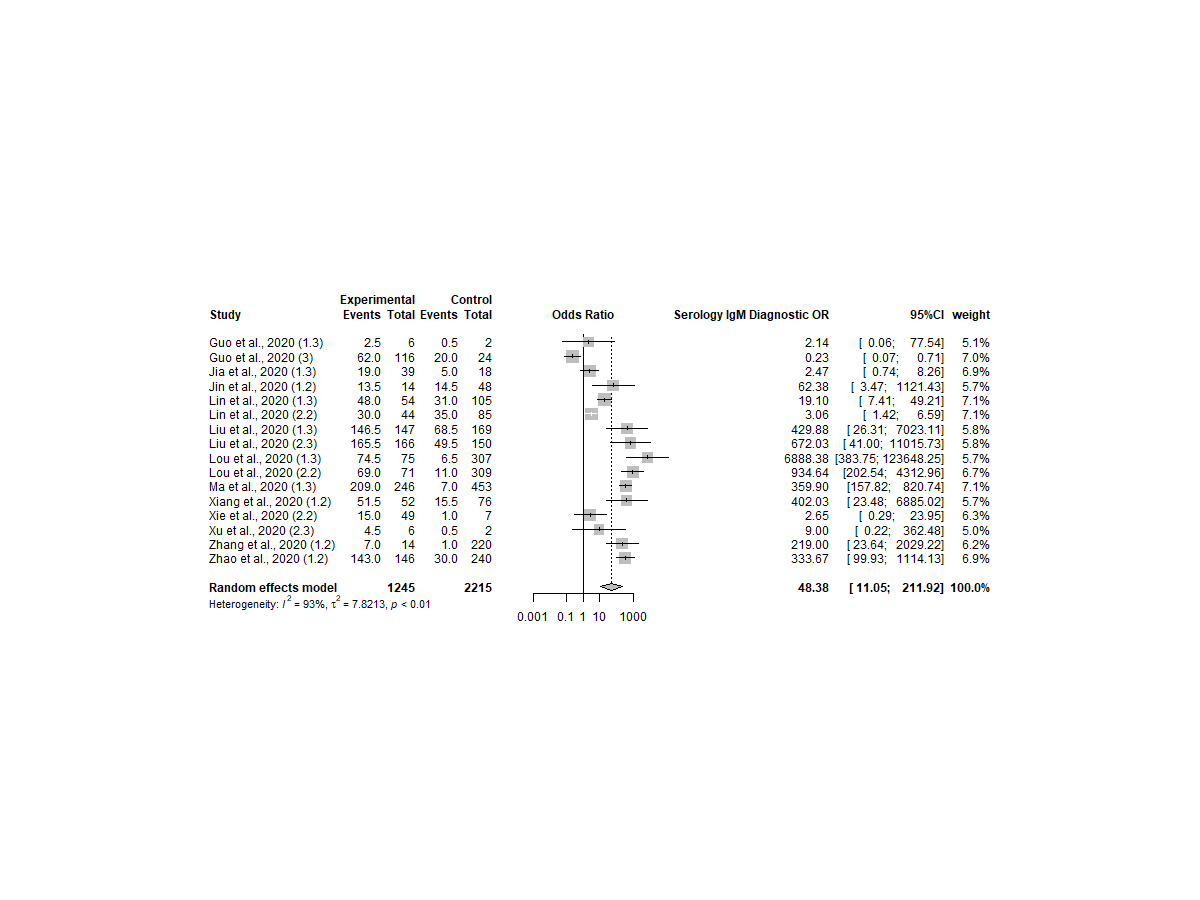


## 8. Serology (Ab)


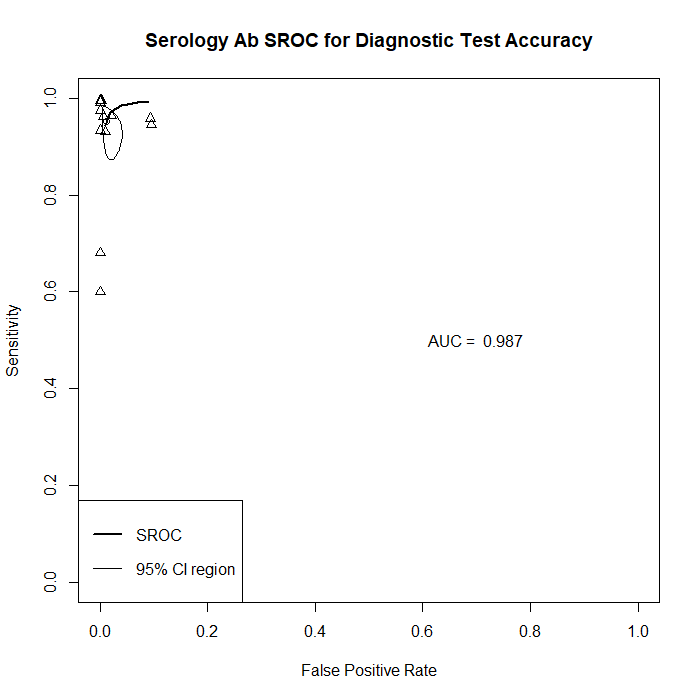

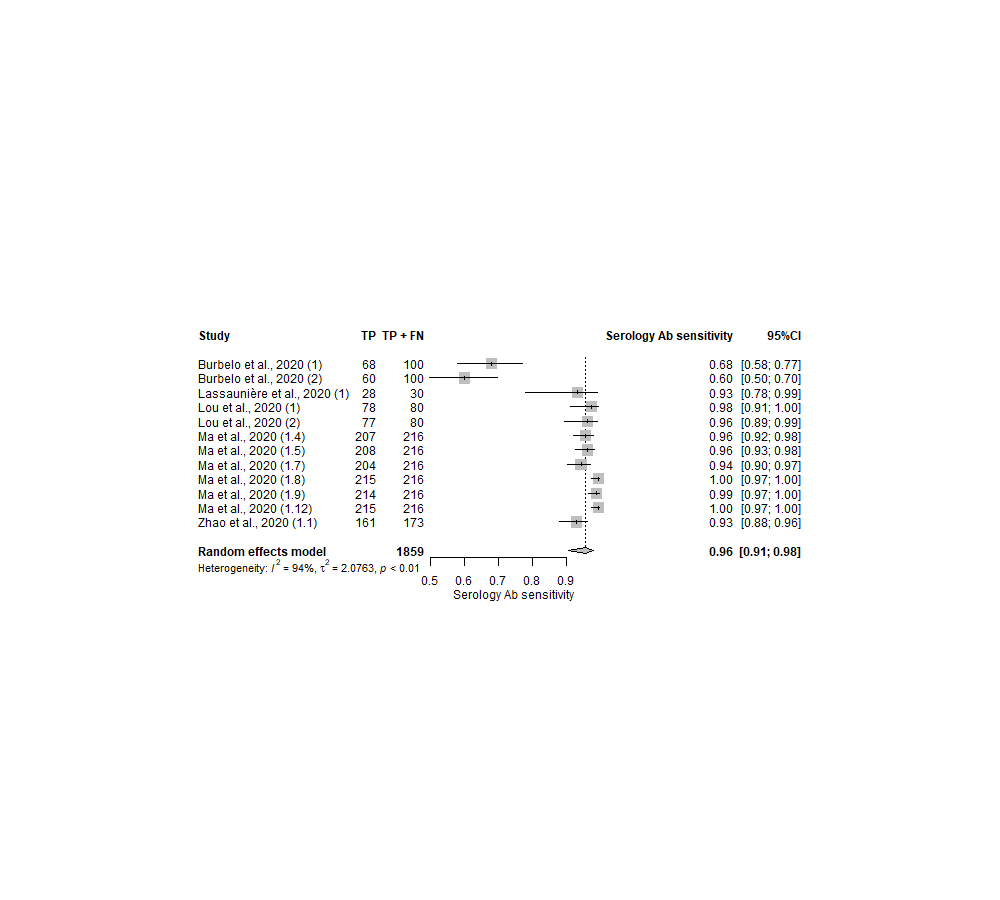

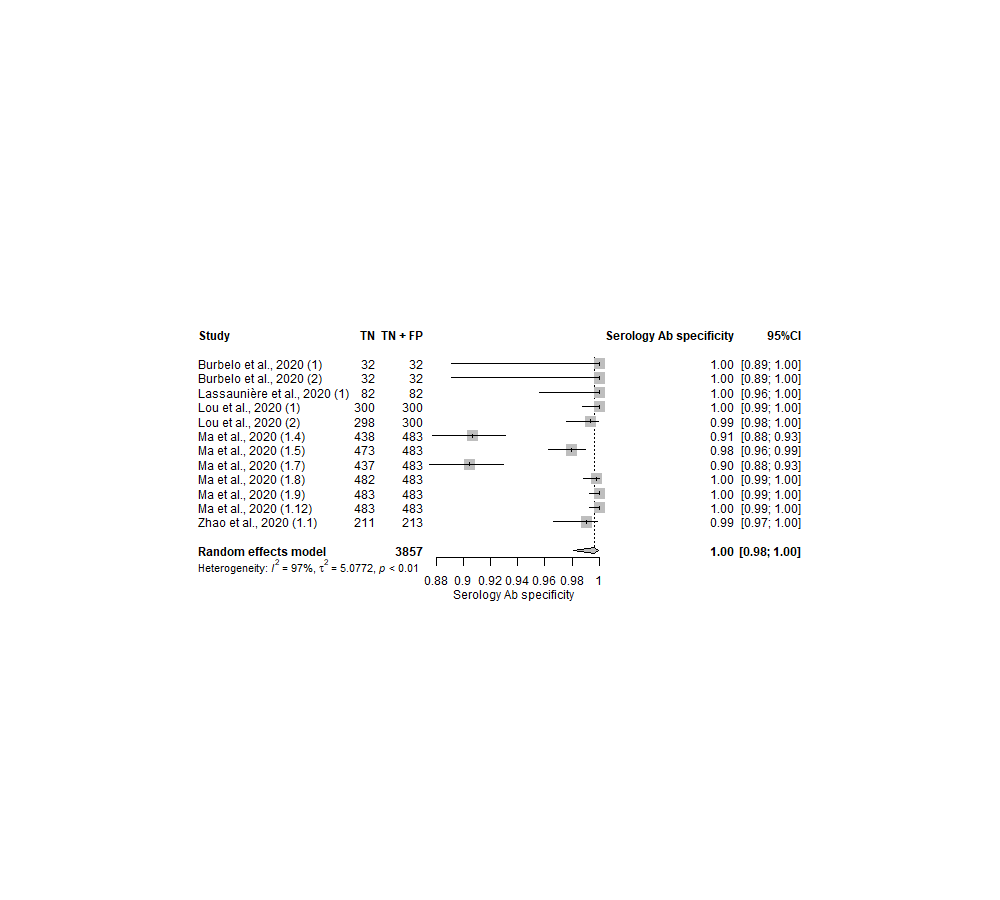


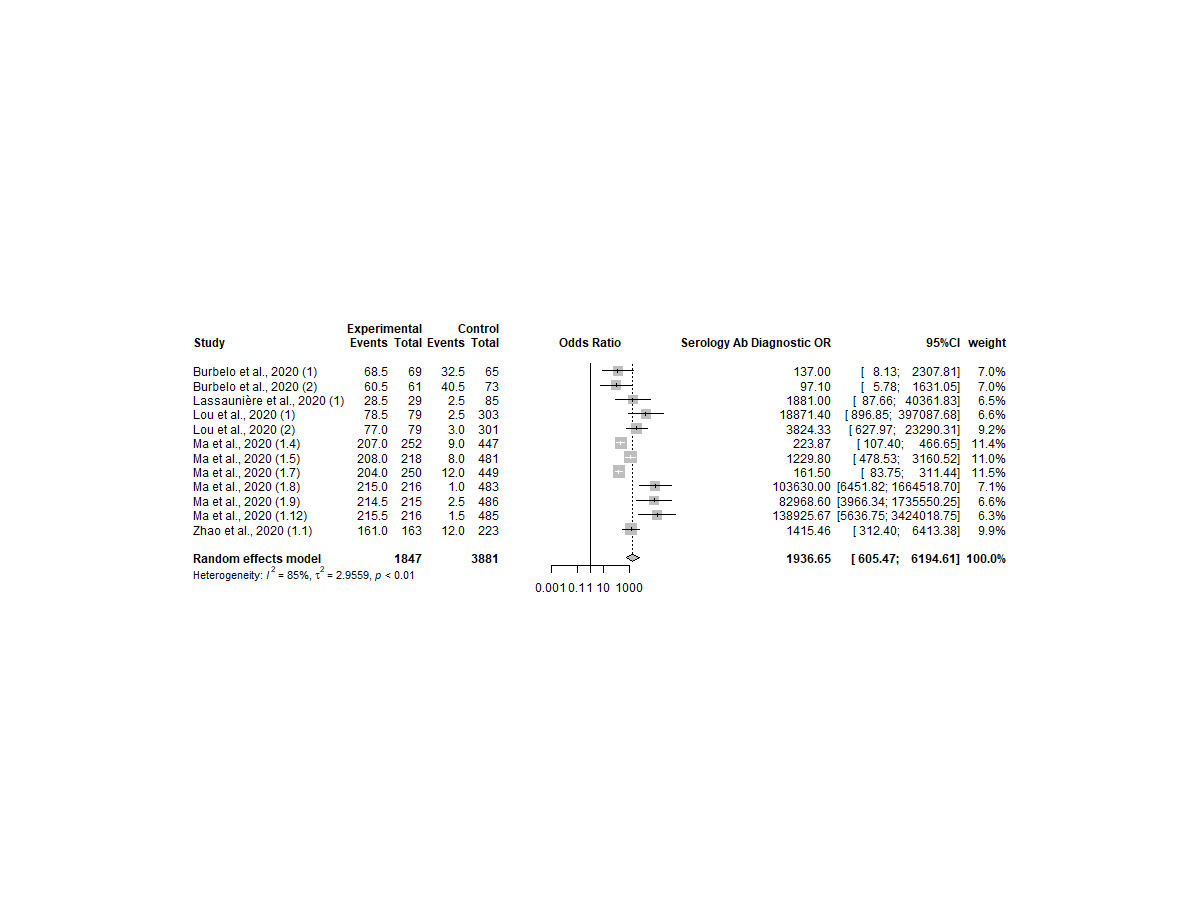


##
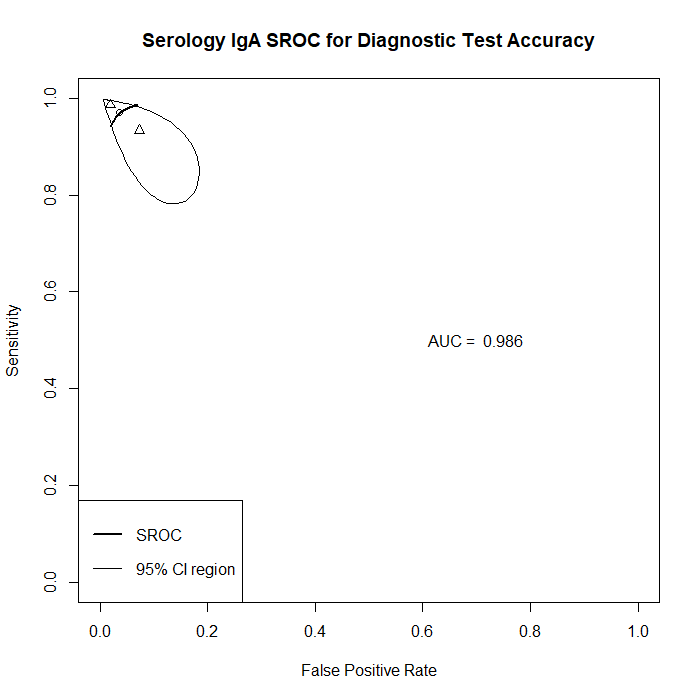
9. Serology (IgA)


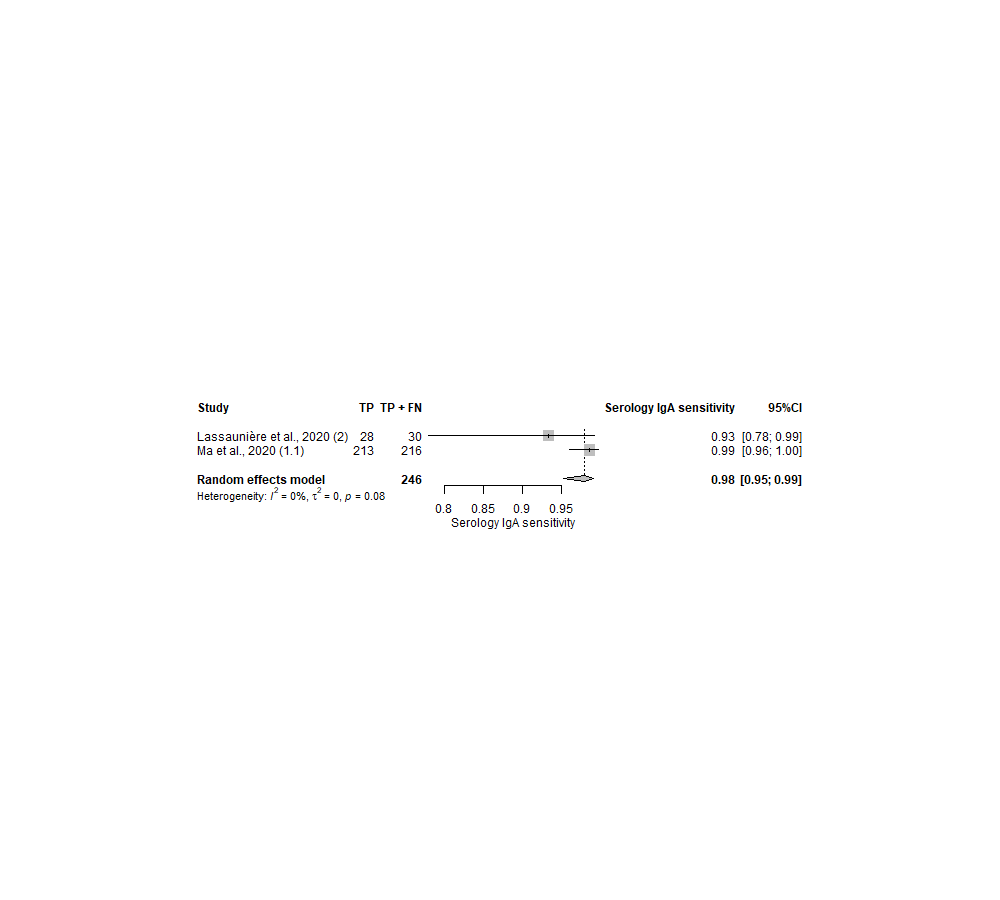


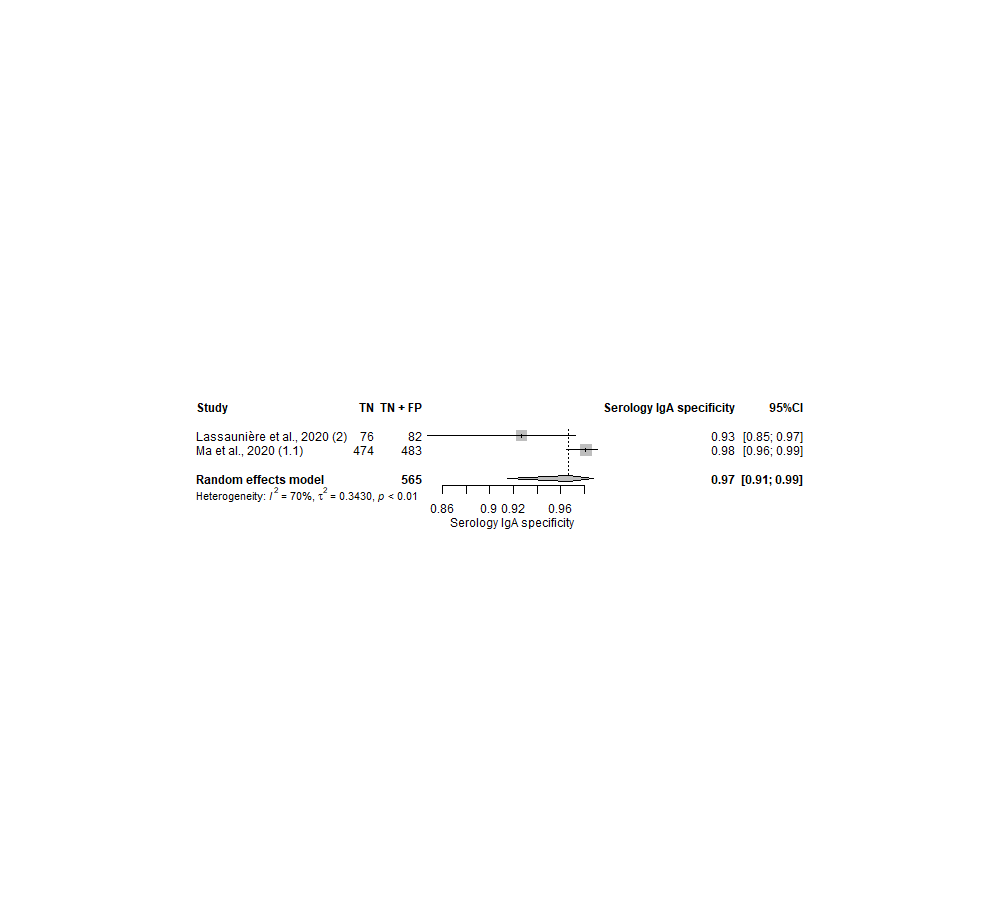


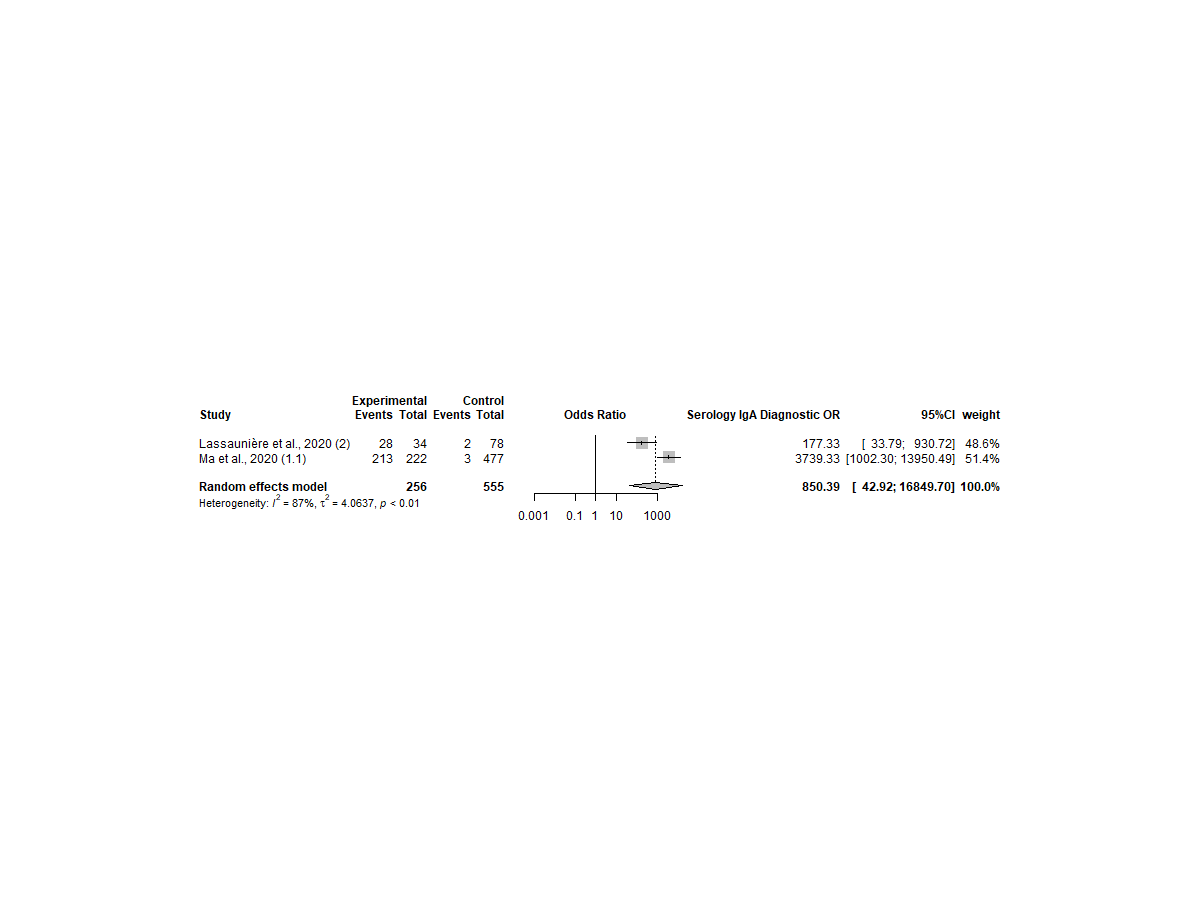


## 10. Serology-POC (IgG and/or IgM)


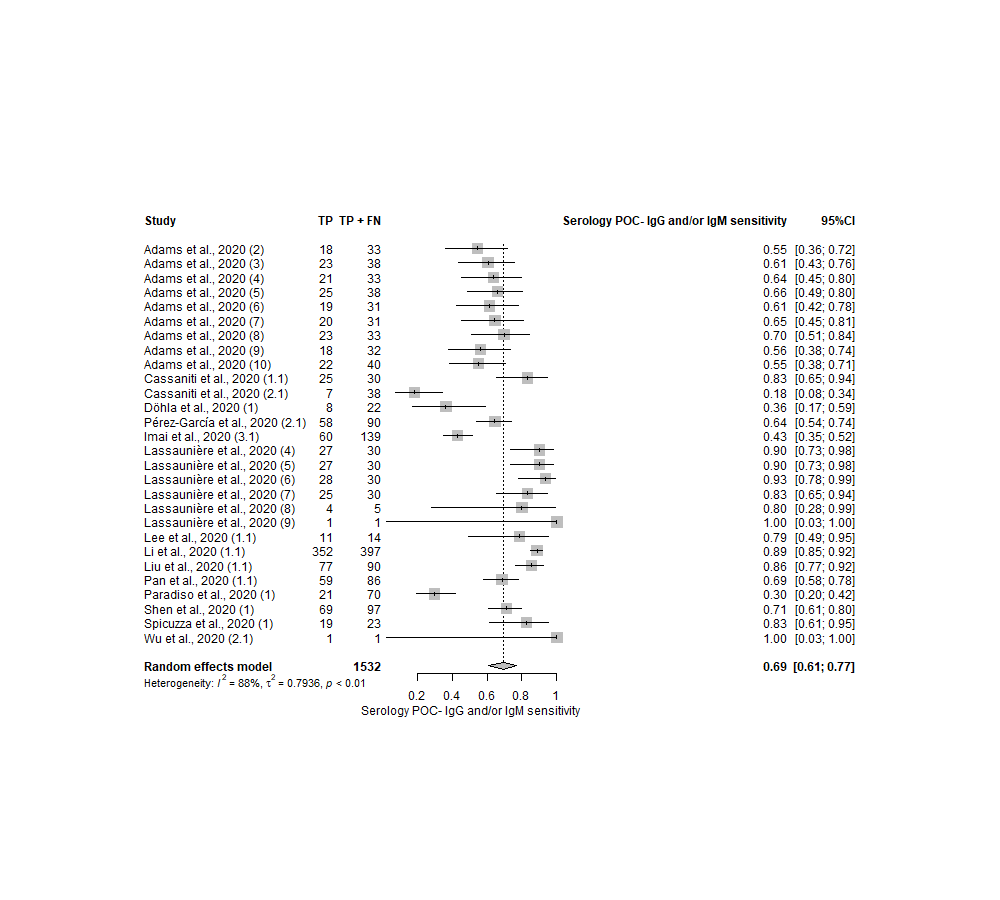

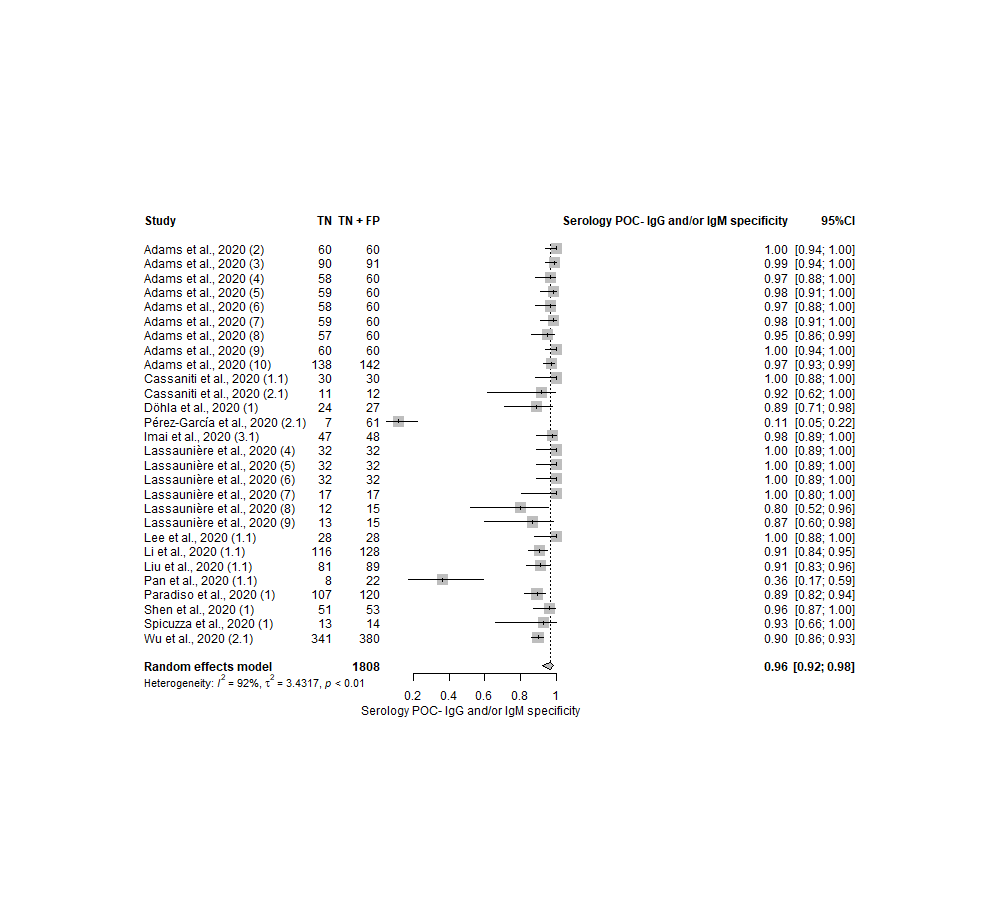

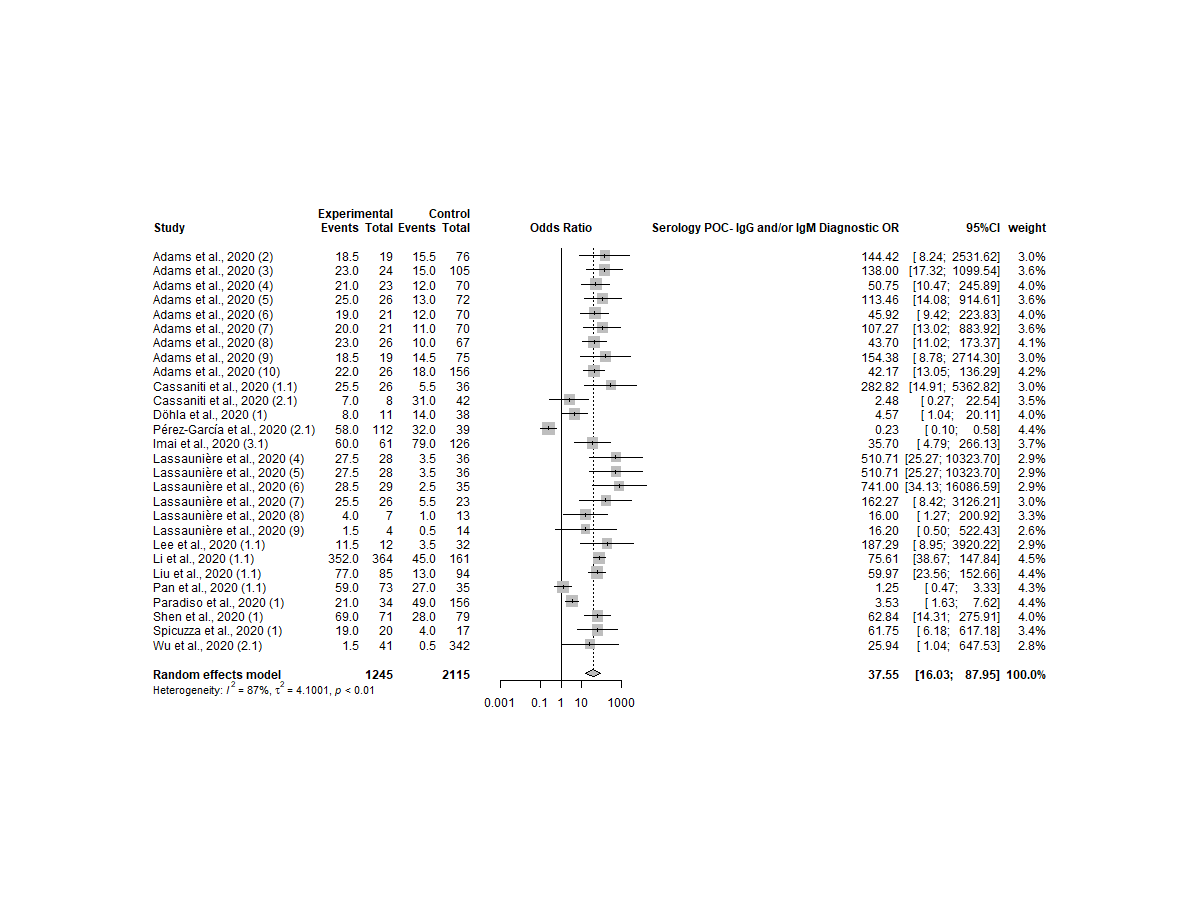

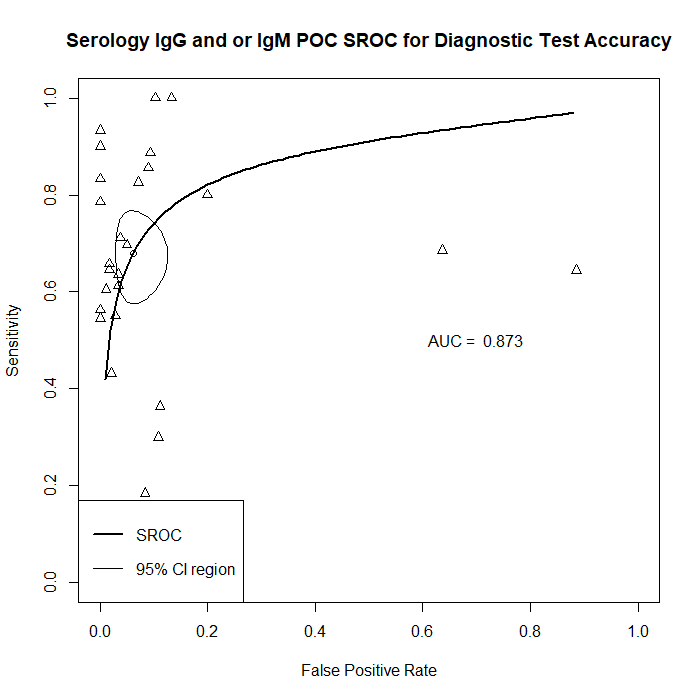


## 11. Serology-POC (IgG and IgM)


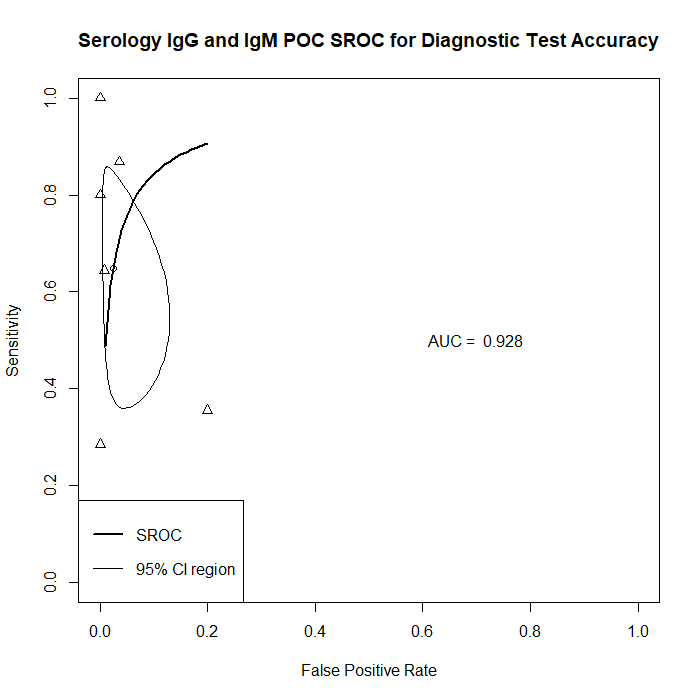

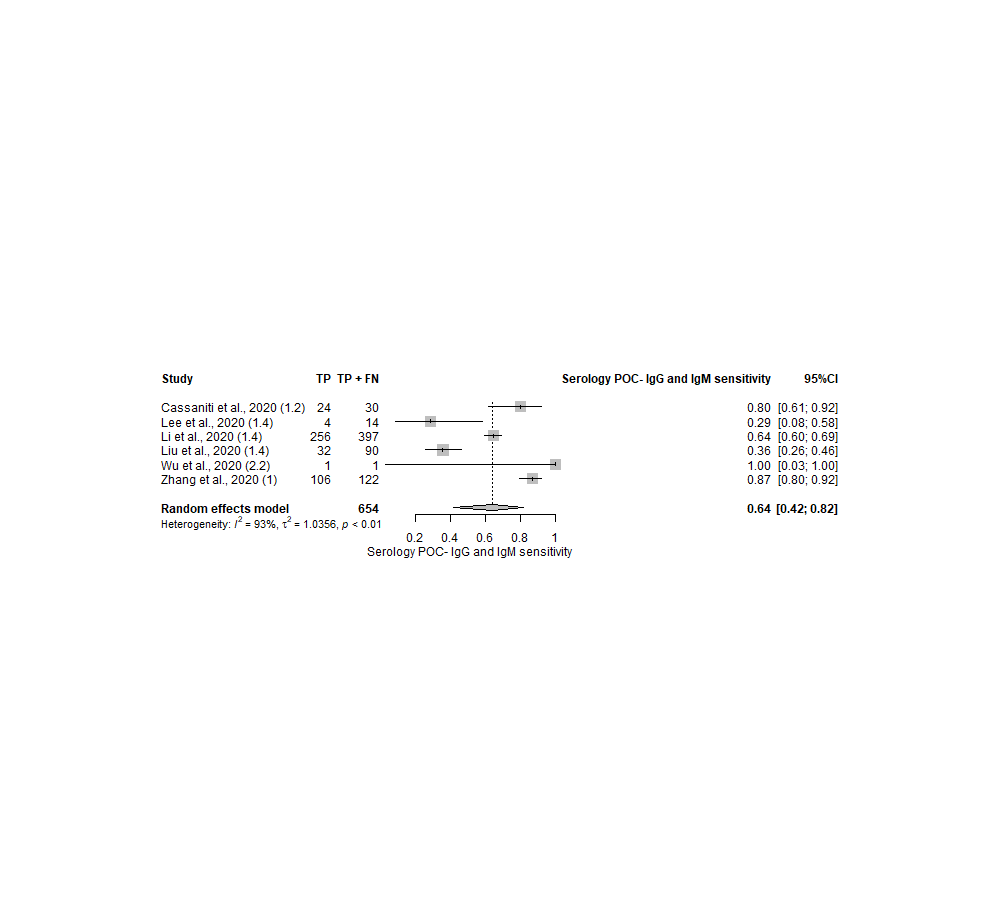


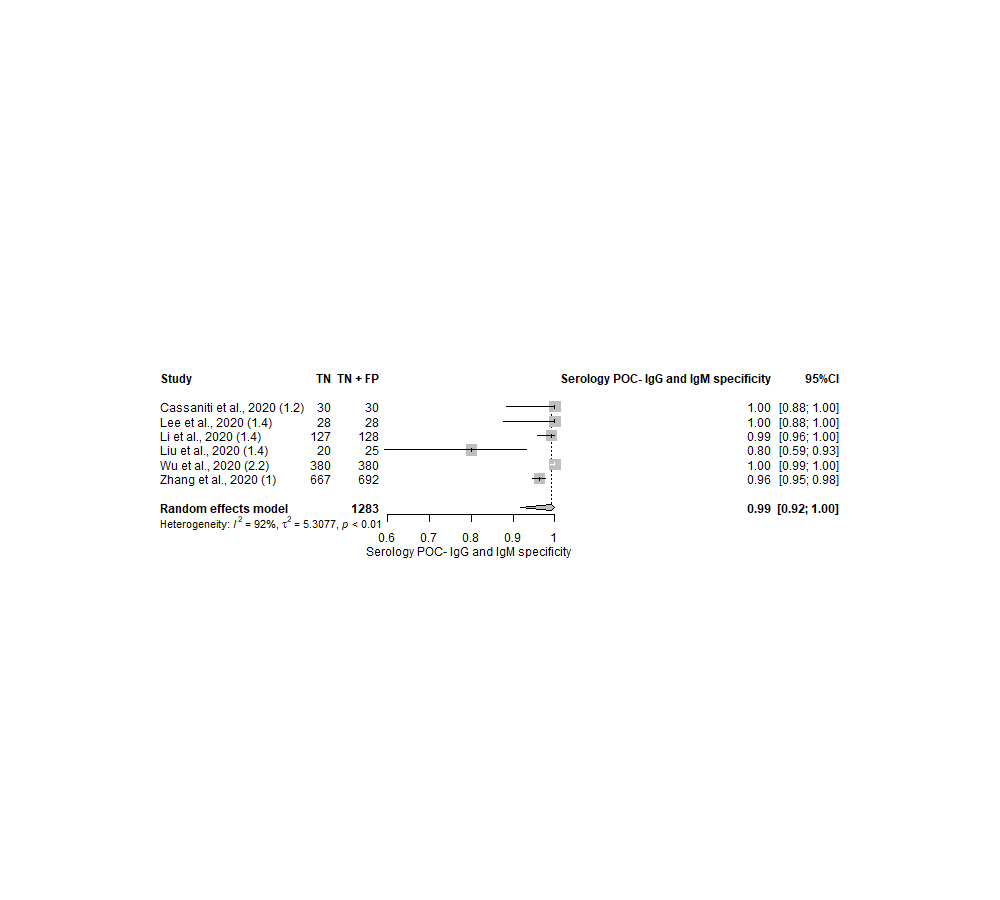


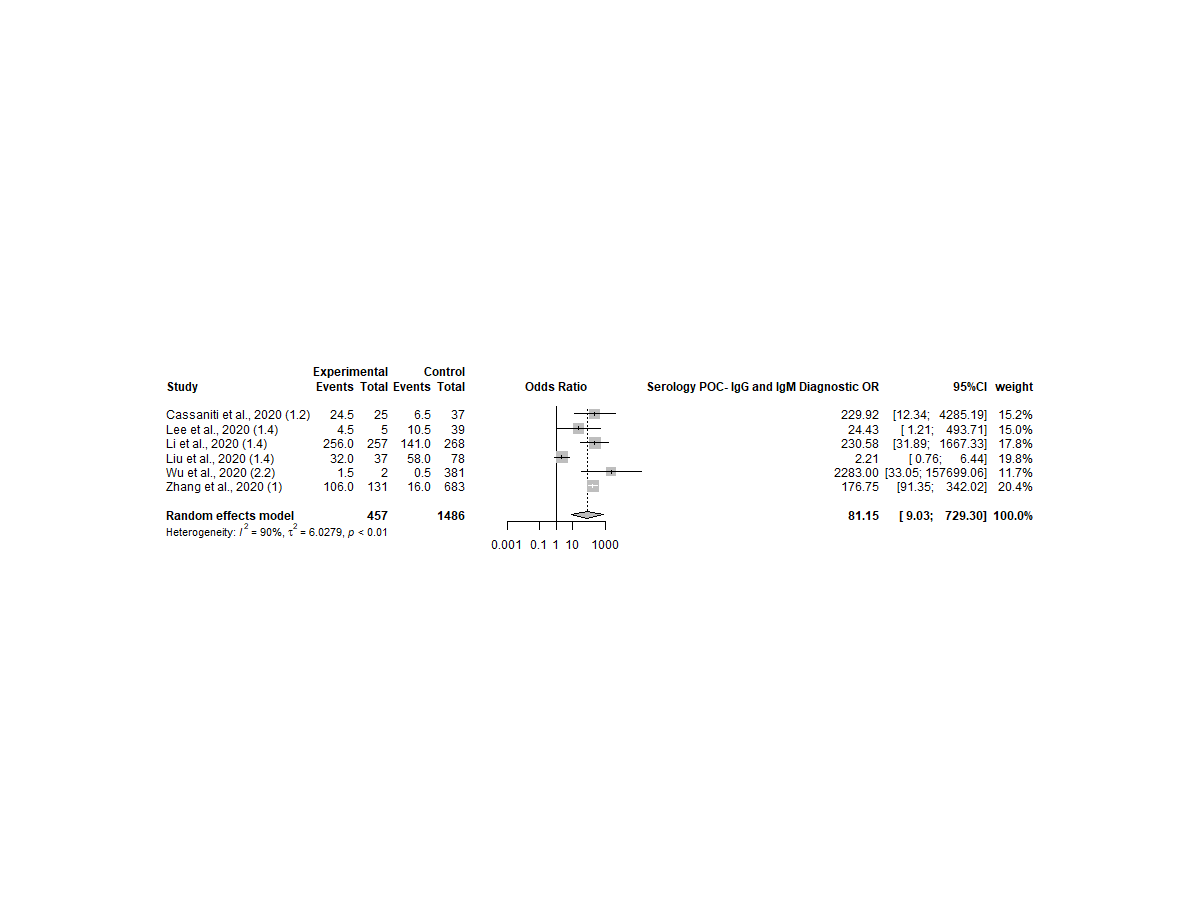


##
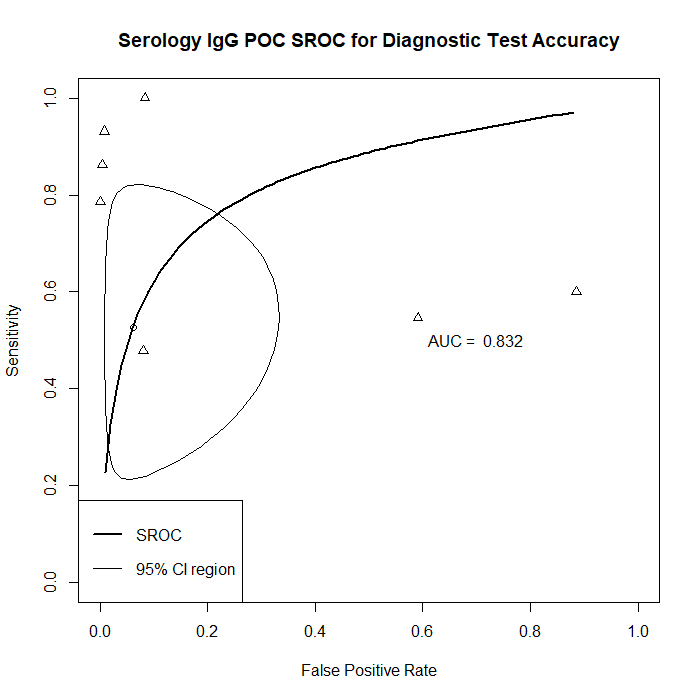
12. Serology-POC (IgG)


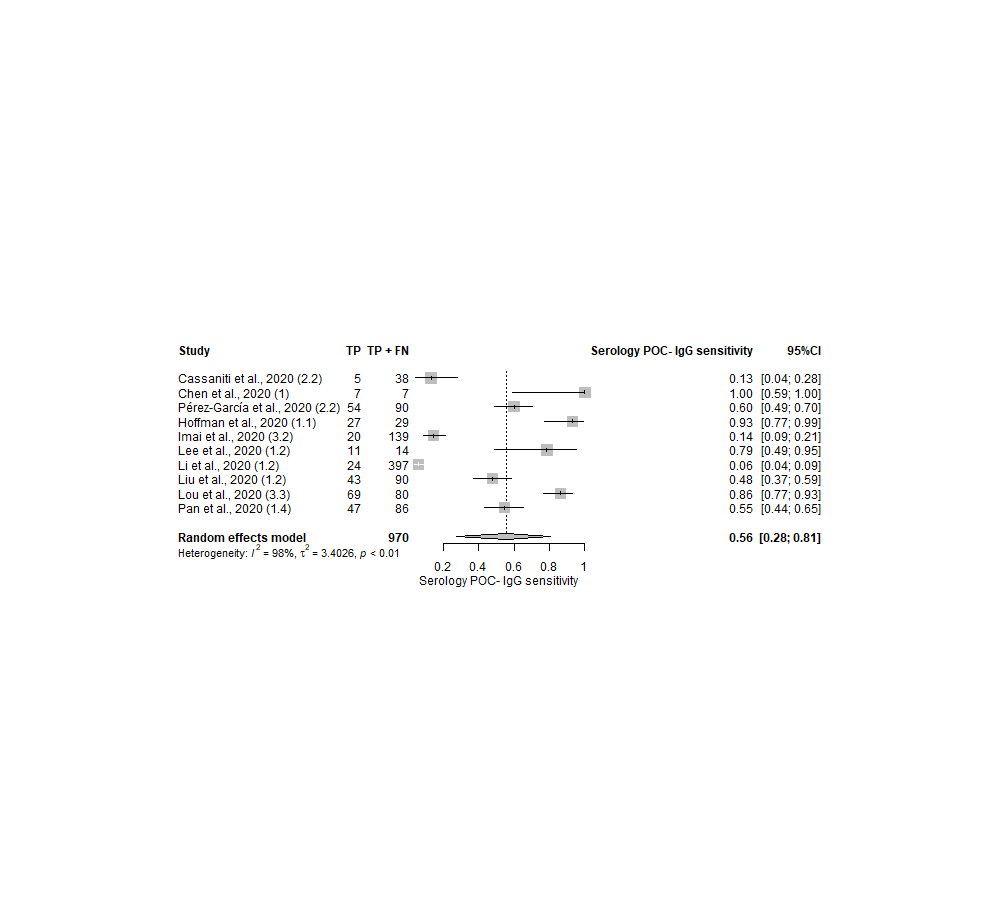


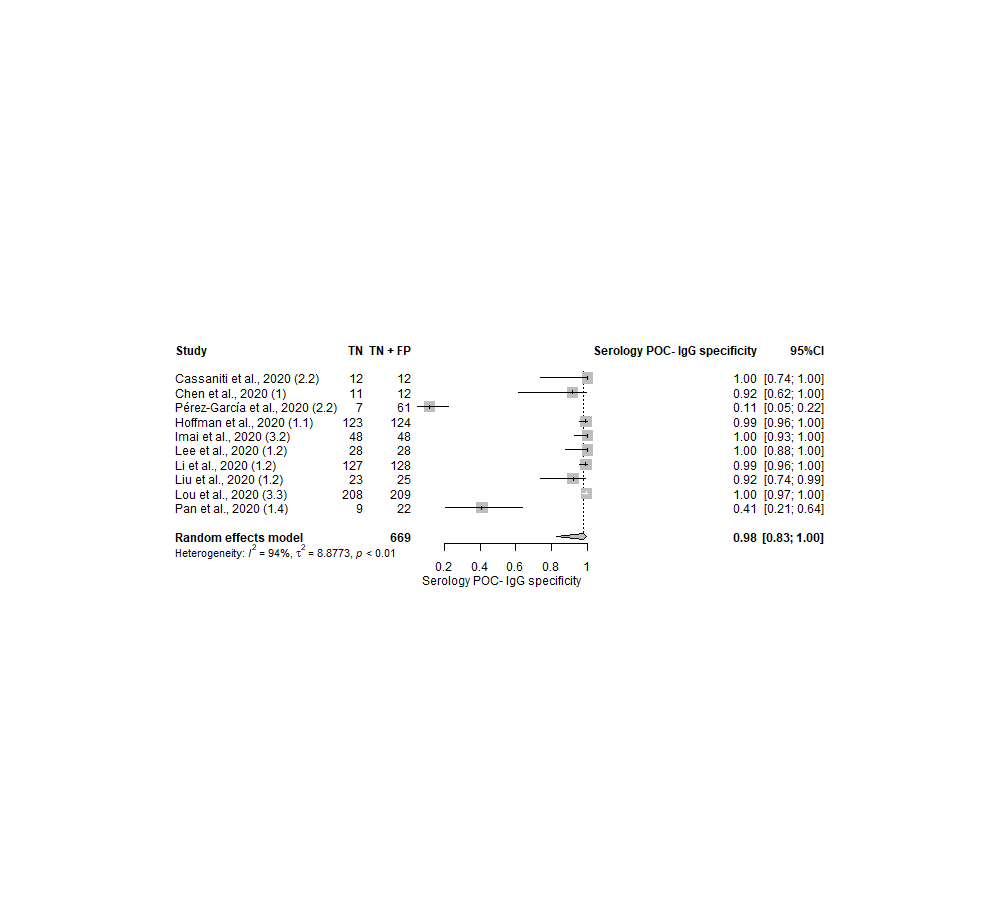


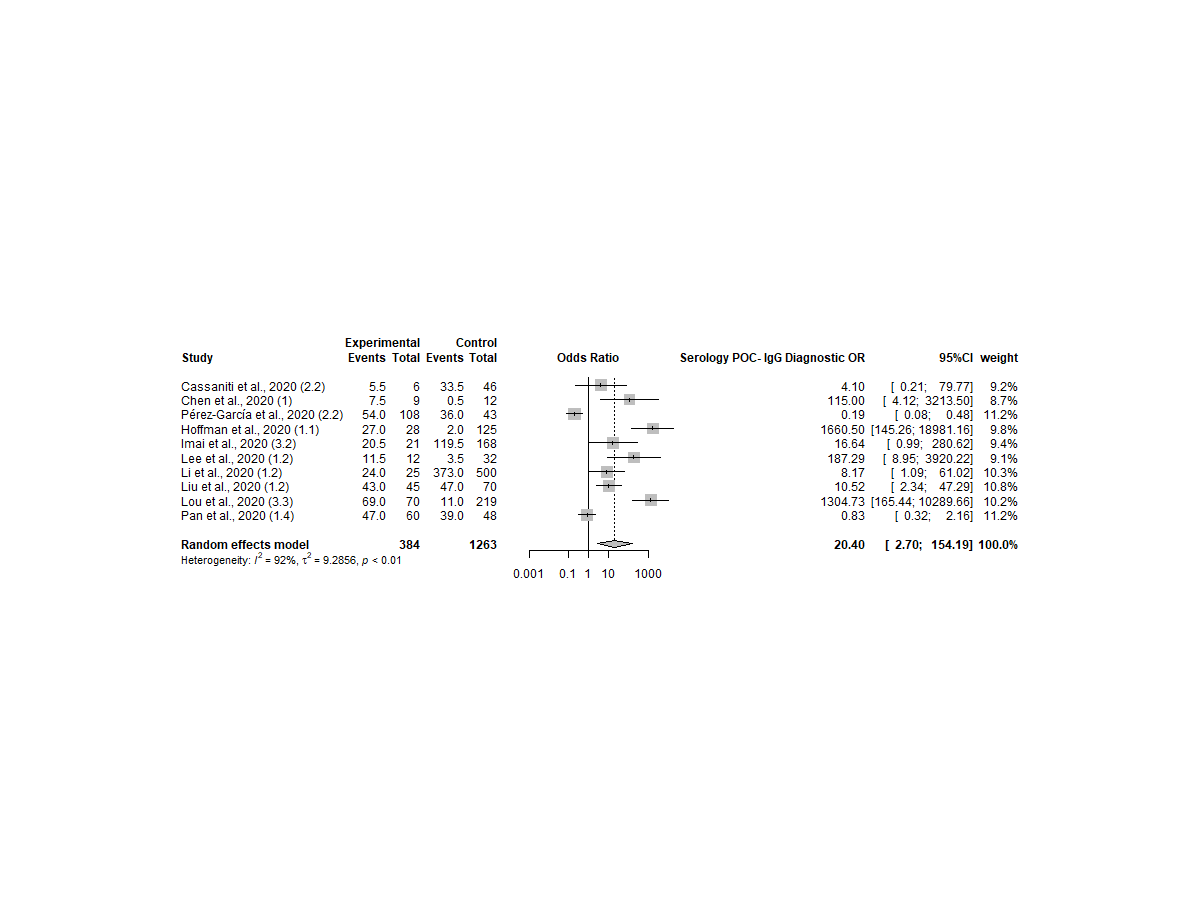


## 13. Serology-POC (IgM)


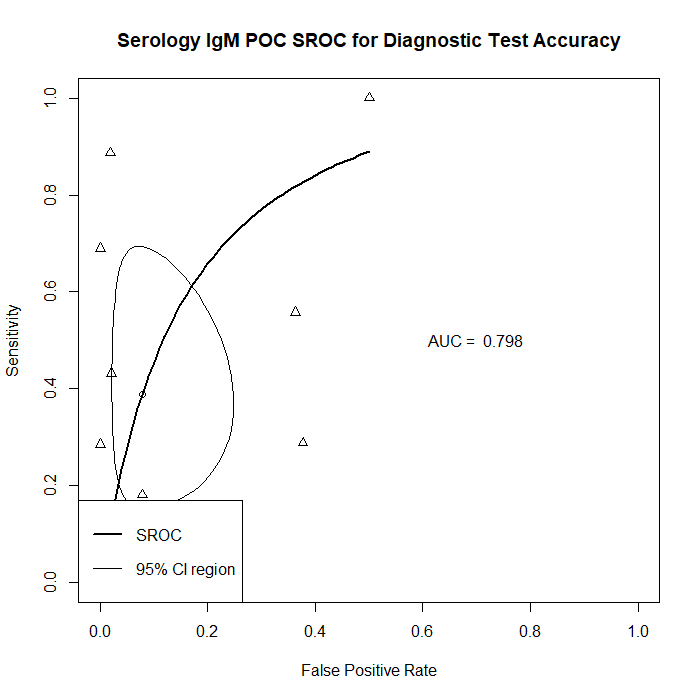

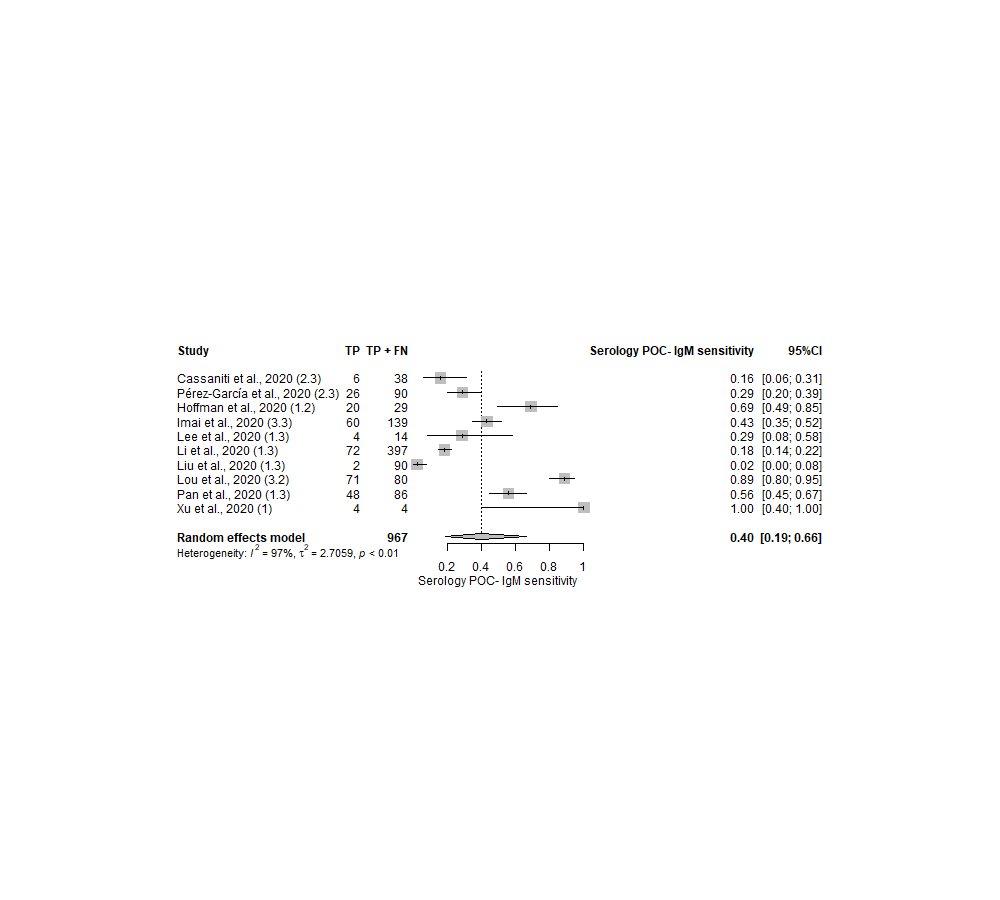


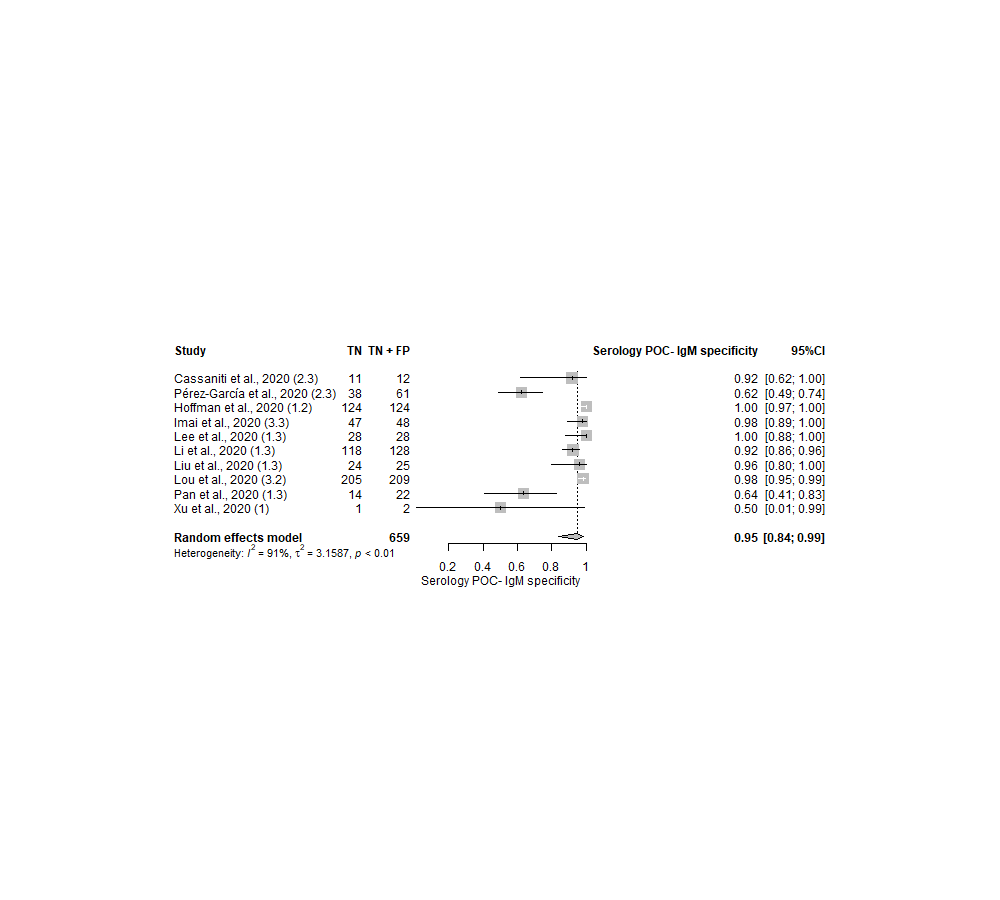


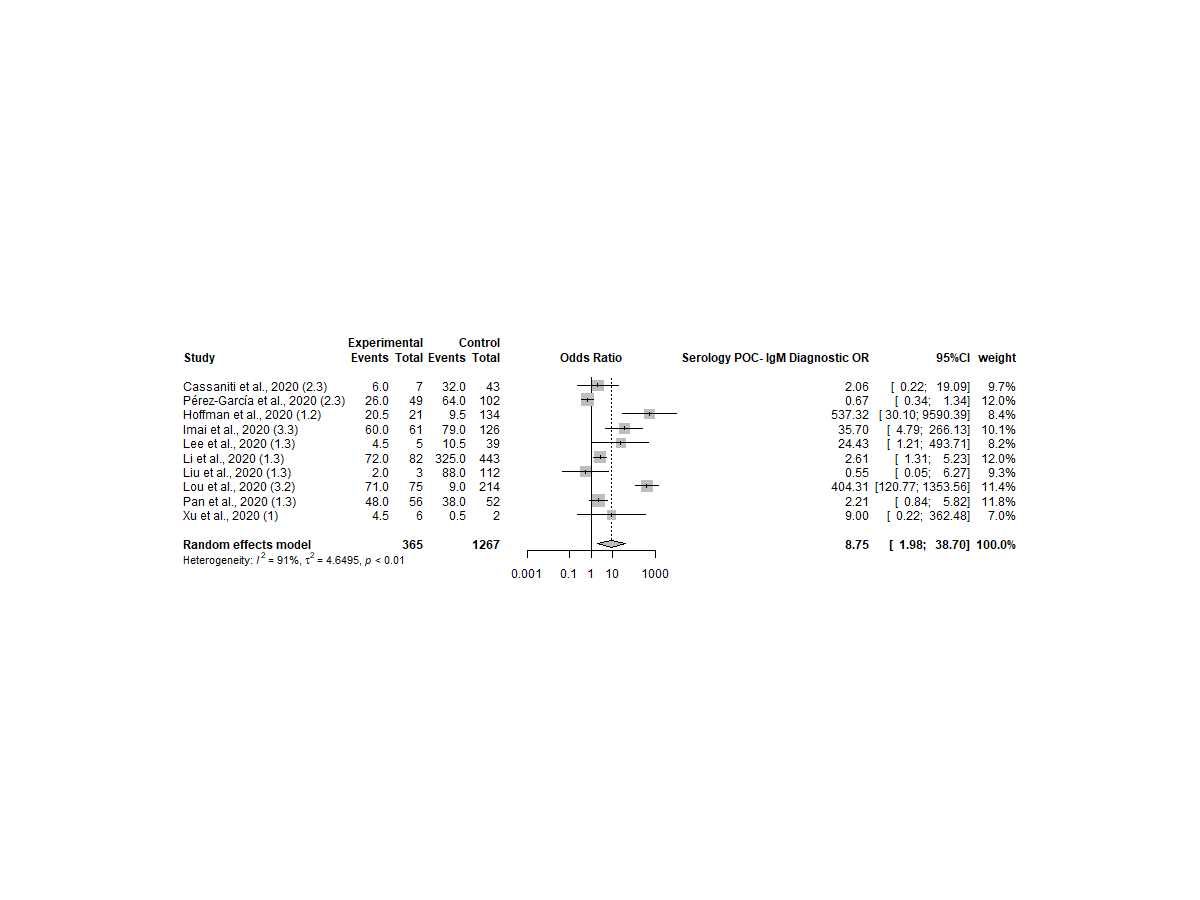


##
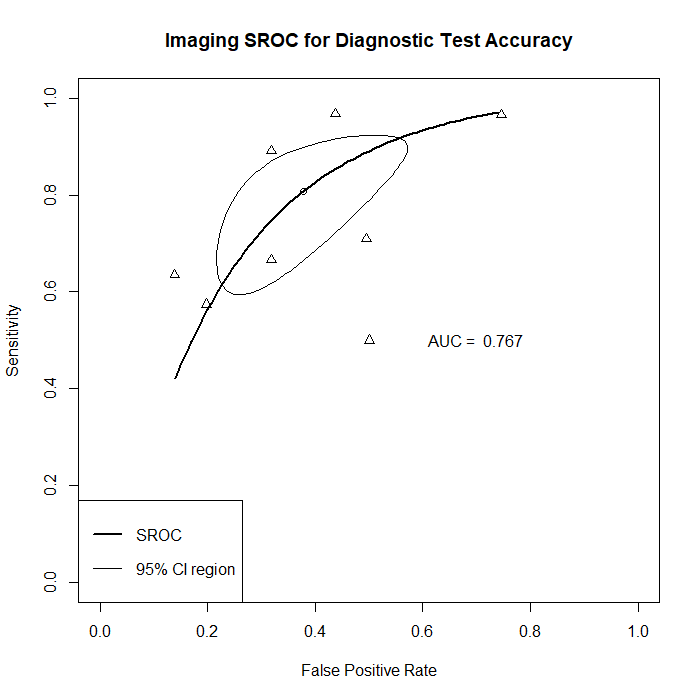
14. Imaging


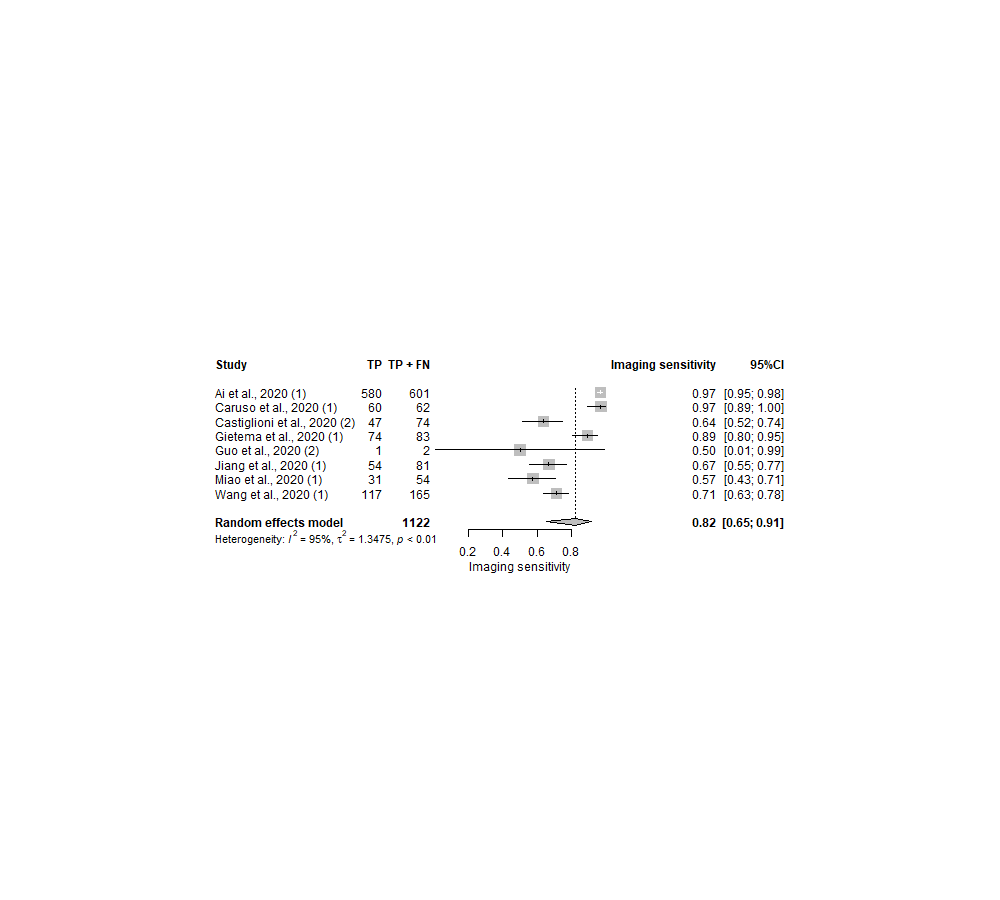


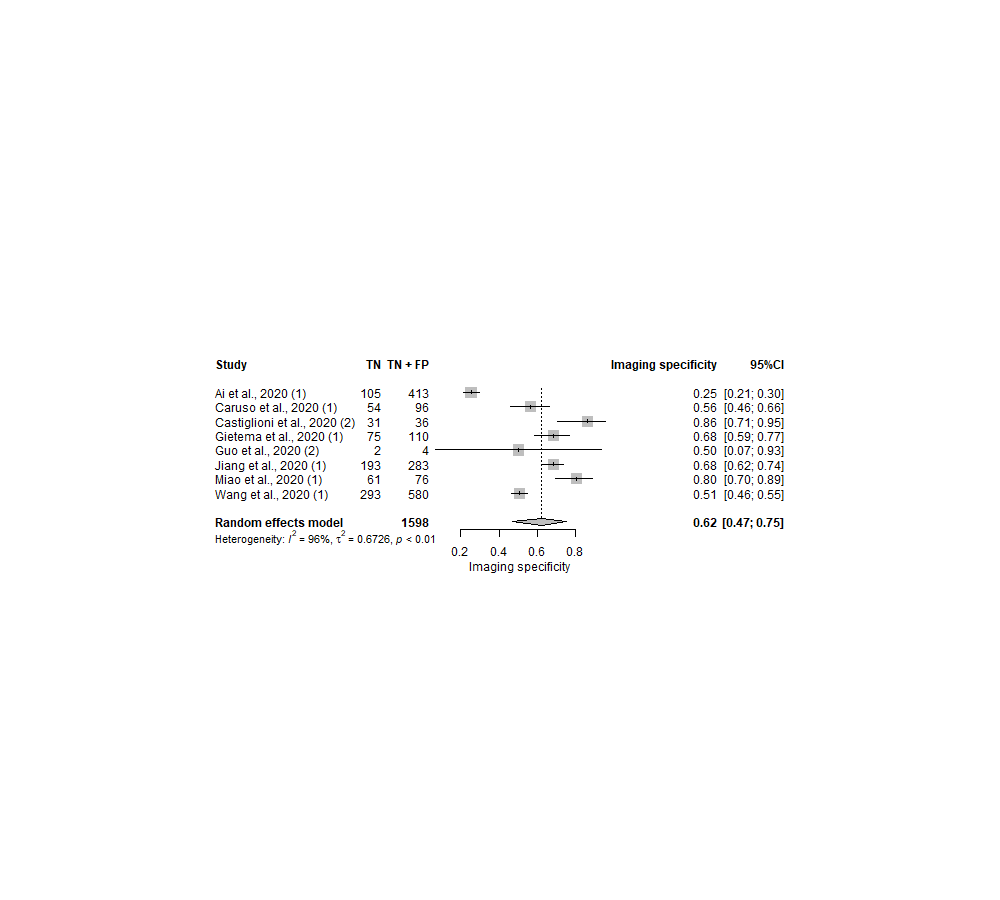


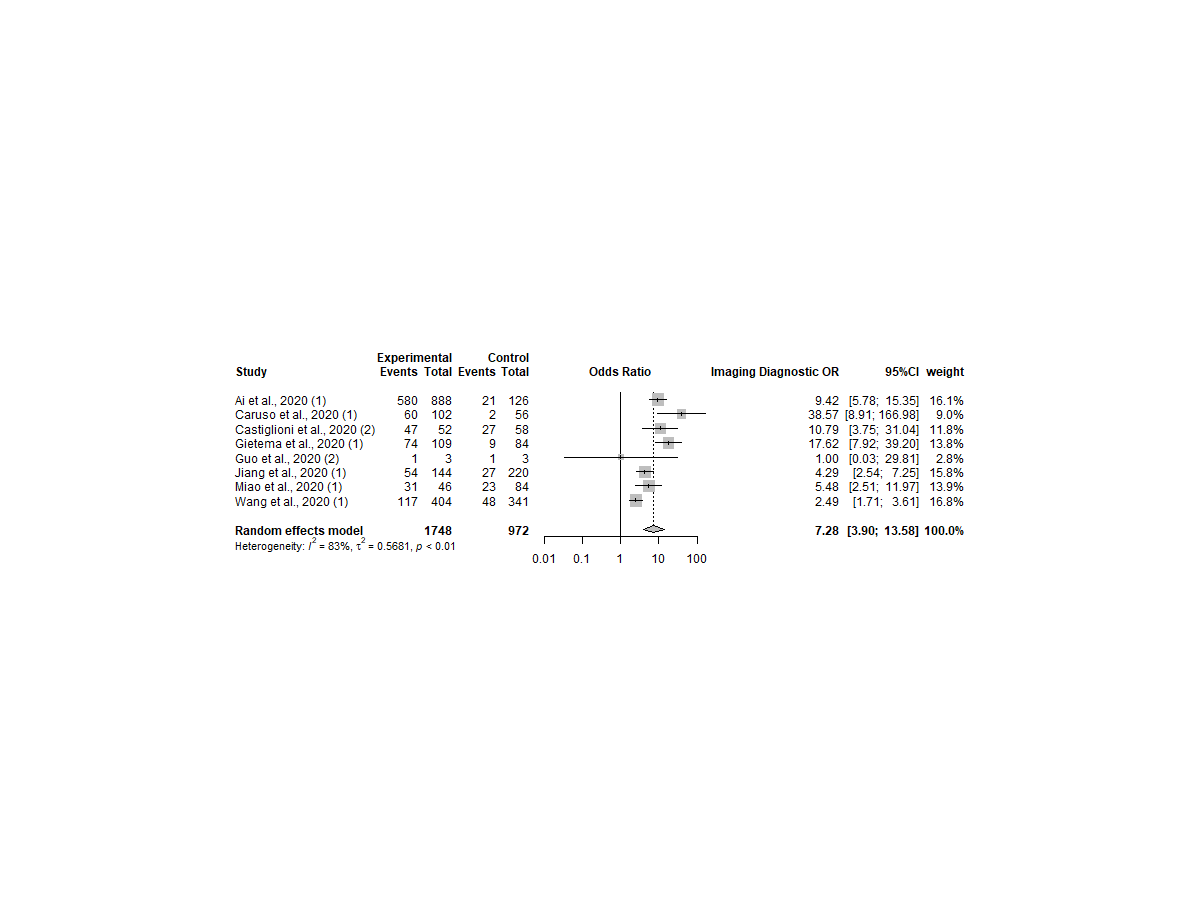


##
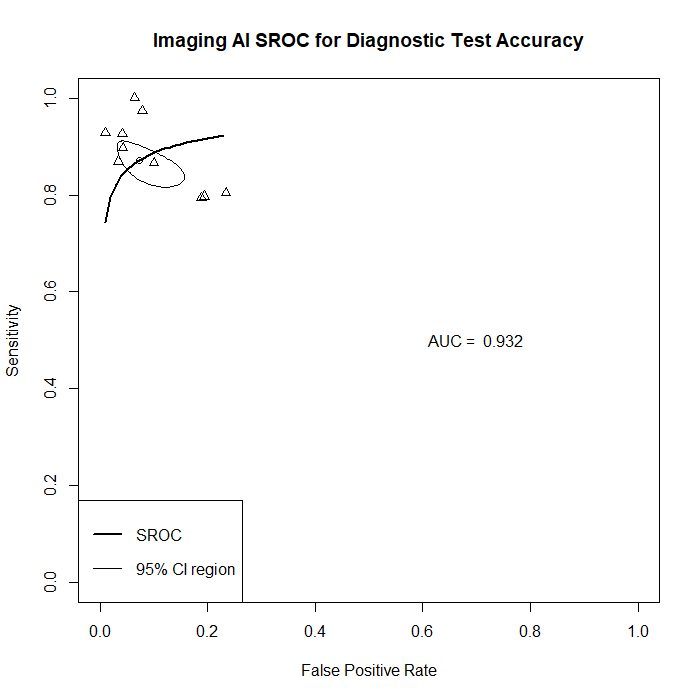
15. Imaging (AI)


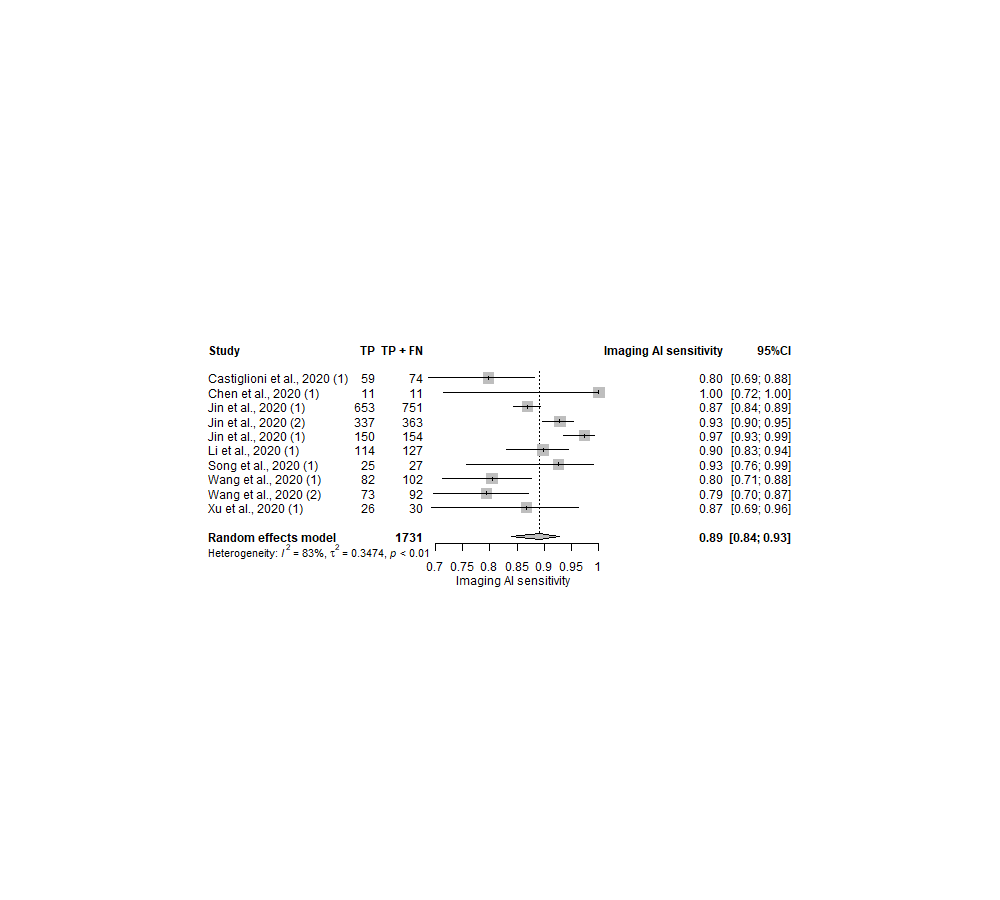


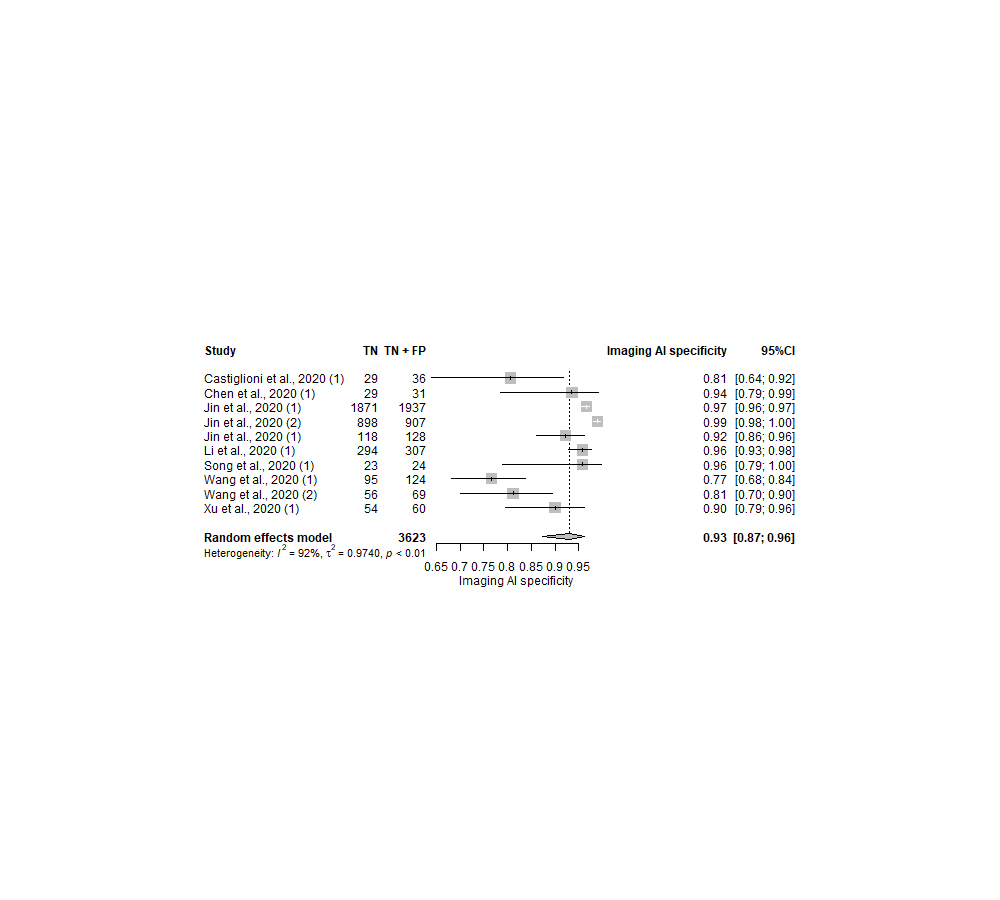


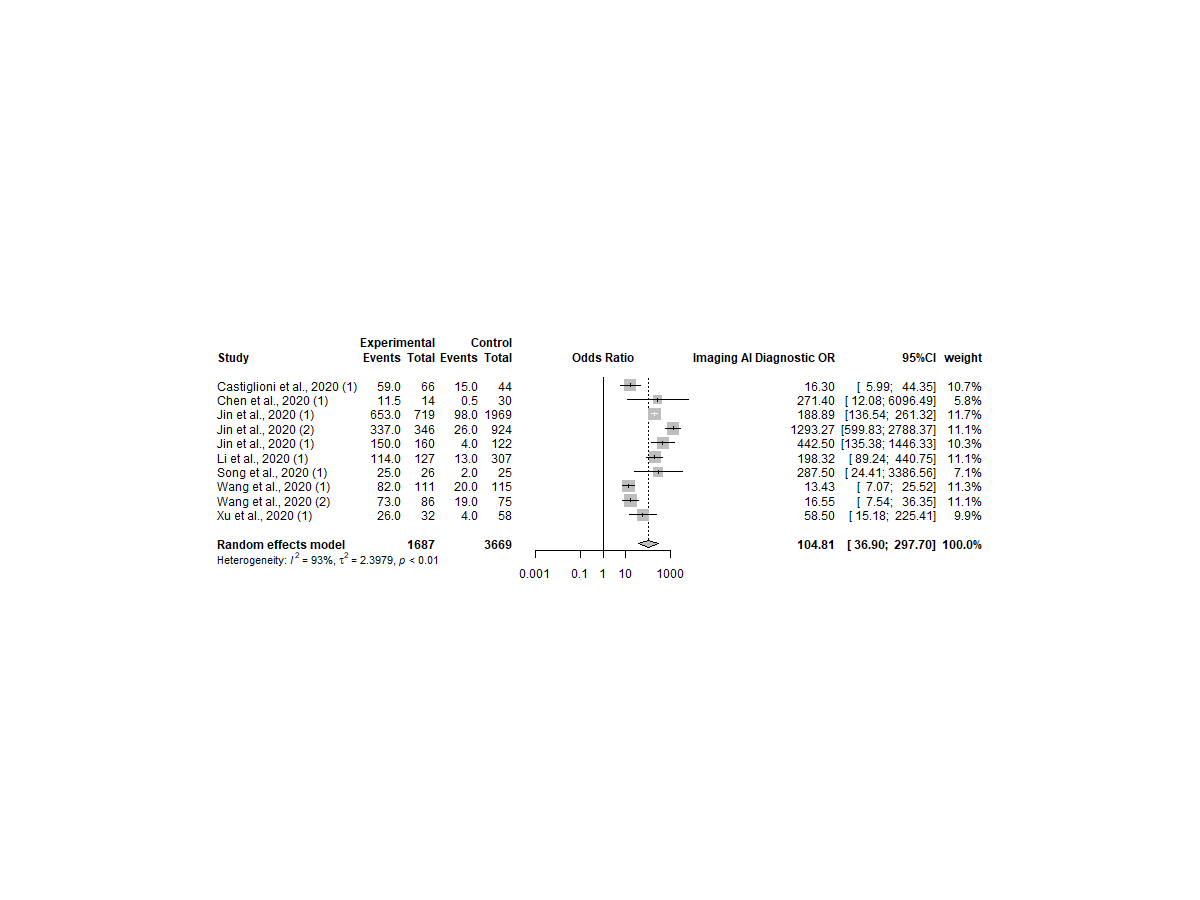


##
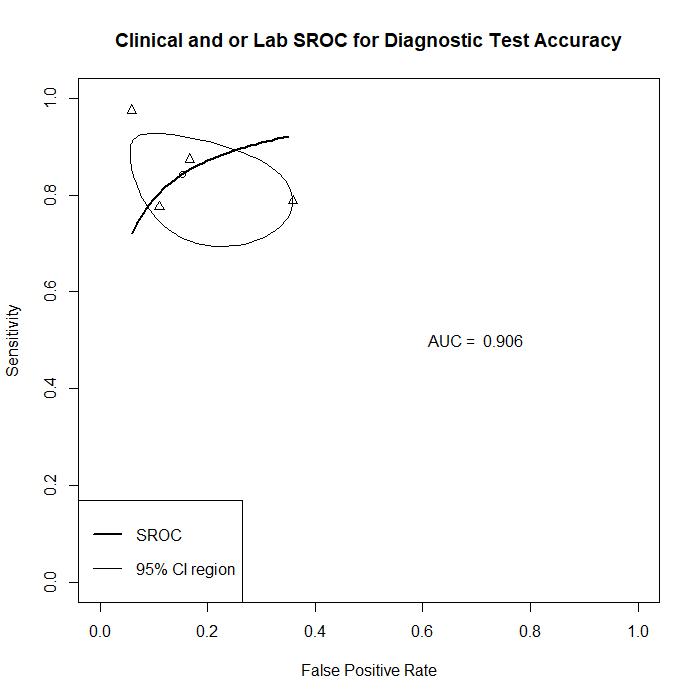
16. Clinical Features and Laboratory Parameters Model


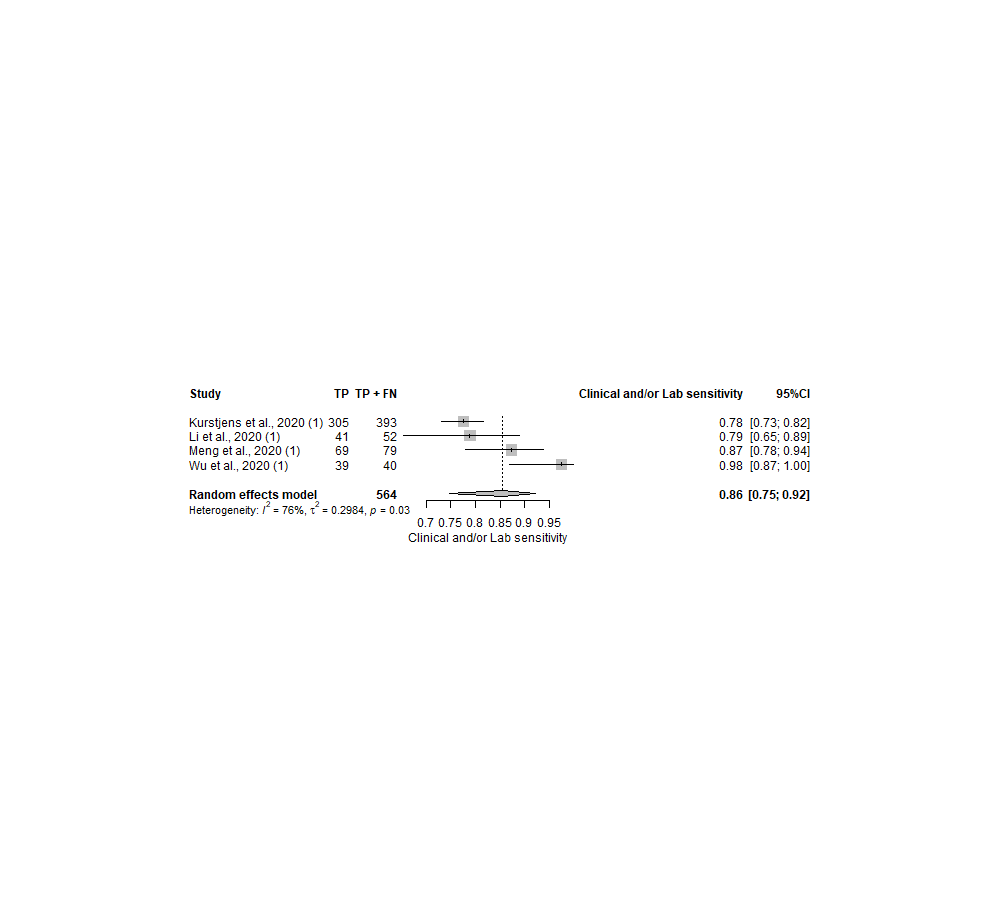


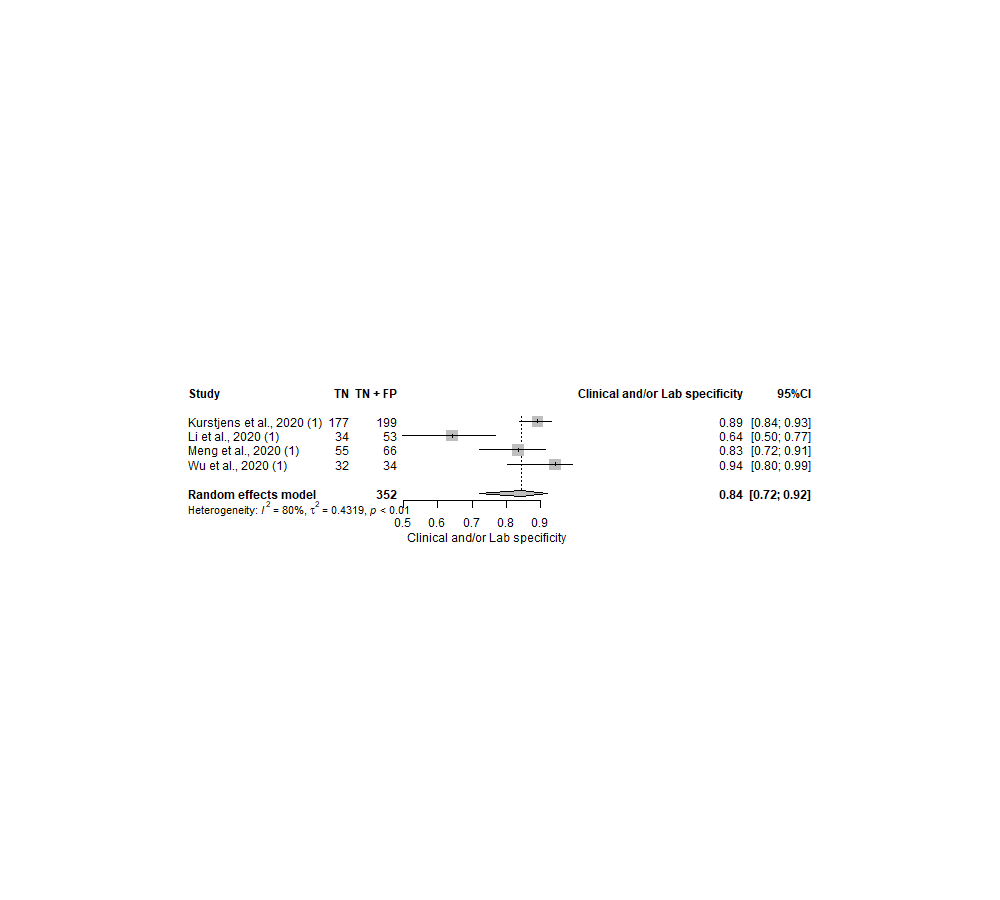


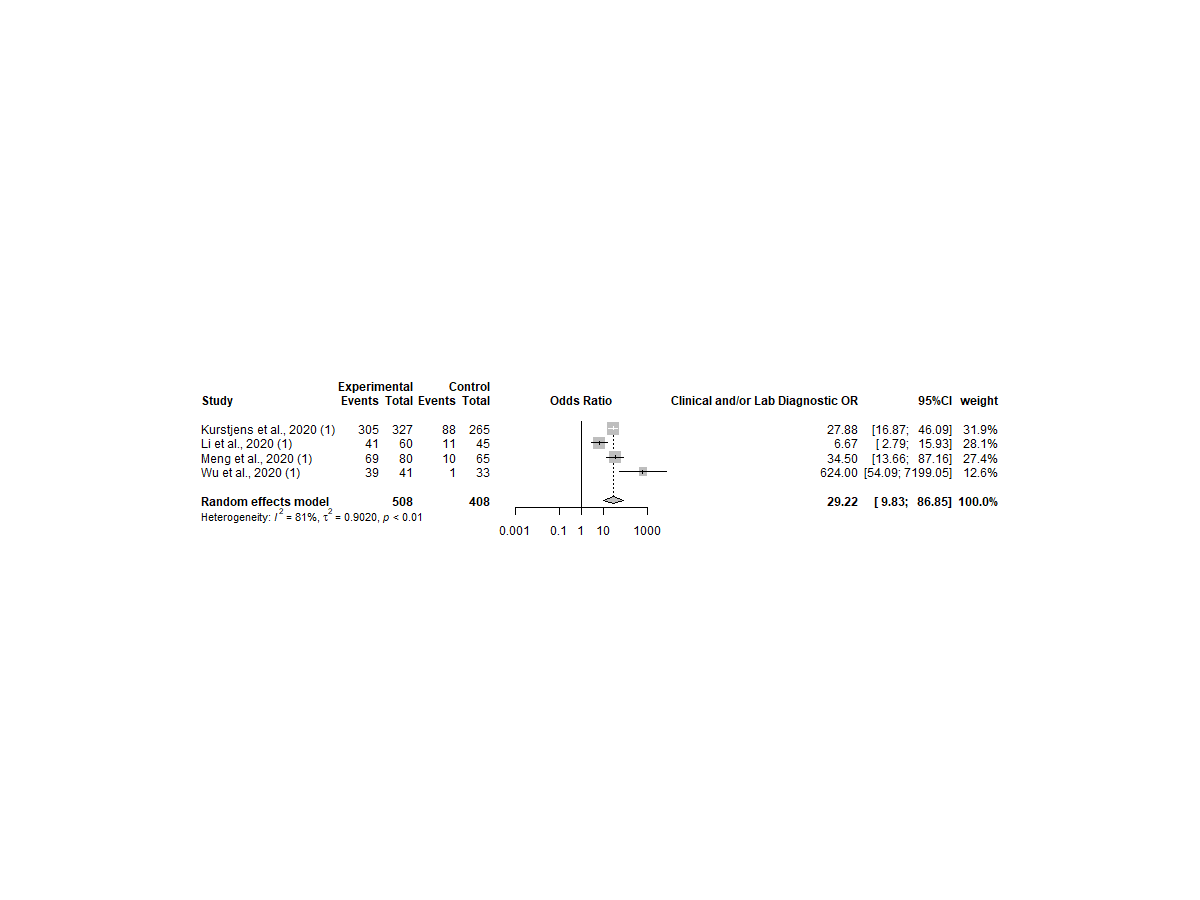


# **Figure S2: Serology Test Late Phase (≥7) Forest Plot on Sensitivity and Specificity, SROC and Diagnostic OR for 1. IgG and/or IgM, 2. IgG, 3. IgM, 4. Ab, 5. IgG and/or IgM (POC), 6. IgG (POC) and 7. IgM (POC)**

## IgG and/or IgM


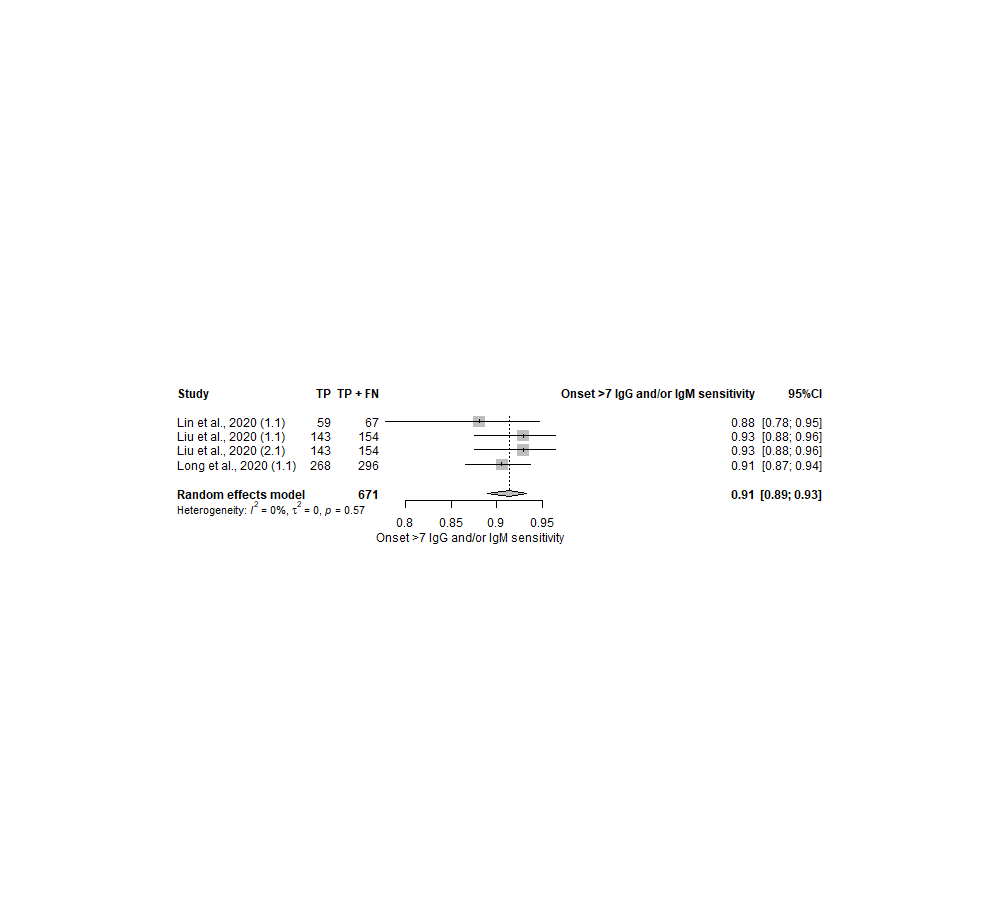


## IgG


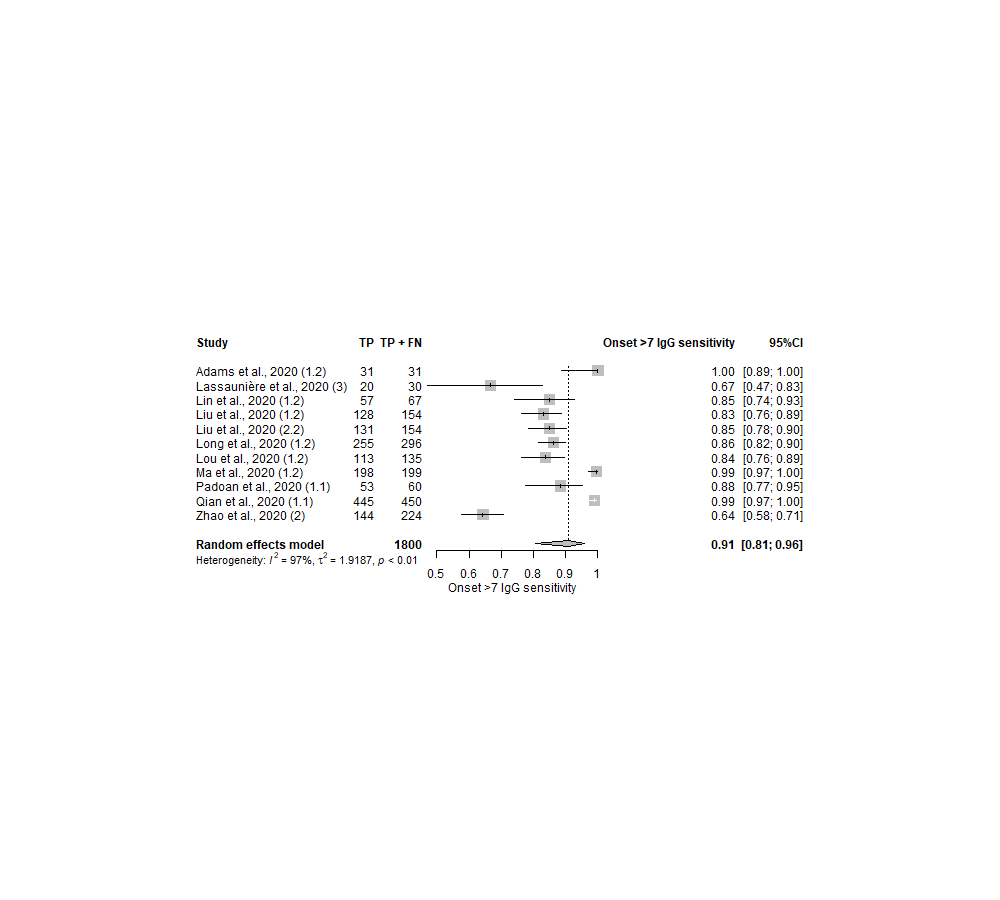


## IgM


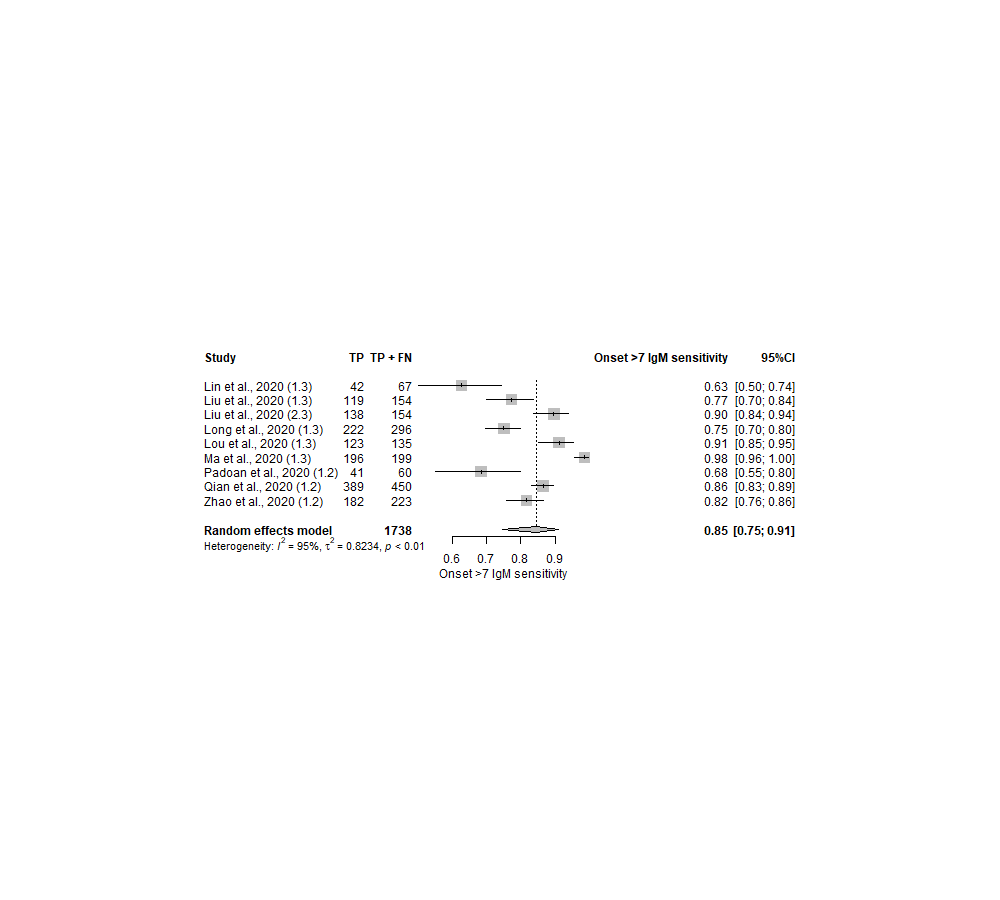


##
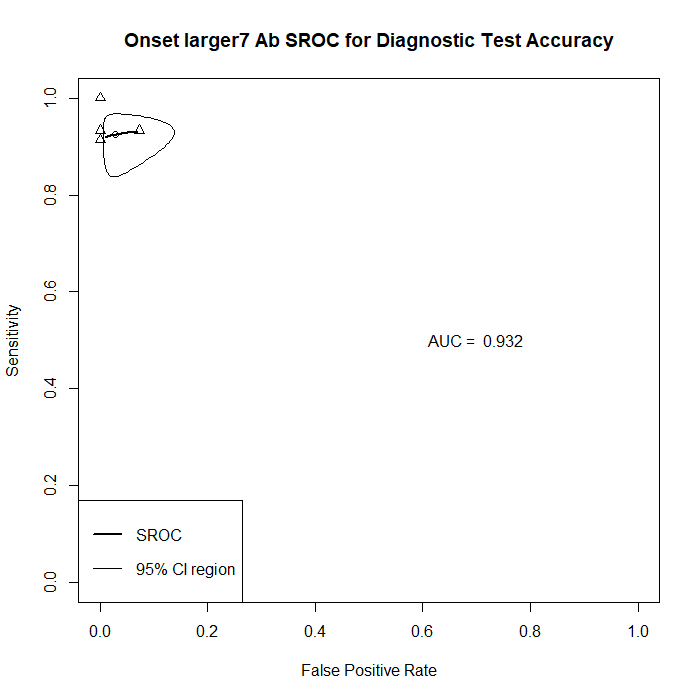
Ab


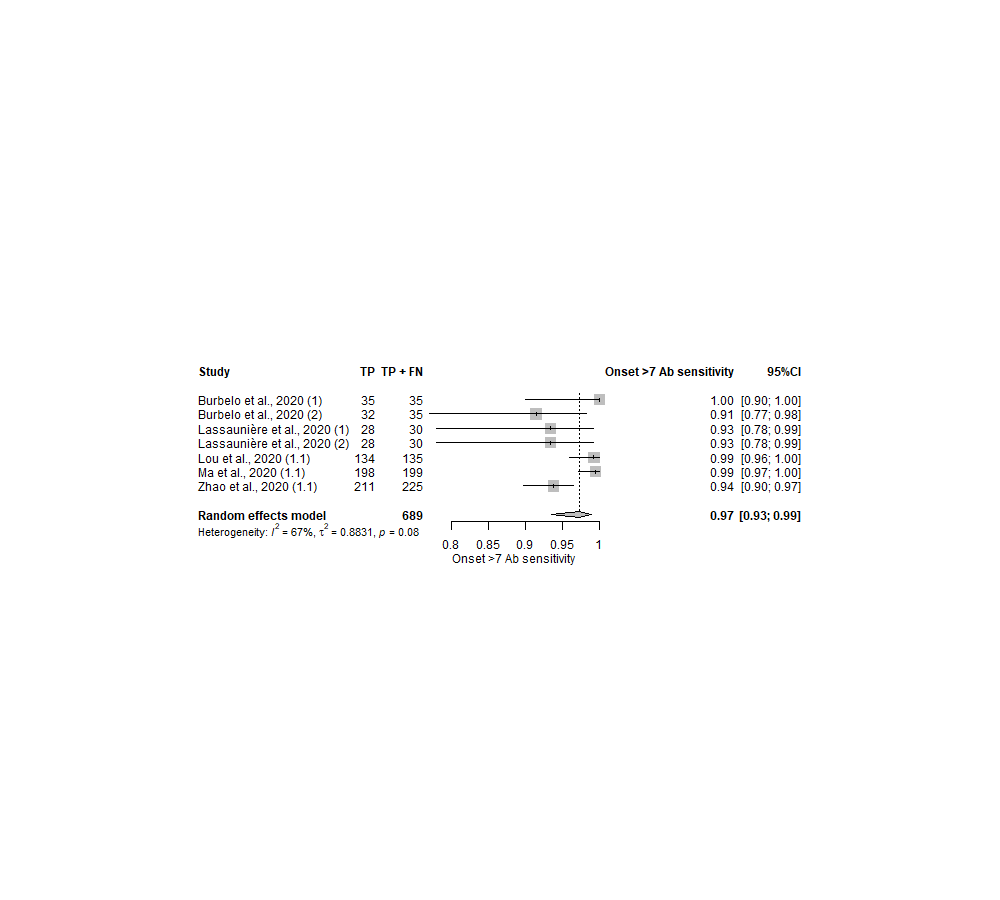


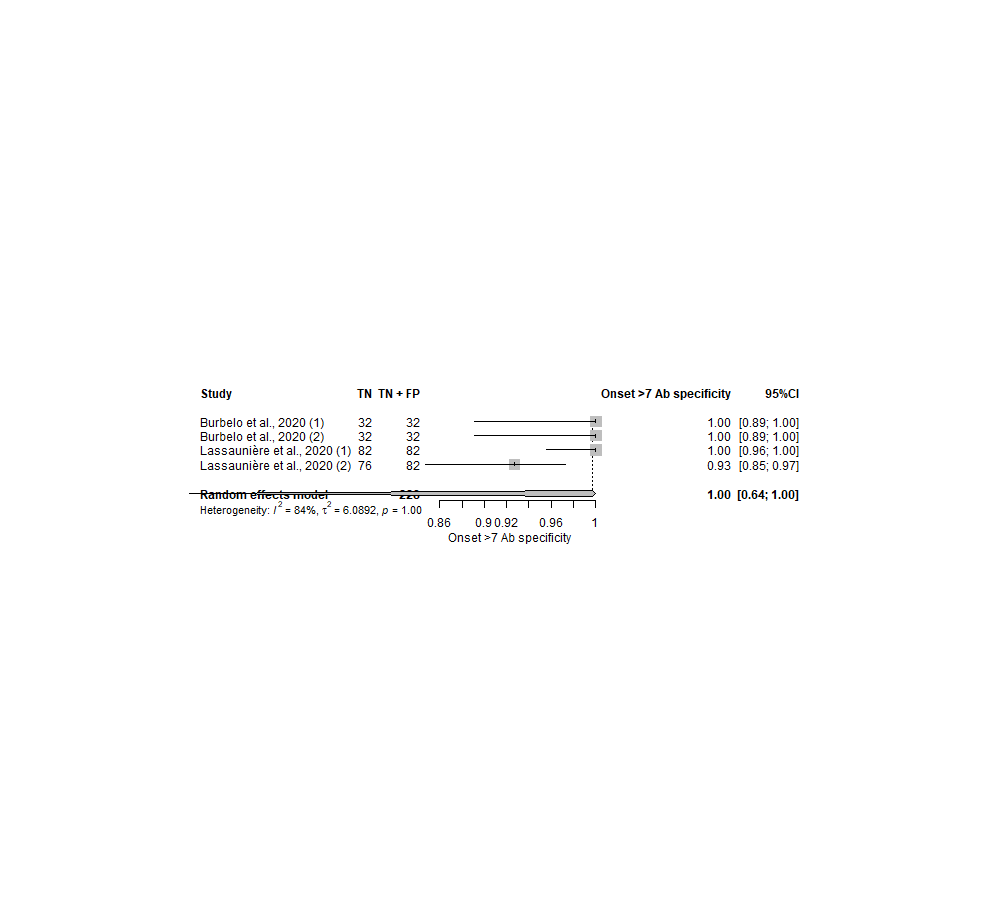


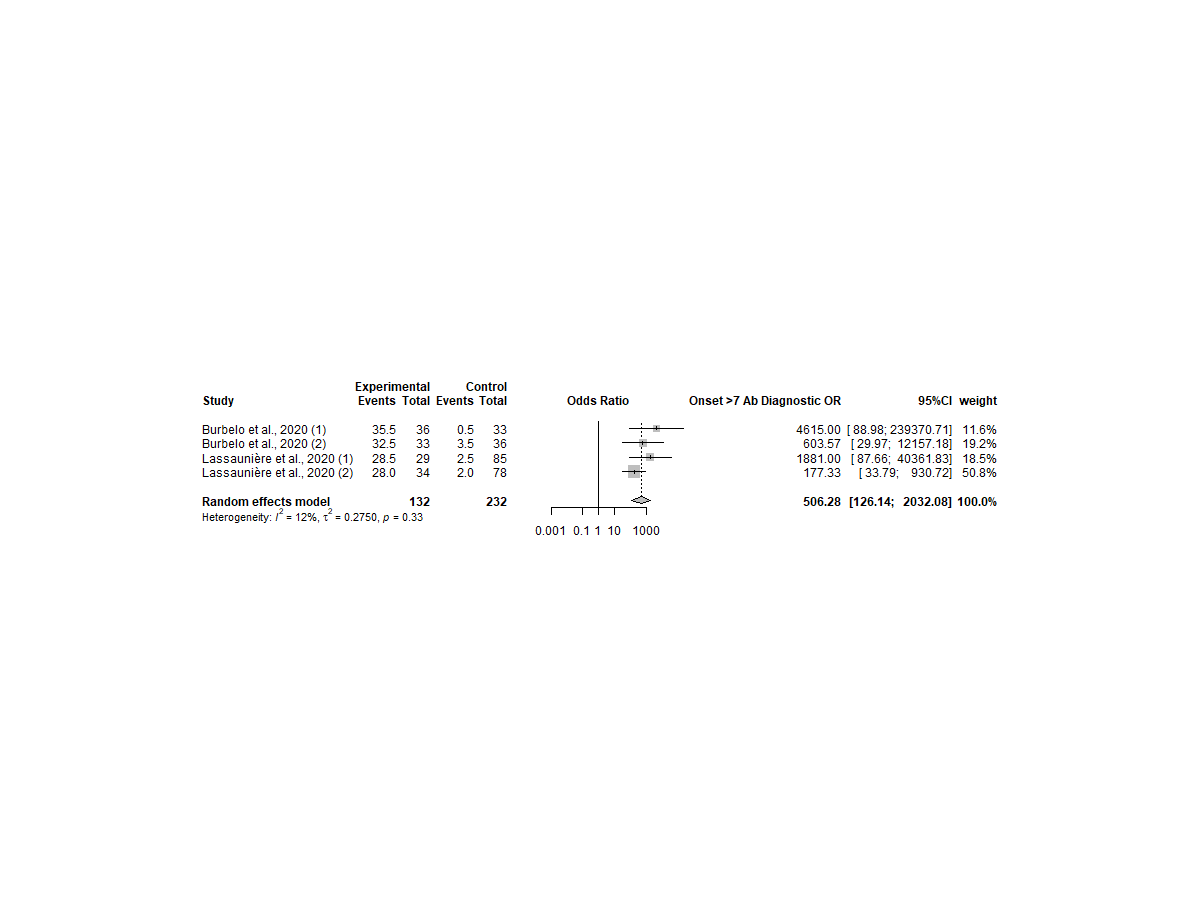


##
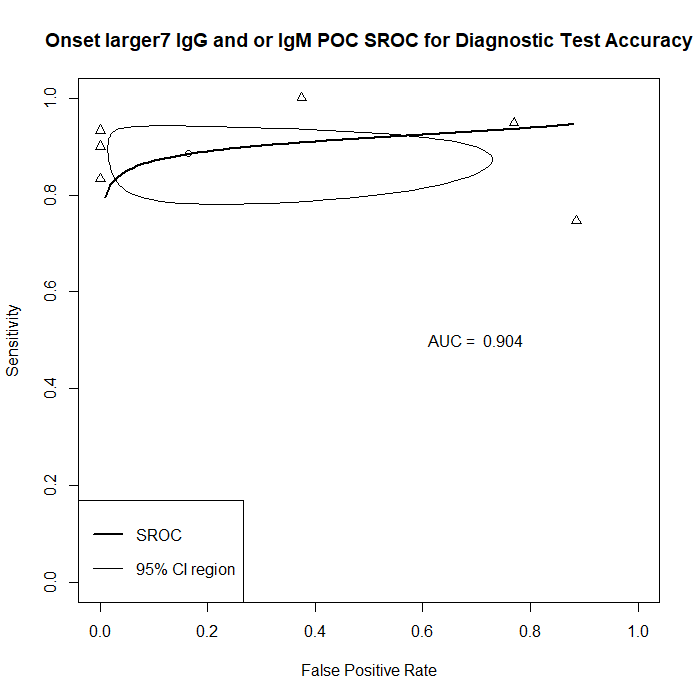
IgG and/or IgM (POC)


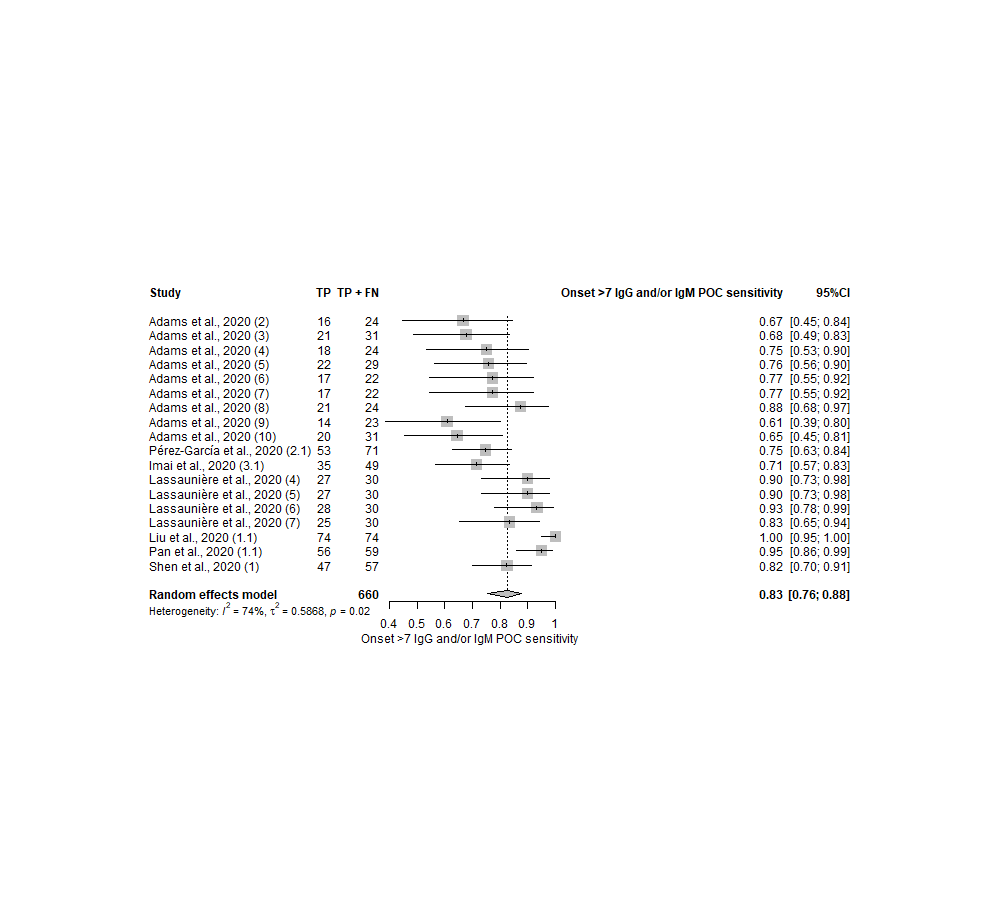


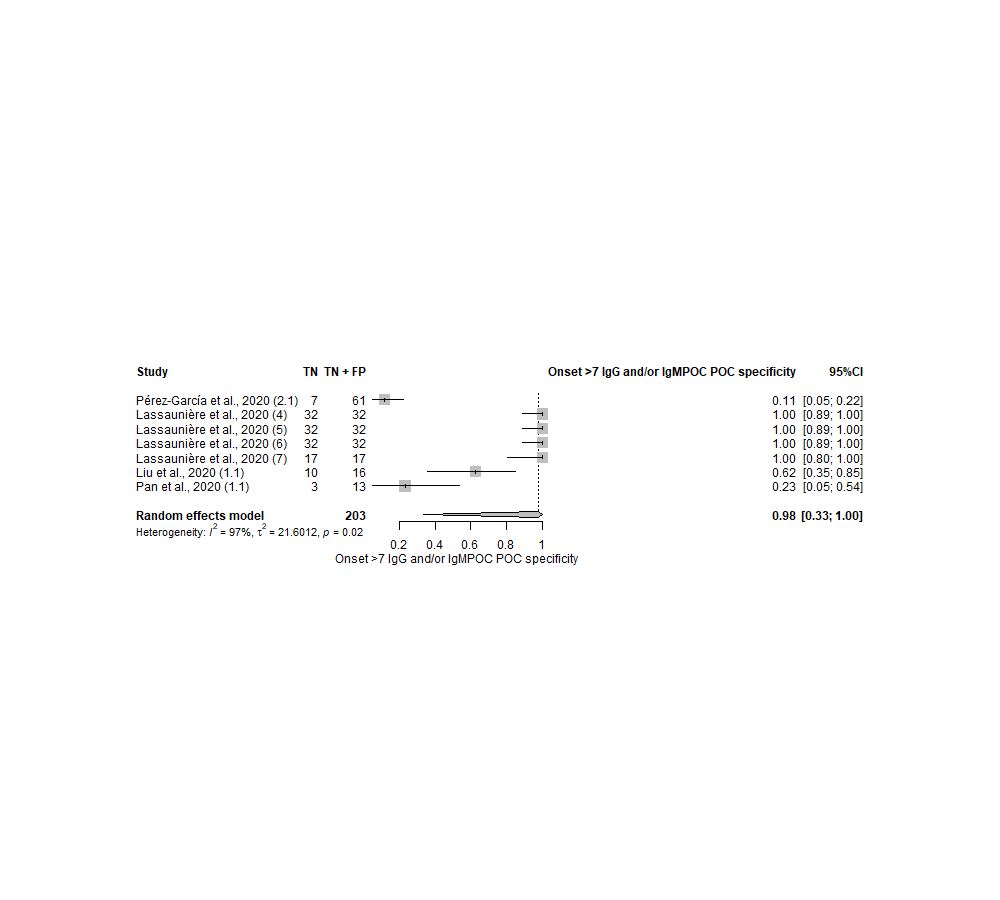


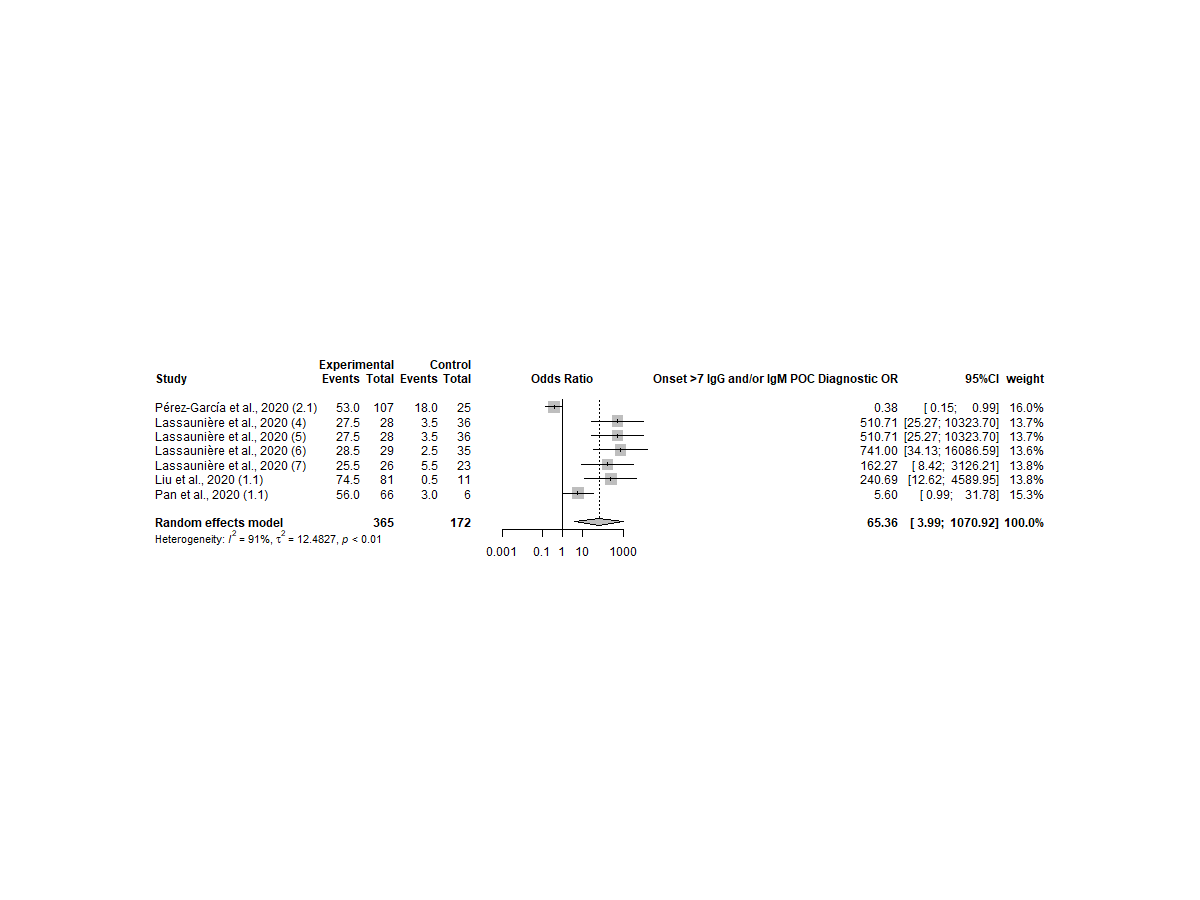


## IgG (POC)


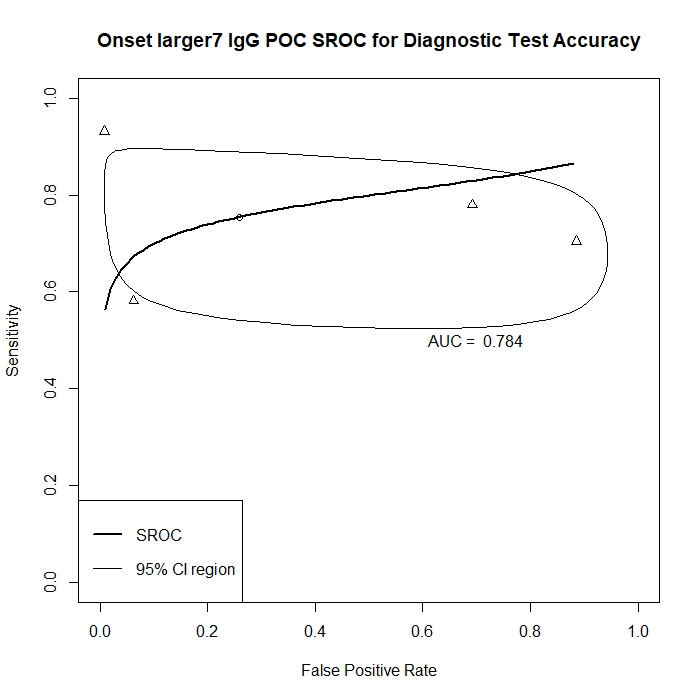

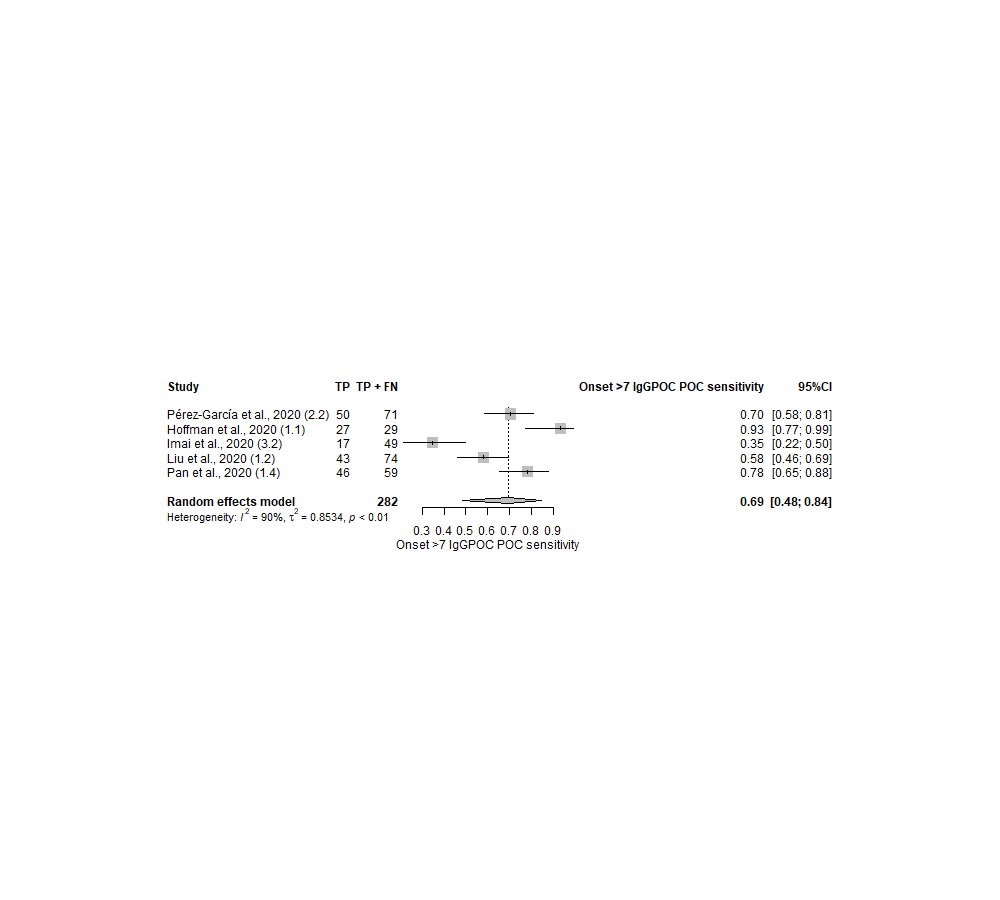


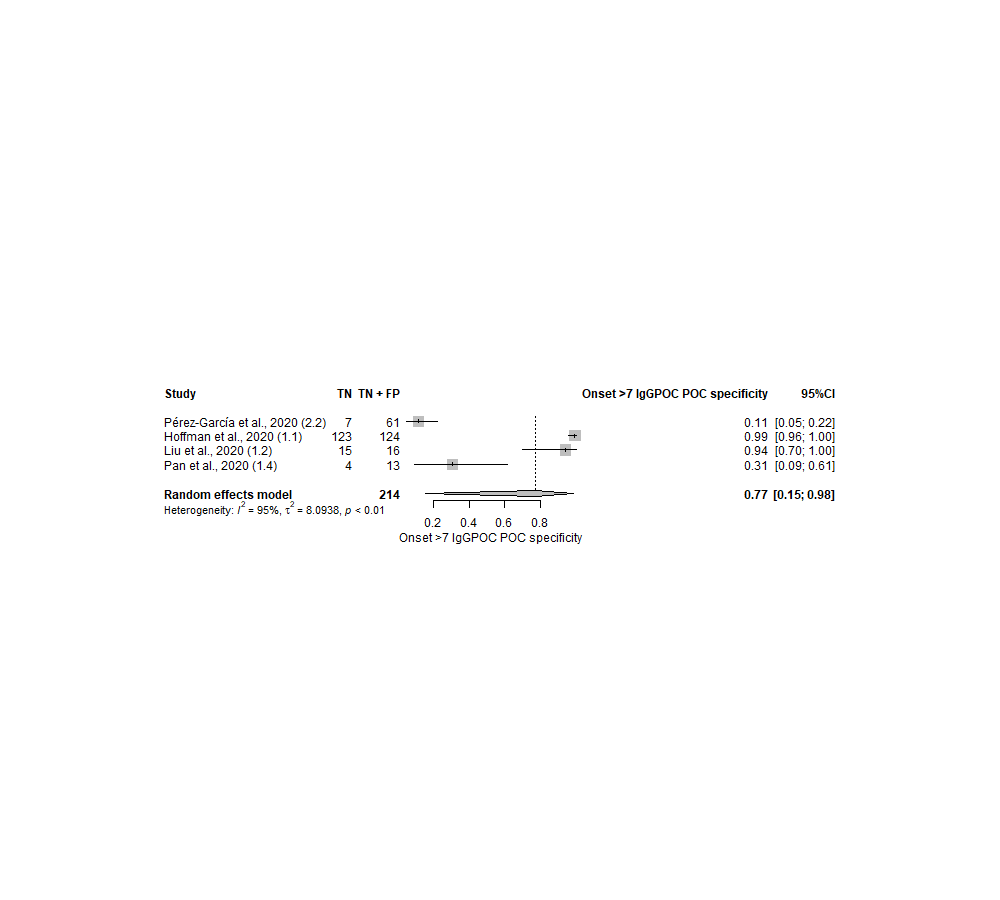


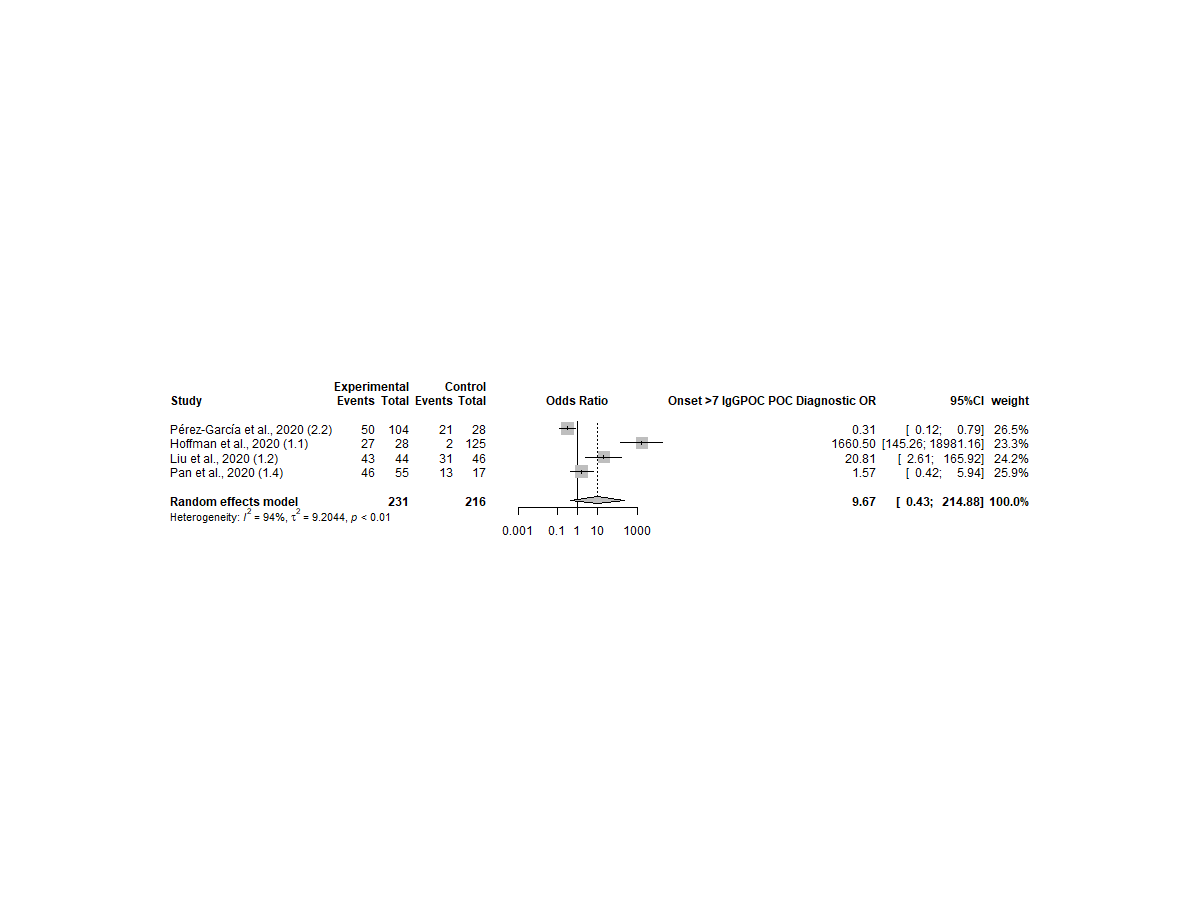


## IgM (POC)


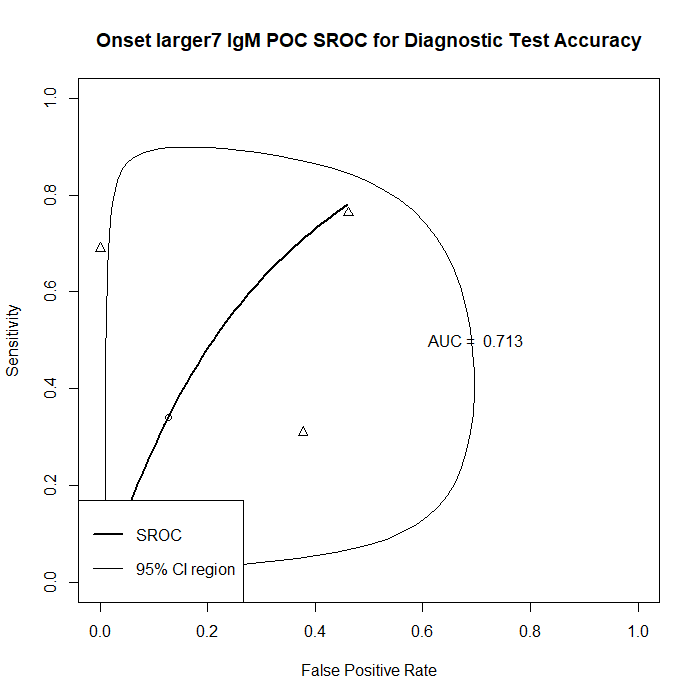

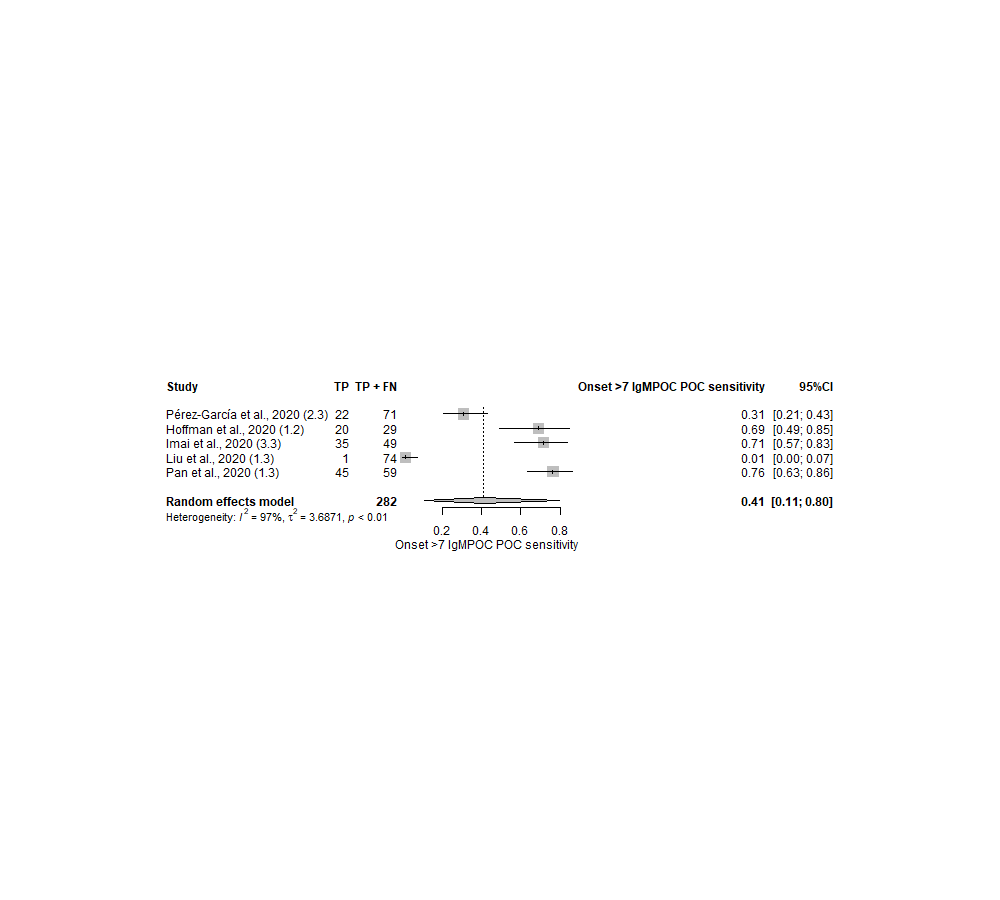

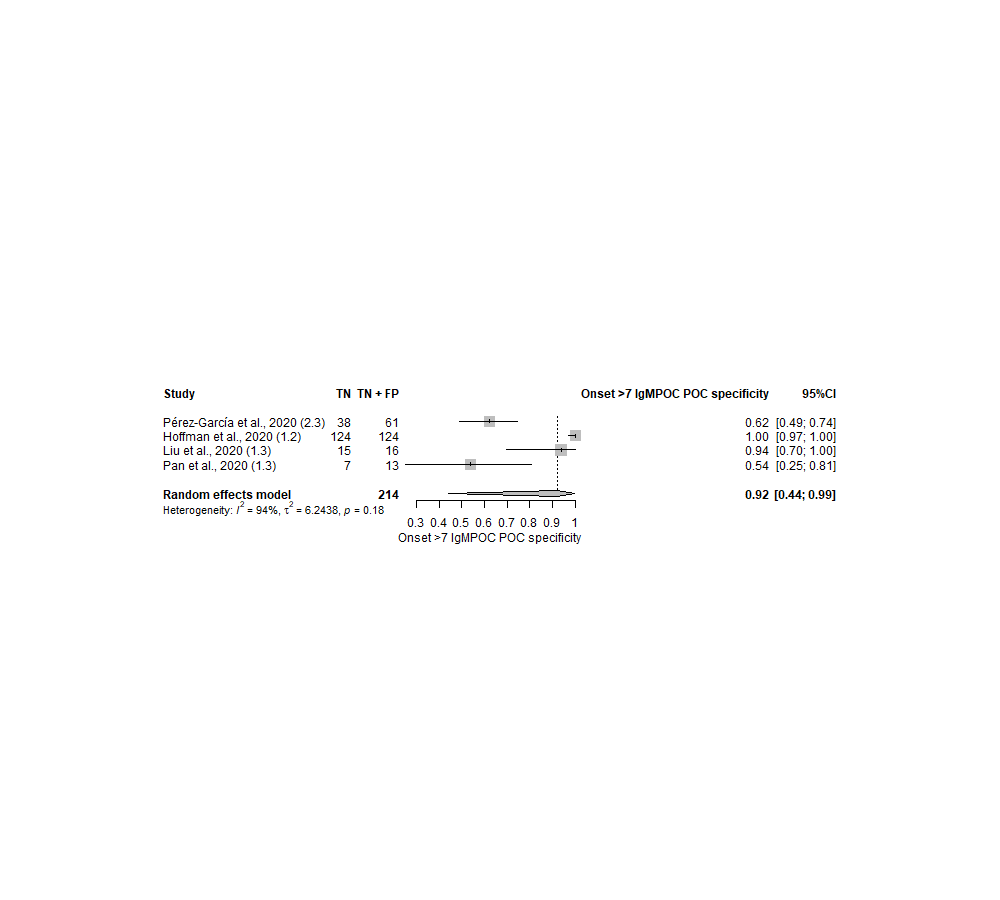


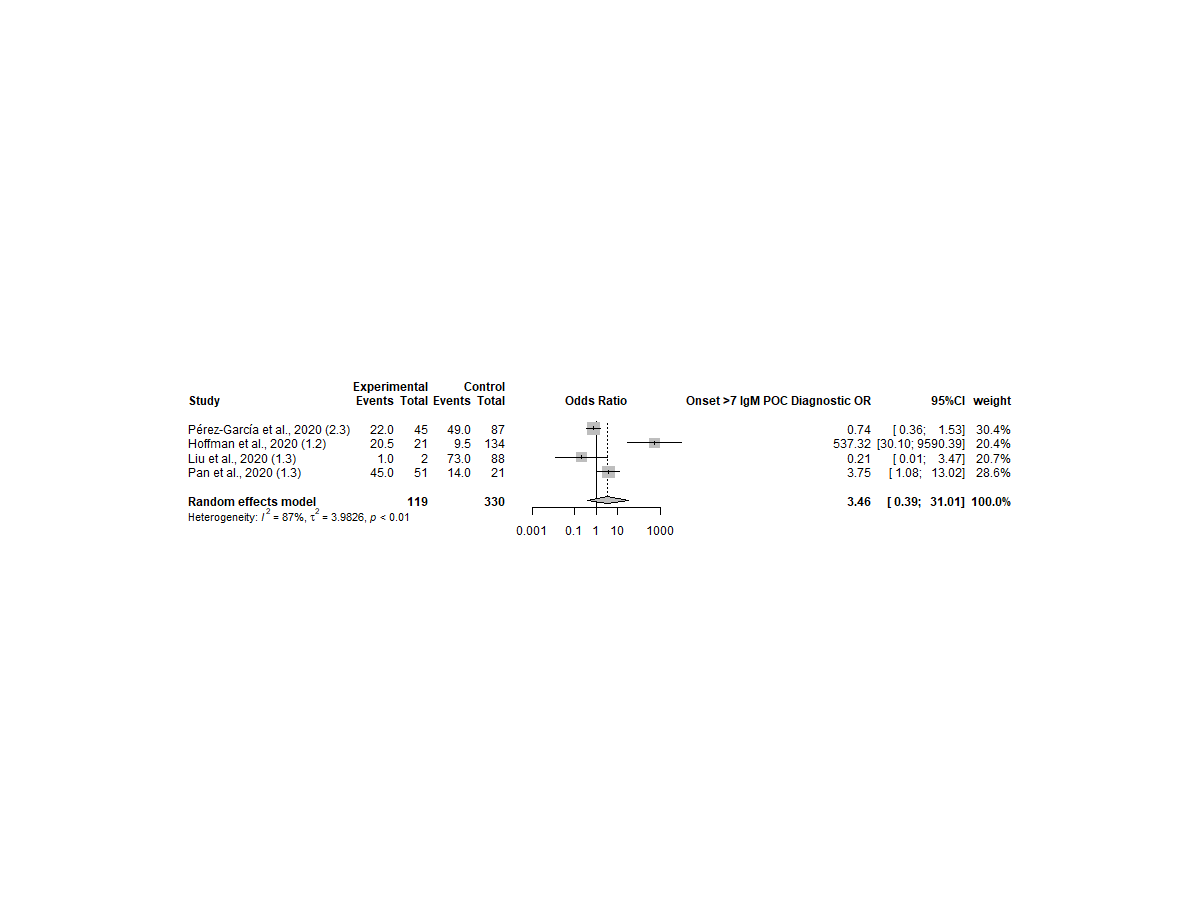


# **Figure S3: Serologic Test Early Phase (≤7) Forest Plot on Sensitivity and Specificity, SROC and Diagnostic OR for 1. IgG and/or IgM, 2. IgG, 3. IgM, 4. Ab, 5. IgG and/or IgM (POC), 6. IgG (POC) and 7. IgM (POC)**

## IgG and/or IgM


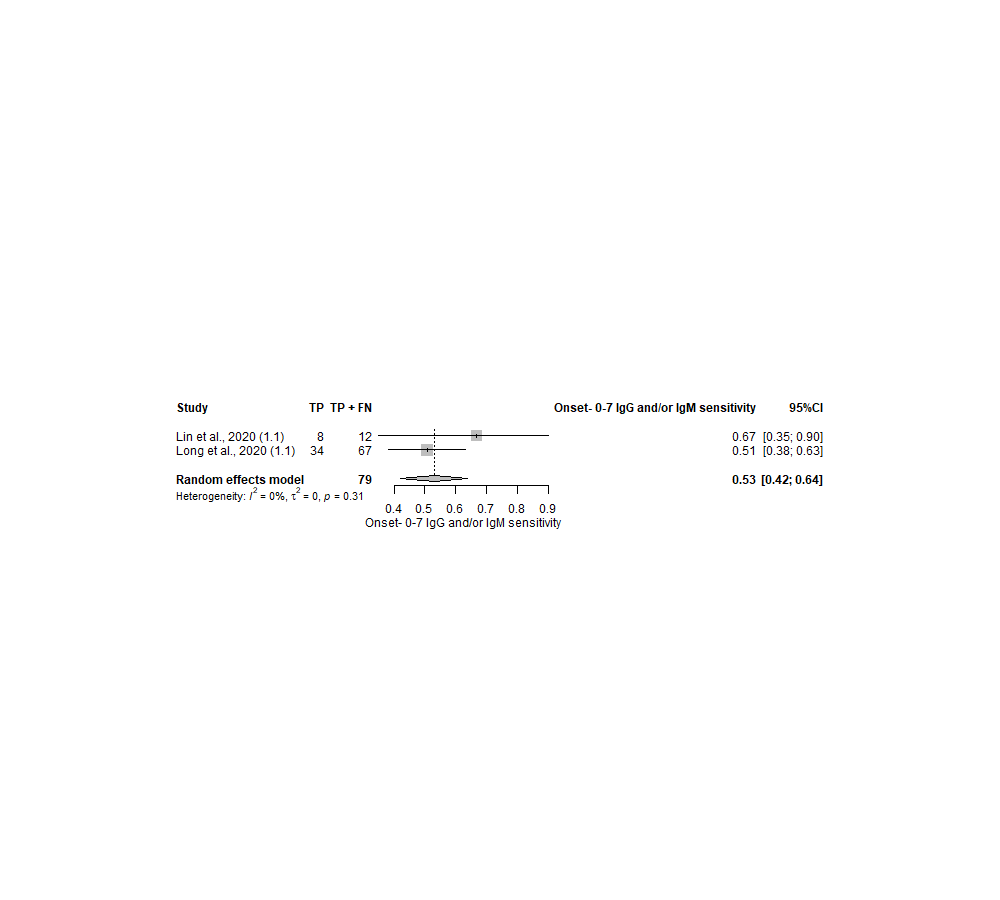


## IgG


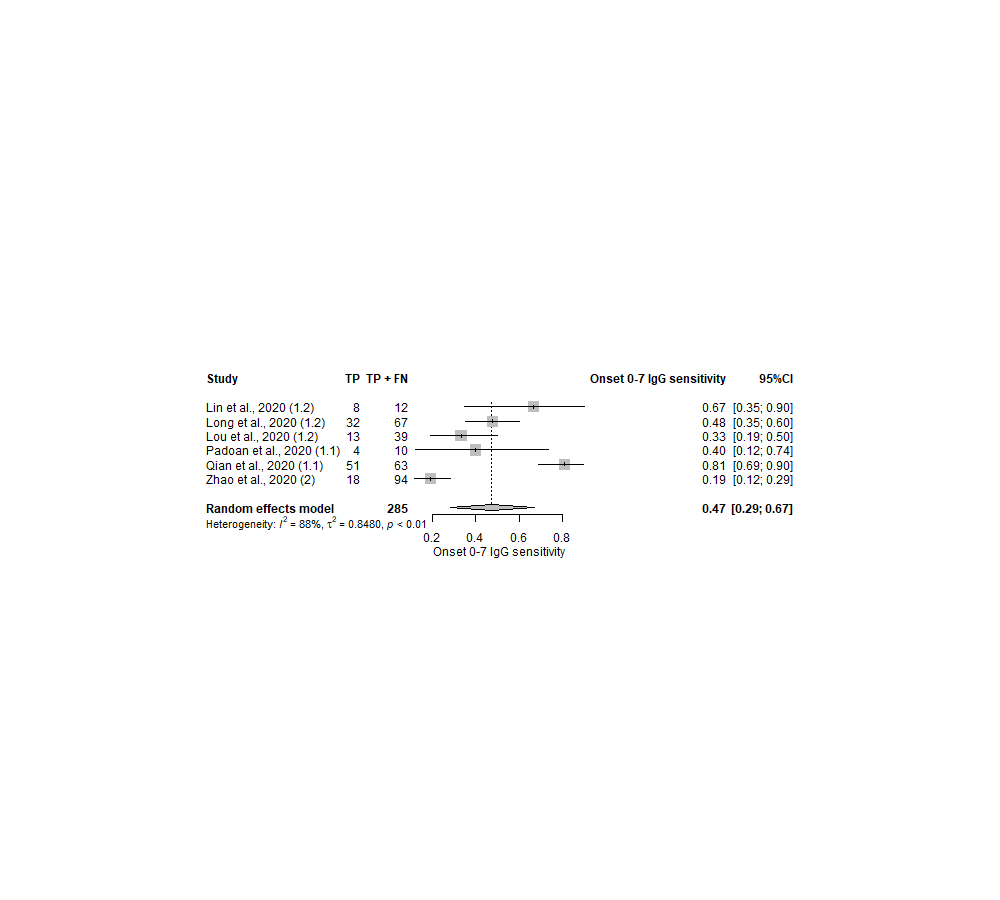


## IgM


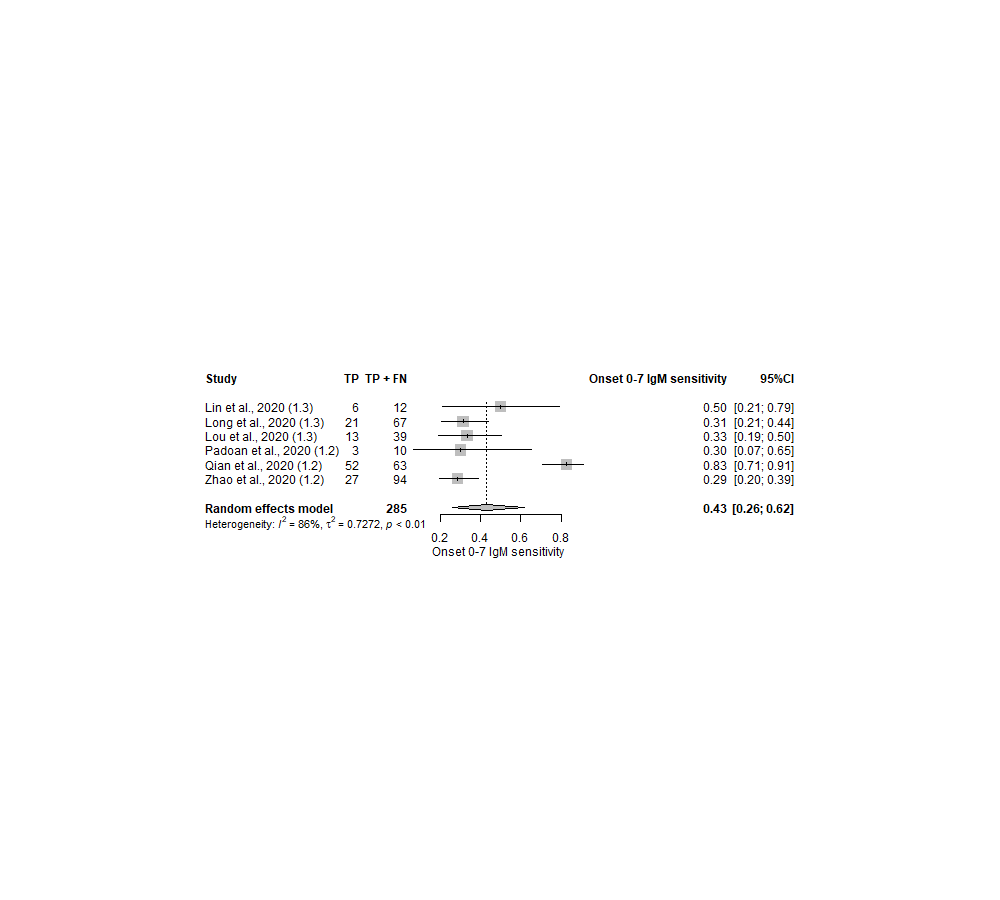


## Ab


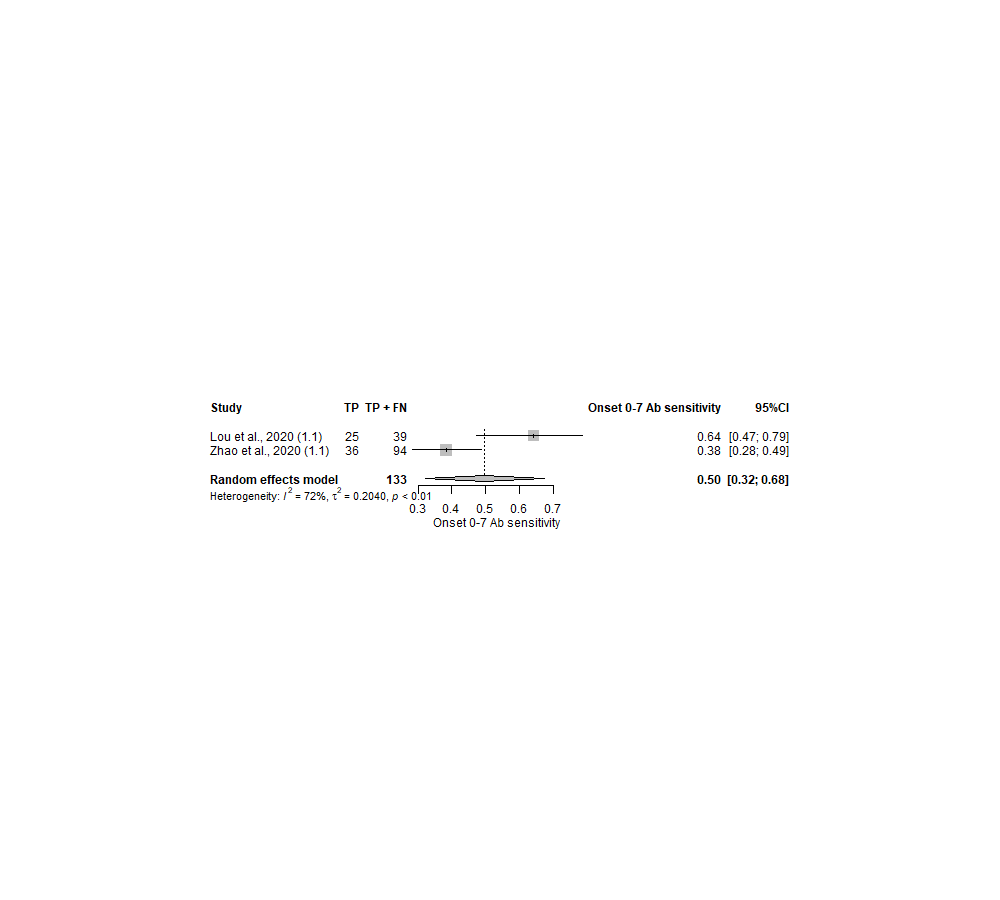


##
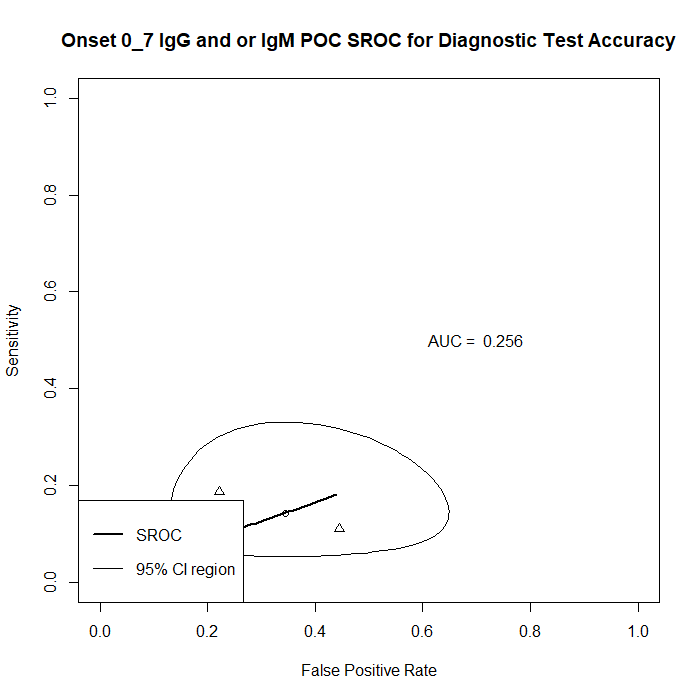
IgG and/or IgM (POC)


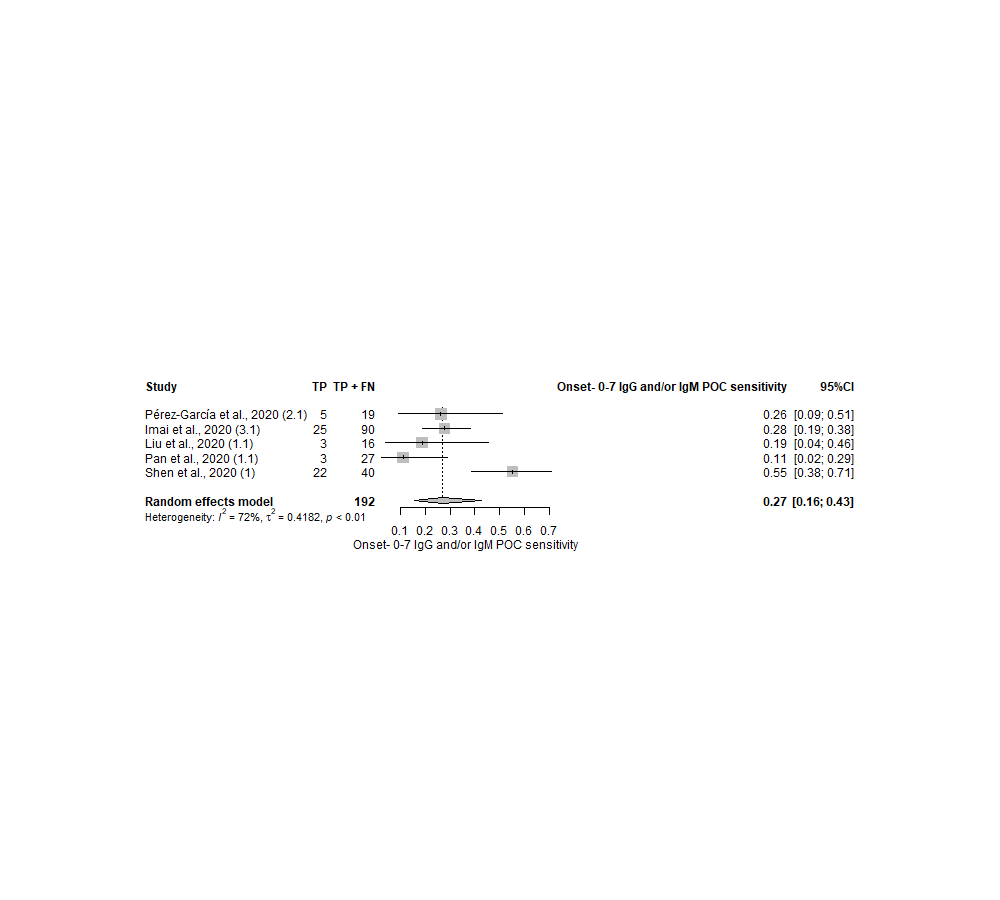


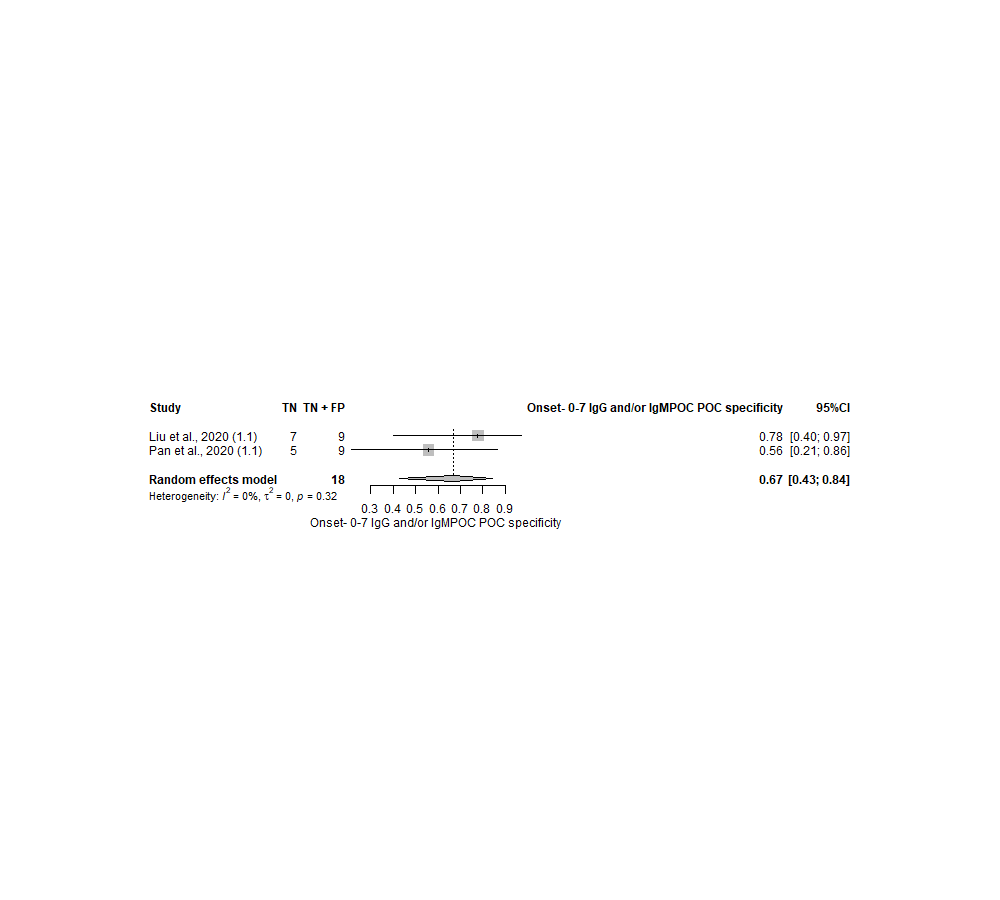


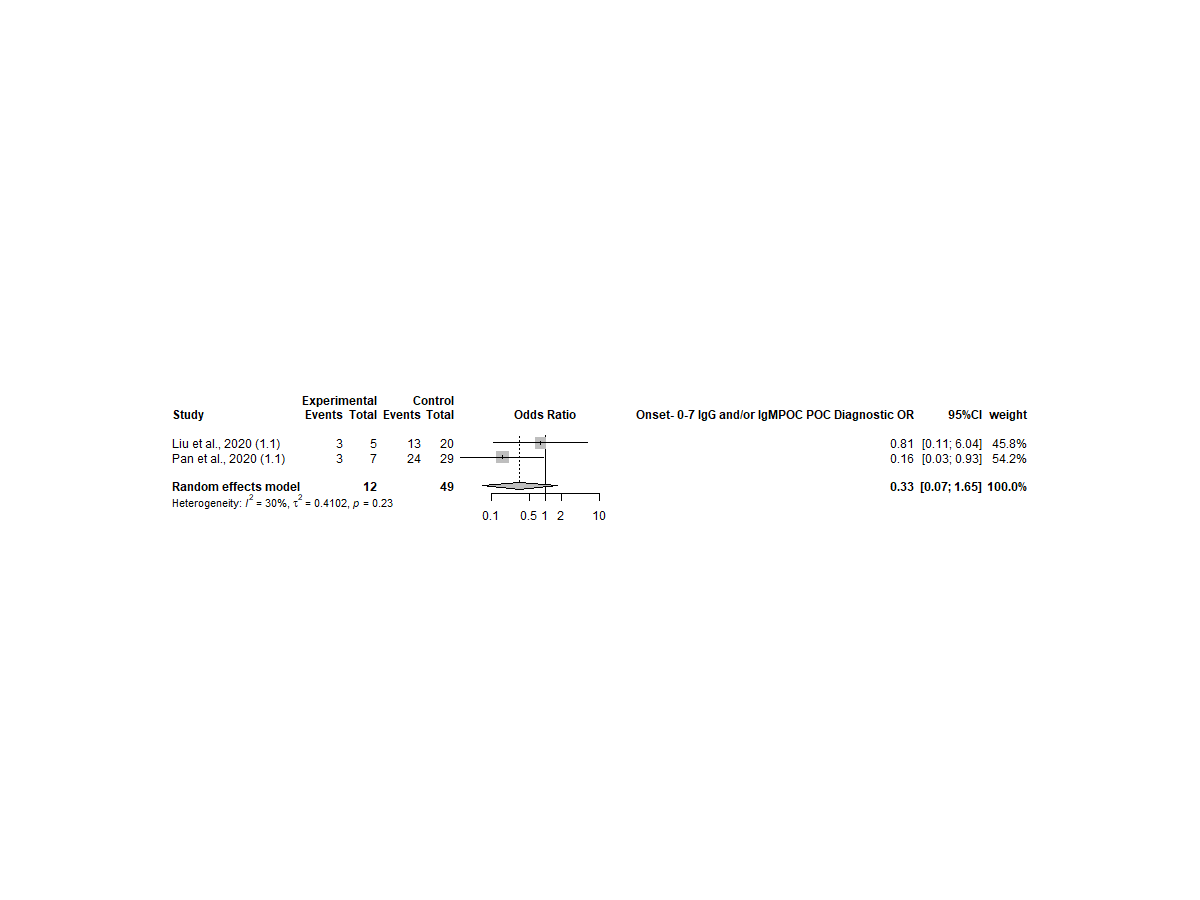


##
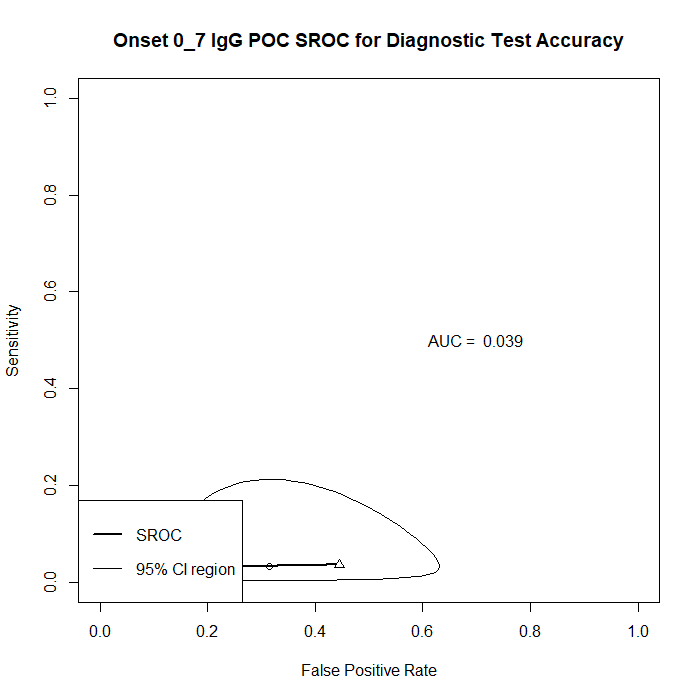
IgG (POC)


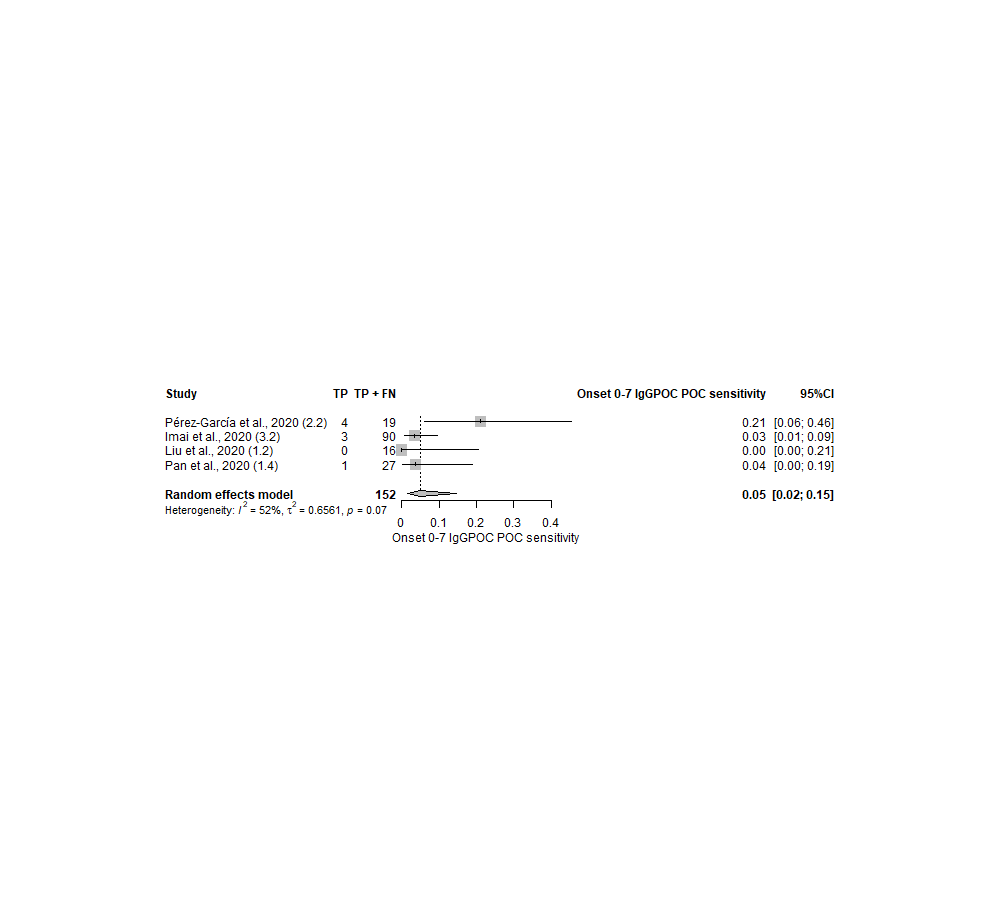


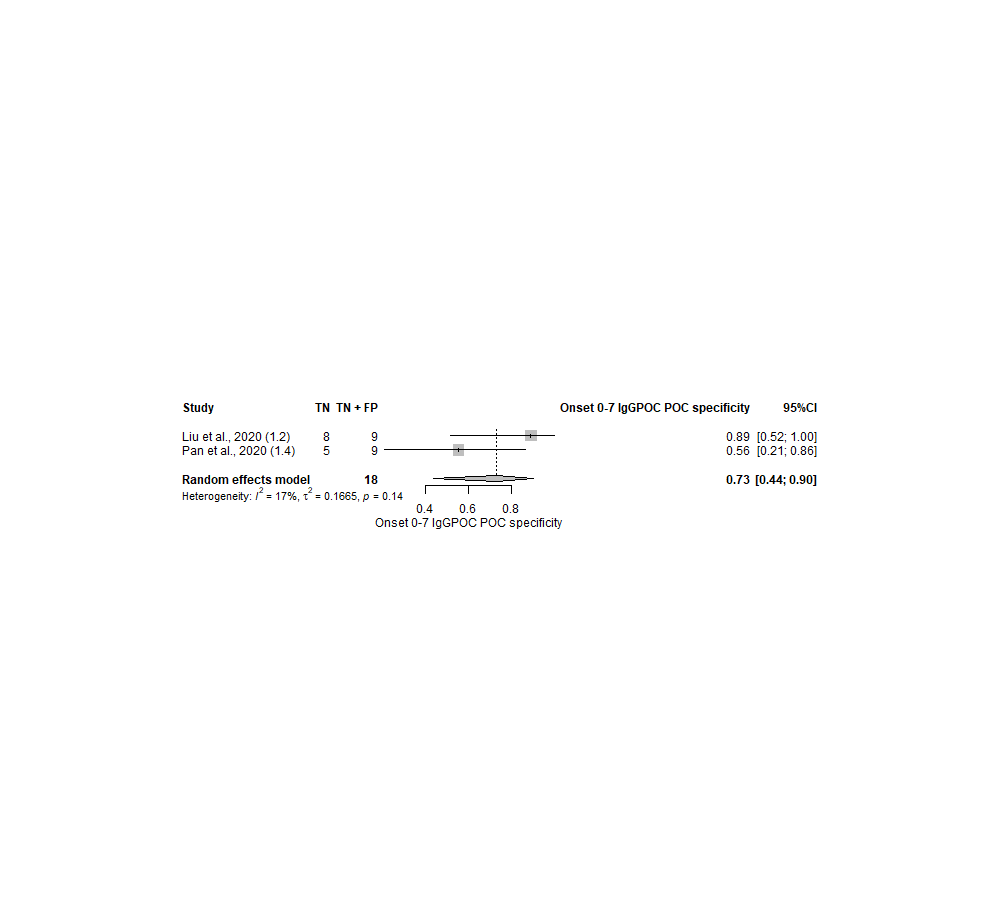


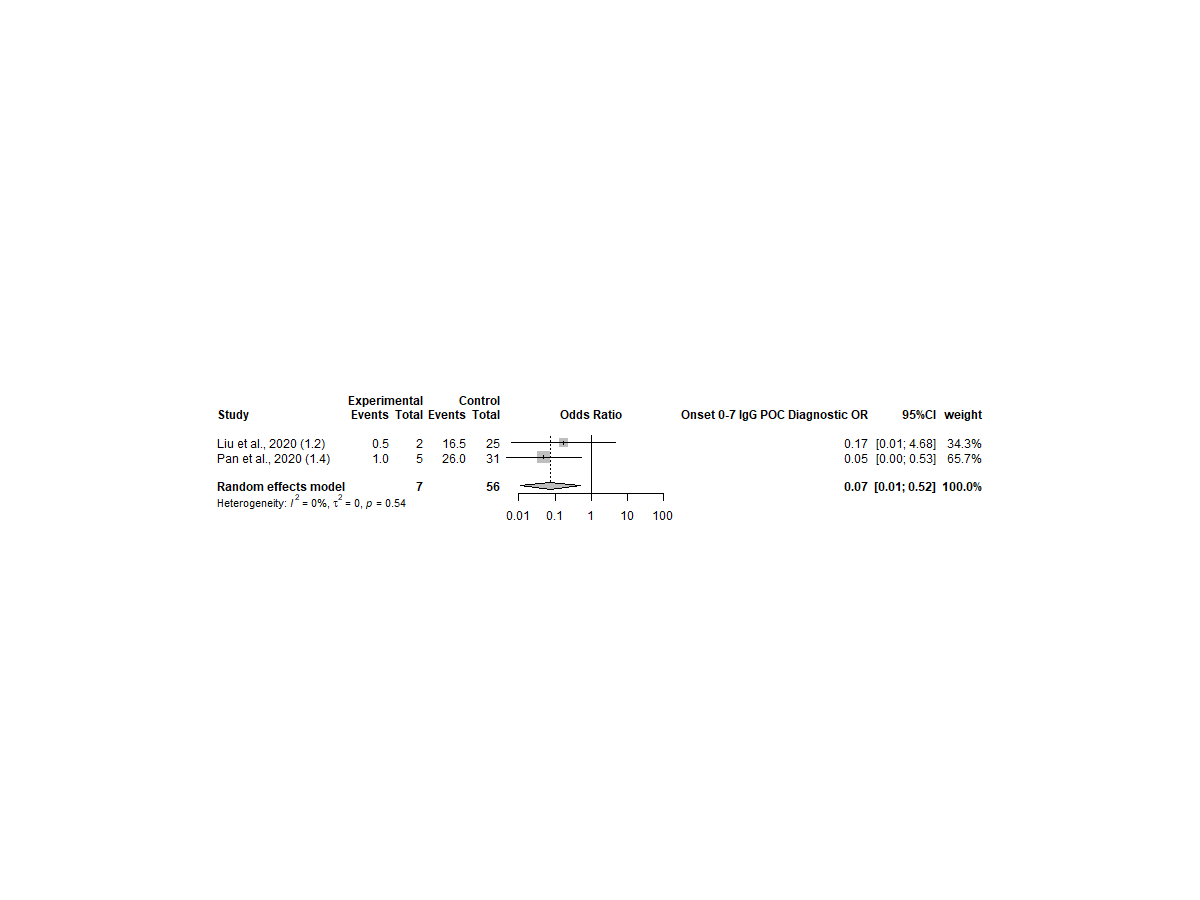


##
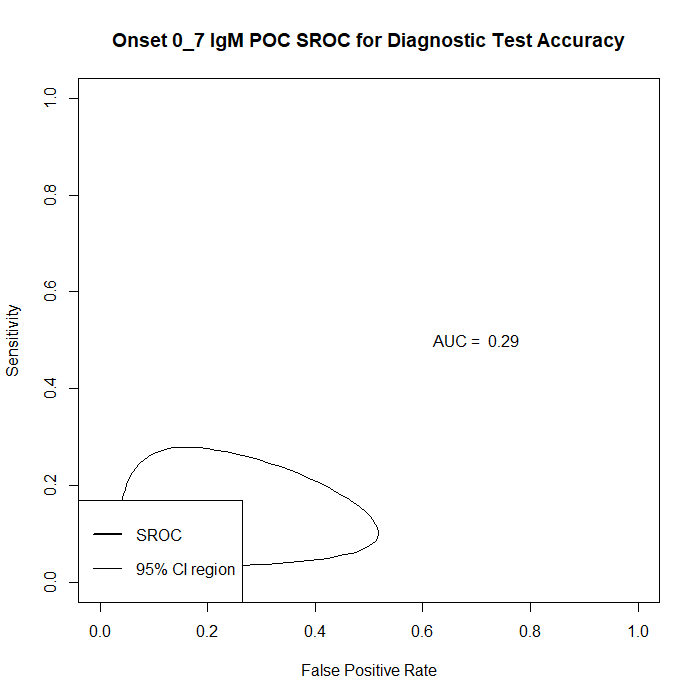
IgM (POC)


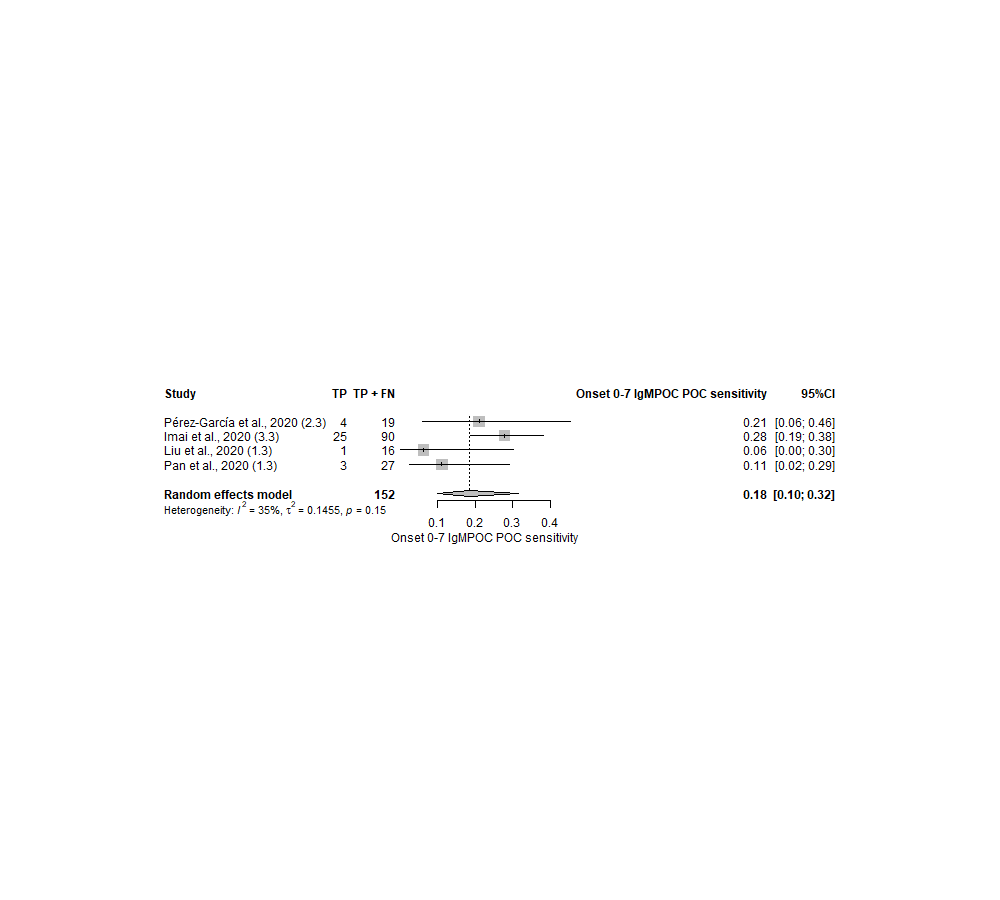


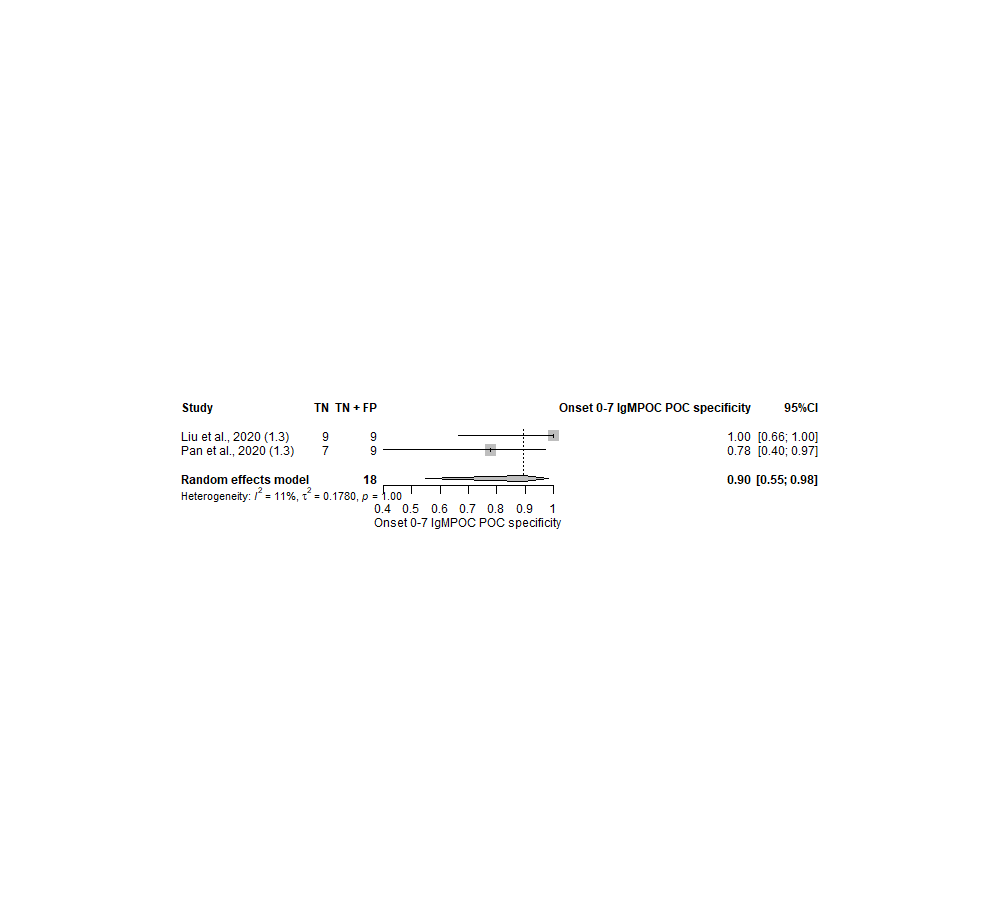


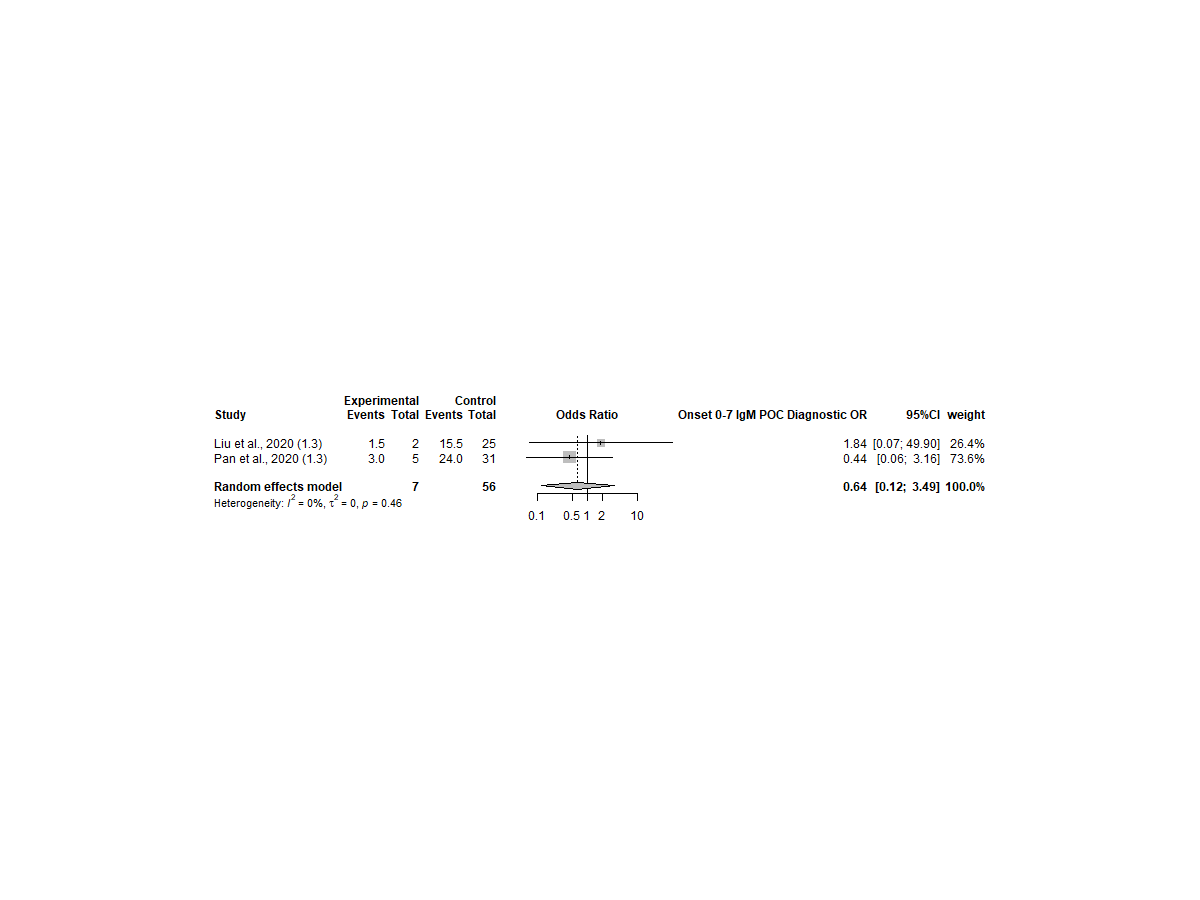


# **Figure S4: Diagnostic Test Accuracy between Symptomatic (Left) and Asymptomatic (Right) Patient Forest Plot on Sensitivity 1. NAAT, 2. Serology IgG and/or IgM, 3. Serology IgG and/or IgM 4. Serology IgG, 5. Serology IgM, 6. Serology IgG and/or IgM (POC), 7. Serology IgG (POC), 8. Serology IgM (POC) and 9. Imaging**

| 1. NAAT |  |
| --- | --- |
| 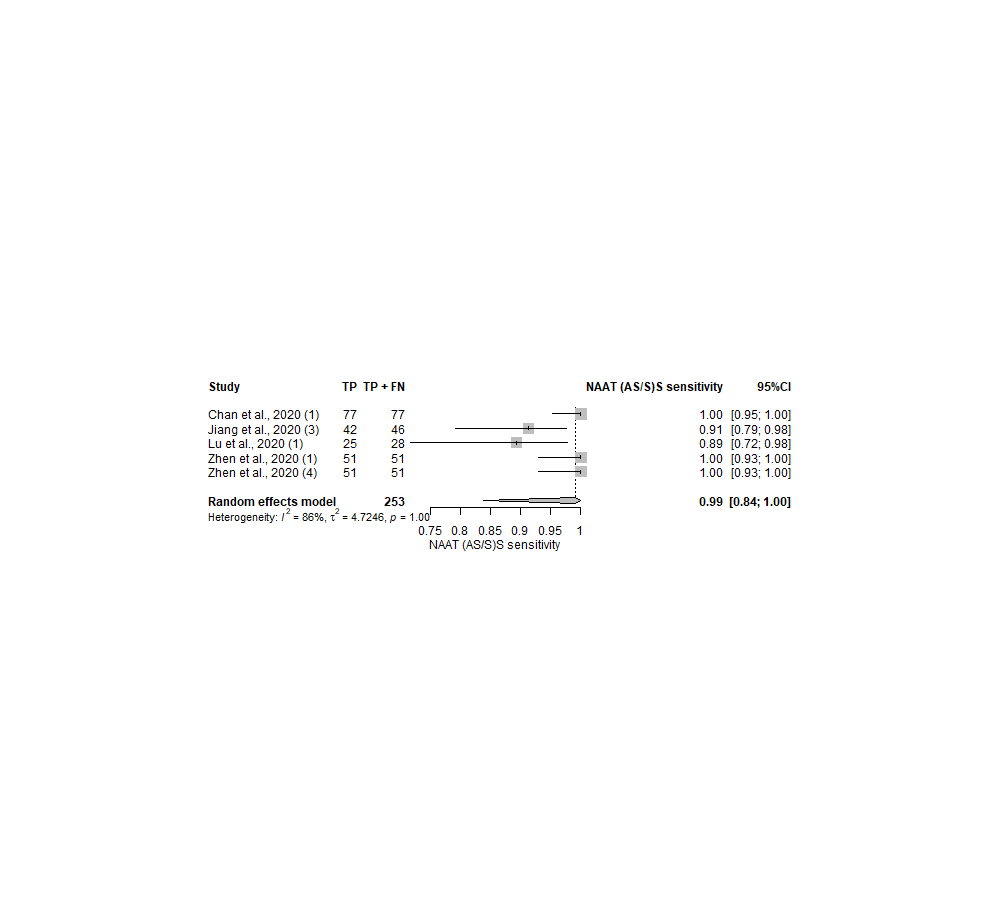 |  |
| 1. Serology IgG and/or IgM |  |
|  |  |
| 1. Serology IgG and IgM |  |
|  |  |
|  |  |
| 1. Serology IgG |  |
|  |  |
| 1. Serology IgM |  |
|  |  |
| 1. Serology (POC) IgG and/or IgM |  |
|  |  |
| 1. Serology (POC) IgG |  |
|  |  |
| 1. Serology (POC) IgM |  |
|  |  |
| 1. Imaging |  |
|  |  |

# **Figure S5: Geographical Forest Plot on Sensitivity and Specificity, SROC and Diagnostic OR for 1. NAAT, 2. NAAT (POC), 3. Serology (IgG and/or IgM), 4. Serology (IgG and IgM), 5. Serology (IgG), 6. Serology (IgM), 7. Serology (Ab), 8. Serology (IgA), 9. Serology (POC, IgG and/or IgM), 10. Serology (POC, IgG and IgM), 11. Serology (POC, IgG), 12. Serology (POC, IgM), 13. Imaging, 14. Imaging (AI), 15. Clinical Features and/or Laboratory Parameters and 16. Molecular + Imaging**

## NAAT

## NAAT (POC)

`

## Serology IgG and/or IgM

## Serology (IgG and IgM)

## Serology (IgG)

## Serology (IgM)

## Serology (Ab)

## Serology (IgA)

## Serology-POC (IgG and/or IgM)

## Serology – POC (IgG and IgM)

## Serology – POC (IgG)

## Serology – POC (IgM)

## Imaging

## Imaging (AI)

## Clinical Features and/or Laboratory Parameters

## Molecular + Imaging

# **Figure S6: Gene Target Forest Plot on Sensitivity and Specificity, SROC and Diagnostic OR for 1. S gene, 2. RdRp gene, 3. ORG1 gene, 4. N gene and 5. E gene.**

## S gene

## RdRp gene

## ORF1 gene

## N gene

## E gene

# **Figure S7: Specimen Site Forest Plot on Sensitivity and Specificity, SROC and Diagnostic OR for 1. Unspecified throat, 2. Nasopharyngeal, 3. Nasal and 4. Sputum**

## Unspecified Throat

## Nasopharyngeal

## Nasal

## Sputum

# **Figure S8: QUADAS Assessment**

# **Figure S9: Publication Bias for a. Serology (POC) IgG and/or IgM b. Serology(POC) IgG and IgM, c. Serology (POC) IgG, d. Serology (POC) IgM, e. Imaging, f. Imaging (AI), g. Serology IgG and/or IgM, h. Serology IgG and IgM, i. Serology IgG, j. Serology IgM, k. Serology Ab, l. NAAT, m. NAAT (POC), n. Clinical Features and/or Laboratory Parameter Model**

a

b

c

d

e

f

g

h

i

j

k

l

m

n

# **Tables**

## **Table S1: Eligibility Criteria based on PICOS tool**

| **Keywords** | “covid-19”, “2019-ncov”, “novel coronavirus”, “wuhan virus”, “SAR-CoV-2”, “diagnos*”, “polymerase chain reaction”, “serology”, “point of care”, “PCR”, “molecular”, “Nucleic-acid amplification tests”, “CT” “valid”, “sensitivity”, “specificity”, “evaluate” |
| --- | --- |
| **Eligibility Criteria** | |
| **Participants** | Cases are laboratory-confirmed COVID-19 patients. No restriction on control definitions, which may or may not be tested for COVID-19. Controls includes 1) laboratory-confirmed negative COVID-19 negative patients, 2) pre-pandemic controls without clinical suspicion of COVID-19, 3) controls with other confirmed infections or 4) healthy controls.  Includes:  i) Adults, Children, Elderly  ii) Any countries and races  iii) Asymptomatic, mild and severe (ICU, death, ECMO, HDU) cases. |
| **Exposure/Intervention** | Diagnostic Tests including Point-of-Care to identify SARS-CoV-2 infection at any point of the infection and any sites of sampling |
| **Comparator** | SARS-CoV-2 samples/patients confirmed by molecular test (e.g RT-PCR) or sequencing |
| **Outcome** | Clinical Sensitivity and Specificity  Other outcome if available: Negative Predictive Value, Positive Predictive Value, Agreement, Accuracy |
| **Language** | English |
| **Time- frame** | All publications from 1^st^ January 2020 until 30th April, 2020 |

## **Table S2: Database Search Terms**

| **Database** | **Search Terms** | |
| --- | --- | --- |
| Pubmed | (((covid-19) OR (2019-ncov)) OR (novel coronavirus)) OR (wuhan virus) OR (SAR-CoV-2)) AND ((diagnos*) OR (polymerase chain reaction) OR (serology) OR (point of care) OR (polymerase chain reaction[MeSH Terms]) OR (serology [MeSH Terms]) OR (PCR) OR (molecular) OR (Nucleic-acid amplification tests) OR (Computed tomography[MeSH Terms]) OR (CT)) AND ((validation study[MeSH Terms]) OR (VALID) OR (SENSITIVITY) OR (SPECIFICITY) OR (evaluate))) Filters: from 2020 - 2020 Sort by: Most Recent | |
| Embased | ('covid 19'/exp OR 'covid 19' OR '2019 ncov' OR (novel AND ('coronavirus'/exp OR coronavirus)) OR (wuhan AND ('virus'/exp OR virus)) OR 'sar cov 2') AND (diagnos* OR (('polymerase'/exp OR polymerase) AND chain AND ('reaction'/exp OR reaction)) OR 'serology'/exp OR serology OR (point AND of AND ('care'/exp OR care)) OR 'pcr'/exp OR pcr OR molecular OR (('nucleic acid'/exp OR 'nucleic acid') AND ('amplification'/exp OR amplification) AND tests) OR (computed AND tomography)) AND (valid OR 'sensitivity'/exp OR sensitivity OR 'specificity'/exp OR specificity OR evaluate) AND 2020:py | |
| Scopus | ( ( covid-19 )  OR  ( 2019-ncov )  OR  ( novel  AND  coronavirus )  OR  "wuhan virus"  OR  ( sar-cov-2 ) )  AND  ( ( diagnos* )  OR  ( polymerase  AND  chain  AND  reaction )  OR  ( serology )  OR  ( point  AND  of  AND  care )  OR  ( pcr )  OR  ( molecular )  OR  ( nucleic-acid  AND  amplification  AND  tests )  OR  ( computed  AND  tomography )  OR  ct )  AND  ( ( valid )  OR  ( sensitivity )  OR  ( specificity )  OR  ( evaluate ) )  AND  ( LIMIT-TO ( PUBYEAR ,  2020 ) ) | |
| Cochrane | #1 | Covid-19 |
|  | #2 | 2019 ncov |
|  | #3 | Novel coronavirus |
|  | #4 | Wuhan virus |
|  | #5 | SAR-CoV-2 |
|  | #6 | #1 OR #2 OR #3 OR #4 OR #5 |
|  | #7 | Diagnos* |
|  | #8 | MeSH descriptor:[Diagnosis] explode all trees |
|  | #9 | Point of care |
|  | #10 | MeSH descriptor:[Point-of-Care Testing] explode all trees |
|  | #11 | Polymerase chain reaction |
|  | #12 | MeSH descriptor:[Polymerase Chain Reaction] explode all trees |
|  | #13 | Serology |
|  | #14 | MeSH descriptor:[Serology] explode all trees |
|  | #15 | PCR |
|  | #16 | Molecular |
|  | #17 | Nucleic-acid amplification test |
|  | #18 | Computed tomography |
|  | #19 | MeSH descriptor:[Diagnostic Tests, Routine] explode all trees |
|  | #20 | #7 OR #8 OR #9 OR #10 OR #11 OR #12 OR #13 OR #14 OR #15 OR #16 OR #17 OR #18 #19 |
|  | #21 | Valid |
|  | #22 | MeSH descriptor:[Validation Study] explode all trees |
|  | #23 | Sensitivity |
|  | #24 | Specificity |
|  | #25 | Evaluate |
|  | #26 | MeSH descriptor:[Sensitivity and Specificity] explode all trees |
|  | #27 | #21 OR #22 OR #23 OR #24 OR #25 OR #26 |
|  | #28 | #6 AND #20 AND #27 |
| Medrvix and Biorvix | "serology "polymerase chain reaction" "Computed tomography" sensitivity specificity PCR CT" (match any words) and full text or abstract or title "covid-19 2019-ncov SARS-CoV-2 diagnos*" (match whole any) and posted between "01 Jan, 2020 and 30 Apr, 2020" | |

## **Table S3a: Summary of Patients’ Characteristic**

| **First Author [Reference]** | **Country of Study** | **Adult/Child; Age;** | **Male (%)** | **Severity** | **COVID-19**  **Reference Test** | |
| --- | --- | --- | --- | --- | --- | --- |
|  |  |  |  | **Cases** | **Cases** | **Controls** |
| Adams et al. (89) | United Kingdom | Adult;  NR | NR | 9 critical, 4 severe, 26 mild cases  Asymptomatic: 1 (2.5) | RT-PCR | Pre-pandemic:  healthy blood donors (n=60)  organ donors (n=50)  healthy volunteer (n=32) |
| Ai et al. (90) | China | Cases:  Adult: 106, Children: 2  Mean (SD) 50.3(17.43)  Range:  1yr 6mth - 90yrs | Case: 53 (49.1) | NR | RT-PCR | NA (no control) |
| Ai et al.  (91) | China | Total:  Adult, Children  Mean (SD): 51(15) Range: 2-95 | Total: 467 (46)  Case: 284 (47.3)  Non case: 183 (44.3) | NR | TaqMan One-Step RT-PCR (Shanghai Huirui Biotechnology Co., Ltd or Shanghai BioGerm Medical Biotechnology Co., Ltd,) | Negative PCR |
| Baek et al.  (92) | Korea | NR | NR | NR | qRT-PCR (iTaq Universal Probes One-Step Kit (Bio-Rad, Hercules, CA, USA)) | 85 nasal swab specimens collected during the outbreak, and previously confirmed in the hospital for other respiratory disease-causing viruses.  55 samples confirmed for other respiratory disease-causing viruses and collected prior to the outbreak  Not all controls were tested |
| Bai et al.  (93) # | China, United States | (In all test set)  Adult, <20 years old (unspecified);  Mean (SD)  Case: 46(16) Control: 62 (19) | 653 (In all sets) | Mild, Medium, Severe, Critical | Taqman One-Step RT-PCR (Shanghai BioGerm Medical Biotechnology Co., Ltd & Shanghai Huirui Biotechnology Co., Ltd); US Cases: COVID-19 RT-PCR test | Pneumonia patients reported from January 1, 2017 to December 30, 2019 from the radiology search engine, no mention of RT-PCR confirmation |
| Brinati et al. (94) # | Italy | Mean (SD) (of the 279 subjects)  61.3 (18.5) | NR | NR | RT-PCR | Negative RT-PCR |
| Broughton et al.  (95) | United State | NR | NR | NR | CDC SARS-CoV-2 qRT-PCR assay | Influenza Patients (n=5), Common human seasonal coronavirus infections (n=7) Negative qRT-PCR for COVID-19 but positive for another viral respiratory virus panel multiplex PCR testing or negative by all testing (n=30)  (All qRT-PCR negative) |
| Burbelo et al. (96) | United State | NIH controls: ND | NIH controls: ND | Asymptomatic: 0 (0) | CDC SARS-CoV-2 RT-PCR assay | Blood donors from 2018 (n=32) |
|  |  | UCSD cases:  Adult: 3, Child: 0;  Mean: 73  Range: 59-84 | UCSD: 2 (66) |  |  |  |
|  |  | UW cases:  Adult: 13, Child: 0;  Mean 66  Range: 43-95 | UW: 10 (77) |  |  |  |
|  |  | EH cases:  Adult: 13, Child: 0;  Mean: 59  Range: 19-88 | EH: 3 (30) |  |  |  |
|  |  | NIH cases:  Adult: 6, Child: 0  Mean: 45  Range: 22-67 | NIH: 5 (83) |  |  |  |
| Butt et al.  (97) |  | NR | NR | NR | rRT-PCR | Negative rRT-PCR |
| Cai et al.  (98) | China | Adult, Child;  Cases  Median (IQR): 48 (37-56)  Range: 0.66-84 | Cases: 151 (54.71) | NR | real time RT-PCR (DAAN Gene Co., Ltd.) | 167 sera from patients with infection with other pathogens |
| Caruso et al.  (99) | Italy | Total  Adult: 158, Child: 0;  Mean (SD): 57 (17)  Range: 18-89 | 83 (52.5) | Asymptomatic: 0 (0) | real time RT-PCR (Charitè, Berlin, Germany) | Negative RT-PCR |
| Cassaniti et al. (100) | Italy | Intensive Care Unit  Adult: 60, Child: 0;  Median (IQR)  Cases: 73.5 (38-86)  Range: 38-86  Controls: 38.5 (25-69)  Range: 25-69 | 36 (60) | NR | real time RT-PCR | Healthy Volunteer (n=30) including 10 subjects with past coronavirus infection.  Negative Covid-19 RT-PCR |
|  |  | Emergency Room  Adult: 50, Child: 0; Median: 61.5 Range: 33-97 | 34 (68) | Asymptomatic: 0 (0) |  | Negative Covid-19 real time RT-PCR |
| Castiglioni et al.  (101) | Italy | NR | NR | Asymptomatic: 0 (0) | RT-PCR | Negative RT-PCR |
| Chan et al.  (17) | Hong Kong | Total  Adult: 37, Child: 0;  Case  Median: 63  Range: 37-75 | Case: 8 (53.3) | Critical: 3  Asymptomatic: 0 (0) | RT-PCR | 22 archived nasopharyngeal aspirates/ swabs and throat swabs that were positive for other respiratory pathogens |
| Chen et al. (102) | China | Testing Set  Adult; Median (IQR)  Case: 42 (34.5-65.5)  Control:47 (34.5-54.5) | 26 (61.9)  Case: 7 (63.6)  Control: 19 (61.29) | NR | Fluorescent RT-PCR (Shanghai Geneodx Biotechnology Co., Ltd.) | 31 controls of other disease (unclear if tested) |
| Chen et al. (103) # | China | Validation cohort only (Total)  Mean (SD): 41.08 (21.07) | Validation cohort only  23 (60.5) | Asymptomatic: NA | RT-PCR and Next Generation Sequencing | Negative RT-PCR |
| Chen et al.  (104) | China | NR | NR | NR | RT-PCR | Negative RT-PCR |
| Diao et al. (105) # | China | NR | NR | NR | RT-PCR; RT-PCR (CT ≤30); RT-PCR (CT ≤40) | Negative nucleic acid |
| Döhla et al.  (106) | Germany | Median (IQR): 46 (28-72) | 25 (51) | NR | RT-qPCR (Altona Diagnostics) | RT-qPCR-negative |
| Dong et al.  (107) | China | NR | NR | Asymptomatic: At least 9 of 126 patients | RT-qPCR (H&R Shanghai Huirui Biotechnology Co., Ltd, BioGerm from Shanghai BioGerm Medical Biotechnology, Daan from Daan Gene Co., Ltd) | Negative RT-qPCR |
| Freeman et al. (108) # | United State | NR | NR | Asymptomatic: 0/99 cases | RT-PCR | Healthy adults between 2016-2019 (n=377), suspected hantavirus patients between 2016-2019 (n=101), suspected HIV patients between 2011-2012 (n=21), suspected hepatitis B virus patients between 2011-2012 (n=10), HCV positive patients between 2011-2012 (n=10) |
| Fu et al. (109) | China | Adult | NR | NR | RT-PCR | 52 cases of non-COVID-19 viral pneumonia, 53 cases of bacterial 254 pneumonia, 54 cases of pulmonary tuberculosis, 100 cases of normal lung  (unclear if tested) |
| Gaeta et al.  (110) | Italy | Adult: 72;  Mean: 68.8  Range: 43-100 | 47 (65.3) | Almost severe (34), severe (29) and critical (9)  Asymptomatic: 0 | RT-PCR | NA (no control) |
| Perez-Garcia et al.  (111) | Spain | Group 1  Median (IQR): 50 (33-65) | 55 (55) | Asymptomatic: 0 | VIASURE SARS-CoV-2 Real Time PCR Detection Kit (Certest Biotech,  Zaragoza, Spain) and Allplex 2019-nCoV assay (Seegene, Seoul, South  Korea) | Patients whose serum sample taken for other serologic studies, from September 1 to November 30, 2019 (before the first cases of COVID-19 were reported) (n=100) |
|  |  | Group 2  Median (IQR): 64 (55-79) | 52 (57.8) | Mild (17), Non-severe pneumonia (87), Severe pneumonia (40), Critical (7)  Asymptomatic: 0 |  | NA (no control) |
|  |  | Group 3  Median (IQR): 67 (57-73) | 45 (73.8) |  |  | Negative Covid-19 Real Time PCR |
| Gietema et al.  (112) | Netherland | Adult; Median (IQR): 66 (55-76) | 113 (58.5) | Asymptomatic: 0 | RT-PCR | Negative PCR |
| Guo et al.  (113) | China | Family-clustered: Adult: 4, Children: 2  Mean: 35 | Family-clustered: 3 (50) | Asymptomatic: 0 | Deep sequencing or qPCR | Negative PCR |
|  |  | Confirm/Probable cases: NR | NR | Severe: 28, mild to moderate: 54 Asymptomatic: 0 |  | qPCR negative with typical manifestation |
|  |  | Negative control: NR | NR |  |  | Adults with acute lower respiratory tract infections in 2018 (n=135), Health individuals undergoing regular health checkup in 2018-2019 (n=150) |
| Hirotsu et al.  (114) | Japan | NR | NR | NR | RT-PCR, NIID recommended single-quencher probes | Negative PCR |
| Hoffman et al.  (115) | Sweden | Adult: at least 80; Children: at least 20 | NR | Asymptomatic: 0 | PCR | Healthy volunteers, without any known history of SARS-CoV-2 infection/COVID-19 (n=24)  Anonymous blood donor sera from healthy adults (n = 80) and serum samples from babies (6–12 months) collected before or during 2018 (n=20) |
| Hou et al.  (116) | China | NR | NR | NR | mNGS | nCoV-/hCoV+ cases (n=17) and healthy subjects (n=36)  (Unclear if tested)  suspected case with negative mNGS (n=9) |
| Huang et al.  (117) | China | NR | NR | NR | RT-qPCR (Shanghai ZJ Bio-Tech, China) | Negative RT-qPCR |
| Imai et al. (118) | Japan | Adult  Median (IQR)  Case: 67 (45-74)  Range: 20-93 | Cases: 64 (57.1) | Asymptomatic: 38 (33.9%) | RT-qPCR | Serum samples from patients from April to October 2019, before SARS-COV2 was first reported in China |
| Infantino et al. (119) # | Italy | Mean (SD)  Case:  59 (23)  Control:  Pre-Covid 19: 49 (17)  Blood donor: 44(11) | Cases: 26 (42.6)  Control: 21 (32.8) | mild to moderate: 30  Severe: 31  Asymptomatic: 0 | RT-PCR | Pre‐COVID‐19 (2018‐2019) disease control group of 44 patients who had rheumatic diseases (n = 31) and infectious diseases (n = 13). Twenty blood donors from the COVID 19 era (winter 2019) |
| Jia et al.  (120) | China | NR | NR | Asymptomatic: 0 | NAT (DAAN, Sansure Biotech, BGI, ShangHai ZJ Biotech, Geneodx,Biogerm) | Negative nucleic acid |
| Jiang et al.  (121) | China | Children and Adult | Of 568 subjects: 334 (58.8) | Of 568 subjects:  severe:14  mild: 73  Asymptomatic: NR | rRT-PCR | Negative RT-PCR |
| Jiang et al. (122) | China | NR | NR | Asymptomatic: 1 | qRT-PCR (Shanghai BioGerm Medical  Biotechnology Co. Ltd., DAAN Gene Co., Ltd) | Negative NMPA RT-PCR |
| Jin et al.  (123) | China | NR | 854 (53.7) | NR | RT-PCR | Test Cohort:  1,229 non-pneumonia subjects (healthy volunteers from Wuhan; PCR negative), 668 CAP [from pre-COVID period], 42 influenza A/B cases [from pre-COVID period]  CC-CCII:  425 non-pneumonia subjects, 482 CAP. |
| Jin et al.  (124) | China | NR | NR | NR | NAT | 21 healthy cases,107 cases with other known lung diseases with CT imaging features similar to COVID-19 to some extent (lobar pneumonia, lobster pneumonia, old lesions, tumor, pneumothorax, nodule, fungal pneumonia, other viral pneumonia)  (unclear if tested) |
| Jin et al.  (125) | China | Adult, Children  Median (IQR):  Case (of 43 cases): 47 (34-59)  Control: 31 (25.5-37.5)  Range: Case: 7-74 | Cases: 39 (51.3%) | Asymptomatic: 0 | real time RT-PCR | Negative molecular test |
| Kurstjens et al. (48) | Netherlands | Validation Cohort  Mean (SD):  Case: 69 (12)  control: 63 (17)  Median (IQR):  Case: 71 (61-77)  control: 67 (51-76) | Case: (63.8)  Control: (53.3) | Asymptomatic: 0 | RT-PCR | Negative RT-PCR |
| Lassaunière et al.  (126) | Denmark | NR | NR | NR | NAT | Archived anonymous serum samples obtained from healthy blood donors 18-64 years with no history of SARS-CoV-2 infection (n = 10) and no recent travel history, sera from patients with acute viral respiratory tract infections caused by other coronaviruses (n = 5) or non-coronaviruses (n = 45), and sera from patients positive for dengue virus (n = 9), cytomegalovirus (CMV; n = 2) and Epstein Barr virus (EBV; n = 10). One patient was positive for both CMV and EBV |
| Lee et al.  (127) | Taiwan | Adult cases: 14;  Median:  Symptomatic: 52  Asymptomatic: 50  Range: case: 30-88 | Case: 7 (50) | Asymptomatic/mild: 8 | rRT-PCR | Negative rRT-PCR |
| Li et al.  (128) | China | Independent Test  Mean (SD):  Covid: 52 (17)  CAP:51 (20)  Non-pneumonia: 41 (13) | 194 (55)  Case: 30 (52)  Control: 164 (84.5) | NR | RT-PCR | Randomly selected from hospital between Aug 16, 2016 and Feb 17, 2020  Community acquired pneumonia= 155 (exams: n=175)  Non-Pneumonia= 130 (exams: n= 132) |
| Li et al.  (49) | China | Adults;  Mean (Range):  Case: 57 (49-69)  Control: 51 (41-61) | 56 (53.3)  Case: 26 (50)  Control: 30 (56.6) | Asymptomatic: 0 (0%) | rRT-PCR (BioGerm, Shanghai, China) | SARS-CoV-2- negative |
| Li et al.  (129) | China | NR | NR | NR | PCR | Negative PCR |
| Lin et al.  (130) | China | Cases:  Adult: 67, Child: 12  Mean: 42.25  Range: 2-78  Control  Adult: 78, Child: 2 | 66 ((41.5)  Case: 28 (35.4)  Control: 38 (47.5) | NR | GeneoDX kit (TaqMan RT-PCR method, targeting the ORF1ab and N genes) | Healthy Cohort: 29  Tuberculosis: 51  All Negative RT-PCR |
| Liu et al.  (131) | China | Median (IQR)  confirm/control: 55 (38.3-65)  confirmed: 54 (39.0-64.0)  control: 55 (38.0-65.0) | confirm/control: 138 (58)  confirmed: 93 (60.8)  control: 45 (52.3) | Asymptomatic: 0 (0%) | rRT-PCR (Daan, Guangzhou, China) | Negative rRT-PCR |
|  |  | NR | NR |  |  | Healthy blood donor: 50  ordinary people: 70  Not all controls were tested |
| Liu et al. (132) | China | NR | NR | Asymptomatic: 0 (0%) | RT-PCR | Healthy Blood Donor  (n=100) |
| Liu et al.  (133) | China | Mean (SD)  case: 76 (15)  control: 56 (21) | 98 (54.7) | Mild/common:46 (51.1%)  Severe/critical:44 (48.9%)  Asymptomatic: 0 (0%) | RT-PCR | Negative PCR |
| Long et al.  (134) | China | Mean (SD)  case: 44.8 (18.2) | case: 20 (55.6) | Asymptomatic: 0 (0%) | rRT-PCR | NA (no control) |
| Long et al.  (135) | China | Confirmed cases  CD | CD | Asymptomatic: 0 (0%) | RT-PCR | NA (no control) |
|  |  | Suspected Cases  NR | NR | Asymptomatic: 0 (0) |  | Negative viral RNA |
|  |  | Close Contact  NR | NR | Asymptomatic: 3/16 (18.8) |  | Negative RT-PCR |
| Lou et al.  (136) | China | Median (IQR)  Total cases: 55 (45-64)  Non critical cases: 51 (38-39)  Critical cases: 65 (52-74) | Cases: 49 (61.3) | Asymptomatic: 0 (0) | qRT-PCR, commercial 1-step real-time reverse transcription polymerase chain reaction (RT-PCR) assay (Bio-Germ, Shanghai, China) | Healthy controls (n=300) |
| Lu et al.  (137) | China | NR | NR | Asymptomatic: 0 (0) | RT-PCR (Liferiver Bio-Tech) | Negative RT-PCR |
| Lu et al.  (138) | China | NR | NR | NR | SARS-CoV-2 RT-qPCR kit (Liferiver Bio, Shanghai, China) | Negative RT-qPCR |
| Ma et al.  (139) | China | Cases: Adult  Mean: cases: 47.4  Median: cases: 48  Range: cases: 21-91 | NR | Critical (5), Severe (17)  Moderate (56) Mild (9)  Asymptomatic: 0 (0) | RT-qPCR | 330 archived sera from healthy donors (samples collected before October 2019), 138 interfering sera from no-COVID-19 patients with underlying diseases, and fifteen sera from once suspected cases (negative RT-qPCR but had typical manifestation of pneumonia) |
| Meng et al.  (47) | China | Adult  Median (IQR)  Case: 48 (58-67)  control: 66 (76-84) | Case: (69.4)  Control: (68.79) | NR | RT-PCR, Viral Gene Sequencing | Negative nucleic acid detection |
| Merindol et al.  (140) | Canada | NR | NR | NR | RT-PCR (Altona RealStar® SARS-CoV-2 RT-PCR Kit) | Negative PCR |
| Miao et al.  (141) | China | Adult: 130;  Mean (SD)  Case: 45.1 (13.4)  Control: 41.8 (13.6)  Range:  case: 19-77  control: 19-81 | 77 (59.2) | Asymptomatic: 0 (0) | RT-PCR | Negative RT-PCR |
| Nörz et al.   (142) | Germany | NR | NR | NR | cobas6800-based SARS-CoV-2 UCT assay | Negative cobas6800-based SARS-CoV-2 UCT assay |
|  |  |  |  |  |  | Clinical samples positive for other respiratory samples (n=32) |
| Osterdahl et al.  (143) | United Kingdom | Adult: 21;  Median (IQR): 76 (61-81)  Range: 52-89 | 4 (30) | NR | Multiplex tandem RT-PCR | Negative PCR |
| Padoan et al.  (144) | Italy | NR | NR | NR | rRT-PCR | NA (no control) |
| Pan et al.  (145) | China | NR | NR | NR | qRT-PCR (BioGerm, Shanghai, China) | Negative rRT-PCR |
| Paradiso et al.  (146) | Italy | Median (of 191): 58.5 | (60.62) | Asymptomatic: 14/160 (8.8) | RT-PCR (Allplex2019-nCoV Assay; Seegene, Seoul, Republic of Korea) | Negative RT-PCR |
| Paradiso et al.  (147) | Italy | NR | NR | Asymptomatic: 0 (0) | RT-PCR (Allplex2019-nCoV Assay; Seegene, Seoul, Republic of Korea) | Negative RT-PCR |
| Poljak et al. (148) | Slovenia | In-house validation panel: NR | NR | NR | LightMix two-target RT-PCR (E gene and RdRp gene) | Negative RT-PCR |
|  |  | Head-to-head prospective comparison: NR |  |  |  | Negative RT-PCR |
| Qian et al.  (149) | China | Confirmed cases  Adult, Child  Mean: 53  Range: 1 month-92 years | NR | NR | RT-PCR | NA (no control) |
|  |  | Control  Adult, Child  Mean: 48  Range: 1-90 |  |  |  | Negative RT-PCR |
|  |  | Suspected Cases (of 54 cases)  Adult  Mean: 49  Range: 26-70 |  |  |  | Negative RT-PCR |
| Shen et al. (150) | China | Median (IQR)  Case: 38 (46-56)  Control: 32 (20-42.5) | 89 (59.3) | Ordinary: 76 (78.4)  Severe: 21 (21.6)  Asymptomatic: 0 (0) | real time RT-PCR | Negative RT-PCR |
| Smithgall et al.  (151) | United State | Adult: 111, Child: 2  Mean: Case: 64.9  control: 42.6  Range:  Adult: 23-101  Child: 1-5 days old | 61 (54)  Case: 53 (60.2)  Control: 8 (32.0) | NR | Roche cobas SARS-CoV-2 assay | Negative RT-PCR |
| Song et al.  (152) | China | NR | NR | NR | fluorescent RT-PCR | 86 healthy people included in testing and training set  (unclear if tested) |
| Spicuzza et al.  (153) | Italy | Mean (SD)  case: 57 (17)  suspected: 67 (15)  Control: NR | NR | Asymptomatic: 0 (0) | real time RT-PCR | Suspected/Control: Negative molecular test |
| van Kasteren et al.  (154) | Netherland | NR | NR | NR | in-house E-gene PCR | Confirmed non-coronavirus respiratory viral infections |
| Mertens et al. (155) # | Belgium and Brussels | NR | NR | Asymptomatic: 0 (0) | qRT-PCR/RT-PCR | Negative PCR |
| Visseaux et al.  (156) | France | NR | NR | NR | WHO RT-PCR | Negative RT-PCR |
| Wang et al. (157) # | China | NR | NR | NR | RT-qPCR kit (kit 1, Huirui, China) | Negative RT-qPCR |
|  |  |  |  |  | RT-qPCR | Negative RT-qPCR |
|  |  |  |  |  | multiplex PCR | Five virus-positive throat samples (influenza A virus, influenza B virus, parainfluenza, respiratory syncytial virus, and rhinovirus) collected from November 2019 to January 2020. All were previously confirmed using a China Food and Drug Administration approved kit (Health Gene Technologies, China) based on multiplex PCR and capillary electrophoresis analysis. |
| Wang et al.  (158) | China | NR | NR | NR | Nucleic acid testing | (Training and validation set)  180 cases of typical viral pneumonia diagnosed previously before the COVID-19 outbreak |
| Wang et al. (159) | China | Validation Set 1  Mean (SD): 49.15 (18.44) | 131 (58.0) | NR | RT-PCR | 124 Other pneumonia patients before Dec 2019 |
|  |  | Validation Set 2  Mean (SD): 58.44 (16.19) | 108 (67.1) |  |  | 69 Other pneumonia patients before Dec 2019 |
| Wong et al. (160) # | Hong Kong | Adult, Children;  Mean (SD): 56 (19)  Range: 16-96 | 26 (41) | Asymptomatic: 9/64 (14.1) | QuantiNova Probe RT-PCR Kit (QIAGEN, Hilden, Germany) | NA (no control) |
| Wu et al. (46) | China | External Validation  NR | NR | NR | RT-PCR | Diagnosed by at least two professional experts based on comprehensive examination results. |
| Wu et al.  (161) | China | Resuming Group  NR | NR | NR | Nucleic acid test | Negative NAT |
|  |  | Hospitalized Group  NR |  |  |  | Negative NAT |
| Xiang et al.  (162) | China | Case/Control  Median (IQR)  Of all 85 confirmed cases: 51 (32.0-65)  control: 34 (29.0-51.0) | NR | Severe (18/85 cases)  Asymptomatic: 0 (0) | RT-PCR | Negative PCR |
|  |  | Suspected  Median (IQR): 44 (35.5-60.5) | 12 (50) | Severe (2 cases)  Asymptomatic: 0 (0) |  | Negative PCR |
| Xie et al.  (163) | China | Median (IQR)  Of all 56 patients:  56.5 (49.25-64.75)  Of 34 severe patients:  60.0 (50.75-67.0) | 24 (42.9) | Asymptomatic: 0 (0) | RT-PCR | Negative nucleic acid test |
| Xie et al. (164) | China | NR | NR | mild, common, severe and fatal | rRT-PCR | NA (no control) |
| Xu et al. (165) | China | Test Set  NR | NR | NR | rRT-PCR | Healthy People & Influenza-A viral pneumonia (IAVP) (n=30 each in testing set) |
| Xu et al.  (166) | China | NR | NR | Asymptomatic: 1/6 | RT-PCR | Negative RT-PCR |
| Yan et al.  (167) | China | NR | NR | NR | rRT-PCR (BGI PathoGenesis Pharmaceutical Technology, Shenzhen, China) | Negative RT-PCR |
| Yip et al.  (168) | Hong Kong | NR | NR | NR | COVID-19-RdRp/Hel assay rRT-PCR (QuantiNova SYBR Green RT-PCR Kit (QIAGEN, Hilden, Germany)) | Negative RT-PCR |
| Yu et al.  (18) | China | Adult, Children;  Of 76 patients:  Median (IQR): 40 (32-63)  Range: 6months -92 yrs | case: 38 (50) | Mild: 59 (77.6%)  Severe: 17 (22.4%)  Asymptomatic: 2/76 (2.6) | RT-PCR kit (Shanghai BioGerm Medical Technology Co Ltd, China). | Negative RT-PCR |
| Yu et al.  (169) | China | NR | NR | NR | RT-qPCR | NA (no control) |
| Zhang et al.  (170) | China | Median  Case:46.5  Range:  Case: 19-57  negative PCR: 1-86 | Case: 4 (50)  Negative PCR: 124 (54.9) | Mild: 3  Severe: 4  Asymptomatic: 2/8 cases (25) | RT-PCR | Negative RT-PCR |
| Zhang et al. (171) | China | NR | NR | NR | RT-PCR | Negative RT-PCR |
| Zhang et al. (172) | China | NR | NR | NR | commercial COVID-19 RT-PCR kit (Jienuo Inc, Shanghai, China) | Negative RT-qPCR |
| Zhao et al. (53) | China | Median (IQR):  Case: 48 (35-61) | Case: 84 (49) | Case: critical (32), non-critical (141)  Asymptomatic: 0 (0) | rRT-PCR | Healthy individuals before the outbreak of SARS-CoV-2 |
| Zhen et al.  (173) | New York, United States | NR | NR | Asymptomatic: 0 (0) | Reference Standard not available  result obtained from at least 3 of the 4 molecular assays | Negative result from 3 molecular assays |
| Zhen et al. (174) | United State | NR | NR | Asymptomatic: 0 (0) | Hologic Panther Fusion® SARS-CoV-2 assay | Negative PCR |
| Zhong et al. (175) # | China | Adult: case: 47  Mean: Case: 48.2  Range: case: 18-82 | case: 16 (34) | Mild (22), Moderate (14), Severe (6), Critical (5) | nucleic acid test | Healthy Control (n=300) |
| Zhu et al. (176) | China | Case  Adult: 33, Child: 0  Mean: 52.5  Range: 25-76 | Case: 18 (54.5) | NR | rRT-PCR (Daangene Co. LtD. and BGI Co. LtD.) | Other pathogen patient (Negative rRT-PCR) |

*NR: Not reported. # Study is not included in analysis. *Patients characteristics mentioned may not include all recruited patients in the study*

## **Table S3b: Summary of diagnostic tests**

| **Author** | **No. of Cases/Controls**  **(Patients, P; Samples, S)** | **Novel Test** | | | | | | **Outcome** | | | | | | |
| --- | --- | --- | --- | --- | --- | --- | --- | --- | --- | --- | --- | --- | --- | --- |
|  |  | **Type of test (LFIA, CLIA etc)** | **Name of test; Manufacturer** | **Target Gene/ Protein ;**  **Antibody** | | **Sample collection; Days from onset** | | **Cut-off** | **Sensitivity (95% CI); [TP/TP+FP]** | **Specificity (95% CI); [TN/TN+FN]** | **PPV** | **NPV** | **Accuracy** | **Agreement** |
| **NAAT (Excluding Sequencing)** | | | | | | | | | | | | | | |
| Baek et al. (92) | S: 14/140 | RT-LAMP | NR | N gene | | Nasal | | Successful RT-LAMP reaction  results in a colorimetric reaction – change in the colour of phenol red pH indicator from pink to yellow | 100  (77, 100) | 99  (95, 100) | - | - | - | - |
| Broughton et al. (95) | S: 40/42 | RT-LAMP + CRISPR (output measured by fluorescence or lateral flow) | DNA Endonuclease-Targeted CRISPR Trans Reporter (DETECTR) (Output uses fluorescence) | E and N gene | | Naso/Oropharyngeal | | NR | 95  (83, 99) | 100  (92, 100) | - | - | - | - |
| Butt et al. (97) | P: 45/25 | RT-LAMP | NR | ORF1A, N gene | | Nasopharyngeal | | Positive samples turned  yellow while negative samples remained pink. | 96  (85, 99) | 100  (86, 100) | 100 | 95 | - | - |
| Chan et al. (17) | S: 77/22 | RT-PCR | COVID-19-RdRp/Hel | RdRp/Hel gene | | Respiratory (NPA/NPS/TS, Saliva, sputum) and non-respiratory (plasma, urine, faeces/rectal swabs) | | NR | 100  (95, 100) | 100  (85, 100) | - | - | - | - |
| Dong et al. (107) | P: 51/71 | RT-dPCR |  | ORF1ab, N, E gene | | Pharyngeal | | quantification of any one of the three gene targets is ≥2 copies/ reaction | 100  (93, 100) | 73  (61, 83) | - | - | 93 | - |
| Hirotsu et al. (114) | S: 10/56 | RT-PCR, double-quencher probes | YCH-N1 assay | N gene (N1) | | Nasopharyngeal | | NR | 100  (69, 100) | 98  (90, 100) | - | - | - | - |
|  | S: 17/49 |  | YCH-N2 assay | N gene (N2) | |  |  |  | 100  (93, 100) | 98  (89, 100) |  |  |  |  |
| Hou et al. (116) | S: 52/62 | RT-LAMP (CRISPR) | CRISPR n-CoV | Orf1 a/b gene | | Nasopharyngeal/ Bronchoalveolar | | Median FC (fold-change) 22.8 Range 5-66.3 | 100  (93, 100) | 100  (94, 100) | - | - | - | - |
|  |  | PCR | NR | NR | |  |  | Median Ct 35.8 Range 28.8-40.4 | 90  (71, 97) | 100  (94, 100) |  |  |  |  |
| Huang et al. (117) | S: 8/8 | RT-LAMP | NR | Orf1ab (O117 primers) | | Throat | | Positive samples turned  yellow while negative samples remained pink. | 100  (63, 100) | 100  (63, 100) | - | - | - | - |
|  |  |  |  | N gene (N15 primers) | |  |  |  | 100  (63, 100) | 100  (63, 100) |  |  |  |  |
| Jiang et al. (122) | S: 47/213 | RT-LAMP | NR | N gene | | sputum, nasopharyngeal swabs and tears | | NR | 91  (80, 98) | 100  (97, 100) | 97.7 | 98.1 | 98.08 | - |
| Lu et al. (137) | S: 28/80 | dPCR | Digital PCR (dPCR) instrument, DropX-2000 RainSure Novel Coronavirus (SARS-CoV-2) Nucleic Acid Detection Kit. | ORF1ab gene | | Pharyngeal, Stool, Serum, Sputum | | NR | 89  (72, 98) | 95  (88, 99) | - | - | 96.3 | - |
| Lu et al. (138) | S: 36/20 | RT-LAMP | NR | N gene | | Throat | | NR | 94  (81, 99) | 90  (68, 99) | - | - | - | 92.9 |
| Merindol et al. (140) | S: 65/23 | PCR | SeeGene AllplexTM 2019-nCoV RT-QPCR Assay | E/N/RdRP genes | | nasopharyngeal and oropharyngeal | | Positive: positive signal (gene E or S in Altona, genes E, N or RdRP in SeeGene) detected at any Cts. Negative: if the internal control was amplified but not the viral genes (N, E and RdRP) | 98  (92, 100) | 100  (85, 100) | - | - | - | - |
| Noerz et al. (142) | S:35/130 | Adaptation of E-gene-LDT assay for NeuModx 96 system (Fully automated system) | NR | E gene | | Oropharyngeal/Nasopharyngeal | | Median ct: 25.15 (IQR: 20.87-27.04) | 100  (90, 100) | 99  (96, 100) | - | - | - | 100 |
|  | S:0/32 |  |  |  |  |  |  |  | NA | 100  (89, 100) |  |  |  | 100 |
| Osterdahl et al. (143) | P: 10/11 | RT-LAMP | MicrosensDx RapiPrep® SARS-CoV-2 research use test. | ORF1a gene | | Pharyngeal/Deep Nasal | | NR | 80  (44,97) | 73  (39, 94) | 73  (39-94) | 80  (44-98) | - | - |
| Poljak et al. (148) | S:63/152  ( In-house validation panel) | Fully automated high throughput PCR | cobas 6800 SARS-CoV-2 (Roche Molecular Systems, Branchburg, NJ, USA) | ORF1ab & E gene | | Nasopharyngeal/Oropharyngeal | | SARS-CoV-2 positive if cobas showed positive results either  for both ORF1 (target 1) and E (target 2) genes or for the ORF1 gene only. | 95  (87, 99) | 99  (96, 100) | - | - | - | 98.1 (95.0-99.4) |
|  | S: 63/438  ( Head-to-head prospective comparison) |  |  |  |  |  |  |  | 100  (94, 100) | 100  (98, 100) | - | - | - | 99.6 (98.4-99.9) |
| van Kasteren et al. (154) | S: 13/8 | RT-PCR | Altona Diagnostics | E, S genes | | naso- and/or oropharyngeal | | Ct range: 18.2-33.85 | 77  (46, 95) | 100  (63, 100) | - | - | - | - |
|  |  |  | BGI | ORF1ab gene | |  |  | Ct range: 18.56-35 | 92  (64, 100) | 100  (63, 100) |  |  |  |  |
|  |  |  | CerTest Biotec | ORF1ab,N genes | |  |  | Ct range: 19.81-36.62 | 85  (55, 98) | 100  (63, 100) |  |  |  |  |
|  |  |  | KH Medical | S, RdRp genes | |  |  | Ct range: 21.25-40 | 92  (64, 100) | 100  (63, 100) |  |  |  |  |
|  |  |  | PrimerDesign | RdRp gene | |  |  | Ct range: 20.26-37.64 | 77  (46, 95) | 100  (63, 100) |  |  |  |  |
|  |  |  | R-BioPharm | E gene | |  |  | Ct range: 19.91-40 | 100  (75, 100) | 100  (63, 100) |  |  |  |  |
|  |  |  | Seegene | RdRp,E genes | |  |  | Ct range: 18.23-37.92 | 92  (64, 100) | 100  (63, 100) |  |  |  |  |
| Yan et al. (167) | S: 58/72 | RT-LAMP | Loopamp RNA amplification kit  (Eiken Chemical Co., Ltd., Tokyo, Japan) | orf1ab | | swabs and bronchoalveolar lavage fluid | | Colour change  from orange to green for positive  reaction. | 100  (94, 100) | 100  (95, 100) | - | - | - | - |
|  |  |  |  | S genes | |  |  |  | 100  (94, 100) | 100  (95, 100) |  |  |  |  |
| Yip et al. (168) | S: 23/36 | rRT-PCR nsp2 assay | COVID-19-nsp2 assay | nsp 2 protein (nonstructural protein) | | (26 respiratory specimens including nasopharyngeal aspirate/swab, throat swab, endotracheal aspirate, sputum and saliva  33 non-respiratory specimens including plasma, urine, rectal swab/stool | | Cp value (Crossing Point): 18.69 to 36.21 | 100  (85, 100) | 100  (90, 100) | - | - | - | - |
| Yu et al. (18) | S: 162/161 | Droplet digital PCR | COVID-19 digital PCR detection kit & TargetingOne Digital PCR System  (TargetingOne, Beijing, China) | ORF1AB gene, N gene | | Nasal swabs, throat swabs, sputum, blood, and urine | | NR | 84  (77, 89) | 98  (94, 99) | - | - | - | - |
| Yu et al. (169) | S: 248/0 | RT-LAMP | iLACO | ORF1ab gene | | NR | | By exposure to blue  light, green fluorescence for  positive reaction whereas it remained pink in negative control | 90  (85, 93) | NA | - | - | - | - |
| Zhang et al. (172) | S: 6/1 | RT-LAMP | NR | ORF1A, gene | | respiratory specimens | | Color change indicate positive sample while remain pink indicate negative sample | 100  (54, 100) | 100  (0, 100) | - | - | - | 100 |
|  |  |  |  | N gene | |  |  |  | 100  (54, 100) | 100  (0, 100) |  |  |  | - |
| Zhen et al. (173) | S: 51/53 | rRT-PCR | New York SARS-CoV-2 Real-time Reverse Transcriptase (RT)-PCR Diagnostic Panel (Modified CDC) | nucleocapsid (N) gene N1 and N2 targets | | nasopharyngeal swab | | Positive specimen when both N1 and N2 targets detected | 100  (93,100) | 98  (90, 100) | - | - | - | - |
|  | S: 51/53 | rRT-PCR | Hologic Panther Fusion® SARS-CoV-2 assay (Hologic Inc., Marlborough, MA) | ORF1ab gene | |  |  | Positive sample when either ORF1a or ORF1b detected | 100  (93,100) | 96  (87,100) |  |  |  |  |
| **NAAT (Excluding Sequencing; POC)** | | | | | | | | | | | | | | |
| Smithgall et al. (151) | S: 88/25 | RT-PCR (POC) | Cepheid Xpert Xpress SARS-CoV-2  (Cepheid, Sunnyvale, CA) | N2, E gene | | nasopharyngeal | | Ct range: 12.1-43.8  0-44.5 | 99  (94, 100) | 92  (74, 99) | - | - | - | - |
|  | S: 88/25 | RT-LAMP (POC) | Abbott ID Now SARS-CoV-2  (Abbott Chicago IL) | RdRp gene | |  |  | NR | 74  (63, 83) | 100  (86,100) |  |  |  |  |
| Visseaux et al. (156) | S: 40/29 | multiplex PCR (POC) | QIAstat-Dx Respiratory SARS-CoV-2 Panel (QIAstat-SARS) | NR | | Nasopharyngeal/lower respiratory tract (66 NPS, 1 BAL and 1 tracheal aspirate and 1 bronchial aspirate) | | NR | 100  (91, 100) | 93  (77, 99) | - | - | - | 97 |
| Zhen et al. (173) | S: 51/53 | rRT-PCR (POC) | Simplexa COVID-19 Direct  (Diasorin Molecular LLC, Cypress, CA) | S gene, ORF1ab gene | | nasopharyngeal swab | | Positive when either S or ORF1ab detected | 100  (93, 100) | 100  (93,100) | - | - | - | - |
|  | S: 51/53 | rRT-PCR (POC) | GenMark ePlex SARS-CoV-2 assay  (GenMark Diagnostics, Carlsbad, CA) | N gene | |  |  | NR | 96  (87, 100) | 100  (93, 100) |  |  |  |  |
| Zhen et al. (174) | S: 58/50 | rRT-PCR (POC) | Cepheid Xpert® Xpress SARS-CoV-2  (Cepheid) | N2, E gene | | nasopharyngeal swabs | | NR | 98  (91, 100) | 100  (93, 100) | - | - | - | - |
|  | S: 58/50 | RT-PCR (POC) | GenMark ePlex® SARS-CoV-2 Test  (GenMark) | N gene | |  |  |  | 91  (81, 97) | 100  (93, 100) |  |  |  |  |
|  | S: 57/50 | RT-LAMP (POC) | Abbott ID NOW™ COVID-19  (Abbott) | RdRp gene | |  |  |  | 88  (76, 95) | 100  (93, 100) |  |  |  |  |
| Zhu et al. (176) | S: 33/96 | mRT-LAMP-LFB (POC) | NR | ORF1ab, N gene | | oropharynx | | Color band | 100  (89, 100) | 100  (96, 100) | - | - | - | - |
| **Sequencing** | | | | | | | | | | | | | | |
| Wang et al. (157)^#^ | P: 19/11  *4 results from novel test were inconclusive | Nanopore Target Sequencing | NR | S Protein, ORF3a, M protein, ORF6, ORF7a, ORF8, N Protein,, ORF10, ORF1a | | Throat swab | | NTS>2.4: positive  NTS 1.2-2.3: inconclusive  NTS<1.2: negative | 100  (82, 100) | 43  (10, 82) | - | - | - | - |
|  | P: 9/4 |  |  |  |  |  |  |  | 100  (66, 100) | 0  (0, 60) |  |  |  |  |
|  | P: 0/5 |  |  |  |  |  |  |  | NA | 100  (48, 100) |  |  |  |  |
| **Imaging** | | | | | | | | | | | | | | |
| Ai et al. (90) | P: 108/0 | CT | NR | NA | | - | | Obvious imaging features of COVID-19 | 89  (81, 94) | NA | - | - | - | - |
| Ai et al. (91) | P: 601/413 | CT | - | NA | | - | | - | 97  (95, 98) | 25  (21, 30) | 65 (62-68) | 83 (76-89) | 68 (65-70) | - |
| Bai et al. (93)^#^ | P: 42/77 | CT (Radiologist only) | - | NA | | - | | - | 79  (64, 89) | 88  (78, 94) | - | - | 85 (77-90) | - |
| Caruso et al. (99) | P: 62/96 | CT | - | NA | | - | | Viral pneumonia | 97  (89,100) | 56  (46,66) | 59  (53-64) | 96  (87-99) | 72 (64-78) | - |
| Castiglioni et al. (101) | P: 74/36 | CXR (Radiologist 2) | - | NA | | - | | - | 64  (52,74) | 86  (71, 95) | 90(80-96) | 53 (61-79) | - | - |
| Gaeta et al. (110) | P: 72/0 | CXR Radiologist 2 | - | NA | | - | | None | 72  (60, 82) | NA | - | - | - | - |
| Gietema et al. (112) | P: 83/110  Overall | CT | Alliance Medical equipped with GE lightspeed 16 slice scanner | NA | | - | | - | 89  (80, 95) | 68  (59-77) | 67.9 (61.4-73.7) | 89.3 (81.6-94.0) | - | - |
| Guo et al. (113) | P: 2/4  (family cluster) | CT | - | NA | | - | | - | 50  (1, 99) | 50  (7, 93) | - | - | - | - |
| Imai et al. (118) | P: 112/0 | CT | - | NA | | - | | Specific features caused by COVID-19 (Based on Bai. H. X. et al. (2020)) | 69  (59, 77) | NA | - | - | - | - |
| Jiang et al. (121) | S (CT):81/283  *60 results from novel test were inconclusive | CT | - | NA | | - | | According to the Diagnosis and Treatment Program of 2019 New Coronavirus Pneumonia (trial sixth version) : bilateral multiple lobular and subsegmental areas of consolidation, bilateral ground glass opacity and subsegmental areas of consolidation | 67  (55, 77) | 68  (62, 74) | 56.8 | 92.3 | - | - |
| Long et al. (134) | P: 36/0 | CT | - | NA | | - | | lobar location and pattern of the lesion | 97  (85, 100) | NA | - | - | - | - |
| Miao et al. (141) | P: 54/76 | CT | - | NA | | - | | GGOs with bilateral pulmonary distribution | 57  (43, 71) | 80  (70, 89) | - | - | - | - |
| Wang et al. (158) | S: 165/580 | CT (Radiologist 1) | - | NA | | - | | - | 71  (63, 78) | 51  (46, 55) | 29 | 86 | 55.8 | - |
| Wong et al. (160)^#^ | P: 64/0 | X-ray (using Radiographic Assessment of Lung Edema score) | - | NA | | - | | Extent of involvement by consolidation or GGO tabulated as CXR severity score. Baseline CXR severity score >0 considered positive | 69  (56, 80) | NA | - | - | - | - |
| Xie et al. (164) | P: 167/0 | CT | - | NA | | - | | - | 96  (92, 98) | NA | - | - | - | - |
| **Imaging (AI)** | | | | | | | | | | | | | | |
| Bai et al. (93)# | NA  (Internal test set) | CT (AI only) | - | NA | | - | | - | 95  (83, 100) | 96  (88, 99) | - | - | 96 (90-98) | - |
|  | P: 42/77  (Test set) | CT (Radiologist + AI assistance) | - | NA | | - | | - | 88  (74, 95) | 91  (82, 96) |  |  | 90 (83-94) |  |
|  | P: 107/168  (External test set) | CT (AI only) | - | NA | | - | | - | 89  (81, 94) | 86  (80, 90) |  |  | 87 (82-90) |  |
|  | P: 180/215  (Equal split test set) | CT (AI only) | - | NA | | - | | - | 94  (90, 97) | 87  (82, 91) |  |  | 91 (87-93) |  |
| Castiglioni et al. (101) | P: 74/36 | CXR (AI) | - | NA | | - | | - | 80  (69, 88) | 81  (64, 92) | 89(82-94) | 66 (57-75) | - | - |
| Chen et al. (102) | P: 11/31 | CT(AI) | CTAngel |  | |  | | when three consecutive images were predicted to have lesions in the same quadrant | 100  (72, 100)  (Per person) | 94  (79, 99)  (Per person) | 84.62 | 100 | 95.24 | - |
| Chen et al. (103) # | P: 19/19 | R Model; Model using radiological features (total number of mixed GGO in peripheral area, total number of consolidation, total number of solid nodules with ground-glass opacities, interlobular septal thickening, crazy raving pattern, tree-in-bud, pleural thickening, offending vessel augmentation in lesions) | - | NA | | - | | - | 100 | 36.8 | - | - | 68.4 | - |
| Fu et al. (109) | S: 4887/CD | CT (AI) | - | NA | | - | | - | 98  (98, 99) | 98.9 | 94.5 | 99.7 | 98.8 | - |
| Jin et al. (123) | P: 751/1937  (Test Cohort) | CT (AI) | - | NA | | - | | (1) lung segmentation network, (2) slice diagnosis network, (3) COVID-infectious slice locating network, (4) visualization module for interpreting the attentional region of deep networks, and (5) image phenotype analysis  module for explaining the features of the attentional region | 87  (84, 89) | 97  (96, 97) | - | - | - | - |
|  | P: 363/907  (CC-CCII Test Cohort) |  |  |  |  |  |  |  | 93  (90, 95) | 99  (98, 100) |  |  |  |  |
| Jin et al. (124) | P: 154/128 | CT (AI) | - | NA | | - | | typical lesion characteristics of COVID-19 pneumonia, including ground-glass opacity, intralobular septal thickening, air bronchogram sign, vessel thickening, crazy-paving pattern, fibre stripes, and honeycomb lung syndrome. | 97  (93, 99) | 92  (86, 96) | - | - | - | - |
| Li et al. (128) | S: 127/307 | CT (AI) | COVID-19 detection neural network (COVNet) | NA | | Median: 7  Range: 0-20 | | - | 90  (83, 94) | 96  (93, 98) | - | - | - | - |
| Song et al. (152) | P: 27/24 | CT (AI) | DeepPneumonia | NA | | - | | - | 93  (76, 99) | 96  (79, 100) | 96 |  | 94 | - |
| Wang et al. (158) | S: 95/360  (Internal validation set) | CT (AI) | - | NA | | - | | - | 88 | 87 | 71 | 95 | 89.5 | - |
|  | S: 70/220  External validation set (based on region of interest) |  |  |  |  |  |  |  | 83 | 67 | 55 | 90 | 79.3 |  |
|  | S: 70/220  External validation set (based on patients) |  |  |  |  |  |  |  | 75 | 86 | 69 | 89 | 82.5 |  |
| Wang et al. (159) | P: 102/124  (Validation set 1) | CT (AI) | - | NA | | - | | - | 80  (71, 88) | 77  (68, 84) | - | - | 78.32 | - |
|  | P: 92/69  (Validation set 2) |  |  |  |  |  |  |  | 79  (70, 87) | 81  (70, 90) |  |  | 80.12 |  |
| Xu et al. (165) | S: 30/60  (Test Set) | CT (AI) | - | NA | | - | | Ground-glass appearance, peripheral distribution along with the pleura, and >1 independent focus of infection in a single case | 87  (69, 96) | 90  (79, 96) | 81.3 | - | - | - |
| **Serology** | | | | | | | | | | | | | | |
| Adams et al. (89) | S: 40/50 | ELISA | University of Oxford | S protein | IgG and/or IgM | Plasma  10 (4-27 (n=16))  13 (8-19 (n=6))  48 (31-62 (n=18)) | Total  4-62 | - | 85  (70, 94) | 100  (93, 100) | - | - | - | - |
|  | S: 40/50 |  |  |  | IgG |  |  | 0.4 | 85  (70, 94) | 100  (93, 100) |  |  |  |  |
|  | S: 40/0 |  |  |  | IgM |  |  | 0.07 | 70  (53, 83) | NA |  |  |  |  |
|  | S: 31/0 |  |  |  | IgG |  | >=10 | 0.4 | 100  (89, 100) | NA |  |  |  |  |
| Burbelo et al. (96) | 100/32 | LIPS | - | - | Anti-N Ab | Plasma, Serum, Blood  UCSD: 7.8 (5-14)  UW: 13.2 (4-24)  EH: 18 (2-50)  NIH: 5.5 (0-11) | Total | 125 000 LU | 68  (58, 77) | 100  (89, 100) | - | - | - | - |
|  | 35/32 |  |  |  |  |  | >14 | 125 000 LU | 100  (90, 100) | 100  (89, 100) |  |  |  |  |
|  | 100/32 |  |  | - | Anti-S Ab |  | Total | - | 60  (50, 70) | 100  (89, 100) |  |  |  |  |
|  | 35/32 |  |  |  |  |  | >14 | 45 000 LU | 91  (77, 98) | 100  (89, 100) |  |  |  |  |
| Cai et al. (98) | P: 276/167  S: 276/167 | MCLIA | - | S protein | IgG and/or IgM | Serum Range: 2-27 | Unspecified | - | 82  (76, 86) | 100  (98, 100) | - | - | - | - |
|  | P: 276/0  S: 276/0 |  |  |  | IgG |  |  | 0.7 | 71  (66, 77) | NA |  |  |  |  |
|  | P: 276/0  S: 276/0 |  |  |  | IgM |  |  | 0.7 | 57  (51, 63) | NA |  |  |  |  |
| Freeman et al. (108)# | P: 99/519 | ELISA | - | S protein | Pan IgG | Serum  >10 days | - | - | 96  (90, 99) | 99  (98, 100) | - | - | - | - |
|  |  |  |  |  | IgM |  |  |  | 76  (64, 86) | NA |  |  |  |  |
|  |  |  |  |  | IgG |  |  |  | 95  (85, 99) | NA |  |  |  |  |
| Guo et al. (113) | P: 2/4  (family cluster) | ELISA | - | N protein | IgM and/or IgG | Serum | Unspecified | 0.13 (IgM), 0.3 (IgG) | 100  (16, 100) | 25  (1, 81) | - | - | - | - |
|  |  |  |  |  | IgG and IgM |  |  |  | 0  (0, 84) | 75  (19, 99) |  |  |  |  |
|  |  |  |  |  | IgG |  |  |  | 0  (0, 84) | 75  (19, 99) |  |  |  |  |
|  |  |  |  |  | IgM |  |  |  | 100  (16, 100) | 25  (1, 81) |  |  |  |  |
|  | P: 82/58  (confirm/ probable case) | ELISA | - |  | IgM |  |  | 0.13 | 76  (65, 84) | 7  (2, 17) |  |  |  |  |
|  | S: 0/285  (negative control) |  |  |  | Ab |  |  | - | NA | 100  (99, 100) |  |  |  |  |
| Infantino et al. (119)^#^ | P: 61/64 | CLIA | SARS‐CoV‐2 antibodies IgM and IgG CLIA kits, (Shenzhen YHLO Biotech Co. Ltd, China) | N protein, S protein | IgM | Mean: 12  Range: 8-17 | Unspecified  >7 | 10 AU/mL | 73  (56, 87) | 94  (85, 98) | 81.5 (64.3-92.9) | 88.1 (78.9-94.4) | - | - |
|  |  |  |  |  | IgG |  |  | 10 AU /mL | 77  (60, 89) | 100  (94, 100) | - | 90.1 (81.8-95.6) |  |  |
| Jia et al. (120) | P: 24/33 | Fluorescence immunochromatographic assay | COVID IgM/IgG antibodies kit  (Beijing Diagreat Biotechnologies Co., Ltd) | - | IgG and/ or IgM | Range 1-34 | Unspecified | - | 88  (68, 97) | 27  (13, 46) | - | - | - | - |
|  |  |  |  |  | IgM |  |  | 0.88 Flu | 79  (58, 93) | 39  (23, 58) |  |  |  |  |
|  |  |  |  |  | IgG |  |  | 1.02 Flu | 67  (45, 84) | 55  (36, 72) |  |  |  |  |
| Jin et al. (125) | P: 27/33 | CLIA | SARS-CoV-2 IgM and IgG chemiluminescence immunoassay (CLIA) kits (Shenzhen YHLO Biotech Co. Ltd , China) | N protein, S protein | IgG | Serum  Case: 16 (IQR: 9-20)  control: 3 (IQR:2-8) | Unspecified | 10 AU/ml | 89  (71, 98) | 91  (76, 98) | 88.9 | 90.9 | - | - |
|  |  |  |  |  | IgM |  |  | - | 48  (29, 68) | 100  (89, 100) | 100 | 70.2 |  |  |
| Lassaunière et al. (126) | P: 30/82 | ELISA | Wantai SARS-CoV2 Ab ELISA  (Beijing Wantai Biological Pharmacy Enterprise) | Spike protein | Total Ab | Serum | Total (≥7) | adding the calculated negative control value to 0.160 | 93  (78, 99) | 100  (96, 100) | 100 | 98 | - | - |
|  | P: 30/82 |  | Euroimmun IgA* (Euroimmun Medizinische Labordiagnostika, Lübeck, Germany) | Spike protein (S1) | Ig A |  | Total (≥7) | ratio >=1.1 | 93  (78, 99) | 93  (85, 97) | 82 | 97 | - | - |
|  | P: 30/82 |  | Euroimmun IgG* (Euroimmun Medizinische Labordiagnostika, Lübeck, Germany) | Spike protein (S1) | IgG |  | Total (≥7) | ratio >=1.1 | 67  (47, 83) | 96  (90, 99) | 87 | 89 | - | - |
| Lin et al. (130) | P: 65/64 | ELISA | Darui Biotech | Nucleocapsid Antigen | IgM | Blood | Unspecified | - | 46  (34, 59) | 78  (66, 87) | - | - | - | - |
|  |  |  |  |  | IgG |  |  |  | 23  (14, 35) | 100  (94, 100) |  |  |  |  |
|  | P: 79/80 | chemiluminescence immunoassay | - | Nucleocapsid Antigen | IgG and IgM | Blood | Total | - | 58  (47, 69) | 99  (93, 100) | - | - | - | - |
|  | P: 12/0 |  |  |  |  |  | ≤7 |  | 50  (21, 79) | NA |  |  |  |  |
|  | P: 67/0 |  |  |  |  |  | >7 |  | 60  (47, 72) | NA |  |  |  |  |
|  | P: 79/80 |  |  |  | IgG and/or IgM |  | Total |  | 85  (75, 92) | 91  (83, 96) |  |  |  |  |
|  | P: 12/0 |  |  |  |  |  | ≤7 |  | 67  (35, 90) | NA |  |  |  |  |
|  | P: 67/0 |  |  |  |  |  | >7 |  | 88  (78, 95) | NA |  |  |  |  |
|  | P: 79/80 |  |  |  | IgM |  | Total |  | 61  (49, 72) | 92  (84, 97) |  |  |  |  |
|  | P: 12/0 |  |  |  |  |  | ≤7 |  | 50  (21, 79) | NA |  |  |  |  |
|  | P: 67/0 |  |  |  |  |  | >7 |  | 63  (50, 74) | NA |  |  |  |  |
|  | P: 79/80 |  |  |  | IgG |  | Total |  | 82  (72, 90) | 98  (91, 100) |  |  |  |  |
|  | P: 12/0 |  |  |  |  |  | ≤7 |  | 67  (35, 90) | NA |  |  |  |  |
|  | P: 67/0 |  |  |  |  |  | >7 |  | 85  (74, 93) | NA |  |  |  |  |
| Liu et al. (131) | P: 153/85 | ELISA | Lizhu, Zhuhai, China | N protein | IgG and/or IgM | Serum | Total | - | 83  (76, 89) | 21  (13, 31) | - | - | - | - |
|  | P: 0/120 |  |  |  |  |  | Unspecified |  | NA | 97  (92, 99) |  |  |  |  |
| Liu et al. (132) | P: 214/100 | rN-based ELISA | Lizhu, Zhuhai, China | N Protein | IgG and/or IgM | Serum  Median: 15  Range: 0-55 | Total | - | 80  (74, 85) | 100  (96, 100) | - | - | - | - |
|  | P: 154/0 |  |  |  |  |  | >10 |  | 93  (88, 96) | NA |  |  |  |  |
|  | P: 214/100 |  |  |  | IgG |  | Total |  | 70  (63, 76) | 100  (96, 100) |  |  |  |  |
|  | P: 154/0 |  |  |  |  |  | >10 |  | 83  (76, 89) | NA |  |  |  |  |
|  | P: 214/100 |  |  |  | IgM |  | Total |  | 68  (62, 74) | 100  (96, 100) |  |  |  |  |
|  | P: 154/0 |  |  |  |  |  | >10 |  | 77  (70, 84) | NA |  |  |  |  |
|  | P: 214/100 | rS-based ELISA | Hotgen, Beijing, China | S Protein | IgG and/or IgM |  | Total |  | 82  (76, 87) | 100  (96, 100) |  |  |  |  |
|  | P: 154/0 |  |  |  |  |  | >10 |  | 93  (88, 96) | NA |  |  |  |  |
|  | P: 214/100 |  |  |  | IgG |  | Total |  | 74  (68, 80) | 100  (96, 100) |  |  |  |  |
|  | P: 154/0 |  |  |  |  |  | >10 |  | 85  (78, 90) | NA |  |  |  |  |
|  | P: 214/100 |  |  |  | IgM |  | Total |  | 77  (71, 83) | 100  (96, 100) |  |  |  |  |
|  | P: 154/0 |  |  |  |  |  | >10 |  | 90  (84, 94) | NA |  |  |  |  |
| Long et al. (135) | S:363/0 | MCLIA | Bioscience Co. | Nucleoprotein, S protein | IgG and/ or IgM | Serum | Total | Antibody levels were presented as the measured chemiluminescence values divided by the cutoff (absorbance/cutoff, S/CO): S/CO > 1 was defined as positive and S/CO ≤ 1 as negative. | 83  (79, 87) | NA | - | - | - | - |
|  | S: 67/0 |  |  |  |  |  | ≤7 |  | 51  (38, 63) | NA |  |  |  |  |
|  | S: 296/0 |  |  |  |  |  | >7 |  | 91  (87, 94) | NA |  |  |  |  |
|  | S:363/0 |  |  |  | IgG |  | Total |  | 79  (75, 83) | NA |  |  |  |  |
|  | S: 67/0 |  |  |  |  |  | ≤7 |  | 48  (35, 60) | NA |  |  |  |  |
|  | S: 296/0 |  |  |  |  |  | >7 |  | 86  (82, 90) | NA |  |  |  |  |
|  | S:363/0 |  |  |  | IgM |  | Total |  | 67  (62, 72) | NA |  |  |  |  |
|  | S: 67/0 |  |  |  |  |  | ≤7 |  | 31  (21, 44) | NA |  |  |  |  |
|  | S: 296/0 |  |  |  |  |  | >7 |  | 75  (70, 80) | NA |  |  |  |  |
|  | P: 0/52 |  |  |  | IgG and/ or IgM |  | Unspecified |  | NA | 92  (81, 98) |  |  |  |  |
|  | P: 16/148 |  |  |  | IgG and/ or IgM |  | Unspecified |  | 100  (79, 100) | 95  (90, 98) |  |  |  |  |
| Lou et al. (136) | P: 80/300 | ELISA | - | S protein | Ab | Blood  Median: 8 (IQR: 6-10) | Total | - | 98  (91, 100) | 100  (99, 100) | - | - | - | - |
|  | P: 39/0 |  |  |  |  |  | ≤7 |  | 64  (47, 79) | NA |  |  |  |  |
|  | P: 135/0 |  |  |  |  |  | >7 |  | 99  (96, 100) | NA |  |  |  |  |
|  | P: 80/300 |  |  |  | IgM |  | Total |  | 92  (84, 97) | 100  (99, 100) |  |  |  |  |
|  | P: 39/0 |  |  |  |  |  | ≤7 |  | 33  (19, 50) | NA |  |  |  |  |
|  | P: 135/0 |  |  |  |  |  | >7 |  | 91  (85, 95) | NA |  |  |  |  |
|  | P: 80/100 |  |  | N protein | IgG |  | Total |  | 89  (80, 95) | 100  (96, 100) |  |  |  |  |
|  | P: 39/0 |  |  |  |  |  | ≤7 |  | 33  (19, 50) | NA |  |  |  |  |
|  | P: 135/0 |  |  |  |  |  | >7 |  | 84  (76, 89) | NA |  |  |  |  |
|  | P: 80/300 | CMIA |  | S protein | Ab | Blood  Median: 8 (IQR: 6-10) | Total | - | 96  (89, 99) | 99  (98, 100) | - | - | - | - |
|  |  |  |  |  | IgM |  |  |  | 86  (77, 93) | 99  (98, 100) |  |  |  |  |
| Ma et al. (139) | S:216/483 | CLIA | - | Receptor-binding domain (RBD) | IgA and IgM | Serum | Unspecified | - | 96  (92, 98) | 91  (88, 93) | - | - | - | 92.3 |
|  | S:216/483 |  |  |  | IgA and IgG |  |  |  | 96  (93, 98) | 98  (96, 99) |  |  |  | 97.4 |
|  | S:216/483 |  |  |  | IgM and IgG |  |  |  | 95  (91, 97) | 92  (89, 94) |  |  |  | 93 |
|  | S:216/483 |  |  |  | IgA and IgM and IgG |  |  |  | 94  (90, 97) | 90  (88, 93) |  |  |  | 91.7 |
|  | S:216/483 |  |  |  | IgA or IgM |  |  |  | 100  (97, 100) | 100  (99, 100) |  |  |  | 99.7 |
|  | S:216/483 |  |  |  | IgA or IgG |  |  |  | 99  (97, 100) | 100  (99, 100) |  |  |  | 99.7 |
|  | S:216/483 |  |  |  | IgM and/ or IgG |  |  |  | 99  (96, 100) | 100  (99, 100) |  |  |  | 99.6 |
|  | S:216/483 |  |  |  | IgA or IgM or IgG |  |  |  | 100  (97, 100) | 100  (99, 100) |  |  |  | 99.9 |
|  | S:216/483 |  |  |  | IgA |  | Unspecified |  | 99  (96, 100) | 98  (96, 99) |  |  |  | 98.3 |
|  | S: 199/0 |  |  |  |  |  | >10 |  | 99  (97, 100) | NA |  |  |  | - |
|  | S:216/483 |  |  |  | IgM |  | Unspecified |  | 97  (93, 99) | 92  (90, 95) |  |  |  | 93.7 |
|  | S: 199/0 |  |  |  |  |  | >10 |  | 98  (96, 100) | NA |  |  |  | - |
|  | S:216/483 |  |  |  | IgG |  | Unspecified |  | 97  (93, 99) | 100  (99, 100) |  |  |  | 98.9 |
|  | S: 199/0 |  |  |  |  |  | >10 |  | 99  (97, 100) | NA |  |  |  | - |
| Padoan et al. (144) | S: 70/0 | CLIA | - | - | IgM | serum | Total | 1.0 AU/mL | 63  (50, 74) | NA | - | - | - | - |
|  | S: 10/0 |  |  |  |  |  | ≤7 |  | 30  (7, 65) | NA |  |  |  |  |
|  | S: 60/0 |  |  |  |  |  | >7 |  | 68  (55, 80) | NA |  |  |  |  |
|  | S: 70/0 |  |  |  | IgG |  | Total | 1.1 AU/mL | 81  (70, 90) | NA |  |  |  |  |
|  | S: 10/0 |  |  |  |  |  | ≤7 |  | 40  (12, 74) | NA |  |  |  |  |
|  | S: 60/0 |  |  |  |  |  | >7 |  | 88  (77, 95) | NA |  |  |  |  |
| Paradiso et al. (147) | P: 0/6 | CLIA | MAGLUMI SARS-CoV-2 IgM/IgG Kit  (Shenzhen New Industries Biomedical Engineering Co) | - | IgG and/or IgM | Blood/ Serum  Range: 0-34 | Unspecified | CLIA >1 AU/mL | NA | 50  (12, 88) | - | - | - | - |
|  |  |  |  |  | IgG and IgM |  |  |  | NA | 83  (36, 100) |  |  |  |  |
|  |  |  |  |  | IgM |  |  |  | NA | 50  (12, 88) |  |  |  |  |
|  |  |  |  |  | IgG |  |  | CLIA >1 AU/mL | NA | 83  (36, 100) |  |  |  |  |
| Qian et al. (149) | P: 513/0 | CLIA | - | - | IgM | - | Total | 10 kAU/L | 86  (83, 89) | NA | - | - | - | - |
|  | P: 63/0 |  |  |  |  |  | <7 |  | 83  (71, 91) | NA |  |  |  |  |
|  | P: 450/0 |  |  |  |  |  | ≥7 |  | 86  (83, 89) | NA |  |  |  |  |
|  | P: 513/0 |  |  |  | IgG |  | Total |  | 97  (95, 98) | NA |  |  |  |  |
|  | P: 63/0 |  |  |  |  |  | <7 |  | 81  (69, 90) | NA |  |  |  |  |
|  | P: 450/0 |  |  |  |  |  | ≥7 |  | 99  (97, 100) | NA |  |  |  |  |
|  | P: 0/972  (Control) |  |  |  | IgM |  | Unspecified |  | NA | 97  (96, 98) |  |  |  |  |
|  |  |  |  |  | IgG |  | Unspecified |  | NA | 97  (96, 98) |  |  |  |  |
|  | P: 0/52  (suspected) |  |  |  | IgM |  | Unspecified |  | NA | 27  (16, 41) |  |  |  |  |
|  |  |  |  |  | IgG |  | Unspecified |  | NA | 13  (6, 26) |  |  |  |  |
| Xiang et al. (162) | P: 66/60  (case/control) | ELISA | Livzon Inc, Zhuhai, China, lot numbers 20200308 [IgM] and 20200308 [IgG] | N protein | IgM | Serum | Unspecified | - | 77  (65, 87) | 100  (94, 100) | 100 | 80 |  | 88.1 |
|  |  |  |  |  | IgG |  |  |  | 83  (72, 91) | 95  (86, 99) | 94.8 | 83.8 |  | 88.9 |
|  | P: 0/24  (suspected) |  |  |  | IgM |  |  |  | NA | 12  (3, 32) |  |  |  |  |
|  |  |  |  |  | IgG |  |  |  | NA | 29  (13, 51) |  |  |  |  |
| Xie et al. (163) | P: 16/40 | Chemiluminescence immunoassay | YHLO Biological Technology Co, Ltd, Shenzhen, China | E and N protein | IgM | Serum | Unspecified | 10 AU/mL | 94  (70, 100) | 15  (6, 30) | - | - | - | - |
|  |  |  |  |  | IgG |  |  |  | 100  (79, 100) | 0  (0, 9) |  |  |  |  |
| Xu et al. (166) | P: 4/2 | ELISA | Institute of Pathogen Biology, Chinese Academy of Medical Sciences & Peking Union Medical College | - | IgG and/or IgM | - | Unspecified | - | 100  (40, 100) | 50  (1, 99) | - | - | - | - |
|  |  |  |  |  | IgG and IgM |  |  |  | 25  (1, 81) | 100  (16, 100) |  |  |  |  |
|  |  |  |  |  | IgM |  |  |  | 100  (40, 100) | 50  (1, 99) |  |  |  |  |
|  |  |  |  |  | IgG |  |  |  | 25 (1, 81) | 100 (16, 100) |  |  |  |  |
| Zhang et al. (170) | P: 8/226 | automated chemiluminescent immunoassay | Shenzhen Yahuilong Biotechnology Co. Ltd., Shenzhen, China | S protein (S1), Nucleocapsid protein (N) | IgM | Venous blood | Unspecified | 10 AU/mL | 88  (47, 100) | 97  (94, 99) | - | - | - | - |
|  |  |  |  |  | IgG |  |  |  | 100  (63, 100) | 99  (97, 100) |  |  |  |  |
| Zhao et al. (53) | P: 173/213 | ELISA | Beijing Wantai Biological Pharmacy Enterprise Co.,Ltd | Spike Protein | Ab | Plasma  Median: 7 (IQR: 5-10) | Total | - | 93  (88, 96) | 99  (97, 100) | - | - | - | - |
|  | P: 94/0 |  |  |  |  |  | ≤7 |  | 38 (28, 49) | NA |  |  |  |  |
|  | P: 225/0 |  |  |  |  |  | >7 |  | 94  (90, 97) | NA |  |  |  |  |
|  | P: 173/213 |  |  |  | IgM |  | Total |  | 83  (76, 88) | 99  (96, 100) |  |  |  |  |
|  | P: 94/0 |  |  |  |  |  | ≤7 |  | 29  (20, 39) | NA |  |  |  |  |
|  | P: 223/0 |  |  |  |  |  | >7 |  | 82  (76, 86) | NA |  |  |  |  |
|  | P: 173/197 |  |  | Nucleoprotein | IgG |  | Total |  | 65  (57, 72) | 99  (96, 100) |  |  |  |  |
|  | P: 94/0 |  |  |  |  |  | ≤7 |  | 19  (12, 29) | NA |  |  |  |  |
|  | P: 224/0 |  |  |  |  |  | >7 |  | 64  (58, 71) | NA |  |  |  |  |
| Zhong et al. (175)# | P: 47/300 | ELISA | - | N | IgG | Serum  Mean: 15.5  Range: 1-29 | Unspecified | 0.443 | 98 | 100 | - | - | - | - |
|  |  |  |  |  | IgM |  |  | - | 98 | 100 |  |  |  |  |
|  |  |  |  | S | IgG |  |  | 0.176 | 96 | 86 |  |  |  |  |
|  |  |  |  |  | IgM |  |  | 0.167 | 89 | 97 |  |  |  |  |
|  |  | Chemiluminescence |  | S and N protein | IgG |  |  | 0.199 | 96 | 97 |  |  |  |  |
|  |  |  |  |  | IgM |  |  | 0.23 | 98 | 95 |  |  |  |  |
| **Serology (POC)** | | | | | | | | | | | | | | |
| Adams et al. (89) | S: 33/60 | LFIA | (commercial) | - | IgG and/or IgM | Plasma  10 (4-27 (n=16)) 13 (8-19 (n=6))  48 (31-62 (n=18)) | Unspecified | - | 55  (36, 72) | 100  (94, 100) | - | - | - | - |
|  | S: 24/0 |  |  |  |  |  | ≥ 10 |  | 67  (45, 84) | NA |  |  |  |  |
|  | S: 38/91 | LFIA | (commercial) |  |  |  | Unspecified | - | 61  (43, 76) | 99  (94, 100) |  |  |  |  |
|  | S: 31/0 |  |  |  |  |  | ≥ 10 |  | 68  (49, 83) | NA |  |  |  |  |
|  | S: 33/60 | LFIA | (commercial) |  |  |  | Unspecified | - | 64  (45, 80) | 97  (88-100) |  |  |  |  |
|  | S: 24/0 |  |  |  |  |  | ≥ 10 |  | 75  (53, 90) | NA |  |  |  |  |
|  | S: 38/60 | LFIA | (commercial) |  |  |  | Unspecified | - | 66  (49, 80) | 98  (91, 100) |  |  |  |  |
|  | S: 29/0 |  |  |  |  |  | ≥ 10 |  | 76  (56, 90) | NA |  |  |  |  |
|  | S: 31/60 | LFIA | (commercial) |  |  |  | Unspecified | - | 61  (42, 78) | 97  (88, 100) |  |  |  |  |
|  | S: 22/0 |  |  |  |  |  | ≥ 10 days |  | 77  (55, 92) | NA |  |  |  |  |
|  | S: 31/60 | LFIA | (commercial) |  |  |  | Unspecified | - | 65  (45, 81) | 98  (91, 100) |  |  |  |  |
|  | S: 22/0 |  |  |  |  |  | ≥ 10 |  | 77  (55, 92) | NA |  |  |  |  |
|  | S: 33/60 | LFIA | (commercial) |  |  |  | Unspecified | - | 70  (51, 84) | 95  (86, 99) |  |  |  |  |
|  | S: 24/0 |  |  |  |  |  | ≥ 10 |  | 88  (68, 97) | NA |  |  |  |  |
|  | S: 32/60 | LFIA | (commercial) |  |  |  | Unspecified | - | 56  (38, 74) | 100  (94, 100) |  |  |  |  |
|  | S: 23/0 |  |  |  |  |  | ≥ 10 |  | 61  (39, 80) | NA |  |  |  |  |
|  | S: 40/142 | LFIA | (commercial) |  |  |  | Unspecified | - | 55  (38, 71) | 97  (93, 99) |  |  |  |  |
|  | S: 31/0 |  |  |  |  |  | ≥ 10 |  | 65  (45, 81) | NA |  |  |  |  |
| Cassaniti et al. (100) | P: 30/30  (Intensive Care Unit) | LFIA | VivaDiag COVID‐19 IgM/IgG Rapid Test (VivaChek) | - | IgG and/or IgM | Serum, Blood | Unspecified | - | 83  (65, 94) | 100  (88, 100) | - | - | - | - |
|  |  |  |  |  | IgG and IgM |  |  |  | 80  (61, 92) | 100  (88, 100) |  |  |  |  |
|  | P: 38/12  (Emergency Room) | LFIA |  |  | IgG and/ or IgM |  |  | - | 18  (8, 34) | 92  (62, 100) | 87.5 | 26.2 |  |  |
|  |  |  |  |  | IgM |  |  |  | 16  (6, 31) | 92  (62, 100) | - | - |  |  |
|  |  |  |  |  | IgG |  |  |  | 13  (4, 28) | 100  (74, 100) |  |  |  |  |
| Chen et al. (104) | S: 7/12 | LFIA | - | Nucleocapsid phosphoprotein | IgG | Serum | Unspecified | 0.66 | 100  (59, 100) | 92  (62, 100) | - | - | - | - |
| Döhla et al. (106) | P: 20/27 | SARS-CoV-2 rapid test, Qualitative IgG/IgM | - | - | IgG and/or IgM | Blood (Finger prick) | Unspecified | - | 36  (17, 59) | 89  (71,98) | 72.7 (39.0-94.0) | 63.2 (46.0-78.2) | - | - |
| Garcia et al. (111) | P: 0/100  (Group 1) | LFIA | AllTest COVID-19 IgG/IgM  (Hangzhou ALLTEST Biotech Co., Ltd., China) | - | IgG and/or IgM | Unspecified | NA | - | NA | 100  (96, 100) | - | - | - | - |
|  |  |  |  |  | IgG |  |  |  | NA | 100  (96, 100) |  |  |  |  |
|  |  |  |  |  | IgM |  |  |  | NA | 100  (96, 100) |  |  |  |  |
|  | P:90/61  (Group 2 and 3) |  |  |  | IgG and/or IgM | Group 2 (PCR positive)  Overall: 17 (IQR: 9-25)  Group 3 (patients with pneumonia of unknown etiology and negative PCR)  Overall: 17 (IQR: 15-20) | Overall |  | 64  (54, 74) | 11  (5, 22) |  |  |  |  |
|  | P: 19/0  (Group 2) |  |  |  |  |  | ≤7 |  | 26  (9, 51) | NA |  |  |  |  |
|  | P: 71/61  (Group 2 and 3) |  |  |  |  |  | >7 |  | 75  (63, 84) | 11  (5, 22) |  |  |  |  |
|  | P:90/61  (Group 2 and 3) |  |  |  | IgG |  | Overall |  | 60  (49, 70) | 11  (5, 22) |  |  |  |  |
|  | P: 19/0  (Group 2) |  |  |  |  |  | ≤7 |  | 21  (6, 46) | NA |  |  |  |  |
|  | P: 71/61  (Group 2 and 3) |  |  |  |  |  | >7 |  | 70  (58, 81) | 11  (5, 22) |  |  |  |  |
|  | P:90/61  (Group 2 and 3) |  |  |  | IgM |  | Overall |  | 29  (20, 39) | 62  (49, 74) |  |  |  |  |
|  | P: 19/0  (Group 2) |  |  |  |  |  | ≤7 |  | 21  (6, 46) | NA |  |  |  |  |
|  | P: 71/61  (Group 2 and 3) |  |  |  |  |  | >7 |  | 31  (21, 43) | 62  (49, 74) |  |  |  |  |
| Hoffman et al. (115) | P: 29/124  S: 29/124 | COVID-19 IgM and IgG rapid test | COVID-19 IgG/IgM Rapid Test Cassette  (Zhejiang Orient Gene Biotech Co Ltd) | - | IgM | blood samples, serum | 9-29 (Total/ >7) | - | 69  (49, 85) | 100  (97, 100) | 100 | 93.2 | - | 94.1 |
|  |  |  |  |  | IgG |  |  |  | 93  (77, 99) | 99  (96, 100) | 96.4 | 98.4 |  | 98 |
| Imai et al. (118) | S: 139/48 | IC | One Step Novel Coronavirus (COVID-19) IgM/IgG Antibody Test | - | IgG and/ or IgM | Serum  6 (IQR: 3-13) | Overall | - | 43  (35, 52) | 98  (89, 100) | - | - | - | - |
|  |  |  |  |  | IgM |  |  |  | 43  (35, 52) | 98  (89, 100) |  |  |  |  |
|  |  |  |  |  | IgG |  |  |  | 14  (9, 21) | 100  (93, 100) |  |  |  |  |
|  | S: 90/0 |  |  |  | IgG and/ or IgM |  | <7 |  | 28  (19, 38) | NA |  |  |  |  |
|  |  |  |  |  | IgM |  |  |  | 28  (19, 38) | NA |  |  |  |  |
|  |  |  |  |  | IgG |  |  |  | 3  (1, 9) | NA |  |  |  |  |
|  | S: 49/0 |  |  |  | IgG and/ or IgM |  | ≥7 |  | 71  (57, 83) | NA |  |  |  |  |
|  |  |  |  |  | IgM |  |  |  | 71  (57, 83) | NA |  |  |  |  |
|  |  |  |  |  | IgG |  |  |  | 35  (22, 50) | NA |  |  |  |  |
| Lassaunière et al. (126) | P: 30/32 | LFIA | 2019-nCOV IgG/IgM Rapid Test  (Dynamiker Biotechnology) | - | IgG and/or IgM | Serum | Total (≥7) | - | 90  (73, 98) | 100  (89, 100) | 100 | 89 | - | - |
|  | P: 30/32 | LFIA | OnSiteTM COVID-19 IgG/IgM Rapid Test  (CTK biotech) |  | IgG and/or IgM |  | Total (≥7) | - | 90  (73, 98) | 100  (89, 100) | 100 | 89 |  |  |
|  | P: 30/32 | LFIA | Anti-SARS-CoV-2 Rapid Test  (Autobio Diagnostics) |  | IgG and/or IgM |  | Total (≥7) | - | 93  (78, 99) | 100  (89, 100) | 100 | 91 |  |  |
|  | P: 30/17 | LFIA | Coronavirus Diseases 2019 (COVID-19) IgM/IgG Antibody Test  (Artron Laboratories) |  | IgG and/or IgM |  | Total (≥7) | - | 83  (65, 94) | 100  (80, 100) | 100 | 74 |  |  |
|  | P: 5/15 | LFIA | 2019-nCoV IgG/IgM Rapid Test Cassette  (Acro Biotech) |  | IgG and/or IgM |  | Total | - | 80  (28, 99) | 80  (52, 96) | 57 | 92 |  |  |
|  | P: 1/15 | LFIA | 2019-nCoV IgG/IgM Rapid Test Cassette (Alltest Biotech) |  | IgG and/or IgM |  | Total | - | 100  (3, 100) | 87  (60, 98) | - | - |  |  |
| Lee et al. (127) | P: 14/28 | LFIA | ALLTEST 2019-nCoV IgG/IgM Rapid Test Cassette  (Hangzhou ALLTEST Biotech Co., Ltd. Hangzhou, China) | N Protein | IgG and/or IgM | Serum | Unspecified | - | 79  (49, 95) | 100  (88, 100) | - | - | - | - |
|  |  |  |  |  | IgG and IgM |  |  |  | 29  (8, 58) | 100  (88, 100) |  |  |  |  |
|  |  |  |  |  | IgM |  |  |  | 29  (8, 58) | 100  (88, 100) |  |  |  |  |
|  |  |  |  |  | IgG |  |  |  | 79  (49, 95) | 100  (88, 100) |  |  |  |  |
| Li et al. (129) | P: 397/128  S: 397/128 | LFIA | Jiangsu Medomics Medical Technologies | S protein | IgG and/or IgM | Blood (Serum and Plasma) | Unspecified | - | 89  (85, 92) | 91  (84, 95) | - | - | - | - |
|  |  |  |  |  | IgG and IgM |  |  |  | 60  (60, 69) | 99  (96, 100) |  |  |  |  |
|  |  |  |  |  | IgM |  |  |  | 18  (14, 22) | 92  (86, 96) |  |  |  |  |
|  |  |  |  |  | IgG |  |  |  | 6  (4, 9) | 99  (96, 100) |  |  |  |  |
| Liu et al. (133) | P: 90/89 | SARS-CoV-2 IgG/IgM test kit (POC) | SARS-CoV-2 IgG/IgM antibody test kit  (a Chinese biotechnology company) | - | IgG and/or IgM | Serum;  Mean:  case: 30 (17)  control: 18 (14) | Total | - | 86  (77, 92) | 91  (83, 96) | 95.1 | 82.7 | 88.3 | - |
|  | P: 16/9 |  |  |  |  |  | ≤7 |  | 19  (4, 46) | 78  (40, 97) | - | - | 40 |  |
|  | P: 74/ 16 |  |  |  |  |  | >7 |  | 100  (95, 100) | 62  (32, 85) |  |  | - |  |
|  | P: 90/25 |  |  |  | IgG and IgM |  | Overall |  | 36  (26, 46) | 80  (59, 93) | - | - | - |  |
|  | P: 16/9 |  |  |  |  |  | ≤7 |  | 13  (2, 38) | 89  (52, 100) |  |  |  |  |
|  | P: 74/ 16 |  |  |  |  |  | >7 |  | 41  (29, 53) | 75  (48, 93) |  |  |  |  |
|  | P: 90/25 |  |  |  | IgG |  | Overall |  | 48  (37, 59) | 92  (74, 99) |  |  |  |  |
|  | P: 16/9 |  |  |  |  |  | ≤7 |  | 0  (0, 21) | 89  (52, 100) |  |  |  |  |
|  | P: 74/ 16 |  |  |  |  |  | >7 |  | 58  (46, 69) | 94  (70, 100) |  |  |  |  |
|  | P: 90/25 |  |  |  | IgM |  | Overall |  | 2  (0, 8) | 96  (80, 100) |  |  |  |  |
|  | P: 16/9 |  |  |  |  |  | ≤7 |  | 6  (0, 30) | 100  (66, 100) |  |  |  |  |
|  | P: 74/ 16 |  |  |  |  |  | >7 |  | 1  (0, 7) | 94  (70, 100) |  |  |  |  |
| Lou et al. (136) | P: 80/209 | LFIA (POC) | - | S Protein | Ab | Blood  Median: 8 (IQR: 6-10) | Total | - | 97  (91, 100) | 95  (91, 98) | - | - | - | - |
|  |  |  |  |  | IgM |  |  |  | 89  (80, 95) | 98  (95, 99) |  |  |  |  |
|  |  |  |  | N protein | IgG |  |  |  | 86  (77, 93) | 100  (97, 100) |  |  |  |  |
| Pan et al. (145) | S:86/22 | Colloidal gold-based immunochromatographic (ICG) strip assay | Zhuhai Livzon Diagnositic Inc. | - | IgM and/or IgG | Whole Blood/Serum/Plasma  Range: 0-34 | Total | - | 69  (58, 78) | 36  (17, 59) | - | - | - | - |
|  | S: 27/9 |  |  |  |  |  | ≤7 |  | 11  (2, 29) | 56  (21, 86) |  |  |  |  |
|  | S: 59/13 |  |  |  |  |  | >7 |  | 95  (86, 99) | 23  (5, 54) |  |  |  |  |
|  | S:86/22 |  |  |  | IgM |  | Total |  | 56  (45, 67) | 64  (41, 83) |  |  |  |  |
|  | S: 27/9 |  |  |  |  |  | ≤7 |  | 11  (2, 29) | 78  (40, 97) |  |  |  |  |
|  | S: 59/13 |  |  |  |  |  | >7 |  | 76  (63, 86) | 54  (25, 81) |  |  |  |  |
|  | S:86/22 |  |  |  | IgG |  | Total |  | 55  (44, 65) | 41  (21, 64) |  |  |  |  |
|  | S: 27/9 |  |  |  |  |  | ≤7 |  | 4  (0, 19) | 56  (21, 86) |  |  |  |  |
|  | S: 59/13 |  |  |  |  |  | >7 |  | 78  (65, 88) | 31  (9, 61) |  |  |  |  |
| Paradiso et al. (146) | P: 70/120 | LFIA | Viva-DiagTM kit  (Jiangsu Medomics Medical Technologies) | - | IgM and/or IgG | Serum | Unspecified | - | 30  (20, 42) | 89  (82, 94) | 67 (60.4-74.1) | - | - | - |
| Paradiso et al. (147) | P: 0/6 | LFIA | Viva-DiagTM kit  (Jiangsu Medomics Medical Technologies) | - | IgM and/or IgG | Serum | Unspecified | signal/cutoff (S/C) ratio ≥1 | NA | 0  (0, 46) | - | -- | - | - |
|  |  |  |  |  | IgG and IgM |  |  |  |  | 83  (36, 100) |  |  |  |  |
|  |  |  |  |  | IgG |  |  |  |  | 83  (36, 100) |  |  |  |  |
|  |  |  |  |  | IgM |  |  |  |  | 0  (0, 46) |  |  |  |  |
| Shen et al. (150) | P: 97/53 | colloidal gold immunochromatography assay (POC) | colloidal gold immunochromatography antibody detection kit (Shanghai Outdo Biotech Co. Ltd, China) | - | IgG and/or IgM | Blood | Total | - | 71  (61, 80) | 96  (87, 100) | 97.2 (89.3-99.5) | 64.6 (52.9-74.8) | - | - |
|  | P: 40/0 |  |  |  |  |  | ≤7 |  | 55  (38, 71) | NA | - | - |  |  |
|  | P: 57/0 |  |  |  |  |  | >7 |  | 82  (70, 91) | NA |  |  |  |  |
| Spicuzza et al. (153) | P: 23/14 | immunochromatographic assay (IC) (POC) | 2019-nCoV IgG/IgM Antibody Rapid Test Kit (Beijing Diagreat Biotechnologies Co., Ltd) | - | IgG and/or IgM | Blood  Median: Case: 18  suspected: 9 | Unspecified | - | 83  (61, 95) | 93  (66, 100) | - | - | - | - |
| Wu et al. (161) | P: 0/1021  (Resuming Group) | Solid-phase immunochromatography | 2019‐nCoV Ab Test (Colloidal Gold)  (Beijing Innovita Biological Technology Co, Ltd) | - | IgG and/or IgM (but patients appear IgG positive only) | - | Unspecified | - | - | 90  (88, 92) | - | - | - | - |
|  | P: 1/380  (Hospitalized Group) |  |  |  | IgG and IgM |  |  |  | 100  (3, 100) | 100  (99, 100) |  |  |  |  |
|  | P: 1/380  (Hospitalized Group) |  |  |  | IgG and/or IgM (but patients appear IgG positive only) |  |  |  | 100  (3, 100) | 90  (86, 93) |  |  |  |  |
| Xu et al. (166) | P: 4/2 | GICA (POC) | Hotgen Biotech Co Ltd, Beijing, China | - | IgM | - | Unspecified | - | 100  (40, 100) | 50  (1, 99) | - | - | - | - |
| Zhang et al. (171) | P: 122/692 | colloidal gold immunochromatography assay (POC) | - | rS-RBD and rS1 proteins | IgG and IgM | Serum | Unspecified | - | 87  (80, 92) | 96  (95, 98) | - | - | - | - |
| **Antigen Tests (POC)** | | | | | | | | | | | | | | |
| Diao et al. (105) # | P:56/31  (Ct≤30) | Fluorescence Immunochromatographic assay (POC) | - | N protein (N antigen) | | Nasopharyngeal | Unspecified | - | 98  (90, 100) | 100  (89, 100) | 100 | 97 | 99 |  |
| Mertens et al. (155) # | S: 328 (total) | immunochromatographic assay (POC) | Ag Respi-Strip | Nucleocapsid protein antigen | | Nasopharyngeal | Unspecified | CT < 22 | 57.6 | 99.5 | 98.7 | 77.7 | 82.6 | - |
| **Laboratory and Clinical Feature** | | | | | | | | | | | | | | |
| Brinati et al. (94) # | NR | Laboratory parameters & Clinical features w/o gender (Leukocytes, platelets, C-reactive Protein, transaminases (AST), transaminases (ALT), Gamma Glutamil Transferasi (GGT), lactose dehydrogenase (LDH), neutrophils, lymphocytes, monocytes, eosinophils, basophils, age) | Random Forest Model | - | | - | | - | 92 | 65 | 83 | - | 82 | - |
|  | NR | Laboratory parameters & Clinical features, unsure if gender is included (Leukocytes, platelets, C-reactive Protein, transaminases (AST), transaminases (ALT), Gamma Glutamil Transferasi (GGT), lactose dehydrogenase (LDH), neutrophils, lymphocytes, monocytes, eosinophils, basophils, age) | Three-way Random Forest Model | - | | - | | - | 95 | 75 | 86 | - | 86 | - |
| Chen et al. (103) # | P: 19/19 | C Model; Model using clinical features (Respiration, heart rate, temperature, white blood cell count, cough, fatigue, lymphocyte count) | - | - | | - | | - | 84.2 | 85.9 | - | - | 86.8 | - |
| Kurstjens et al. (48) | P: 393/199 | Corona-score (clinical features) | - | Lab measurers (CRP, ALC, ANC, LDH, Ferritin), age, sex, CXR/CT | | Nasopharyngeal, Pharyngeal, Venous blood | | 9 | 78  (73, 82) | 89  (84, 93) | - | - | - | - |
| Li et al. (49) | P: 52/53 | Fever + respiratory symptoms + eosinopenia (<0.02) | - | Blood | | - | | - | 79  (65, 89) | 64  (50, 77) | 68.3 | 75.6 | - | - |
| Meng et al. (47) | S: 79/66 | Smartphone application based on clinical and demographic indicators | COVID-19 Diagnosis Aid APP | age, Activated Partial Thromboplastin Time, Red Blood Cell Distribution Width-SD, Uric Acid, Triglyceride, Serum Potassium, Albumin/globulin, 3-Hydroxybutyrate, Serum Calcium | | - | | - | 87  (78, 94) | 83  (72, 91) | 86.25 | 84.62 | - | - |
| Wu et al. (46) | P: 5/18  S:15/18  (Test Set) | Laboratory parameters (Total protein, glucose, calcium, creatine kinase isoenzyme, magnesium, basophil, bilirubin, creatinine, lactase dehydrogenase, kalium, platelet distribution width) | COVID-19 Assistant Discrimination 2.0 | Laboratory parameters | | Blood | | - | 100 | 94.4 |  |  | 97 |  |
|  | S:40/34  (External Validation set) |  |  |  |  |  |  | 0.43 | 95.1 | 97 |  |  | 96 |  |
| **Imaging & Clinical Features** | | | | | | | | | | | | | | |
| Chen et al. (103) # | P: 19/19 | CR Model; Model using clinical and radiological features (Total number of mixed GGO in peripheral area, tree-in-bud, offending vessel augmentation in lesions, repiration, heart ratio, temperature, WBC count, cough, fatigue, lymphocyte category) | - | - | | - | | - | 73.7 | 78.9 | - | - | 76.3 | - |
| **Serology (POC) + Imaging** | | | | | | | | | | | | | | |
| Imai et al. (118) | P: 112/0 | IC (POC) & CT | IC (POC): One Step Novel Coronavirus (COVID-19) IgM/IgG Antibody Test (Artron, Burnaby, Canada) | - | IgG and IgM | Serum | Overall | - | 78  (69, 85) | NA | - | - | - | - |
| **NAAT + Imaging** | | | | | | | | | | | | | | |
| Jiang et al. (121) | P: 87/481  S (CT):81/283  S(PCR): 623/792 | PCR + CT | - | - | | - | | - | 91  (83, 96) | 67  (61, 72) | 62.2 | 97.9 | - | - |
| Long et al. (134) | P: 36/0 | PCR + CT | - | - | | - | | - | 81  (64, 92) | NA | - | - | - | - |
| Wong et al. (160)^#^ | P: 64/0 | X-ray (Radiographic Assessment of Lung Edema score) + PCR | - | NA | | - | | Extent of involvement by consolidation or GGO tabulated as CXR severity score. baseline CXR severity score >0 considered positive | 59  (46, 71) | NA | - | - | - | - |
| Xie et al. (164) | P: 167/0 | CT + PCR | - | NA | | - | | - | 93  (88, 96) | NA | - | - | - | - |
| **Combination of Serology** | | | | | | | | | | | | | | |
| Lin et al. (130) | P: 65/64 | Combined ELISA and Chemiluminescence | ELISA: Darui Biotech | Nucleocapsid Antigen | IgM | Blood | Unspecified | - | 38  (27, 51) | 98  (92, 100) | - | - | - | - |
|  |  |  |  |  | IgG |  |  |  | 23  (14, 35) | 100  (94, 100) |  |  |  |  |
| **Serology + Serology (POC)** | | | | | | | | | | | | | | |
| Lou et al. (136) | P: 80/209 | Combined ELISA + CMIA+ LFIA (POC) | - | S Protein | Ab | Blood | Total | - | 99  (93, 100) | 94  (90, 97) | - | - | - | - |
|  | P: 80/209 |  |  |  | IgM |  |  |  | 94  (86, 98) | 97  (94, 99) |  |  |  |  |
|  | P: 80/100 |  |  | N Protein | IgG |  |  |  | 94  (86, 98) | 99  (95, 100) |  |  |  |  |

NA: Not applicable

NR: Not Reported

# Study is not included in meta-analysis

*The table may not include all outcomes/ recruited subjects/ tests from the included studies.

## **Table S4: Quality Assessment of Diagnostic Accuracy Studies 2 (QUADAS-2) checklist**

| **Domain 1: Patient Selection** | |
| --- | --- |
| Describe methods of patient selection |  |
| Was a consecutive or random sample of patients enrolled? | Yes/ No/ Unclear |
| Was a case-control design avoided? | Yes/ No |
| Did the study avoid inappropriate exclusions? | Yes/ No/ Unclear |
| Did the study avoid inappropriate inclusions? | Yes/ No/ Unclear |
| Risk of bias: Could the selection of patients have introduced bias? | High/ Low/ Unclear |
| Describe included patients (prior testing, presentation, intended use of index test and setting): |  |
| Applicability: Are there concerns that the included patients do not match the review question? | High/ Low/ Unclear |
| **Domain 2: Index Test** | |
| Describe the index test and how it was conducted and interpreted: |  |
| Were the index test results interpreted without knowledge of the results of the reference standard? | Yes/ No/ Unclear |
| If a threshold was used, was it pre-specified? | Yes/ No/ Unclear |
| Risk of bias: Could the conduct or interpretation of the index test have introduced bias? | High/ Low/ Unclear |
| Applicability: Are there concerns that the index test, its conduct, or interpretation differ from the review question? | High/ Low/ Unclear |
| **Domain 3: Reference Standard** | |
| Describe the reference standard and how it was conducted and interpreted: |  |
| Is the reference standard likely to correctly classify the target condition? | Yes/ No/ Unclear |
| Were the reference standard results interpreted without knowledge of the results of the index test? | Yes/ No/ Unclear |
| The definition of the reference standard did not incorporate results from the index test(s)? | Yes/ No/ Unclear/ Na (Imaging) |
| Risk of bias: Could the reference standard, its conduct, or its interpretation have introduced bias? | High/ Low/ Unclear |
| Applicability: Are there concerns that the target condition as defined by the reference standard does not match the review question? | High/ Low/ Unclear |
| **Domain 4: Flow and Timing** | |
| Describe any patients who did not receive the index test(s) and/or reference standard or who were excluded from the 2x2 table (refer to flow diagram): |  |
| Describe the time interval and any interventions between index test(s) and reference standard: |  |
| Was there an appropriate interval between index test(s) and reference standard? | Yes/ No/ Unclear |
| Did all patients receive a reference standard? | Yes/ No/ Unclear |
| Were all patients included in the analysis? | Yes/ No/ Unclear |
| Risk of bias: Could the patient flow have introduced bias? | High/ Low/ Unclear |

## **Table S5: Studies Excluded during Full-Text Screening**

| **Population** | |
| --- | --- |
| **1** | Ackerman, C. M., Myhrvold, C., Thakku, S. G., Freije, C. A., Metsky, H. C., Yang, D. K., . . . Sabeti, P. C. (2020). Massively multiplexed nucleic acid detection using Cas13. Nature. doi:10.1038/s41586-020-2279-8 |
| **2** | Al-karawi, D., Al-Zaidi, S., Polus, N., & Jassim, S. (2020). Machine Learning Analysis of Chest CT Scan Images as a Complementary Digital Test of Coronavirus (COVID-19) Patients. medRxiv, 2020.2004.2013.20063479. doi:10.1101/2020.04.13.20063479 |
| **3** | Annamalai, P., Kanta, M., Ramu, P., Ravi, B., Veerapandian, K., & Srinivasan, R. (2020). A SIMPLE COLORIMETRIC MOLECULAR DETECTION OF NOVEL CORONAVIRUS (COVID-19), AN ESSENTIAL DIAGNOSTIC TOOL FOR PANDEMIC SCREENING. medRxiv, 2020.2004.2010.20060293. doi:10.1101/2020.04.10.20060293 |
| **4** | Barra, G. B., Santa Rita, T. H., Mesquita, P. G., Jacomo, R. H., & Nery, L. F. A. (2020). Analytical sensibility and specificity of two RT-qPCR protocols for SARS-CoV-2 detection performed in an automated workflow. medRxiv, 2020.2003.2007.20032326. doi:10.1101/2020.03.07.20032326 |
| **5** | Corman, V. M., Landt, O., Kaiser, M., Molenkamp, R., Meijer, A., Chu, D. K. W., . . . Drosten, C. (2020). Detection of 2019 novel coronavirus (2019-nCoV) by real-time RT-PCR. Eurosurveillance, 25(3). doi:10.2807/1560-7917.ES.2020.25.3.2000045 |
| **6** | Ding, X., Yin, K., Li, Z., & Liu, C. (2020). All-in-One Dual CRISPR-Cas12a (AIOD-CRISPR) Assay: A Case for Rapid, Ultrasensitive and Visual Detection of Novel Coronavirus SARS-CoV-2 and HIV virus. bioRxiv, 2020.2003.2019.998724. doi:10.1101/2020.03.19.998724 |
| **7** | Eis-Hübinger, A. M., Hönemann, M., Wenzel, J. J., Berger, A., Widera, M., Schmidt, B., . . . Panning, M. (2020). Ad hoc laboratory-based surveillance of SARS-CoV-2 by real-time RT-PCR using minipools of RNA prepared from routine respiratory samples. Journal of Clinical Virology, 127. doi:10.1016/j.jcv.2020.104381 |
| **8** | Gonzalez-Gonzalez, E., Trujillo-de Santiago, G., Lara-Mayorga, I. M., Martinez-Chapa, S. O., & Alvarez, M. M. (2020). Portable and accurate diagnostics for COVID-19: Combined use of the miniPCR thermocycler and a well-plate reader for SARS-CoV-2 virus detection. medRxiv, 2020.2004.2003.20052860. doi:10.1101/2020.04.03.20052860 |
| **9** | Guo, L., Sun, X., Wang, X., Liang, C., Jiang, H., Gao, Q., . . . Li, W. (2020). SARS-CoV-2 detection with CRISPR diagnostics. bioRxiv, 2020.2004.2010.023358. doi:10.1101/2020.04.10.023358 |
| **10** | Hu, K., Patel, J., & Patel, B. C. (2020). Ophthalmic Manifestations Of Coronavirus (COVID-19). In StatPearls. Treasure Island (FL): StatPearls Publishing |
| **11** | Jung, Y. J., Park, G.-S., Moon, J. H., Ku, K., Beak, S.-H., Kim, S., . . . Kim, H. G. (2020). Comparative analysis of primer-probe sets for the laboratory confirmation of SARS-CoV-2. bioRxiv, 2020.2002.2025.964775. doi:10.1101/2020.02.25.964775 |
| **12** | Nelson, A. C., Auch, B., Schomaker, M., Gohl, D. M., Grady, P., Johnson, D., . . . Yohe, S. (2020). Analytical Validation of a COVID-19 qRT-PCR Detection Assay Using a 384-well Format and Three Extraction Methods. bioRxiv, 2020.2004.2002.022186. doi:10.1101/2020.04.02.022186 |
| **13** | Park, G. S., Ku, K., Baek, S. H., Kim, S. J., Kim, S. I., Kim, B. T., & Maeng, J. S. (2020). Development of Reverse Transcription Loop-Mediated Isothermal Amplification Assays Targeting SARS-CoV-2. J Mol Diagn. doi:10.1016/j.jmoldx.2020.03.006 |
| **14** | Rabe, B. A., & Cepko, C. (2020). SARS-CoV-2 Detection Using an Isothermal Amplification Reaction and a Rapid, Inexpensive Protocol for Sample Inactivation and Purification. medRxiv, 2020.2004.2023.20076877. doi:10.1101/2020.04.23.20076877 |
| **15** | Tan, X., Lin, C., Zhang, J., Khaing Oo, M. K., & Fan, X. (2020). Rapid and quantitative detection of COVID-19 markers in micro-liter sized samples. bioRxiv, 2020.2004.2020.052233. doi:10.1101/2020.04.20.052233 |
| **16** | Wang, D. (2020). One-pot Detection of COVID-19 with Real-time Reverse-transcription Loop-mediated Isothermal Amplification (RT-LAMP) Assay and Visual RT-LAMP Assay. bioRxiv, 2020.2004.2021.052530. doi:10.1101/2020.04.21.052530 |
| **17** | Woo, C. H., Jang, S., Shin, G., Jung, G. Y., & Lee, J. W. (2020). Sensitive one-step isothermal detection of pathogen-derived RNAs. medRxiv, 2020.2003.2005.20031971. doi:10.1101/2020.03.05.20031971 |
| **18** | Xiang, J., Yan, M., Li, H., Liu, T., Lin, C., Huang, S., & Shen, C. (2020). Evaluation of Enzyme-Linked Immunoassay and Colloidal Gold- Immunochromatographic Assay Kit for Detection of Novel Coronavirus (SARS-Cov-2) Causing an Outbreak of Pneumonia (COVID-19). medRxiv, 2020.2002.2027.20028787. doi:10.1101/2020.02.27.20028787 |
| **19** | Yong, G., Yi, Y., Tuantuan, L., Xiaowu, W., Xiuyong, L., Ang, L., & Mingfeng, H. (2020). Evaluation of the auxiliary diagnostic value of antibody assays for the detection of novel coronavirus (SARS-CoV-2). J Med Virol. doi:10.1002/jmv.25919 |
| **20** | Beal, S. G., Posa, M., Gaffar, M., Reppucci, J., Mack, J. A., Gurka, M. J., . . . Kelly, M. N. (2020). Performance and Impact of a CLIA-waived, Point-of-care Respiratory PCR Panel in a Pediatric Clinic. Pediatr Infect Dis J, 39(3), 188-191. doi:10.1097/inf.0000000000002544 |
| **21** | Himoto Y, Sakata A, Kirita M, Hiroi T, Kobayashi KI, Kubo K, et al. Diagnostic performance of chest CT to differentiate COVID-19 pneumonia in non-high-epidemic area in Japan |
| **Intervention** | |
| **1** | Agostini, A., Floridi, C., Borgheresi, A., Badaloni, M., Esposto Pirani, P., Terilli, F., . . . Giovagnoni, A. (2020). Proposal of a low-dose, long-pitch, dual-source chest CT protocol on third-generation dual-source CT using a tin filter for spectral shaping at 100 kVp for CoronaVirus Disease 2019 (COVID-19) patients: a feasibility study. Radiol Med, 125(4), 365-373. doi:10.1007/s11547-020-01179-x |
| **2** | Arumugam, A., Faron, M. L., Yu, P., Markham, C., & Wong, S. (2020). A Rapid COVID-19 RT-PCR Detection Assay for Low Resource Settings. bioRxiv, 2020.2004.2029.069591. doi:10.1101/2020.04.29.069591 |
| **3** | Chate, R. C., Fonseca, E., Passos, R. B. D., Teles, G., Shoji, H., & Szarf, G. (2020). Presentation of pulmonary infection on CT in COVID-19: initial experience in Brazil. J Bras Pneumol, 46(2), e20200121. doi:10.36416/1806-3756/e20200121 |
| **4** | Fang, Y., Zhang, H., Xie, J., Lin, M., Ying, L., Pang, P., & Ji, W. (2020). Sensitivity of Chest CT for COVID-19: Comparison to RT-PCR. Radiology, 200432. doi:10.1148/radiol.2020200432 |
| **5** | Kavsak, P. A., De Wit, K., & Worster, A. (2020). Clinical chemistry tests for patients with COVID-19-important caveats for interpretation. Clin Chem Lab Med. doi:10.1515/cclm-2020-0436 |
| **6** | Lim, Z. Y., Khoo, H. W., Hui, T. C. H., Kok, S. S. X., Kwan, K. E. L., Young, B. E., . . . Kaw, G. J. L. (2020). Variable computed tomography appearances of COVID-19. Singapore Med J. doi:10.11622/smedj.2020066 |
| **7** | Lu, W., Zhang, S., Chen, B., Chen, J., Xian, J., Lin, Y., . . . Su, Z. Z. (2020). A Clinical Study of Noninvasive Assessment of Lung Lesions in Patients with Coronavirus Disease-19 (COVID-19) by Bedside Ultrasound. Ultraschall Med. doi:10.1055/a-1154-8795 |
| **8** | Singh, D., Kumar, V., Vaishali, & Kaur, M. (2020). Classification of COVID-19 patients from chest CT images using multi-objective differential evolution-based convolutional neural networks. Eur J Clin Microbiol Infect Dis. doi:10.1007/s10096-020-03901-z |
| **9** | Yasukawa, K., & Minami, T. (2020). Point-of-Care Lung Ultrasound Findings in Patients with Novel Coronavirus Disease (COVID-19) Pneumonia. Am J Trop Med Hyg. doi:10.4269/ajtmh.20-0280 |
| **10** | Huang, P., Liu, T., Huang, L., Liu, H., Lei, M., Xu, W., . . . Liu, B. (2020). Use of Chest CT in Combination with Negative RT-PCR Assay for the 2019 Novel Coronavirus but High Clinical Suspicion. Radiology, 295(1), 22-23. doi:10.1148/radiol.2020200330 |
| **11** | Lu, X., Zhang, L., Du, H., Zhang, J., Li, Y. Y., Qu, J., . . . Li, Y. (2020). SARS-CoV-2 infection in children. New England Journal of Medicine, 382(17), 1663-1665. |
| **12** | Ng, M.-Y., Lee, E. Y. P., Yang, J., Yang, F., Li, X., Wang, H., . . . Kuo, M. D. (2020). Imaging Profile of the COVID-19 Infection: Radiologic Findings and Literature Review. Radiology: Cardiothoracic Imaging, 2(1), e200034. doi:10.1148/ryct.2020200034 |
| **13** | Poggiali, E., Dacrema, A., Bastoni, D., Tinelli, V., Demichele, E., Mateo Ramos, P., . . . Magnacavallo, A. (2020). Can Lung US Help Critical Care Clinicians in the Early Diagnosis of Novel Coronavirus (COVID-19) Pneumonia? Radiology, 295(3), E6. doi:10.1148/radiol.2020200847 |
| **14** | To, K. K.-W., Tsang, O. T.-Y., Leung, W.-S., Tam, A. R., Wu, T.-C., Lung, D. C., . . . Yuen, K.-Y. (2020). Temporal profiles of viral load in posterior oropharyngeal saliva samples and serum antibody responses during infection by SARS-CoV-2: an observational cohort study. The Lancet Infectious Diseases, 20(5), 565-574. doi:10.1016/S1473-3099(20)30196-1 |
| **15** | Wan, S., Xiang, Y., Fang, W., Zheng, Y., Li, B., Hu, Y., . . . Yang, R. (2020). Clinical features and treatment of COVID-19 patients in northeast Chongqing. J Med Virol, 92(7), 797-806. doi:10.1002/jmv.25783 |
| **16** | Wang, L., Gao, Y.-h., Lou, L.-L., & Zhang, G.-J. (2020). The clinical dynamics of 18 cases of COVID-19 outside of Wuhan, China. European Respiratory Journal, 55(4), 2000398. doi:10.1183/13993003.00398-2020 |
| **17** | Xia, W., Shao, J., Guo, Y., Peng, X., Li, Z., & Hu, D. (2020). Clinical and CT features in pediatric patients with COVID-19 infection: Different points from adults. Pediatric Pulmonology, 55(5), 1169-1174. doi:10.1002/ppul.24718 |
| **18** | Xiao, S.-Y., Wu, Y., & Liu, H. (2020). Evolving status of the 2019 novel coronavirus infection: Proposal of conventional serologic assays for disease diagnosis and infection monitoring. Journal of Medical Virology, 92(5), 464-467. doi:10.1002/jmv.25702 |
| **19** | Zhang, S., Li, H., Huang, S., You, W., & Sun, H. (2020). High-resolution computed tomography features of 17 cases of coronavirus disease 2019 in Sichuan province, China. European Respiratory Journal, 55(4). |
| **20** | Zhu, W., Xie, K., Lu, H., Xu, L., Zhou, S., & Fang, S. Initial clinical features of suspected coronavirus disease 2019 in two emergency departments outside of Hubei, China. Journal of Medical Virology, n/a(n/a). doi:10.1002/jmv.25763 |
| **21** | Elghamrawy SM, Hassanien AE. Diagnosis and Prediction Model for COVID19 Patients Response to Treatment based on Convolutional Neural Networks and Whale Optimization Algorithm Using CT Images |
| **22** | Xu X, Yu C, Qu J, Zhang L, Jiang S, Huang D, et al. Imaging and clinical features of patients with 2019 novel coronavirus SARS-CoV-2 |
| **23** | Yang W, Cao Q, Qin L, Wang X, Cheng Z, Pan A, et al. Clinical characteristics and imaging manifestations of the 2019 novel coronavirus disease (COVID-19):A multi-center study in Wenzhou city, Zhejiang, China |
| **Outcome** | |
| **1** | Amanat, F., Stadlbauer, D., Strohmeier, S., Nguyen, T., Chromikova, V., McMahon, M., . . . Moran, T. (2020). A serological assay to detect SARS-CoV-2 seroconversion in humans. medRxiv, 2020.2003.2017.20037713. doi:10.1101/2020.03.17.20037713 |
| **2** | Brown, J. R., O’Sullivan, D., Pereira, R. P. A., Whale, A. S., Busby, E., Huggett, J., & Harris, K. (2020). Comparison of SARS-CoV2 N gene real-time RT-PCR targets and commercially available mastermixes. bioRxiv, 2020.2004.2017.047118. doi:10.1101/2020.04.17.047118 |
| **3** | Bruce, E. A., Huang, M.-L., Perchetti, G. A., Tighe, S., Laaguiby, P., Hoffman, J. J., . . . Botten, J. W. (2020). DIRECT RT-qPCR DETECTION OF SARS-CoV-2 RNA FROM PATIENT NASOPHARYNGEAL SWABS WITHOUT AN RNA EXTRACTION STEP. bioRxiv, 2020.2003.2020.001008. doi:10.1101/2020.03.20.001008 |
| **4** | Buonsenso, D., Raffaelli, F., Tamburrini, E., Biasucci, D. G., Salvi, S., Smargiassi, A., . . . Moro, F. (2020). Clinical role of lung ultrasound for the diagnosis and monitoring of COVID-19 pneumonia in pregnant women. Ultrasound Obstet Gynecol. doi:10.1002/uog.22055 |
| **5** | He, X., Yang, X., Zhang, S., Zhao, J., Zhang, Y., Xing, E., & Xie, P. (2020). Sample-Efficient Deep Learning for COVID-19 Diagnosis Based on CT Scans. medRxiv, 2020.2004.2013.20063941. doi:10.1101/2020.04.13.20063941 |
| **6** | Ishige, T., Murata, S., Taniguchi, T., Miyabe, A., Kitamura, K., Kawasaki, K., . . . Matsushita, K. (2020). Highly sensitive detection of SARS-CoV-2 RNA by multiplex rRT-PCR for molecular diagnosis of COVID-19 by clinical laboratories. Clin Chim Acta, 507, 139-142. doi:10.1016/j.cca.2020.04.023 |
| **7** | Kelly, J. C., Dombrowksi, M., O'Neil-Callahan, M., Kernberg, A. S., Frolova, A. I., & Stout, M. J. (2020). False-Negative COVID-19 Testing: Considerations in Obstetrical Care. Am J Obstet Gynecol MFM, 100130. doi:10.1016/j.ajogmf.2020.100130 |
| **8** | Konrad, R., Eberle, U., Dangel, A., Treis, B., Berger, A., Bengs, K., . . . Sing, A. (2020). Rapid establishment of laboratory diagnostics for the novel coronavirus SARS-CoV-2 in Bavaria, Germany, February 2020. Eurosurveillance, 25(9). doi:10.2807/1560-7917.ES.2020.25.9.2000173 |
| **9** | Li, Y., & Xia, L. (2020). Coronavirus Disease 2019 (COVID-19): Role of Chest CT in Diagnosis and Management. AJR. American journal of roentgenology, 1-7. doi:10.2214/AJR.20.22954 |
| **10** | Lieberman, J., Pepper, G., Naccache, S. N., Huang, M., Jerome, K. R., & Greninger, A. L. (2020). Comparison of Commercially Available and Laboratory Developed Assays for in vitro Detection of SARS-CoV-2 in Clinical Laboratories. medRxiv, 2020.2004.2024.20074559. doi:10.1101/2020.04.24.20074559 |
| **11** | Liu, J., Yu, H., & Zhang, S. (2020). The indispensable role of chest CT in the detection of coronavirus disease 2019 (COVID-19). European Journal of Nuclear Medicine and Molecular Imaging. doi:10.1007/s00259-020-04795-x |
| **12** | Liu, W. D., Chang, S. Y., Wang, J. T., Tsai, M. J., Hung, C. C., Hsu, C. L., & Chang, S. C. (2020). Prolonged virus shedding even after seroconversion in a patient with COVID-19. J Infect. doi:10.1016/j.jinf.2020.03.063 |
| **13** | Okba, N. M. A., Müller, M. A., Li, W., Wang, C., GeurtsvanKessel, C. H., Corman, V. M., . . . Haagmans, B. L. (2020). Severe Acute Respiratory Syndrome Coronavirus 2-Specific Antibody Responses in Coronavirus Disease 2019 Patients. Emerg Infect Dis, 26(7). doi:10.3201/eid2607.200841 |
| **14** | Perera, R. A., Mok, C. K., Tsang, O. T., Lv, H., Ko, R. L., Wu, N. C., . . . Peiris, M. (2020). Serological assays for severe acute respiratory syndrome coronavirus 2 (SARS-CoV-2), March 2020. Euro Surveill, 25(16). doi:10.2807/1560-7917.es.2020.25.16.2000421 |
| **15** | Pfefferle, S., Reucher, S., Nörz, D., & Lütgehetmann, M. (2020). Evaluation of a quantitative RT-PCR assay for the detection of the emerging coronavirus SARS-CoV-2 using a high throughput system. Eurosurveillance, 25(9). doi:10.2807/1560-7917.ES.2020.25.9.2000152 |
| **16** | Prokop, M., van Everdingen, W., van Rees Vellinga, T., Quarles van Ufford, J., Stöger, L., Beenen, L., . . . Brink, M. (2020). CO-RADS - A categorical CT assessment scheme for patients with suspected COVID-19: definition and evaluation. Radiology, 201473. doi:10.1148/radiol.2020201473 |
| **17** | Saito, M., Adachi, E., Yamayoshi, S., Koga, M., Iwatsuki-Horimoto, K., Kawaoka, Y., & Yotsuyanagi, H. (2020). Gargle lavage as a safe and sensitive alternative to swab samples to diagnose COVID-19: a case report in Japan. Clin Infect Dis. doi:10.1093/cid/ciaa377 |
| **18** | Seo, G., Lee, G., Kim, M. J., Baek, S. H., Choi, M., Ku, K. B., . . . Kim, S. I. (2020). Rapid Detection of COVID-19 Causative Virus (SARS-CoV-2) in Human Nasopharyngeal Swab Specimens Using Field-Effect Transistor-Based Biosensor. ACS Nano, 14(4), 5135-5142. doi:10.1021/acsnano.0c02823 |
| **19** | Sun, Y., Koh, V., Marimuthu, K., Ng, O. T., Young, B., Vasoo, S., . . . Leo, Y. S. (2020). Epidemiological and Clinical Predictors of COVID-19. Clin Infect Dis. doi:10.1093/cid/ciaa322 |
| **20** | Toptan, T., Hoehl, S., Westhaus, S., Bojkova, D., Berger, A., Rotter, B., . . . Widera, M. (2020). Optimized qRT-PCR approach for the detection of intra- and extra-cellular SARS-CoV-2 RNAs. bioRxiv, 2020.2004.2020.052258. doi:10.1101/2020.04.20.052258 |
| **21** | Vogels, C. B. F., Brito, A. F., Wyllie, A. L., Fauver, J. R., Ott, I. M., Kalinich, C. C., . . . Grubaugh, N. D. (2020). Analytical sensitivity and efficiency comparisons of SARS-COV-2 qRT-PCR primer-probe sets. medRxiv, 2020.2003.2030.20048108. doi:10.1101/2020.03.30.20048108 |
| **22** | Wan, W. Y., Lim, S. H., & Seng, E. H. (2020). Cross-reaction of sera from COVID-19 patients with SARS-CoV assays. medRxiv, 2020.2003.2017.20034454. doi:10.1101/2020.03.17.20034454 |
| **23** | Wang, K., Kang, S., Tian, R., Zhang, X., & Wang, Y. (2020). Imaging manifestations and diagnostic value of chest CT of coronavirus disease 2019 (COVID-19) in the Xiaogan area. Clinical Radiology, 75(5), 341-347. doi:10.1016/j.crad.2020.03.004 |
| **24** | Wang, M., Wu, Q., Xu, W., Qiao, B., Wang, J., Zheng, H., . . . Li, Y. (2020). Clinical diagnosis of 8274 samples with 2019-novel coronavirus in Wuhan. medRxiv, 2020.2002.2012.20022327. doi:10.1101/2020.02.12.20022327 |
| **25** | Woelfel, R., Corman, V. M., Guggemos, W., Seilmaier, M., Zange, S., Mueller, M. A., . . . Wendtner, C. (2020). Clinical presentation and virological assessment of hospitalized cases of coronavirus disease 2019 in a travel-associated transmission cluster. medRxiv, 2020.2003.2005.20030502. doi:10.1101/2020.03.05.20030502 |
| **26** | Zhang, Z., Shen, Y., Wang, H., Zhao, L., & Hu, D. (2020). High-resolution computed tomographic imaging disclosing COVID-19 pneumonia: A powerful tool in diagnosis. Journal of Infection. doi:10.1016/j.jinf.2020.03.047 |
| **27** | Zheng, C., Deng, X., Fu, Q., Zhou, Q., Feng, J., Ma, H., . . . Wang, X. (2020). Deep Learning-based Detection for COVID-19 from Chest CT using Weak Label. medRxiv, 2020.2003.2012.20027185. doi:10.1101/2020.03.12.20027185 |
| **28** | Zhou, F., Yu, X., Tong, X., & Zhang, R. (2020). Clinical features and outcomes of 197 adult discharged patients with COVID-19 in Yichang, Hubei. medRxiv, 2020.2003.2026.20041426. doi:10.1101/2020.03.26.20041426 |
| **29** | Bernheim, A., Mei, X., Huang, M., Yang, Y., Fayad, Z. A., Zhang, N., . . . Chung, M. (2020). Chest CT Findings in Coronavirus Disease-19 (COVID-19): Relationship to Duration of Infection. Radiology, 295(3), 200463. doi:10.1148/radiol.2020200463 |
| **30** | Chen, L., Liu, W., Zhang, Q., Xu, K., Ye, G., Wu, W., . . . Liu, Y. (2020). RNA based mNGS approach identifies a novel human coronavirus from two individual pneumonia cases in 2019 Wuhan outbreak. Emerg Microbes Infect, 9(1), 313-319. doi:10.1080/22221751.2020.1725399 |
| **31** | Cheng, Z., Lu, Y., Cao, Q., Qin, L., Pan, Z., Yan, F., & Yang, W. (2020). Clinical Features and Chest CT Manifestations of Coronavirus Disease 2019 (COVID-19) in a Single-Center Study in Shanghai, China. American Journal of Roentgenology, 215(1), 121-126. doi:10.2214/AJR.20.22959 |
| **32** | Guan, W.-j., Ni, Z.-y., Hu, Y., Liang, W.-h., Ou, C.-q., He, J.-x., . . . Zhong, N.-s. (2020). Clinical Characteristics of Coronavirus Disease 2019 in China. New England Journal of Medicine, 382(18), 1708-1720. doi:10.1056/NEJMoa2002032 |
| **33** | Pan, Y., Zhang, D., Yang, P., Poon, L. L. M., & Wang, Q. (2020). Viral load of SARS-CoV-2 in clinical samples. The Lancet. Infectious diseases, 20(4), 411-412. doi:10.1016/S1473-3099(20)30113-4 |
| **34** | Zhang, W., Du, R.-H., Li, B., Zheng, X.-S., Yang, X.-L., Hu, B., . . . Zhou, P. (2020). Molecular and serological investigation of 2019-nCoV infected patients: implication of multiple shedding routes. Emerg Microbes Infect, 9(1), 386-389. doi:10.1080/22221751.2020.1729071 |
| **35** | Yang W, Dang X, Wang Q, Xu M, Zhao Q, Zhou Y, et al. Rapid Detection of SARS-CoV-2 Using Reverse transcription RT-LAMP method |
| **Comparator** | |
| **1** | Alcoba-Florez, J., Gonzalez-Montelongo, R., Inigo-Campos, A., Garcia-Martinez de Artola, D., Gil-Campesino, H., Ciuffreda, L., . . . Flores, C. (2020). Fast SARS-CoV-2 detection by RT-qPCR in preheated nasopharyngeal swab samples. medRxiv, 2020.2004.2008.20058495. doi:10.1101/2020.04.08.20058495 |
| **2** | Bukhari, S. U. K., Bukhari, S. S. K., Syed, A., & Shah, S. S. H. (2020). The diagnostic evaluation of Convolutional Neural Network (CNN) for the assessment of chest X-ray of patients infected with COVID-19. medRxiv, 2020.2003.2026.20044610. doi:10.1101/2020.03.26.20044610 |
| **3** | Chu, D. K. W., Pan, Y., Cheng, S. M. S., Hui, K. P. Y., Krishnan, P., Liu, Y., . . . Poon, L. L. M. (2020). Molecular Diagnosis of a Novel Coronavirus (2019-nCoV) Causing an Outbreak of Pneumonia. Clin Chem, 66(4), 549-555. doi:10.1093/clinchem/hvaa029 |
| **4** | Fomsgaard, A. S., & Rosenstierne, M. W. (2020). An alternative workflow for molecular detection of SARS-CoV-2 - escape from the NA extraction kit-shortage, Copenhagen, Denmark, March 2020. Euro Surveill, 25(14). doi:10.2807/1560-7917.ES.2020.25.14.2000398 |
| **5** | Gueguim Kana, E. B., Zebaze Kana, M. G., Donfack Kana, A. F., & Azanfack Kenfack, R. H. (2020). A web-based Diagnostic Tool for COVID-19 Using Machine Learning on Chest Radiographs (CXR). medRxiv, 2020.2004.2021.20063263. doi:10.1101/2020.04.21.20063263 |
| **6** | Hassanien, A. E., Mahdy, L. N., Ezzat, K. A., Elmousalami, H. H., & Aboul Ella, H. (2020). Automatic X-ray COVID-19 Lung Image Classification System based on Multi-Level Thresholding and Support Vector Machine. medRxiv, 2020.2003.2030.20047787. doi:10.1101/2020.03.30.20047787 |
| **7** | Khobahi, S., Agarwal, C., & Soltanalian, M. (2020). CoroNet: A Deep Network Architecture for Semi-Supervised Task-Based Identification of COVID-19 from Chest X-ray Images. medRxiv, 2020.2004.2014.20065722. doi:10.1101/2020.04.14.20065722 |
| **8** | Lei, P., Fan, B., Mao, J., & Wang, P. (2020). Multiple parameters required for diagnosis of COVID-19 in clinical practice. Journal of Infection. doi:10.1016/j.jinf.2020.03.016 |
| **9** | Nalla, A. K., Casto, A. M., Huang, M. W., Perchetti, G. A., Sampoleo, R., Shrestha, L., . . . Greninger, A. L. (2020). Comparative Performance of SARS-CoV-2 Detection Assays using Seven Different Primer/Probe Sets and One Assay Kit. J Clin Microbiol. doi:10.1128/jcm.00557-20 |
| **10** | Xiong, Y., Li, Z.-Z., Zhuang, Q.-Z., Chao, Y., Li, F., Ge, Y.-Y., . . . Huang, X.-Z. (2020). Comparative performance of four nucleic acid amplification tests for SARS-CoV-2 virus. bioRxiv, 2020.2003.2026.010975. doi:10.1101/2020.03.26.010975 |
| **11** | Yang, Y., Yang, M., Shen, C., Wang, F., Yuan, J., Li, J., . . . Liu, Y. (2020). Evaluating the accuracy of different respiratory specimens in the laboratory diagnosis and monitoring the viral shedding of 2019-nCoV infections. medRxiv, 2020.2002.2011.20021493. doi:10.1101/2020.02.11.20021493 |
| **12** | Zhao, R., Li, M., Song, H., Chen, J., Ren, W., Feng, Y., . . . Sun, L. (2020). Serological diagnostic kit of SARS-CoV-2 antibodies using CHO-expressed full-length SARS-CoV-2 S1 proteins. medRxiv, 2020.2003.2026.20042184. doi:10.1101/2020.03.26.20042184 |
| **13** | Gozes, O., Frid-Adar, M., Greenspan, H., Browning, P. D., Zhang, H., Ji, W., . . . Siegel, E. (2020). Rapid ai development cycle for the coronavirus (covid-19) pandemic: Initial results for automated detection & patient monitoring using deep learning ct image analysis. arXiv preprint arXiv:2003.05037. |
| **14** | Grzelak L, Temmam S, Planchais C, Demeret C, Huon C, Guivel F, et al. SARS-CoV-2 serological analysis of COVID-19 hospitalized patients, pauci-symptomatic individuals and blood donors |
| **Study Design** | |
| **1** | Adams, H. J. A., Kwee, T. C., & Kwee, R. M. (2020). COVID-19 and chest CT: do not put the sensitivity value in the isolation room and look beyond the numbers. Radiology, 201709. doi:10.1148/radiol.2020201709 |
| **2** | Zhifeng, J., Feng, A., & Li, T. (2020). Consistency analysis of COVID-19 nucleic acid tests and the changes of lung CT. Journal of Clinical Virology, 127. doi:10.1016/j.jcv.2020.104359 |
| **3** | Bai HX, Hsieh B, Xiong Z, Halsey K, Choi JW, Tran TML, et al. Performance of radiologists in differentiating COVID-19 from viral pneumonia on chest CT |
| **4** | Beltrán-Pavez C, Márquez CL, Muñoz G, Valiente-Echeverría F, Gaggero A, Soto-Rifo R, et al. SARS-CoV-2 detection from nasopharyngeal swab samples without RNA extraction |
| **Language** | |
| **1** | Xiong, Z., Fu, L., Zhou, H., Liu, J. K., Wang, A. M., Huang, Y., . . . Liao, W. H. (2020). Construction and evaluation of a novel diagnosis process for 2019-Corona Virus Disease. Zhonghua yi xue za zhi, 100, E019. doi:10.3760/cma.j.cn112137-20200228-00499 |
| **2** | Li, X., Liu, J., Liu, Q., Yu, L., Wu, S., & Yin, X. (2020). [Optimization of a fluorescent qPCR detection for RNA of SARS-CoV-2]. Sheng Wu Gong Cheng Xue Bao, 36(4), 732-739. doi:10.13345/j.cjb.200088 |
| **3** | Nie, W., Feng, Z., Mao, X., Rong, P., Wang, W., & Liang, Q. (2020). First CT characteristic appearance of patients with coronavirus disease 2019. Zhong Nan Da Xue Xue Bao Yi Xue Ban, 45(3), 262-268. doi:10.11817/j.issn.1672-7347.2020.200136 |

## **Table S6: Comparison of sensitivity and specificity estimates for Serology and Serology (POC) in early and late phase of disease**

| **Test** | **Early Phase ( ≤7 days)** | | | | | | **Late Phase (≥7 days)** | | | | | |
| --- | --- | --- | --- | --- | --- | --- | --- | --- | --- | --- | --- | --- |
|  | **No. of studies** | **Sensitivity** | **No. of studies** | **Specificity** | **DOR** | **SROC** | **No. of studies** | **Sensitivity** | **No. of studies** | **Specificity** | **DOR** | **SROC** |
| **Serology** | | | | | | | | | | | | |
| IgG and/or IgM | 2 | 67*  (35, 90)  51*  (38, 63) | 0 | - | - | - | 4 | 91  (89, 93) | 0 | - | - | - |
| IgG and IgM | 1 | 50*  (21, 79) | 0 | - | - | - | 1 | 60*  (47, 72) | 0 | - | - | - |
| IgG | 6 | 47  (29, 67) | 0 | - | - | - | 11 | 91  (81, 96) | 1 | 96*  (90, 99) | - | - |
| IgM | 6 | 43  (26, 62) | 0 | - | - | - | 10 | 85  (75, 91) | 1 | 94*  (85, 98) | - | - |
| Ab | 2 | 64*  (47, 79)  38*  (28, 49) | 0 | - | - | - | 7 | 97  (93, 99) | 4 | 100  (64, 100) | 506.3  (126.1, 2032.1) | 0.932 |
| **Serology (POC)** | | | | | | | | | | | | |
| IgG and/or IgM | 5 | 27  (16, 43) | 2 | 78*  (40, 97)  56*  (21, 86) |  |  | 18 | 83  (76, 88) | 7 | 98  (33, 100) | 65.4  (4.0, 1070.9) | 0.904 |
| IgG and IgM | 1 | 13*  (2, 38) | 1 | 89*  (52, 100) | - | - | 1 | 41*  (29, 53) | 1 | 75*  (48, 93) | - | - |
| IgG | 4 | 5  (2, 15) | 2 | 89* (52, 100)  56* (21, 86) | - | - | 5 | 69  (48, 84) | 4 | 77  (15, 98) | 9.7  (0.4, 214.9) | 0.784 |
| IgM | 4 | 18  (10, 32) | 2 | 100* (66, 100)  78* (40, 97) | - | - | 5 | 41  (11, 80) | 4 | 92  (44, 99) | 3.5  (0.4, 31.0) | 0.713 |

*results were not pooled due to insufficient studies

## **Table S7: Pooled sensitivity estimates for symptomatic and asymptomatic patients**

| **Tests** | **Symptomatic** | | **Asymptomatic** | |
| --- | --- | --- | --- | --- |
|  | **No. of Studies** | **Sensitivity** | **No. of Studies** | **Sensitivity** |
| Serology IgG and/or IgM | 9 | 90 (80, 95) | 2 | 100* (3, 100)  100* (16, 100) |
| Serology IgG and IgM | 4 | 74 (11, 98) | 2 | 0* (0, 98)  50* (1, 99) |
| Serology IgG | 14 | 82 (73, 89) | 2 | 0* (0, 98)  100* (16, 100) |
| Serology IgM | 16 | 82 (73, 88) | 2 | 100* (3, 100)  50* (1, 99) |
| Serology IgG and/or IgM (POC) | 7 | 66 (41, 84) | 1 | 39* (24, 57) |
| Serology IgG (POC) | 7 | 62 (26, 88) | 1 | 0* (0, 9) |
| Serology IgM (POC) | 8 | 45 (17, 76) | 2 | 39* (24, 57)  100* (3, 100) |
| NAAT | 5 | 99 (84, 100) | 2 | 100* (63, 100)  100* (3 100) |
| Imaging | 8 | 82 (67, 91) | 1 | 100* (90, 100) |

* results were not pooled due to insufficient studies

## **Table S8. Pooled sensitivity and specificity estimates for different diagnostic tests by geographical regions.**

|  | **Asia (Exc. China)** | | | | **America** | | | | **China** | | | | **Europe** | | | |
| --- | --- | --- | --- | --- | --- | --- | --- | --- | --- | --- | --- | --- | --- | --- | --- | --- |
|  | **Total no. of studies** | **Sensitivity** | **Total no. of studies** | **Specificity** | **Total no. of studies** | **Sensitivity** | **Total no. of studies** | **Specificity** | **Total no. of studies** | **Sensitivity** | **Total no. of studies** | **Specificity** | **Total no. of studies** | **Sensitivity** | **Total no. of studies** | **Specificity** |
| NAAT | 5 | 98  (93, 100) | 5 | 99  (97, 100) | 4 | 98  (94, 99) | 4 | 98  (95, 99) | 14 | 92  (88, 94) | 13 | 99  (95, 100) | 11 | 90  (83, 95) | 12 | 99  (96, 100) |
| NAAT (POC) | 0 | - | 0 | - | 7 | 96  (87, 99) | 7 | 100  (32, 100) | 1 | 100*  (89, 100) | 1 | 100*  (96, 100) | 1 | 100*  (91, 100) | 1 | 93*  (77, 99) |
| Serology IgG and/or IgM | 0 | - | 0 | - | 0 | - | 0 | - | 11 | 89  (81, 93) | 12 | 96  (73, 100) | 1 | 85*  (70, 94) | 2 | 100*  (93, 100)  50*  (12, 88) |
| Serology IgG and IgM | 0 | - | 0 | - | 0 | - | 0 | - | 4 | 55  (43, 67) | 4 | 95  (86, 98) | 0 | - | 1 | 83*  (36, 100) |
| Serology IgG | 0 | - | 0 | - | 0 | - | 0 | - | 17 | 80  (67, 89) | 17 | 97  (80, 100) | 3 | 79  (72, 85) | 3 | 97  (93, 99) |
| Serology IgM | 0 | - | 0 | - | 0 | - | 0 | - | 19 | 79  (71, 86) | 19 | 93  (71, 99) | 2 | 70* (53, 83)  63* (50, 74) | 2 | 94*  (85, 98)  50*  (12, 88) |
| Serology Ab | 0 | - | 0 | - | 2 | 68* (58, 77)  60* (50, 70) | 2 | 100*  (89, 100)  100*  (89, 100) | 9 | 98  (95, 99) | 10 | 99  (97, 100) | 1 | 93*  (78, 99) | 1 | 100*  (96, 100) |
| Serology IgA | 0 | - | 0 | - | 0 | - | 0 | - | 1 | 99*  (96, 100) | 1 | 98*  (96, 99) | 1 | 93*  (78, 99) | 1 | 93*  (85, 97) |
| Serology POC- IgG and/or IgM | 2 | 43*  (35, 52)  79*  (49, 95) | 2 | 98*  (89, 100)  100*  (88, 100) | 0 | - | 0 | - | 5 | 80  (70, 88) | 6 | 88  (74, 95) | 21 | 67  (57, 76) | 23 | 97  (92, 99) |
| Serology POC- IgG and IgM | 1 | 29*  (8, 58) | 1 | 100*  (88, 100) | 0 | - | 0 | - | 4 | 67  (40, 86) | 4 | 99  (86, 100) | 1 | 80*  (61, 92) | 2 | 100*  (88, 100)  83*  (36, 100) |
| Serology POC- IgG | 2 | 14*  (9, 21)  79*  (49, 95) | 2 | 100*  (93, 100)  100*  (88, 100) | 0 | - | 0 | - | 5 | 60  (19, 91) | 5 | 95  (62, 99) | 3 | 59  (15, 92) | 5 | 92  (27, 100) |
| Serology POC- IgM | 2 | 43*  (35, 52)  59*  (8, 58) | 2 | 98*  (89, 100)  100*(88, 100) | 0 | - | 0 | - | 5 | 49  (9, 91) | 5 | 91  (73, 97) | 3 | 35  (15, 64) | 5 | 96  (7, 100) |
| Imaging | 2 | 69*  (59, 77)  69*  (56, 80) | 0 | - | 0 | - | 0 | - | 8 | 85  (70, 94) | 5 | 56  (36, 74) | 4 | 83  (66, 93) | 3 | 70  (54, 82) |
| Imaging AI | 0 | - | 0 | - | 0 | - | 0 | - | 10 | 92  (87, 96) | 9 | 94  (88, 97) | 1 | 80*  (69, 88) | 1 | 81*  (64, 92) |
| Clinical and/or Laboratory | 0 | - | 0 | - | 0 | - | 0 | - | 3 | 89  (76, 95) | 3 | 82  (64, 93) | 1 | 78*  (73, 82) | 1 | 89*  (84, 93) |
| NAAT + Imaging | 1 | 59*  (47, 71) | 0 | - | 0 | - | 0 | - | 3 | 91  (84, 94) | 1 | 67*  (61, 72) | 0 | - | 0 | - |

* results were not pooled due to insufficient studies

## **Table S9: Pooled sensitivity and specificity estimates for different genes**

| **Gene** | **No. of Studies** | **Sensitivity** | **No. of Studies** | **Specificity** |
| --- | --- | --- | --- | --- |
| N | 11 | 96 (91, 98) | 10 | 99 (97, 100) |
| ORF1 | 12 | 97 (91, 99) | 11 | 99 (95, 100) |
| S | 2 | 77* (46, 95)  100* (94, 100) | 2 | 100* (63, 100)  100* (95, 100) |
| RdRp | 4 | 92 (77, 97) | 4 | 99 (77, 100) |
| E | 4 | 97 (74, 100) | 5 | 99 (96, 100) |
| Nsp2 | 1 | 100* (85, 100) | 1 | 100* (90, 100) |

* results were not pooled due to insufficient studies

## **Table S10: Pooled sensitivity and specificity estimates for different specimen sites**

| **Sites** | **No. of Studies** | **Sensitivity** | **No. of Studies** | **Specificity** |
| --- | --- | --- | --- | --- |
| Nasopharyngeal | 12 | 98 (93, 99) | 12 | 99 (96, 100) |
| Unspecified Throat | 18 | 96 (92, 98) | 18 | 97 (92, 99) |
| Nasal | 3 | 85 (53, 97) | 2 | 73* (39, 94)  100* (91, 100) |
| Sputum | 2 | 100* (75, 100)  90* (81, 95) | 1 | 90* (73, 98) |
| Saliva | 1 | 100* (91, 100) | 0 | - |
| Stool | 1 | 100* (40, 100) | 0 | - |

* results were not pooled due to insufficient studies

## **Table S11: QUADAS-2**

|  | **Risk of Bias** | | | | **Applicability Concerns** | | |
| --- | --- | --- | --- | --- | --- | --- | --- |
|  | **PATIENT SELECTION** | **INDEX TEST** | **REFERENCE STANDARD** | **FLOW AND TIMING** | **PATIENT SELECTION** | **INDEX TEST** | **REFERENCE STANDARD** |
| Adams et al., ELISA | High | Unclear | Unclear | High | High | High | Low |
| Adams et al., LFIA (POC) | High | Unclear | Unclear | High | High | Low | Low |
| Ai et al., CT | High | High | Unclear | High | Low | Low | Low |
| Ai et al., CT | High | Unclear | Unclear | Low | Low | Low | Low |
| Baek et al., RT-LAMP | High | Unclear | Unclear | High | High | High | Low |
| Bai et al., CT(AI) | High | High | Unclear | High | High | High | Low |
| Brinati et al., Laboratory Parameters | Unclear | High | Unclear | Unclear | Unclear | Low | Low |
| Broughton et al., Molecular | High | High | Unclear | Unclear | High | High | Low |
| Burbelo et al., LIPS | High | Unclear | Unclear | High | High | High | Low |
| Butt et al., RT-LAMP | High | Unclear | Unclear | Low | High | High | Low |
| Cai et al., MCLIA | High | Unclear | Unclear | High | High | High | Low |
| Caruso et al., CT | Low | Unclear | Unclear | High | Low | Low | Low |
| Cassaniti et al., LFIA (POC) | High | Unclear | Unclear | High | High | Low | Low |
| Castiglioni et al., X-Ray (POC, AI) | High | High | Unclear | Unclear | High | High | Low |
| Chan et al., rRT-PCR | High | Unclear | Unclear | High | High | High | Low |
| Chen et al., CT (AI) | High | High | Unclear | Unclear | High | High | Low |
| Chen et al., CT (AI) | High | High | Unclear | Unclear | High | High | Low |
| Chen et al., LFIA (POC) | High | High | Unclear | Unclear | High | High | Low |
| Diao et al., Fluorescence Immunochromatographic assay | High | Unclear | Unclear | Low | Low | High | Low |
| Döhla et al., SARS-CoV-2 rapid test, Qualitative IgG/IgM (POC) | High | Low | Unclear | Unclear | Low | Low | Low |
| Dong et al., RT-Dpcr | High | Unclear | Unclear | Unclear | High | High | Low |
| Freeman et al., ELISA | High | High | Unclear | High | High | High | Low |
| Fu et al., CT(AI) | High | High | Unclear | Unclear | High | High | Low |
| Gaeta et al., X-ray | High | Unclear | Unclear | Unclear | High | Low | Low |
| Garcia et al., LFIA (POC) | High | Unclear | Unclear | High | High | Low | Low |
| Gietema et al., CT | High | Unclear | Unclear | Low | Low | Low | Low |
| Guo et al., ELISA | High | Unclear | Unclear | High | High | High | Low |
| Hirotsu et al., RT-PCR, double-quencher probes | Unclear | Unclear | Unclear | Low | Low | Unclear | Low |
| Hoffman et al., IgM and IgG rapid test (POC) | High | Unclear | Unclear | High | High | Low | Low |
| Hou et al., CRIPSR | High | High | High | Unclear | High | High | Low |
| Hou et al., PCR | High | Unclear | High | Unclear | High | Unclear | Low |
| Huang et al., RT-LAMP | Unclear | Unclear | Low | Low | Low | High | Low |
| Imai et al., IC(POC) + CT | High | Unclear | Unclear | High | High | Low | Low |
| Imai et al., IC (POC) only | High | Unclear | Unclear | High | High | Low | Low |
| Imai et al., CT only | High | Unclear | Unclear | High | High | Low | Low |
| Infantino et al., CLIA | High | Low | Unclear | High | High | Low | Low |
| Jia et al., IC | Unclear | High | Unclear | Unclear | Low | Low | Low |
| Jiang et al., CT | Low | High | Unclear | High | Low | Low | Low |
| Jiang et al., RT-LAMP | Unclear | Unclear | Unclear | Low | Low | High | Low |
| Jin et al., CT(AI) | High | High | Unclear | High | High | High | Low |
| Jin et al., CT (AI) | High | High | Unclear | Unclear | High | High | Low |
| Jin et al., CLIA | High | Low | Unclear | Unclear | Low | Low | Low |
| Kurstjens et al., Clinical features | Unclear | High | Unclear | Unclear | Low | Low | Low |
| Lassaunière et al., LFIA (POC) | High | Unclear | Unclear | High | High | Low | Low |
| Lassaunière et al., ELISA | High | Unclear | Unclear | High | High | Low | Low |
| Lee et al., LFIA (POC) | High | Unclear | Low | High | High | Low | Low |
| Li et al., CT (AI) | High | High | Unclear | High | High | High | Low |
| Li et al., Clinical + Lab | High | Unclear | Unclear | Unclear | High | High | Low |
| Li et al., LFIA (POC) | Unclear | Unclear | Unclear | Unclear | Low | Low | Low |
| Lin et al., CLIA | High | High | Low | Unclear | High | High | Low |
| Lin et al., ELISA | High | Unclear | Low | High | High | Low | Low |
| Lin et al., CLIA+ ELISA | High | High | Low | High | High | High | Low |
| Liu et al., ELISA | High | Unclear | Unclear | High | High | Low | Low |
| Liu et al., ELISA | High | Unclear | Unclear | High | High | Low | Low |
| Liu et al., Serology (POC) | Unclear | Unclear | Unclear | Unclear | Low | Low | Low |
| Long et al., CT | High | Unclear | Unclear | High | Low | Low | Low |
| Long et al., PCR+CT | High | High | Unclear | High | Low | Low | Low |
| Long et al., MCLIA | High | High | Unclear | High | High | Low | Low |
| Lou et al., ELISA | High | Unclear | Unclear | High | High | Low | Low |
| Lou et al., CMIA | High | Unclear | Unclear | High | High | Low | Low |
| Lou et al., LFIA (POC) | High | Unclear | Unclear | High | High | Low | Low |
| Lou et al., ELISA+CMIA+LFIA(POC) | High | Unclear | Unclear | High | High | Low | Low |
| Lu et al., Dpcr | Unclear | Unclear | Unclear | Unclear | Low | Low | Low |
| Lu et al., RT-LAMP | High | Unclear | Unclear | Low | High | High | Low |
| Ma et al., Chemical luminescence | High | Unclear | Unclear | High | High | High | Low |
| Meng et al., Lab Features (AI) | High | High | Unclear | Unclear | High | High | Low |
| Merindol et al., RT-QPCR | High | Unclear | Unclear | Low | High | Low | Low |
| Miao et al., CT | Low | High | Unclear | Unclear | Low | Low | Low |
| Noerz et al., Molecular | Unclear | Unclear | Unclear | High | Unclear | High | Low |
| Osterdahl et al., RT-LAMP | High | Unclear | Unclear | Low | Low | Low | Low |
| Padoan et al., CLIA | High | Unclear | Unclear | Unclear | High | Low | Low |
| Pan et al., ICG strip assay | Unclear | Unclear | Unclear | High | High | Low | Low |
| Paradiso et al., Serology (POC) | Low | Low | Unclear | High | Low | Low | Low |
| Paradiso et al., CLIA (POC) | Low | Low | High | High | Low | Low | Low |
| Poljak et al., Fully automated high throughput PCR | Unclear | Low | Unclear | High | Low | Low | Low |
| Qian et al., CLIA | High | Unclear | Unclear | High | High | High | Low |
| Shen et al., IC (POC) | High | Unclear | Unclear | Unclear | High | Low | Low |
| Smithgall et al., Molecular (Xpert Xpress) | Unclear | Unclear | Unclear | Low | Low | Low | Low |
| Smithgall et al., Molecular (ID Now) | Unclear | Unclear | Unclear | Low | Low | Low | Low |
| Song et al., CT (AI) | High | High | Unclear | Unclear | High | High | Low |
| Spicuzza et al., IC (POC) | High | High | Unclear | Unclear | High | Low | Low |
| van Kasteren et al., RT-PCR | High | Unclear | High | High | High | Low | Low |
| Mertens et al., IC (POC) | Unclear | Unclear | Unclear | Low | Low | High | Low |
| Visseaux et al., Multiplex PCR (POC) | Unclear | Unclear | Unclear | Low | Low | Low | Low |
| Wang et al., Nanopore Target Sequencing | High | Unclear | Unclear | High | High | High | Low |
| Wang et al., CT (AI) | High | High | Unclear | High | High | High | Low |
| Wang et al., CT (AI) | High | High | Unclear | High | High | High | Low |
| Wong et al., X-Ray | High | Unclear | Unclear | Low | High | Low | Low |
| Wong et al., X-ray+PCR | High | High | Unclear | Low | High | Low | Low |
| Wu et al., Laboratory Parameters (AI) | High | High | Unclear | Unclear | High | High | Low |
| Wu et al., Solid-phase immunochromatography | Unclear | Unclear | Unclear | Unclear | Low | Low | Low |
| Xiang et al., ELISA | High | Unclear | Unclear | High | High | Low | Low |
| Xie et al., Chemiluminescence immunoassay | Unclear | Unclear | Unclear | Unclear | Low | Low | Low |
| Xie et al.,CT | High | Unclear | Unclear | Low | Unclear | Low | Low |
| Xie et al., PCR+CT | High | High | Unclear | Low | Unclear | Low | Low |
| Xu et al., CT (AI) | High | Low | Unclear | High | High | High | Low |
| Xu et al., GICA | High | Unclear | Unclear | High | High | Low | Low |
| Xu et al., ELISA | High | Unclear | Unclear | High | High | Low | Low |
| Yan et al., RT-LAMP | Unclear | Unclear | Unclear | Low | Unclear | High | Low |
| Yip et al., rRT-PCR | High | Unclear | Unclear | Unclear | Unclear | High | Low |
| Yu et al., dd PCR | Unclear | Unclear | Unclear | High | Low | Low | Low |
| Yu et al., RT-LAMP | High | Unclear | Unclear | Unclear | Unclear | High | Low |
| Zhang et al., CLIA (POC) | High | Unclear | Low | High | High | Low | Low |
| Zhang et al., GICA (POC) | Unclear | Unclear | Unclear | Unclear | Low | High | Low |
| Zhang et al., RT-LAMP | Unclear | Unclear | Unclear | Low | Unclear | High | Low |
| Zhao et al., ELISA | High | Unclear | Unclear | High | High | Low | Low |
| Zhen et al., RT-PCR | Unclear | Unclear | High | Low | Low | Low | Low |
| Zhen et al., RT-PCR (POC) | Unclear | Unclear | High | Low | Low | Low | Low |
| Zhen et al., RT-LAMP (POC) | Unclear | Unclear | Unclear | High | Unclear | Low | Low |
| Zhen et al., RT-PCR (POC) | Unclear | Unclear | Unclear | Low | Unclear | Low | Low |
| Zhong et al., ELISA | High | High | Unclear | High | High | High | Low |
| Zhong et al., chemiluminescence | High | High | Unclear | High | High | High | Low |
| Zhu et al., mRT-LAMP-LFB | Unclear | Unclear | Unclear | Low | High | High | Low |

References

1. Adams ER, Ainsworth M, Anand R, Andersson MI, Auckland K, Baillie JK, et al. Antibody testing for COVID-19: A report from the National COVID Scientific Advisory Panel. *medRxiv* (2020):2020.04.15.20066407. doi: 10.1101/2020.04.15.20066407.
2. Ai J, Gong J, Xing L, He R, Tian F, Wang J, et al. Analysis of factors associated early diagnosis in coronavirus disease 2019 (COVID-19). *medRxiv* (2020):2020.04.09.20059352. doi: 10.1101/2020.04.09.20059352.
3. Ai T, Yang Z, Hou H, Zhan C, Chen C, Lv W, et al. Correlation of Chest CT and RT-PCR Testing for Coronavirus Disease 2019 (COVID-19) in China: A Report of 1014 Cases. *Radiology* (2020) 296(2):E32-e40. Epub 2020/02/27. doi: 10.1148/radiol.2020200642. PubMed PMID: 32101510; PubMed Central PMCID: PMCPMC7233399.
4. Baek YH, Um J, Antigua KJC, Park JH, Kim Y, Oh S, et al. Development of a reverse transcription-loop-mediated isothermal amplification as a rapid early-detection method for novel SARS-CoV-2. *Emerging microbes & infections* (2020):1-31. Epub 2020/04/21. doi: 10.1080/22221751.2020.1756698. PubMed PMID: 32306853.
5. Bai HX, Wang R, Xiong Z, Hsieh B, Chang K, Halsey K, et al. AI Augmentation of Radiologist Performance in Distinguishing COVID-19 from Pneumonia of Other Etiology on Chest CT. *Radiology* (2020):201491. Epub 2020/04/28. doi: 10.1148/radiol.2020201491. PubMed PMID: 32339081; PubMed Central PMCID: PMCselected.
6. Brinati D, Campagner A, Ferrari D, Locatelli M, Banfi G, Cabitza F. Detection of COVID-19 Infection from Routine Blood Exams with Machine Learning: A Feasibility Study. *Journal of Medical Systems* (2020) 44(8):135. doi: 10.1007/s10916-020-01597-4.
7. Broughton JP, Deng X, Yu G, Fasching CL, Servellita V, Singh J, et al. CRISPR-Cas12-based detection of SARS-CoV-2. *Nature biotechnology* (2020). Epub 2020/04/18. doi: 10.1038/s41587-020-0513-4. PubMed PMID: 32300245.
8. Burbelo PD, Riedo FX, Morishima C, Rawlings S, Smith D, Das S, et al. Sensitivity in Detection of Antibodies to Nucleocapsid and Spike Proteins of Severe Acute Respiratory Syndrome Coronavirus 2 in Patients With Coronavirus Disease 2019. *J Infect Dis* (2020) 222(2):206-13. Epub 2020/05/20. doi: 10.1093/infdis/jiaa273. PubMed PMID: 32427334; PubMed Central PMCID: PMCPMC7313936.
9. Butt AM, Siddique S, An X, Tong Y. Development of a dual-gene loop-mediated isothermal amplification (LAMP) detection assay for SARS-CoV-2: A preliminary study. *medRxiv* (2020):2020.04.08.20056986. doi: 10.1101/2020.04.08.20056986.
10. Cai X-f, Chen J, li Hu J-, Long Q-x, Deng H-j, Liu P, et al. A Peptide-Based Magnetic Chemiluminescence Enzyme Immunoassay for Serological Diagnosis of Coronavirus Disease 2019. *The Journal of Infectious Diseases* (2020) 222(2):189-93. doi: 10.1093/infdis/jiaa243.
11. Caruso D, Zerunian M, Polici M, Pucciarelli F, Polidori T, Rucci C, et al. Chest CT Features of COVID-19 in Rome, Italy. *Radiology* (2020):201237. Epub 2020/04/04. doi: 10.1148/radiol.2020201237. PubMed PMID: 32243238.
12. Cassaniti I, Novazzi F, Giardina F, Salinaro F, Sachs M, Perlini S, et al. Performance of VivaDiag COVID-19 IgM/IgG Rapid Test is inadequate for diagnosis of COVID-19 in acute patients referring to emergency room department. *Journal of medical virology* (2020):10.1002/jmv.25800. doi: 10.1002/jmv.25800. PubMed PMID: 32227490.
13. Castiglioni I, Ippolito D, Interlenghi M, Monti CB, Salvatore C, Schiaffino S, et al. Artificial intelligence applied on chest X-ray can aid in the diagnosis of COVID-19 infection: a first experience from Lombardy, Italy. *medRxiv* (2020):2020.04.08.20040907. doi: 10.1101/2020.04.08.20040907.
14. Chen J, Wu L, Zhang J, Zhang L, Gong D, Zhao Y, et al. Deep learning-based model for detecting 2019 novel coronavirus pneumonia on high-resolution computed tomography: a prospective study. *medRxiv* (2020):2020.02.25.20021568. doi: 10.1101/2020.02.25.20021568.
15. Chen X, Tang Y, Mo Y, Li S, Lin D, Yang Z, et al. A diagnostic model for coronavirus disease 2019 (COVID-19) based on radiological semantic and clinical features: a multi-center study. *European Radiology* (2020). doi: 10.1007/s00330-020-06829-2.
16. Chen Z, Zhang Z, Zhai X, Li Y, Lin L, Zhao H, et al. Rapid and sensitive detection of anti-SARS-CoV-2 IgG using lanthanide-doped nanoparticles-based lateral flow immunoassay. *Anal Chem* (2020). Epub 2020/04/24. doi: 10.1021/acs.analchem.0c00784. PubMed PMID: 32323974.
17. Diao B, Wen K, Chen J, Liu Y, Yuan Z, Han C, et al. Diagnosis of Acute Respiratory Syndrome Coronavirus 2 Infection by Detection of Nucleocapsid Protein. *medRxiv* (2020):2020.03.07.20032524. doi: 10.1101/2020.03.07.20032524.
18. Döhla M, Boesecke C, Schulte B, Diegmann C, Sib E, Richter E, et al. Rapid point-of-care testing for SARS-CoV-2 in a community screening setting shows low sensitivity. *Public Health* (2020) 182:170-2. Epub 2020/04/26. doi: 10.1016/j.puhe.2020.04.009. PubMed PMID: 32334183; PubMed Central PMCID: PMCPMC7165286.
19. Dong L, Zhou J, Niu C, Wang Q, Pan Y, Sheng S, et al. Highly accurate and sensitive diagnostic detection of SARS-CoV-2 by digital PCR. *medRxiv* (2020):2020.03.14.20036129. doi: 10.1101/2020.03.14.20036129.
20. Freeman B, Lester S, Mills L, Rasheed MAU, Moye S, Abiona O, et al. Validation of a SARS-CoV-2 spike protein ELISA for use in contact investigations and sero-surveillance. *bioRxiv* (2020):2020.04.24.057323. doi: 10.1101/2020.04.24.057323.
21. Fu M, Yi S-L, Zeng Y, Ye F, Li Y, Dong X, et al. Deep Learning-Based Recognizing COVID-19 and other Common Infectious Diseases of the Lung by Chest CT Scan Images. *medRxiv* (2020):2020.03.28.20046045. doi: 10.1101/2020.03.28.20046045.
22. Gaeta M, Cicero G, Marino MA, Angelo T, Mormina EM, Mazziotti S, et al. EFFECTIVENESS OF BASELINE AND POST-PROCESSED CHEST X-RAY IN NONEARLY COVID-19 PATIENTS. *medRxiv* (2020):2020.04.16.20061044. doi: 10.1101/2020.04.16.20061044.
23. Pérez-García F, Pérez-Tanoira R, Romanyk J, Arroyo T, Gómez-Herruz P, Cuadros-González J. Alltest rapid lateral flow immunoassays is reliable in diagnosing SARS-CoV-2 infection from 14 days after symptom onset: A prospective single-center study. *J Clin Virol* (2020) 129:104473. Epub 2020/06/07. doi: 10.1016/j.jcv.2020.104473. PubMed PMID: 32504945; PubMed Central PMCID: PMCPMC7255149.
24. Gietema HA, Zelis N, Nobel JM, Lambriks LJG, van Alphen LB, Oude Lashof AML, et al. CT in relation to RT-PCR in diagnosing COVID-19 in The Netherlands: A prospective study. *PLoS One* (2020) 15(7):e0235844. Epub 2020/07/10. doi: 10.1371/journal.pone.0235844. PubMed PMID: 32645053; PubMed Central PMCID: PMCPMC7347219.
25. Guo L, Ren L, Yang S, Xiao M, Chang, Yang F, et al. Profiling Early Humoral Response to Diagnose Novel Coronavirus Disease (COVID-19). *Clinical infectious diseases : an official publication of the Infectious Diseases Society of America* (2020). doi: 10.1093/cid/ciaa310.
26. Hirotsu Y, Mochizuki H, Omata M. Double-quencher probes improve detection sensitivity toward Severe Acute Respiratory Syndrome Coronavirus 2 (SARS-CoV-2) in a reverse-transcription polymerase chain reaction (RT-PCR) assay. *Journal of Virological Methods* (2020) 284:113926. doi: h<ttps://doi.org/10.1016/j.jviromet.2020.113926.>
27. Hoffman T, Nissen K, Krambrich J, Rönnberg B, Akaberi D, Esmaeilzadeh M, et al. Evaluation of a COVID-19 IgM and IgG rapid test; an efficient tool for assessment of past exposure to SARS-CoV-2. *Infection Ecology and Epidemiology* (2020) 10(1). doi: 10.1080/20008686.2020.1754538.
28. Hou T, Zeng W, Yang M, Chen W, Ren L, Ai J, et al. Development and Evaluation of A CRISPR-based Diagnostic For 2019-novel Coronavirus. *medRxiv* (2020):2020.02.22.20025460. doi: 10.1101/2020.02.22.20025460.
29. Huang WE, Lim B, Hsu CC, Xiong D, Wu W, Yu Y, et al. RT-LAMP for rapid diagnosis of coronavirus SARS-CoV-2. *Microbial biotechnology* (2020). Epub 2020/04/26. doi: 10.1111/1751-7915.13586. PubMed PMID: 32333644.
30. Imai K, Tabata S, Ikeda M, Noguchi S, Kitagawa Y, Matuoka M, et al. Clinical evaluation of an immunochromatographic IgM/IgG antibody assay and chest computed tomography for the diagnosis of COVID-19. *Journal of Clinical Virology* (2020) 128:104393. doi: h<ttps://doi.org/10.1016/j.jcv.2020.104393.>
31. Infantino M, Grossi V, Lari B, Bambi R, Perri A, Manneschi M, et al. Diagnostic accuracy of an automated chemiluminescent immunoassay for anti-SARS-CoV-2 IgM and IgG antibodies: an Italian experience. *Journal of medical virology* (2020). Epub 2020/04/25. doi: 10.1002/jmv.25932. PubMed PMID: 32330291.
32. Jia X, Zhang P, Tian Y, Wang J, Zeng H, Wang J, et al. Clinical significance of IgM and IgG test for diagnosis of highly suspected COVID-19 infection. *medRxiv* (2020):2020.02.28.20029025. doi: 10.1101/2020.02.28.20029025.
33. Jiang G, Ren X, Liu Y, Chen H, Liu W, Guo Z, et al. Application and optimization of RT-PCR in diagnosis of SARS-CoV-2 infection. *medRxiv* (2020):2020.02.25.20027755. doi: 10.1101/2020.02.25.20027755.
34. Jiang M, Pan W, Arasthfer A, Fang W, Ling L, Fang H, et al. Development and Validation of a Rapid, Single-Step Reverse Transcriptase Loop-Mediated Isothermal Amplification (RT-LAMP) System Potentially to Be Used for Reliable and High-Throughput Screening of COVID-19. *Frontiers in Cellular and Infection Microbiology* (2020) 10(331). doi: 10.3389/fcimb.2020.00331.
35. Jin C, Chen W, Cao Y, Xu Z, Zhang X, Deng L, et al. Development and Evaluation of an AI System for COVID-19 Diagnosis. *medRxiv* (2020):2020.03.20.20039834. doi: 10.1101/2020.03.20.20039834.
36. Jin S, Wang B, Xu H, Luo C, Wei L, Zhao W, et al. AI-assisted CT imaging analysis for COVID-19 screening: Building and deploying a medical AI system in four weeks. *medRxiv* (2020):2020.03.19.20039354. doi: 10.1101/2020.03.19.20039354.
37. Jin Y, Wang M, Zuo Z, Fan C, Ye F, Cai Z, et al. Diagnostic value and dynamic variance of serum antibody in coronavirus disease 2019. *International Journal of Infectious Diseases* (2020) 94:49-52. doi: 10.1016/j.ijid.2020.03.065.
38. Lassaunière R, Frische A, Harboe ZB, Nielsen ACY, Fomsgaard A, Krogfelt KA, et al. Evaluation of nine commercial SARS-CoV-2 immunoassays. *medRxiv* (2020):2020.04.09.20056325. doi: 10.1101/2020.04.09.20056325.
39. Lee Y-L, Liao C-H, Liu P-Y, Cheng C-Y, Chung M-Y, Liu C-E, et al. Dynamics of anti-SARS-Cov-2 IgM and IgG antibodies among COVID-19 patients. *The Journal of infection* (2020):S0163-4453(20)30230-9. doi: 10.1016/j.jinf.2020.04.019. PubMed PMID: 32335168.
40. Li L, Qin L, Xu Z, Yin Y, Wang X, Kong B, et al. Artificial Intelligence Distinguishes COVID-19 from Community Acquired Pneumonia on Chest CT. *Radiology* (2020):200905. Epub 2020/03/20. doi: 10.1148/radiol.2020200905. PubMed PMID: 32191588; PubMed Central PMCID: PMCselected.
41. Li Z, Yi Y, Luo X, Xiong N, Liu Y, Li S, et al. Development and clinical application of a rapid IgM-IgG combined antibody test for SARS-CoV-2 infection diagnosis. *Journal of Medical Virology* (2020). doi: 10.1002/jmv.25727.
42. Lin D, Liu L, Zhang M, Hu Y, Yang Q, Guo J, et al. Evaluations of the serological test in the diagnosis of 2019 novel coronavirus (SARS-CoV-2) infections during the COVID-19 outbreak. *European Journal of Clinical Microbiology & Infectious Diseases* (2020) 39(12):2271-7. doi: 10.1007/s10096-020-03978-6.
43. Liu L, Liu W, Zheng Y, Jiang X, Kou G, Ding J, et al. A preliminary study on serological assay for severe acute respiratory syndrome coronavirus 2 (SARS-CoV-2) in 238 admitted hospital patients. *Microbes and Infection* (2020) 22(4):206-11. doi: h<ttps://doi.org/10.1016/j.micinf.2020.05.008.>
44. Liu W, Liu L, Kou G, Zheng Y, Ding Y, Ni W, et al. Evaluation of Nucleocapsid and Spike Protein-based ELISAs for detecting antibodies against SARS-CoV-2. *Journal of clinical microbiology* (2020). doi: 10.1128/JCM.00461-20.
45. Liu Y, Liu Y, Diao B, Ren F, Wang Y, Ding J, et al. Diagnostic Indexes of a Rapid IgG/IgM Combined Antibody Test for SARS-CoV-2. *medRxiv* (2020):2020.03.26.20044883. doi: 10.1101/2020.03.26.20044883.
46. Long C, Xu H, Shen Q, Zhang X, Fan B, Wang C, et al. Diagnosis of the Coronavirus disease (COVID-19): rRT-PCR or CT? *European journal of radiology* (2020):108961.
47. Long Q-X, Liu B-Z, Deng H-J, Wu G-C, Deng K, Chen Y-K, et al. Antibody responses to SARS-CoV-2 in patients with COVID-19. *Nature Medicine* (2020) 26(6):845-8. doi: 10.1038/s41591-020-0897-1.
48. Lou B, Li T-D, Zheng S-F, Su Y-Y, Li Z-Y, Liu W, et al. Serology characteristics of SARS-CoV-2 infection after exposure and post-symptom onset. *European Respiratory Journal* (2020) 56(2):2000763. doi: 10.1183/13993003.00763-2020.
49. Lu R, Wang J, Li M, Wang Y, Dong J, Cai W. SARS-CoV-2 detection using digital PCR for COVID-19 diagnosis, treatment monitoring and criteria for discharge. *medRxiv* (2020):2020.03.24.20042689. doi: 10.1101/2020.03.24.20042689.
50. Lu R, Wu X, Wan Z, Li Y, Jin X, Zhang C. A Novel Reverse Transcription Loop-Mediated Isothermal Amplification Method for Rapid Detection of SARS-CoV-2. *Int J Mol Sci* (2020) 21(8). Epub 2020/04/25. doi: 10.3390/ijms21082826. PubMed PMID: 32325642; PubMed Central PMCID: PMCPMC7216271.
51. Ma H, Zeng W, He H, Zhao D, Jiang D, Zhou P, et al. Serum IgA, IgM, and IgG responses in COVID-19. *Cellular & Molecular Immunology* (2020) 17(7):773-5. doi: 10.1038/s41423-020-0474-z.
52. Merindol N, Pépin G, Marchand C, Rheault M, Peterson C, Poirier A, et al. Optimization of SARS-CoV-2 detection by RT-QPCR without RNA extraction. *bioRxiv* (2020):2020.04.06.028902. doi: 10.1101/2020.04.06.028902.
53. Miao C, Jin M, Miao L, Yang X, Huang P, Xiong H, et al. Early chest computed tomography to diagnose COVID-19 from suspected patients: A multicenter retrospective study. *American Journal of Emergency Medicine* (2020). doi: 10.1016/j.ajem.2020.04.051.
54. Nörz D, Fischer N, Schultze A, Kluge S, Mayer-Runge U, Aepfelbacher M, et al. Clinical evaluation of a SARS-CoV-2 RT-PCR assay on a fully automated system for rapid on-demand testing in the hospital setting. *Journal of Clinical Virology* (2020) 128:104390. doi: h<ttps://doi.org/10.1016/j.jcv.2020.104390.>
55. Osterdahl MF, Lee KA, Ni Lochlainn M, Wilson S, Douthwaite S, Horsfall R, et al. Detecting SARS-CoV-2 at point of care: Preliminary data comparing Loop-mediated isothermal amplification (LAMP) to PCR. *medRxiv* (2020):2020.04.01.20047357. doi: 10.1101/2020.04.01.20047357.
56. Padoan A, Cosma C, Sciacovelli L, Faggian D, Plebani M. Analytical performances of a chemiluminescence immunoassay for SARS-CoV-2 IgM/IgG and antibody kinetics. *Clinical chemistry and laboratory medicine* (2020). doi: 10.1515/cclm-2020-0443.
57. Pan Y, Li X, Yang G, Fan J, Tang Y, Zhao J, et al. Serological immunochromatographic approach in diagnosis with SARS-CoV-2 infected COVID-19 patients. *Journal of Infection* (2020). doi: 10.1016/j.jinf.2020.03.051.
58. Paradiso AV, De Summa S, Loconsole D, Procacci V, Sallustio A, Centrone F, et al. Clinical meanings of rapid serological assay in patients tested for SARS-Co2 RT-PCR. *medRxiv* (2020):2020.04.03.20052183. doi: 10.1101/2020.04.03.20052183.
59. Paradiso AV, De Summa s, Silvestris N, Tommasi S, Tufaro A, De Palma G, et al. RAPID SEROLOGICAL TESTS HAVE A ROLE IN ASYMPTOMATIC HEALTH WORKERS COVID-19 SCREENING. *medRxiv* (2020):2020.04.15.20057786. doi: 10.1101/2020.04.15.20057786.
60. Poljak M, Korva M, Knap Gašper N, Fujs Komloš K, Sagadin M, Uršič T, et al. Clinical evaluation of the cobas SARS-CoV-2 test and a diagnostic platform switch during 48 hours in the midst of the COVID-19 pandemic. *J Clin Microbiol* (2020). Epub 2020/04/12. doi: 10.1128/jcm.00599-20. PubMed PMID: 32277022.
61. Qian C, Zhou M, Cheng F, Lin X, Gong Y, Xie X, et al. Development and multicenter performance evaluation of fully automated SARS-CoV-2 IgM and IgG immunoassays. *Clinical Chemistry and Laboratory Medicine (CCLM)* (2020) 58(9):1601. doi: h<ttps://doi.org/10.1515/cclm-2020-0548.>
62. Shen B, Zheng Y, Zhang X, Zhang W, Wang D, Jin J, et al. Clinical evaluation of a rapid colloidal gold immunochromatography assay for SARS-Cov-2 IgM/IgG. *Am J Transl Res* (2020) 12(4):1348-54. PubMed PMID: 32355546.
63. Smithgall MC, Scherberkova I, Whittier S, Green DA. Comparison of Cepheid Xpert Xpress and Abbott ID Now to Roche cobas for the Rapid Detection of SARS-CoV-2. *Journal of Clinical Virology* (2020) 128:104428. doi: h<ttps://doi.org/10.1016/j.jcv.2020.104428.>
64. Song Y, Zheng S, Li L, Zhang X, Zhang X, Huang Z, et al. Deep learning Enables Accurate Diagnosis of Novel Coronavirus (COVID-19) with CT images. *medRxiv* (2020):2020.02.23.20026930. doi: 10.1101/2020.02.23.20026930.
65. Spicuzza L, Montineri A, Manuele R, Crimi C, Pistorio MP, Campisi R, et al. Reliability and usefulness of a rapid IgM-IgG antibody test for the diagnosis of SARS-CoV-2 infection: A preliminary report. *The Journal of infection* (2020):S0163-4453(20)30231-0. doi: 10.1016/j.jinf.2020.04.022. PubMed PMID: 32335175.
66. van Kasteren PB, van der Veer B, van den Brink S, Wijsman L, de Jonge J, van den Brandt A, et al. Comparison of seven commercial RT-PCR diagnostic kits for COVID-19. *Journal of Clinical Virology* (2020) 128:104412. doi: h<ttps://doi.org/10.1016/j.jcv.2020.104412.>
67. Mertens P, De Vos N, Martiny D, Jassoy C, Mirazimi A, Cuypers L, et al. Development and Potential Usefulness of the COVID-19 Ag Respi-Strip Diagnostic Assay in a Pandemic Context. *Frontiers in Medicine* (2020) 7(225). doi: 10.3389/fmed.2020.00225.
68. Visseaux B, Le Hingrat Q, Collin G, Bouzid D, Lebourgeois S, Le Pluart D, et al. Evaluation of the QIAstat-Dx Respiratory SARS-CoV-2 Panel, the first rapid multiplex PCR commercial assay for SARS-CoV-2 detection. *J Clin Microbiol* (2020). Epub 2020/04/29. doi: 10.1128/jcm.00630-20. PubMed PMID: 32341142.
69. Wang M, Fu A, Hu B, Tong Y, Liu R, Liu Z, et al. Nanopore Targeted Sequencing for the Accurate and Comprehensive Detection of SARS-CoV-2 and Other Respiratory Viruses. *Small* (2020) 16(32):2002169. doi: h<ttps://doi.org/10.1002/smll.202002169.>
70. Wang S, Kang B, Ma J, Zeng X, Xiao M, Guo J, et al. A deep learning algorithm using CT images to screen for Corona Virus Disease (COVID-19). *medRxiv* (2020):2020.02.14.20023028. doi: 10.1101/2020.02.14.20023028.
71. Wang S, Zha Y, Li W, Wu Q, Li X, Niu M, et al. A fully automatic deep learning system for COVID-19 diagnostic and prognostic analysis. *European Respiratory Journal* (2020) 56(2):2000775. doi: 10.1183/13993003.00775-2020.
72. Wong HYF, Lam HYS, Fong AH-T, Leung ST, Chin TW-Y, Lo CSY, et al. Frequency and Distribution of Chest Radiographic Findings in COVID-19 Positive Patients. *Radiology* 0(0):201160. doi: 10.1148/radiol.2020201160. PubMed PMID: 32216717.
73. Wu X, Fu B, Chen L, Feng Y. Serological tests facilitate identification of asymptomatic SARS-CoV-2 infection in Wuhan, China. *Journal of medical virology* (2020). Epub 2020/04/21. doi: 10.1002/jmv.25904. PubMed PMID: 32311142.
74. Xiang F, Wang X, He X, Peng Z, Yang B, Zhang J, et al. Antibody Detection and Dynamic Characteristics in Patients with COVID-19. *Clinical infectious diseases : an official publication of the Infectious Diseases Society of America* (2020). Epub 2020/04/20. doi: 10.1093/cid/ciaa461. PubMed PMID: 32306047.
75. Xie J, Ding C, Li J, Wang Y, Guo H, Lu Z, et al. Characteristics of Patients with Coronavirus Disease (COVID-19) Confirmed using an IgM-IgG Antibody Test. *Journal of medical virology* (2020). Epub 2020/04/25. doi: 10.1002/jmv.25930. PubMed PMID: 32330303.
76. Xie X, Zhong Z, Zhao W, Zheng C, Wang F, Liu J. Chest CT for Typical 2019-nCoV Pneumonia: Relationship to Negative RT-PCR Testing. *Radiology* (2020):200343. doi: 10.1148/radiol.2020200343.
77. Xu X, Jiang X, Ma C, Du P, Li X, Lv S, et al. A Deep Learning System to Screen Novel Coronavirus Disease 2019 Pneumonia. *Engineering* (2020) 6(10):1122-9. doi: h<ttps://doi.org/10.1016/j.eng.2020.04.010.>
78. Xu Y, Xiao M, Liu X, Xu S, Du T, Xu J, et al. Significance of Serology Testing to Assist Timely Diagnosis of SARS-CoV-2 infections: Implication from a Family Cluster. *Emerging microbes & infections* (2020):1-12. Epub 2020/04/15. doi: 10.1080/22221751.2020.1752610. PubMed PMID: 32286155.
79. Yan C, Cui J, Huang L, Du B, Chen L, Xue G, et al. Rapid and visual detection of 2019 novel coronavirus (SARS-CoV-2) by a reverse transcription loop-mediated isothermal amplification assay. *Clinical Microbiology and Infection* (2020). doi: 10.1016/j.cmi.2020.04.001.
80. Yip CC, Ho CC, Chan JF, To KK, Chan HS, Wong SC, et al. Development of a Novel, Genome Subtraction-Derived, SARS-CoV-2-Specific COVID-19-nsp2 Real-Time RT-PCR Assay and Its Evaluation Using Clinical Specimens. *Int J Mol Sci* (2020) 21(7). Epub 2020/04/12. doi: 10.3390/ijms21072574. PubMed PMID: 32276333; PubMed Central PMCID: PMCPMC7177594.
81. Yu L, Wu S, Hao X, Dong X, Mao L, Pelechano V, et al. Rapid Detection of COVID-19 Coronavirus Using a Reverse Transcriptional Loop-Mediated Isothermal Amplification (RT-LAMP) Diagnostic Platform. *Clinical Chemistry* (2020) 66(7):975-7. doi: 10.1093/clinchem/hvaa102.
82. Zhang J, Zhang X, Liu J, Ban Y, Li N, Wu Y, et al. Serological detection of 2019-nCoV respond to the epidemic: A useful complement to nucleic acid testing. *International Immunopharmacology* (2020) 88:106861. doi: h<ttps://doi.org/10.1016/j.intimp.2020.106861.>
83. Zhang P, Gao Q, Wang T, Ke Y, Mo F, Jia R, et al. Evaluation of recombinant nucleocapsid and spike proteins for serological diagnosis of novel coronavirus disease 2019 (COVID-19). *medRxiv* (2020):2020.03.17.20036954. doi: 10.1101/2020.03.17.20036954.
84. Zhang Y, Odiwuor N, Xiong J, Sun L, Nyaruaba RO, Wei H, et al. Rapid Molecular Detection of SARS-CoV-2 (COVID-19) Virus RNA Using Colorimetric LAMP. *medRxiv* (2020):2020.02.26.20028373. doi: 10.1101/2020.02.26.20028373.
85. Zhen W, Manji R, Smith E, Berry GJ. Comparison of Four Molecular In Vitro Diagnostic Assays for the Detection of SARS-CoV-2 in Nasopharyngeal Specimens. *J Clin Microbiol* (2020). Epub 2020/04/29. doi: 10.1128/jcm.00743-20. PubMed PMID: 32341143.
86. Zhen W, Smith E, Manji R, Schron D, Berry GJ. Clinical Evaluation of Three Sample-To-Answer Platforms for the Detection of SARS-CoV-2. *J Clin Microbiol* (2020). Epub 2020/04/26. doi: 10.1128/jcm.00783-20. PubMed PMID: 32332061.
87. Zhong L, Chuan J, Gong B, Shuai P, Zhou Y, Zhang Y, et al. Detection of serum IgM and IgG for COVID-19 diagnosis. *Science China Life Sciences* (2020) 63(5):777-80. doi: 10.1007/s11427-020-1688-9.
88. Zhu X, Wang X, Han L, Chen T, Wang L, Li H, et al. Multiplex reverse transcription loop-mediated isothermal amplification combined with nanoparticle-based lateral flow biosensor for the diagnosis of COVID-19. *Biosensors and Bioelectronics* (2020) 166:112437. doi: <https://doi.org/10.1016/j.bios.2020.112437>.
